# Supplementary material for: Critical Consciousness as a Framework for Health Equity–Focused Peer Learning
Source: MedEdPORTAL. 2021 Apr 28;17:11145. doi: 10.15766/mep_2374-8265.11145 (PMC8079426; doi:10.15766/mep_2374-8265.11145)
Supplement: Supplementary file 1 — Workshop 1 Presentation.pptxWorkshop 1 Student Handout.docxWorkshop 2 Presentation.pptxWorkshop 2 Student Handout.docxWorkshop 3 Presentation.pptxWorkshop 3 Student Handout.docxWorkshop 4 Presentation.pptxWorkshop 5 Presentation.pptxFacilitator Orientation.pptxWorkshop 1 Facilitator Guide.docxWorkshop 2 Facilitator Guide.docxWorkshop 3 Facilitator Guide.docxWorkshop 4 Facilitator Guide.docxWorkshop 5 Facilitator Guide.docxEvaluation Tools.docx [file mep_2374-8265.11145-s001.zip › A. Workshop 1 Presentation.pptx]

## Slide 1
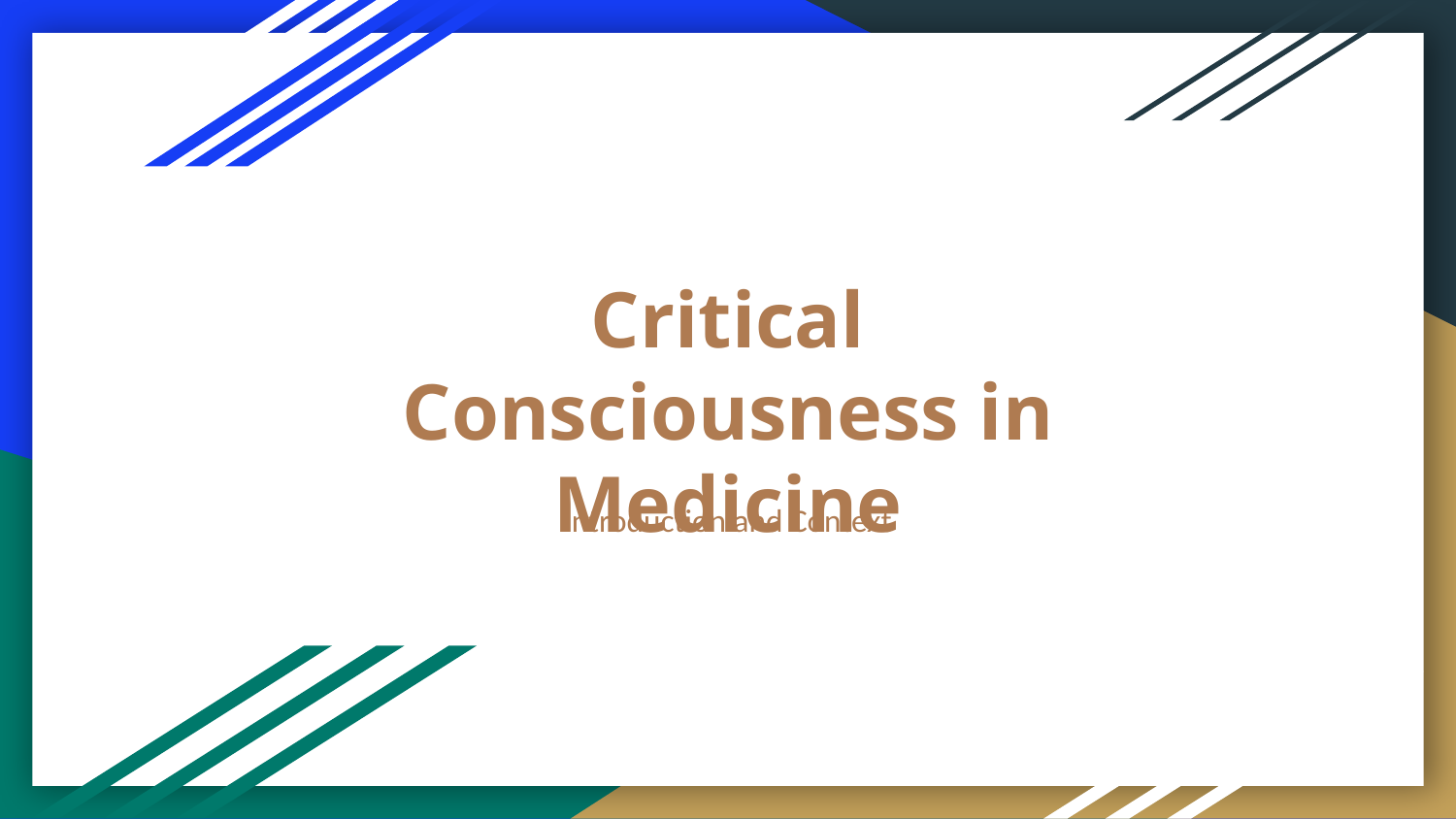

# Critical Consciousness in Medicine
Introduction and Context

## Slide 2
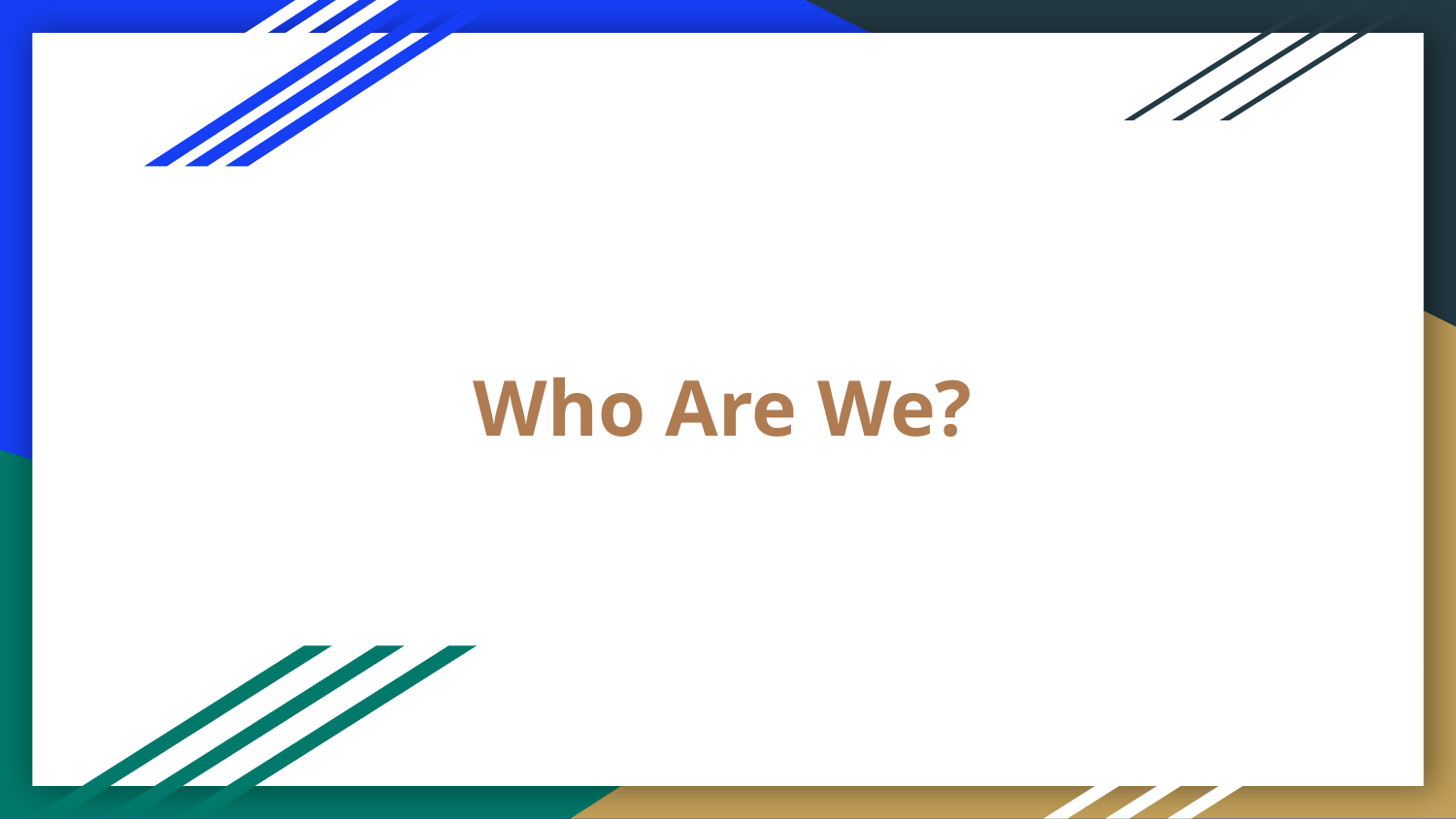

# Who Are We?

## Slide 3
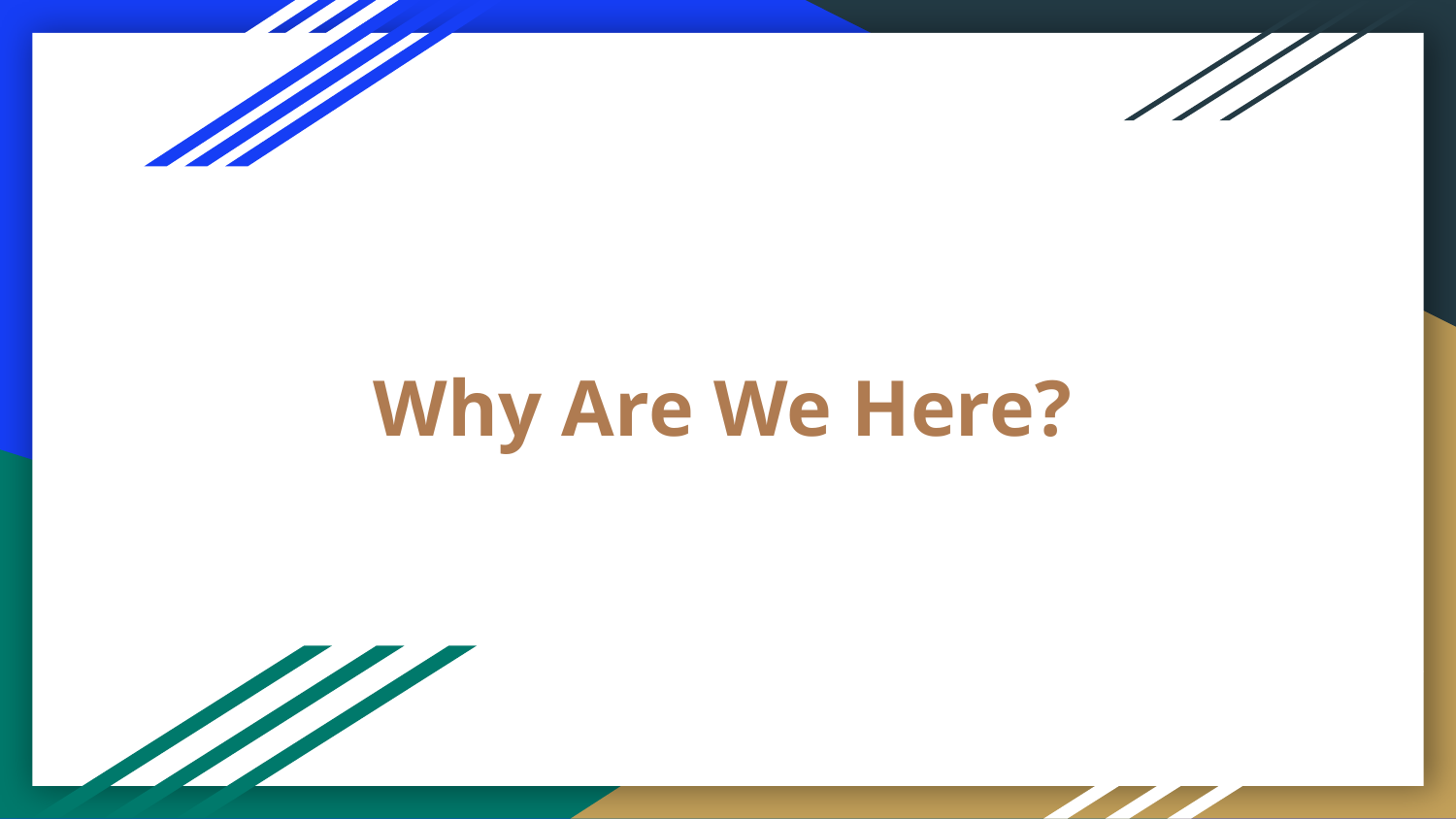

# Why Are We Here?

## Slide 4
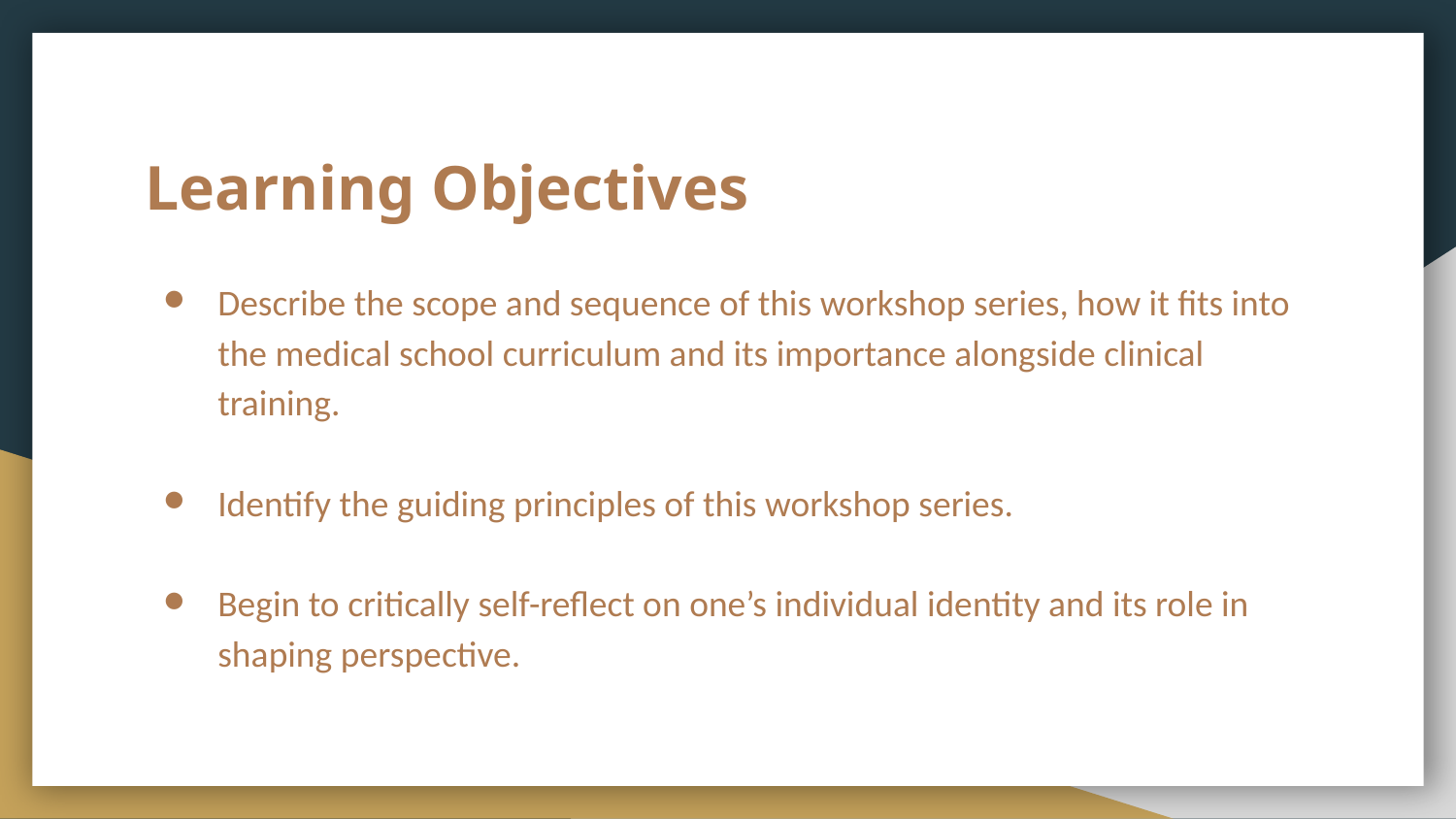

# Learning Objectives
Describe the scope and sequence of this workshop series, how it fits into the medical school curriculum and its importance alongside clinical training.
Identify the guiding principles of this workshop series.
Begin to critically self-reflect on one’s individual identity and its role in shaping perspective.

## Slide 5
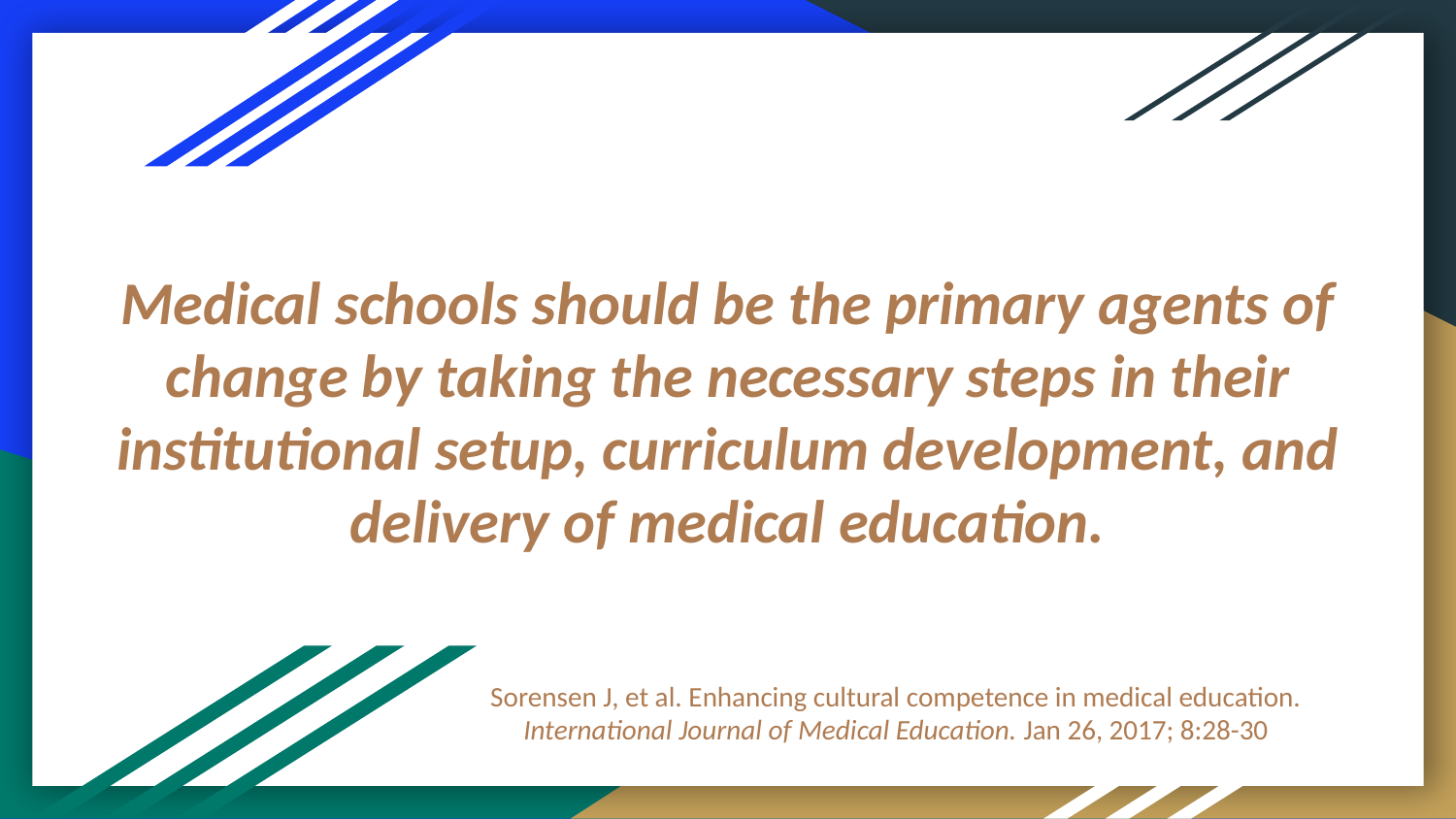

# Medical schools should be the primary agents of change by taking the necessary steps in their institutional setup, curriculum development, and delivery of medical education.
Sorensen J, et al. Enhancing cultural competence in medical education. International Journal of Medical Education. Jan 26, 2017; 8:28-30

## Slide 6
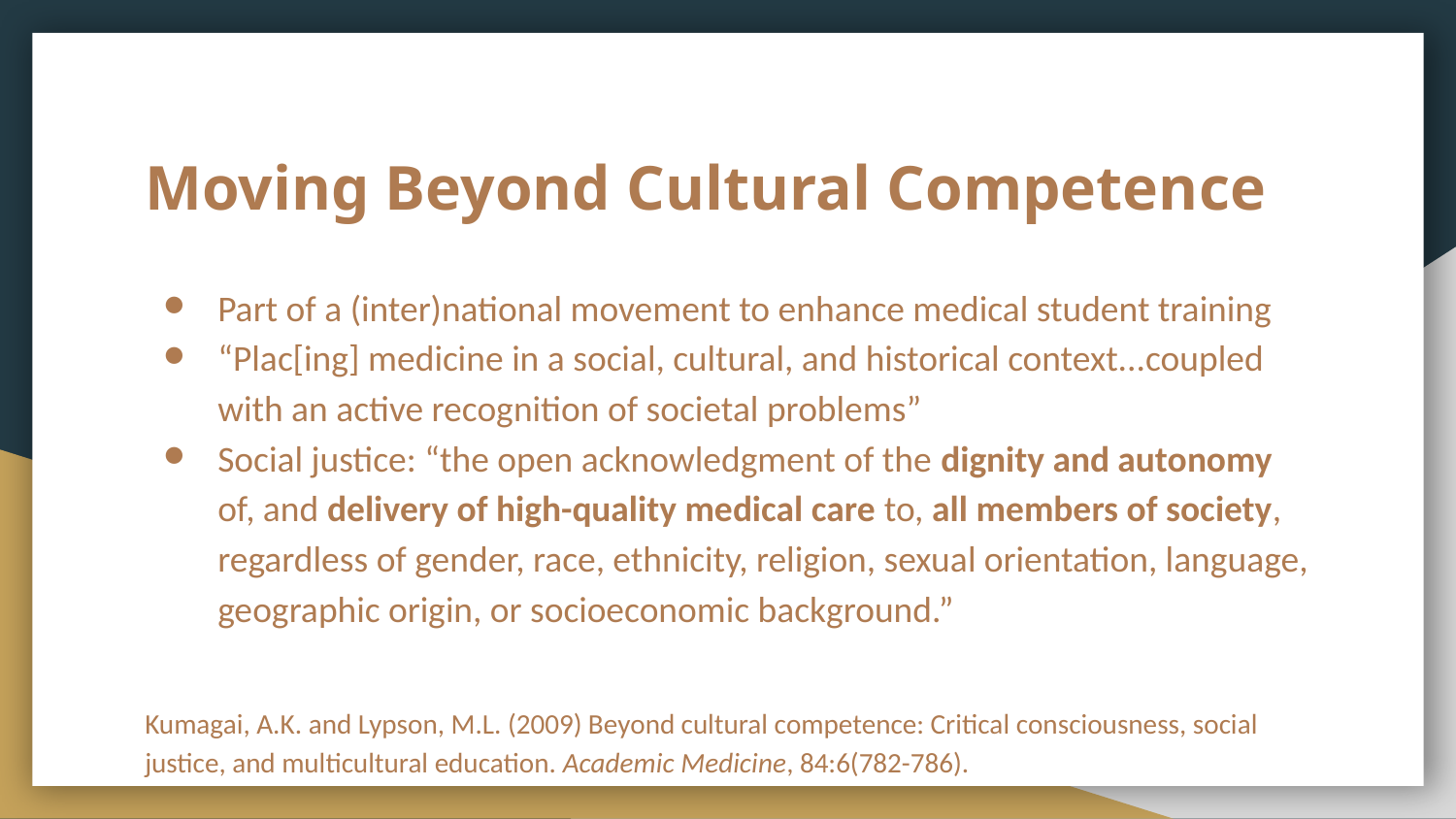

# Moving Beyond Cultural Competence
Part of a (inter)national movement to enhance medical student training
“Plac[ing] medicine in a social, cultural, and historical context...coupled with an active recognition of societal problems”
Social justice: “the open acknowledgment of the dignity and autonomy of, and delivery of high-quality medical care to, all members of society, regardless of gender, race, ethnicity, religion, sexual orientation, language, geographic origin, or socioeconomic background.”
Kumagai, A.K. and Lypson, M.L. (2009) Beyond cultural competence: Critical consciousness, social justice, and multicultural education. Academic Medicine, 84:6(782-786).

## Slide 7
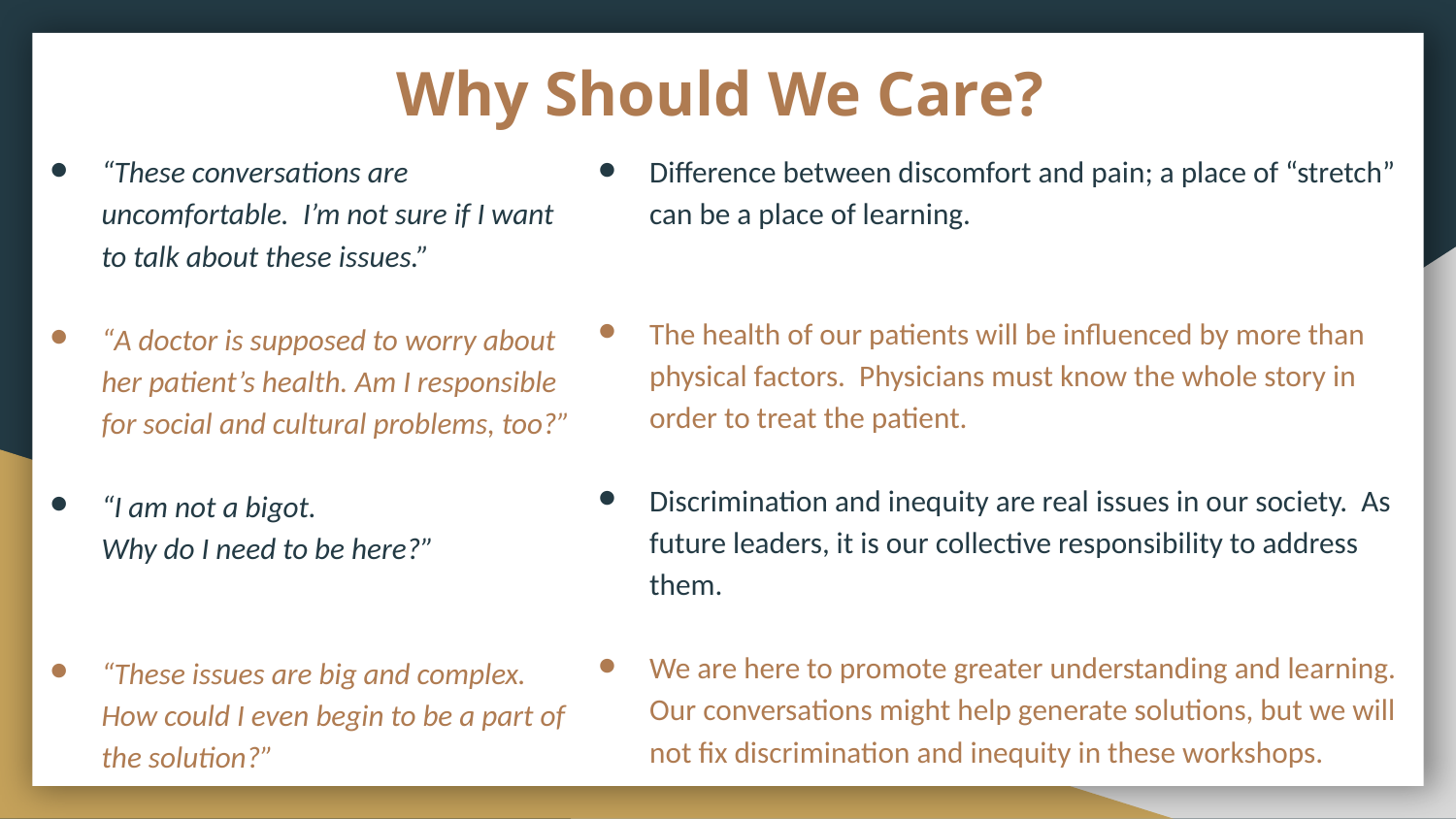

# Why Should We Care?
“These conversations are uncomfortable. I’m not sure if I want to talk about these issues.”
“A doctor is supposed to worry about her patient’s health. Am I responsible for social and cultural problems, too?”
“I am not a bigot.
Why do I need to be here?”
“These issues are big and complex. How could I even begin to be a part of the solution?”
Difference between discomfort and pain; a place of “stretch” can be a place of learning.
The health of our patients will be influenced by more than physical factors. Physicians must know the whole story in order to treat the patient.
Discrimination and inequity are real issues in our society. As future leaders, it is our collective responsibility to address them.
We are here to promote greater understanding and learning. Our conversations might help generate solutions, but we will not fix discrimination and inequity in these workshops.

## Slide 8
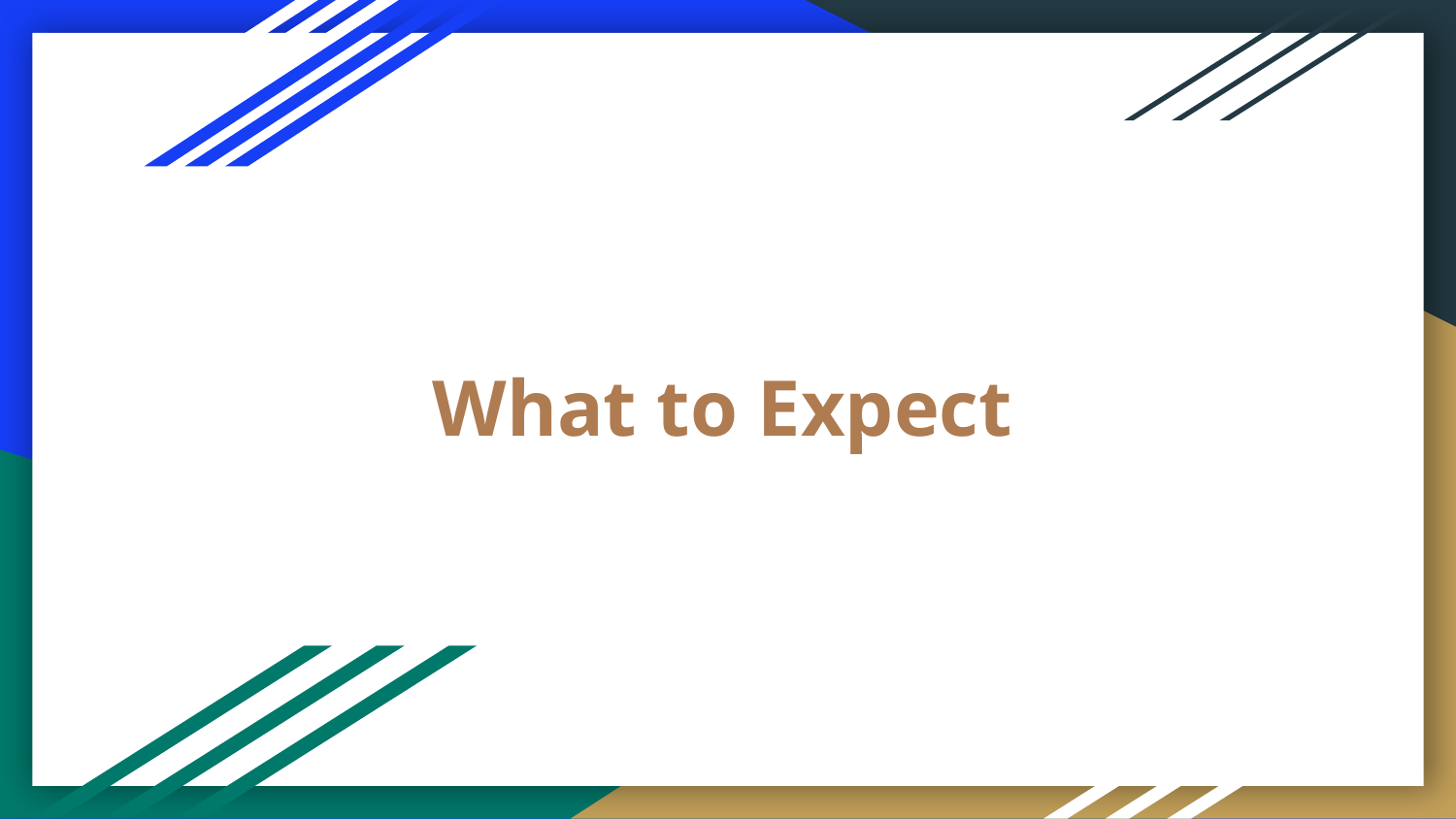

# What to Expect

## Slide 9
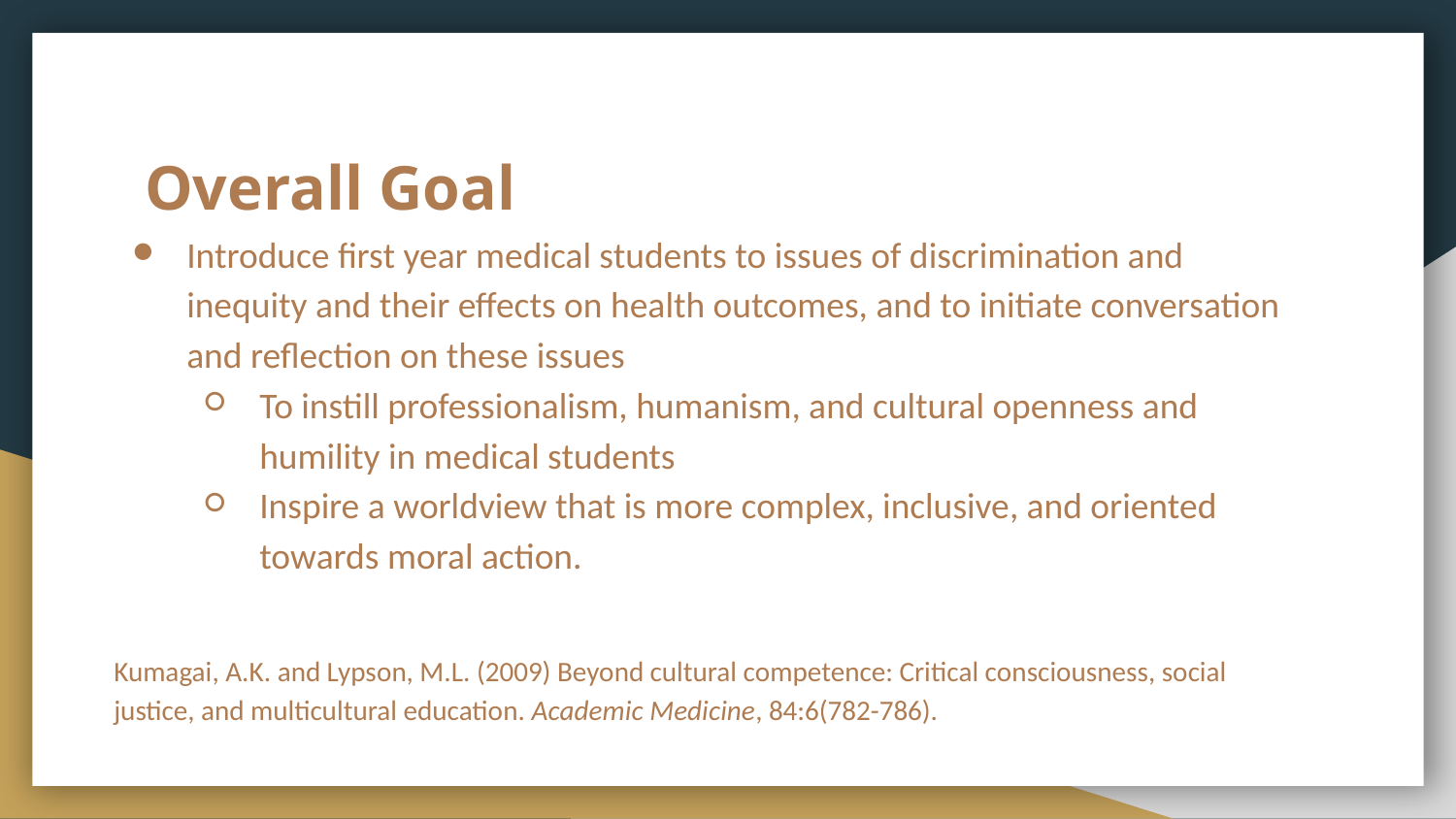

# Overall Goal
Introduce first year medical students to issues of discrimination and inequity and their effects on health outcomes, and to initiate conversation and reflection on these issues
To instill professionalism, humanism, and cultural openness and humility in medical students
Inspire a worldview that is more complex, inclusive, and oriented towards moral action.
Kumagai, A.K. and Lypson, M.L. (2009) Beyond cultural competence: Critical consciousness, social justice, and multicultural education. Academic Medicine, 84:6(782-786).

## Slide 10
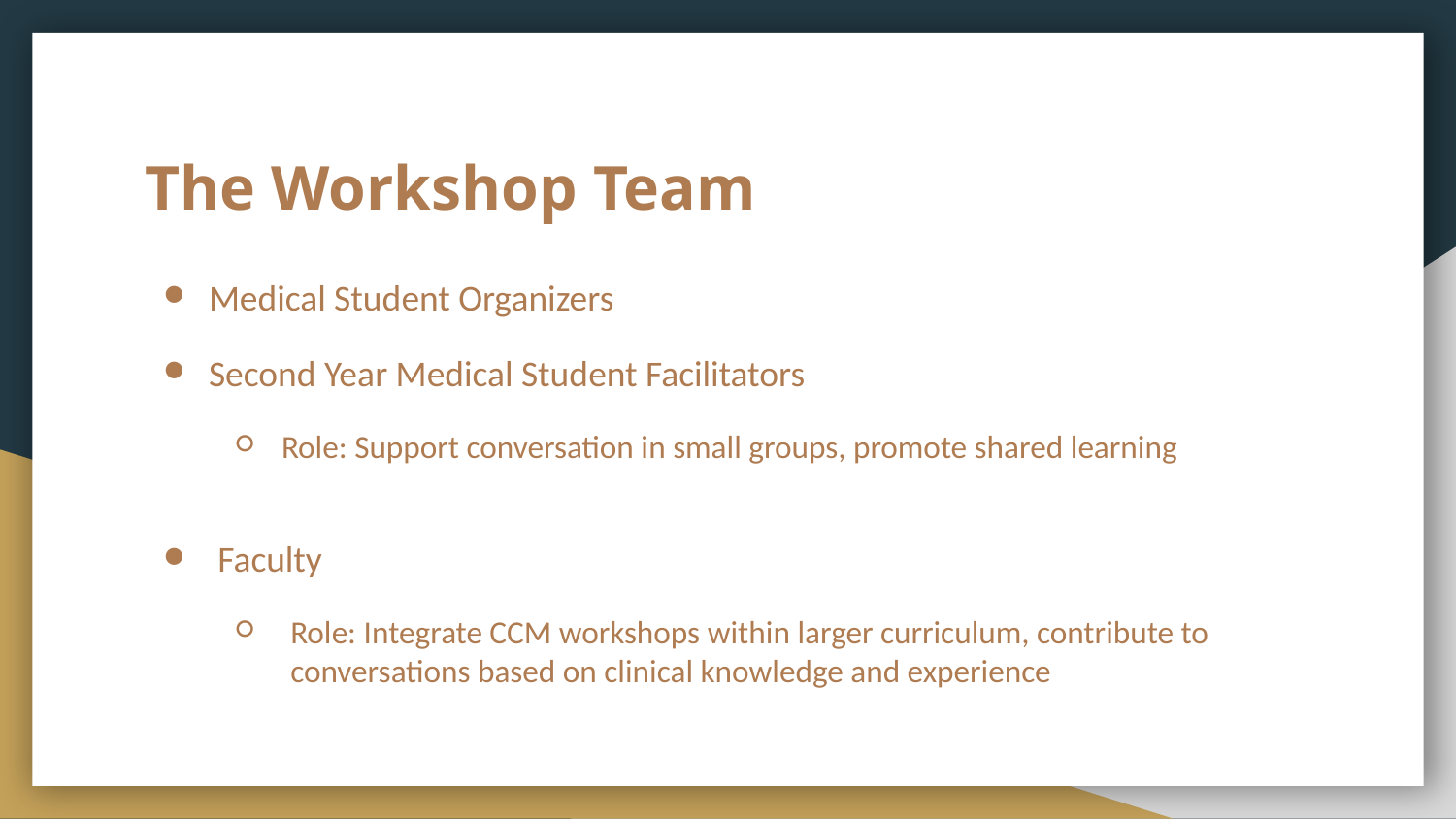

# The Workshop Team
Medical Student Organizers
Second Year Medical Student Facilitators
Role: Support conversation in small groups, promote shared learning
Faculty
Role: Integrate CCM workshops within larger curriculum, contribute to conversations based on clinical knowledge and experience

## Slide 11
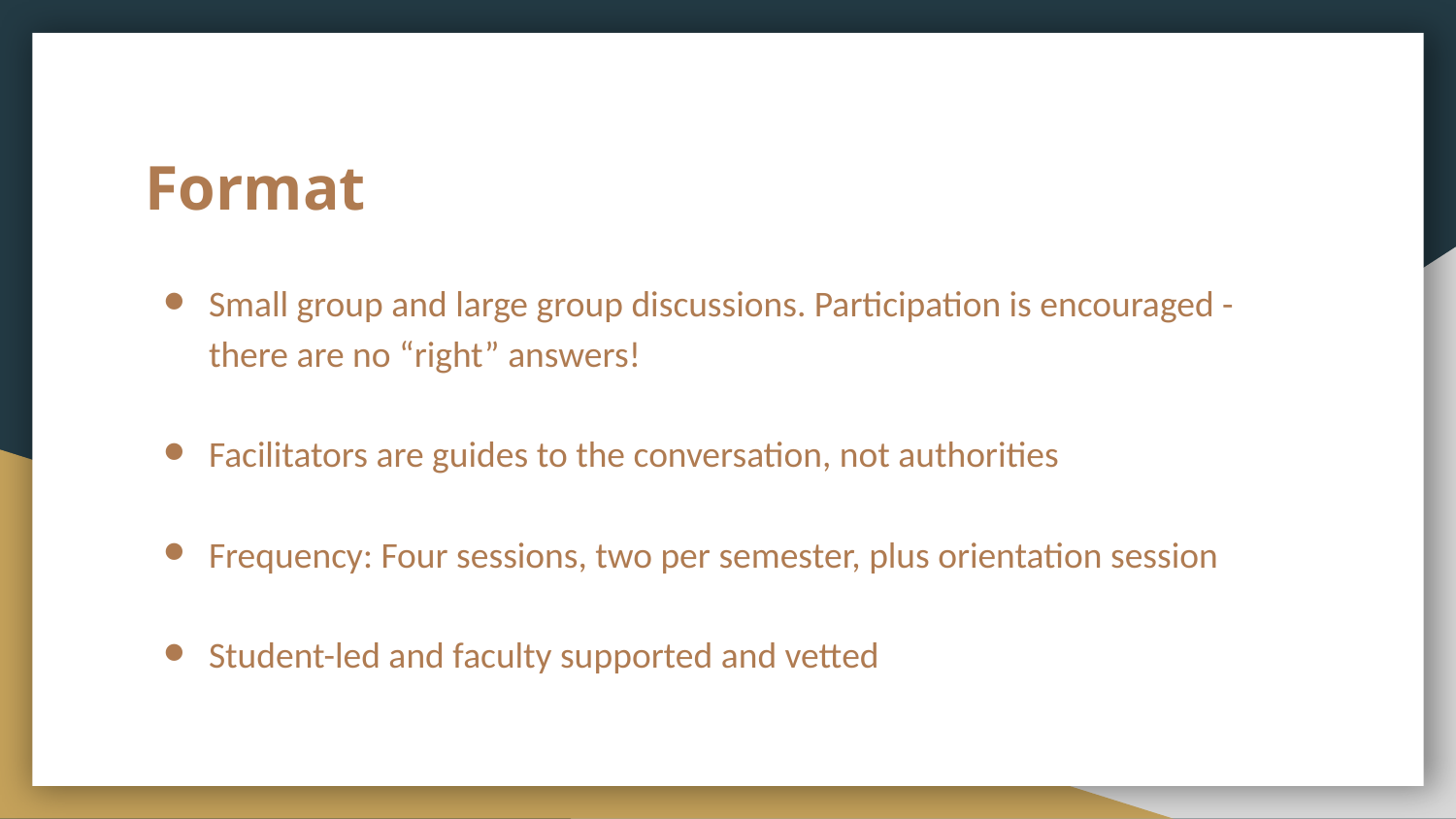

# Format
Small group and large group discussions. Participation is encouraged - there are no “right” answers!
Facilitators are guides to the conversation, not authorities
Frequency: Four sessions, two per semester, plus orientation session
Student-led and faculty supported and vetted

## Slide 12
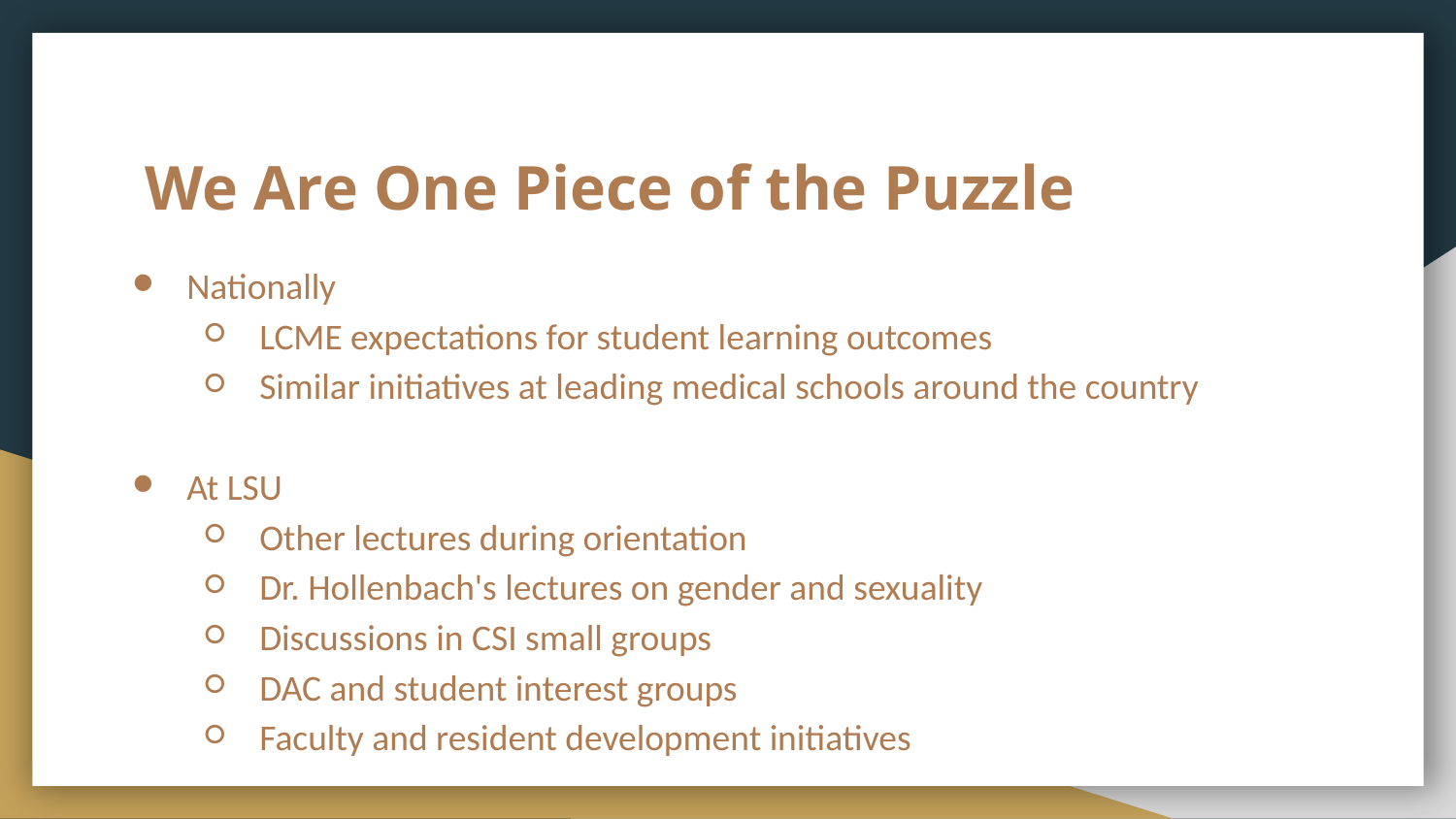

# We Are One Piece of the Puzzle
Nationally
LCME expectations for student learning outcomes
Similar initiatives at leading medical schools around the country
At LSU
Other lectures during orientation
Dr. Hollenbach's lectures on gender and sexuality
Discussions in CSI small groups
DAC and student interest groups
Faculty and resident development initiatives

## Slide 13
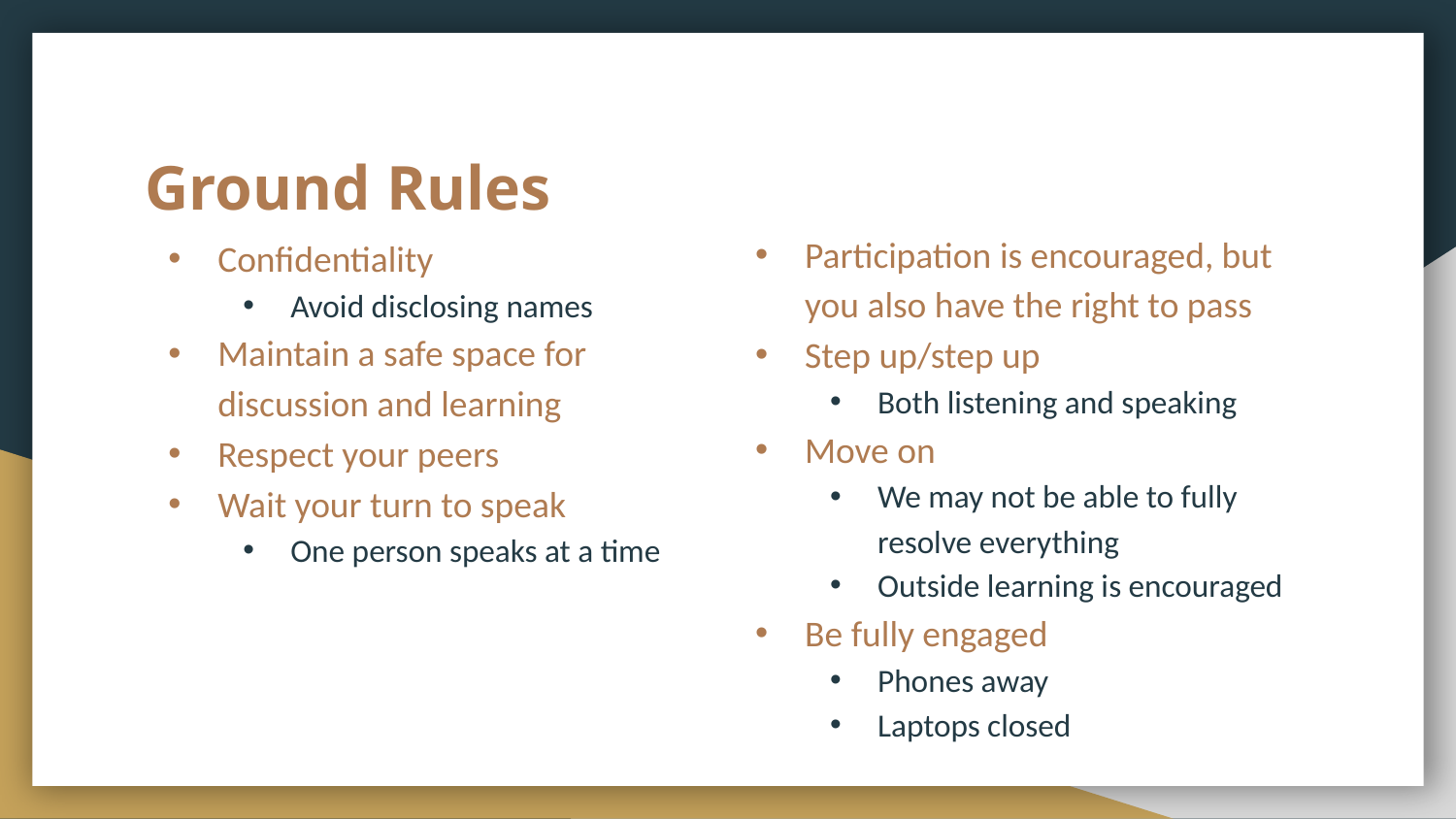

# Ground Rules
Participation is encouraged, but you also have the right to pass
Step up/step up
Both listening and speaking
Move on
We may not be able to fully resolve everything
Outside learning is encouraged
Be fully engaged
Phones away
Laptops closed
Confidentiality
Avoid disclosing names
Maintain a safe space for discussion and learning
Respect your peers
Wait your turn to speak
One person speaks at a time

## Slide 14
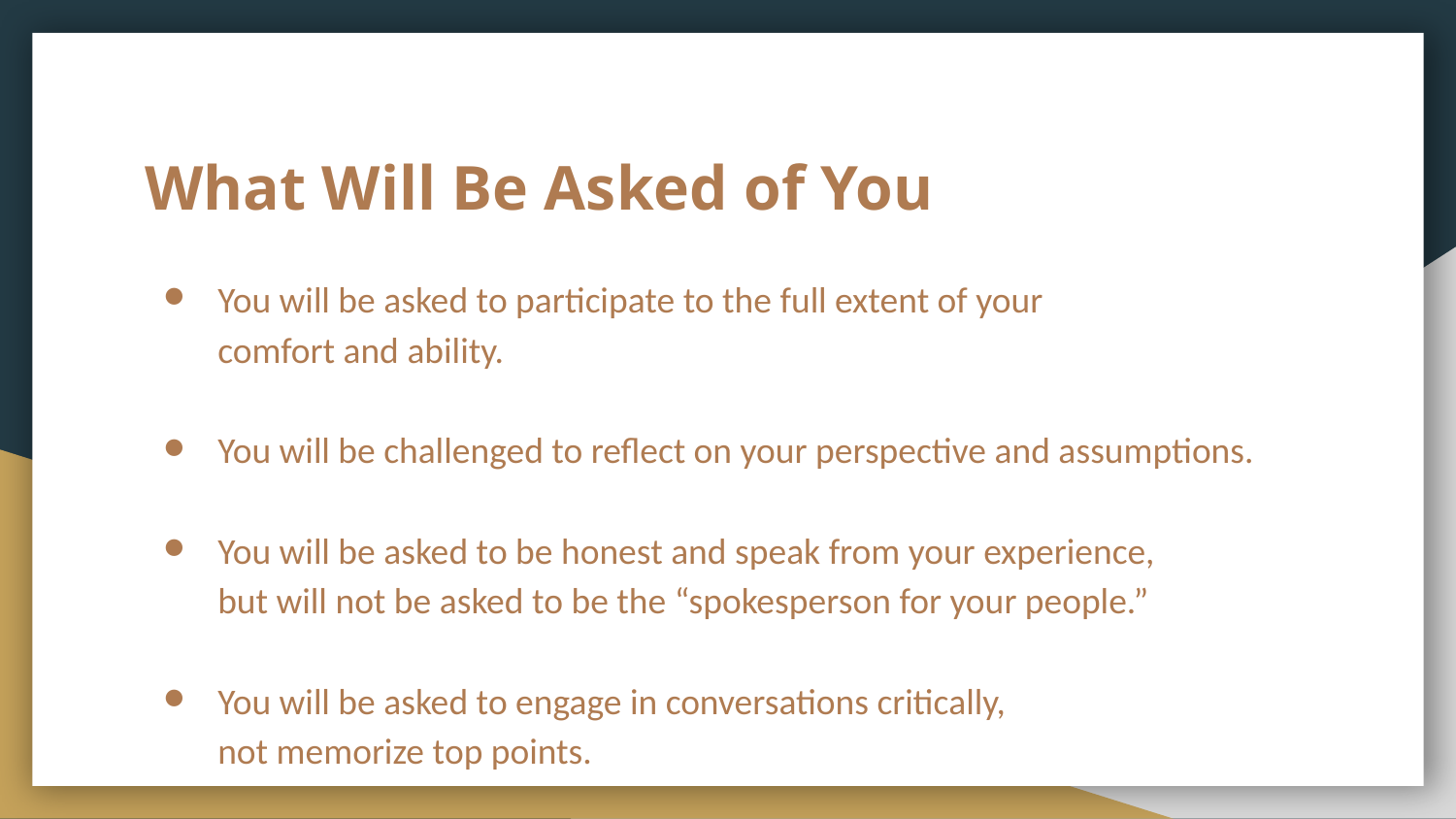

# What Will Be Asked of You
You will be asked to participate to the full extent of your
comfort and ability.
You will be challenged to reflect on your perspective and assumptions.
You will be asked to be honest and speak from your experience,
but will not be asked to be the “spokesperson for your people.”
You will be asked to engage in conversations critically,
not memorize top points.

## Slide 15
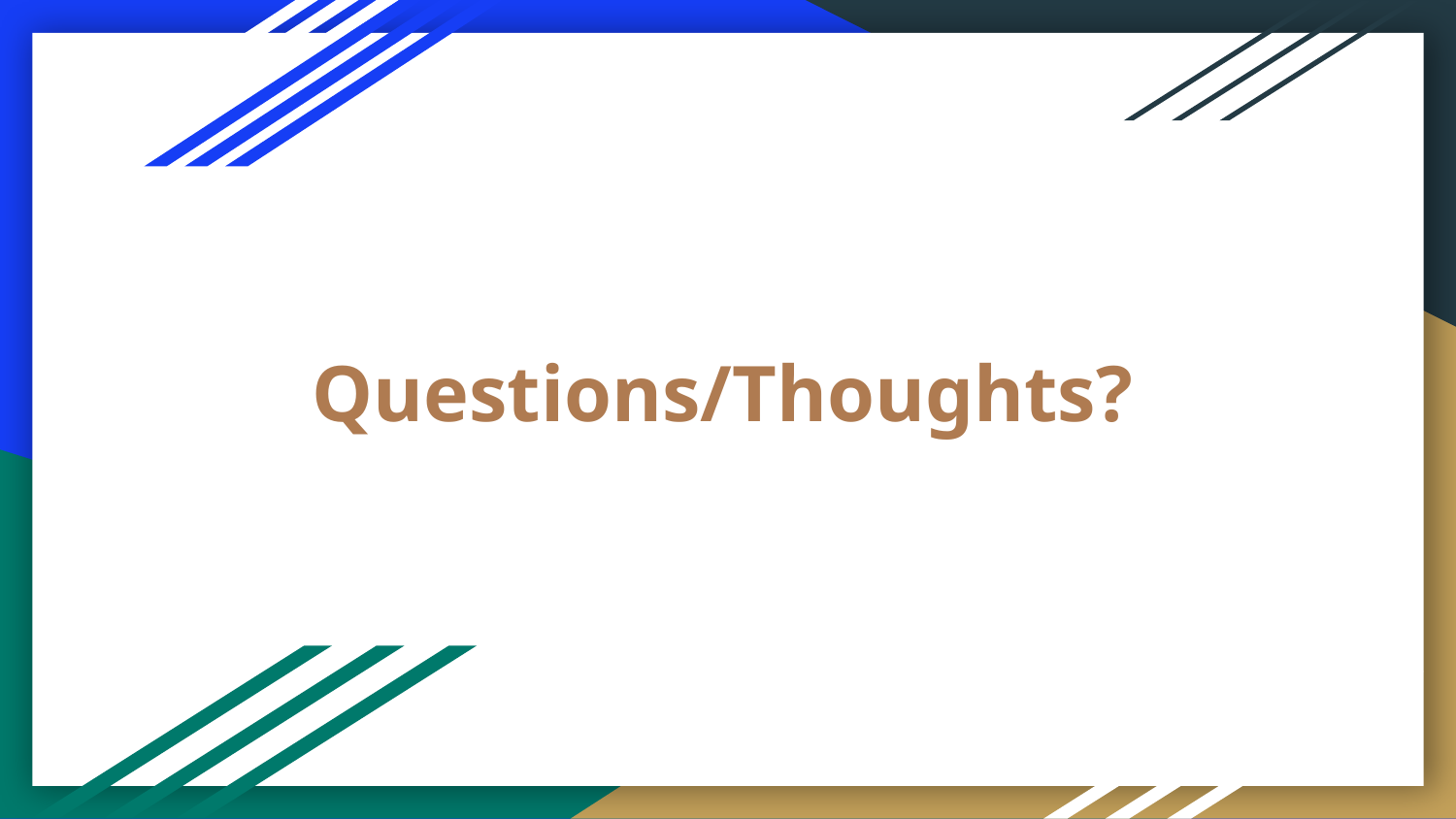

# Questions/Thoughts?

## Slide 16
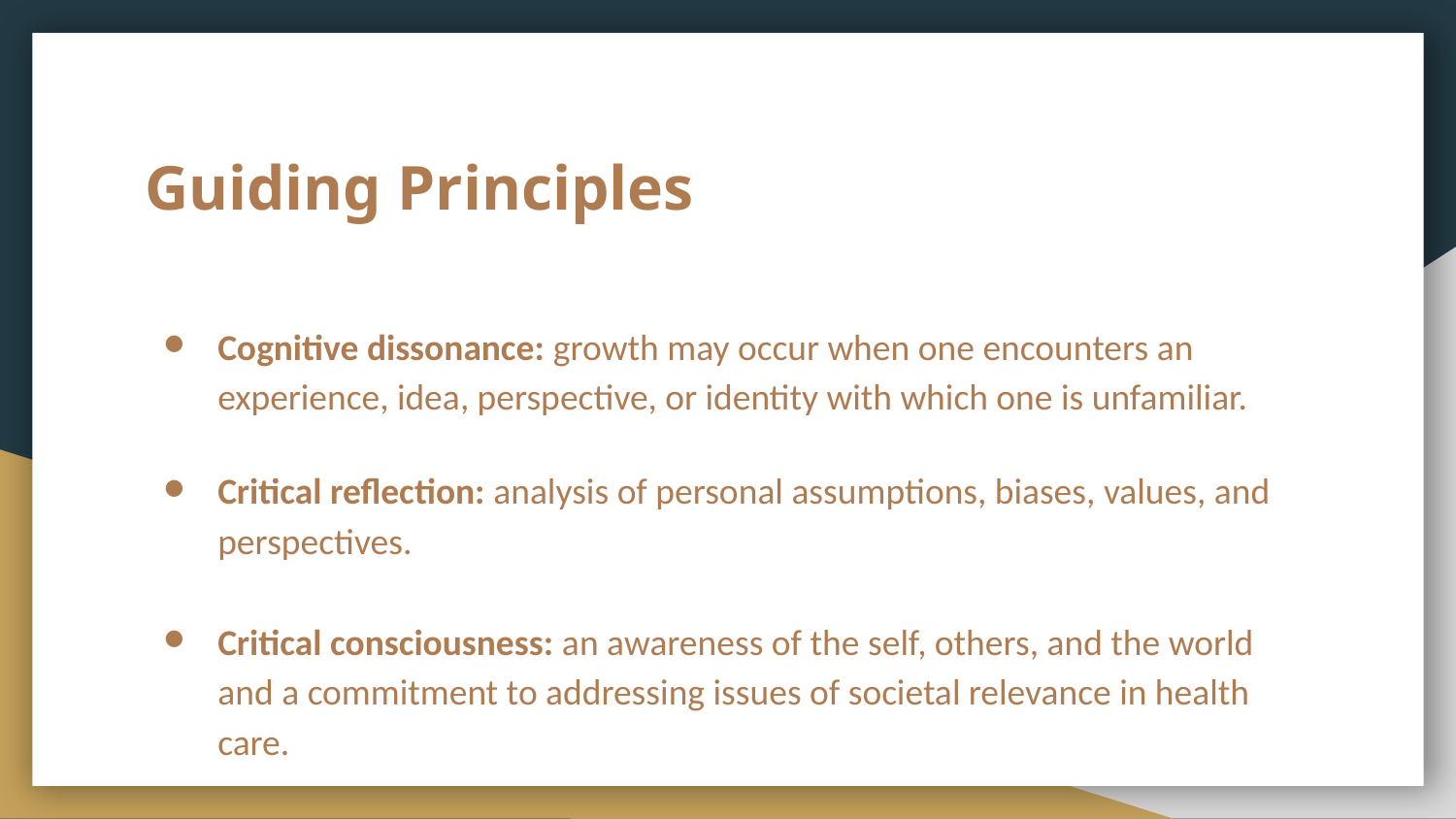

# Guiding Principles
Cognitive dissonance: growth may occur when one encounters an experience, idea, perspective, or identity with which one is unfamiliar.
Critical reflection: analysis of personal assumptions, biases, values, and perspectives.
Critical consciousness: an awareness of the self, others, and the world and a commitment to addressing issues of societal relevance in health care.
Kumagai, A.K. and Lypson, M.L. (2009) Beyond cultural competence: Critical consciousness, social justice, and multicultural education. Academic Medicine, 84:6(782-786).

## Slide 17
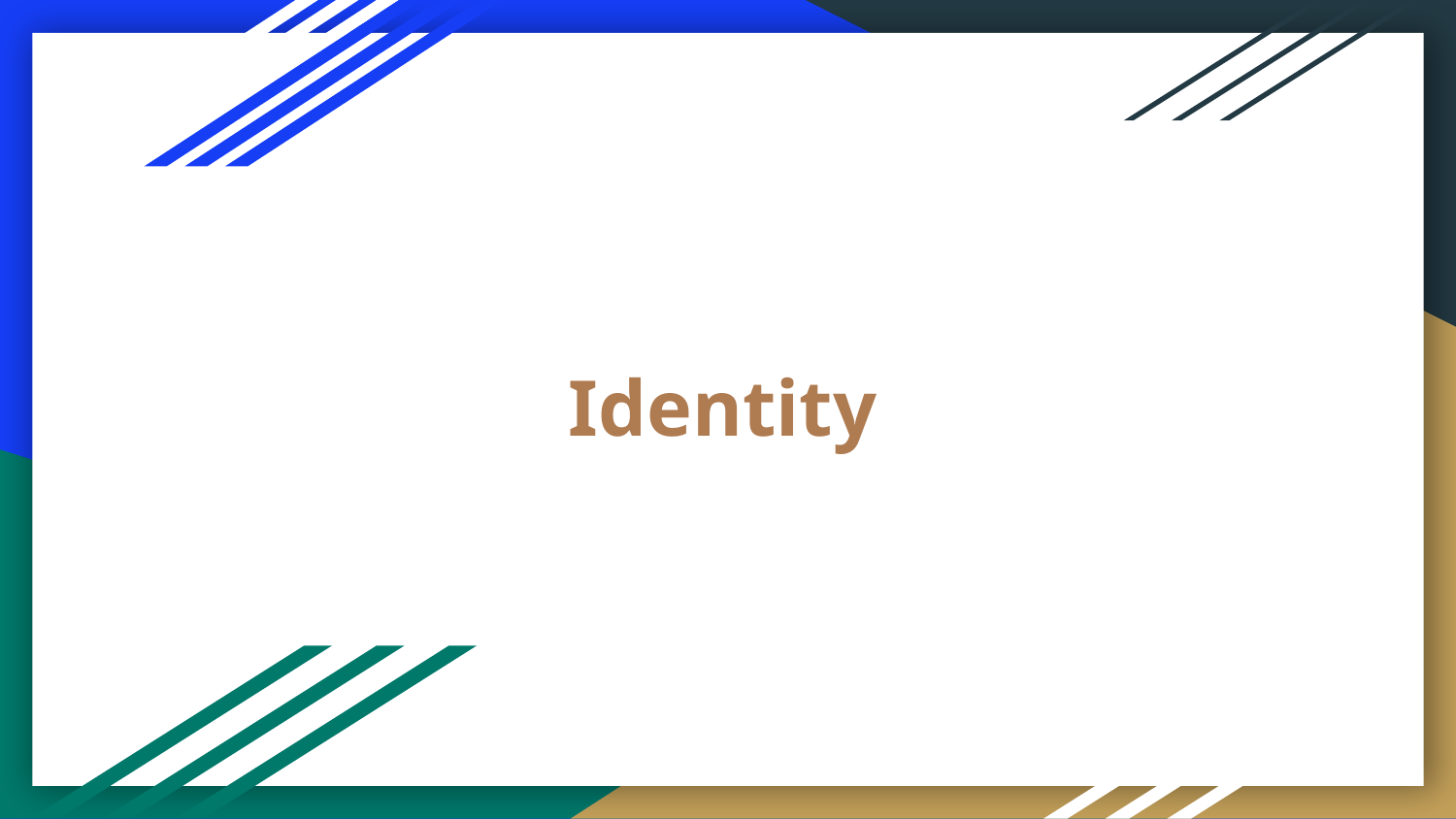

# Identity

## Slide 18
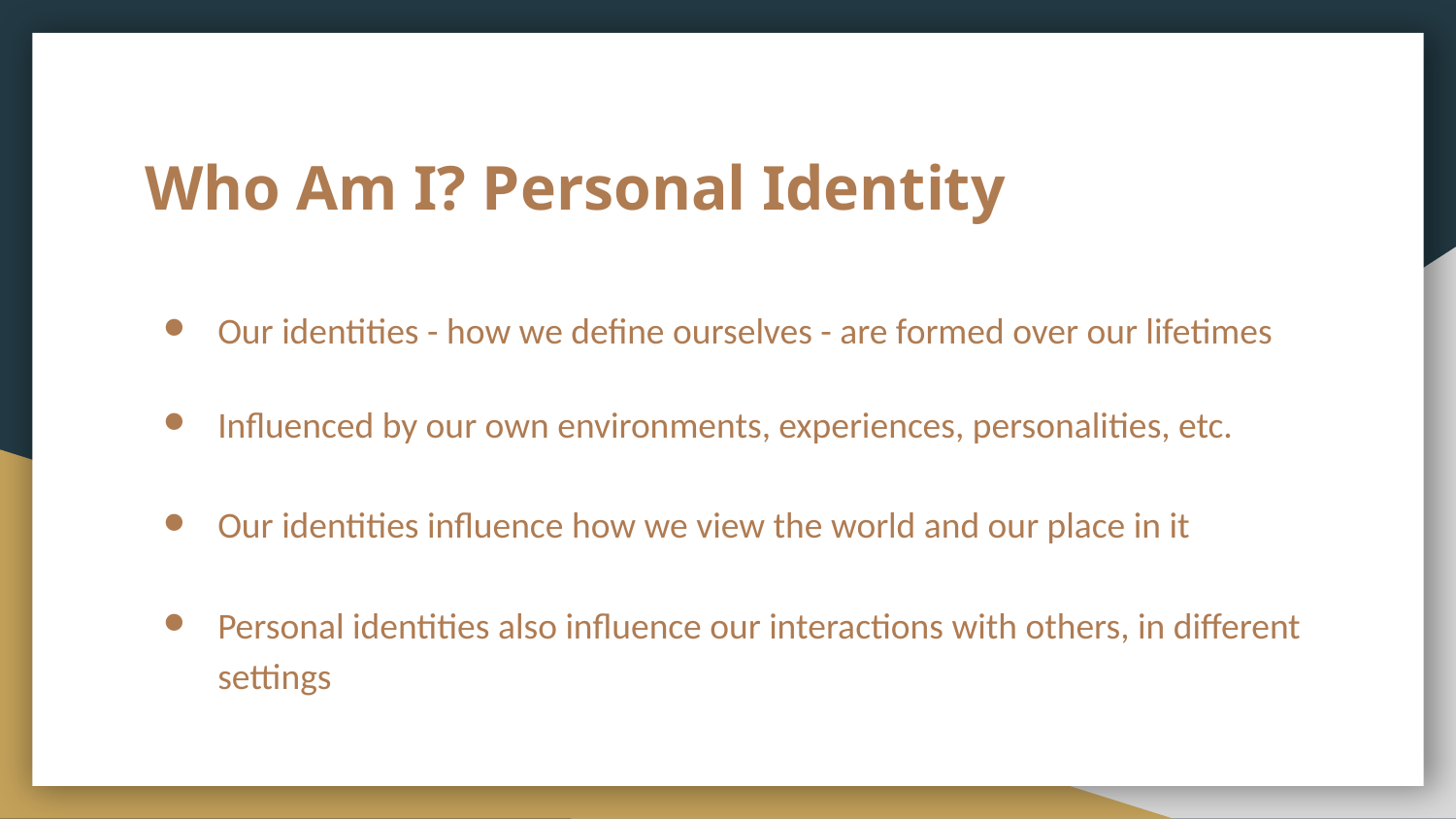

# Who Am I? Personal Identity
Our identities - how we define ourselves - are formed over our lifetimes
Influenced by our own environments, experiences, personalities, etc.
Our identities influence how we view the world and our place in it
Personal identities also influence our interactions with others, in different settings

## Slide 19
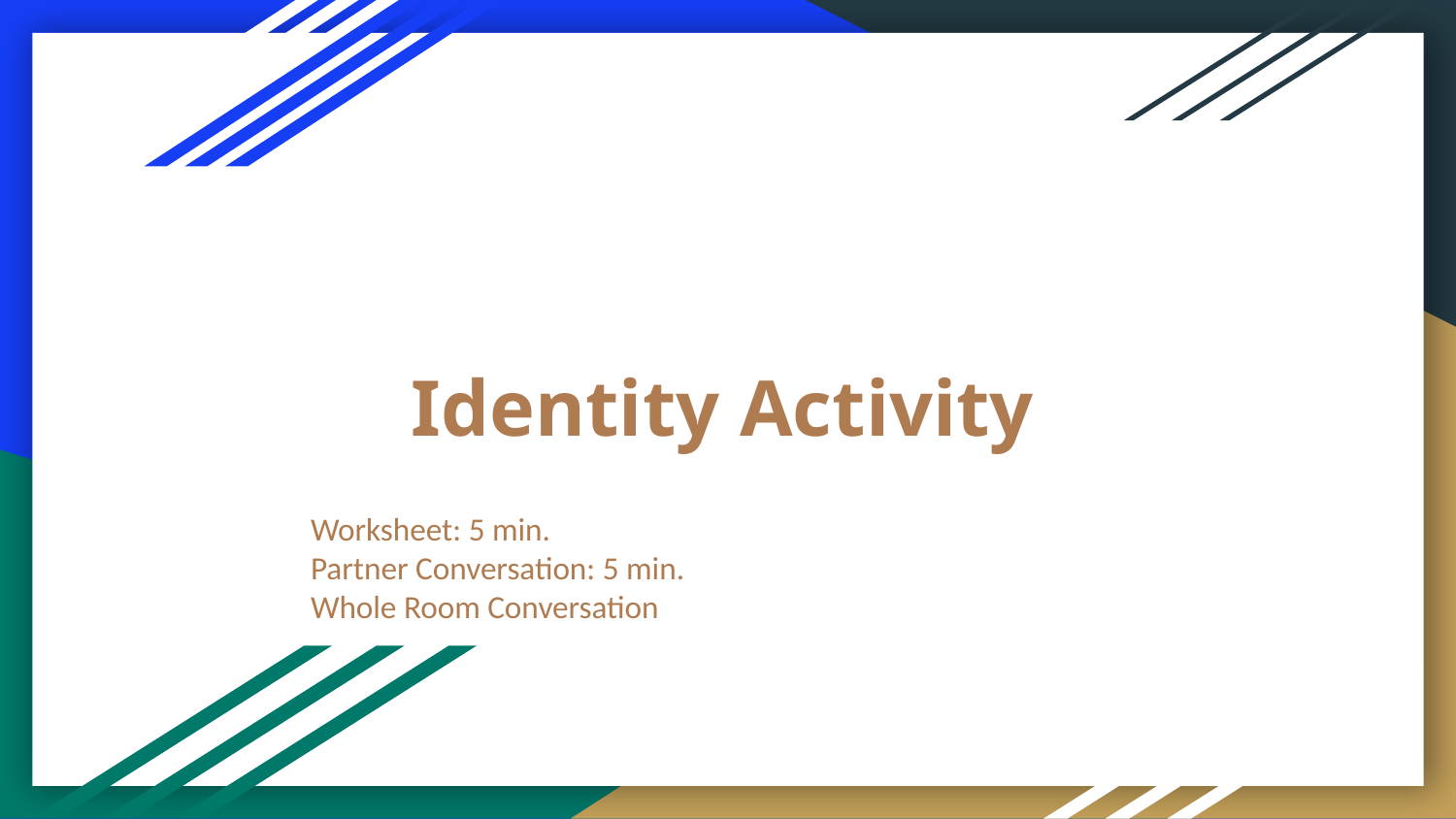

# Identity Activity
Worksheet: 5 min.
Partner Conversation: 5 min.
Whole Room Conversation

## Slide 20
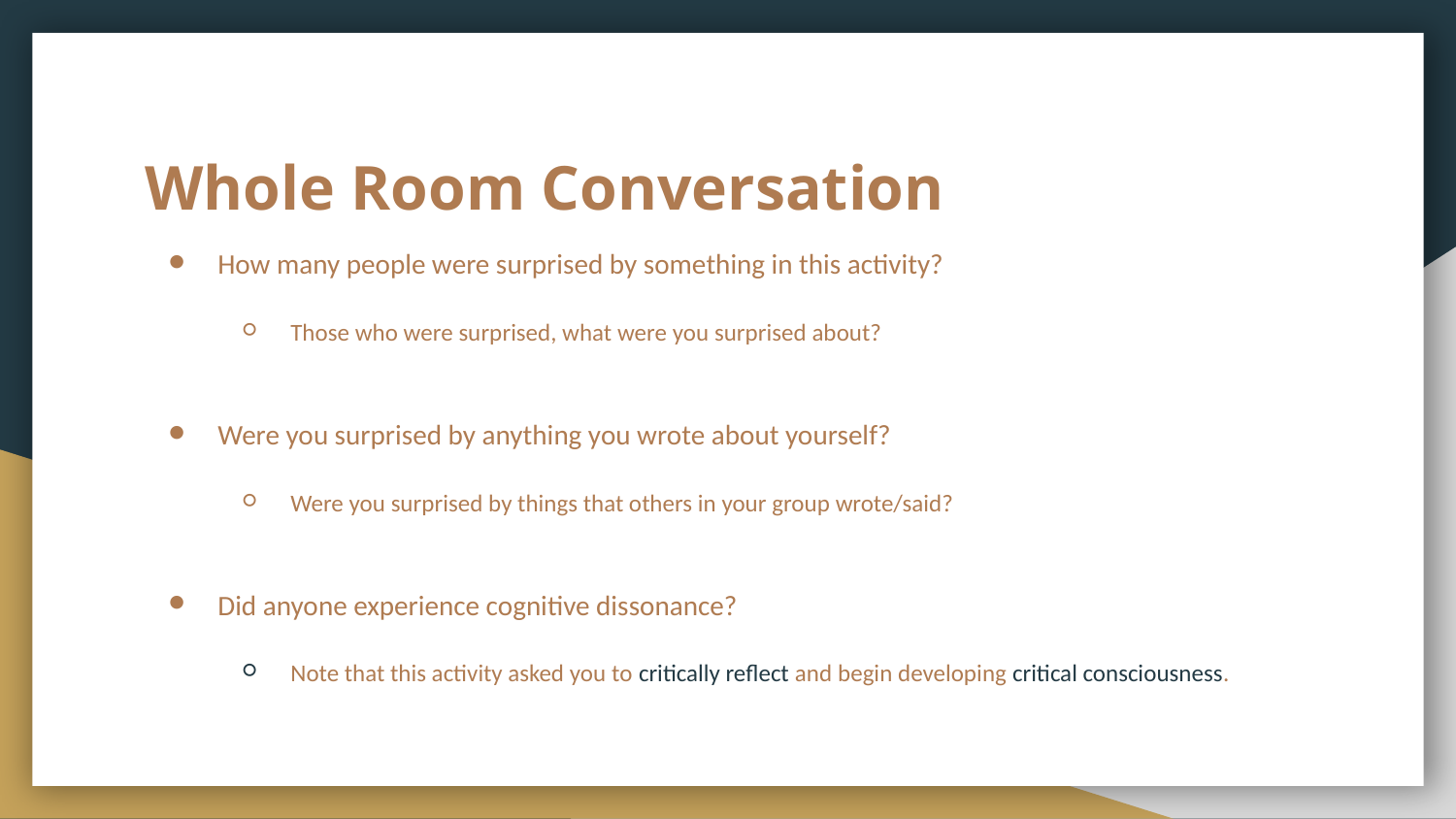

# Whole Room Conversation
How many people were surprised by something in this activity?
Those who were surprised, what were you surprised about?
Were you surprised by anything you wrote about yourself?
Were you surprised by things that others in your group wrote/said?
Did anyone experience cognitive dissonance?
Note that this activity asked you to critically reflect and begin developing critical consciousness.

## Slide 21
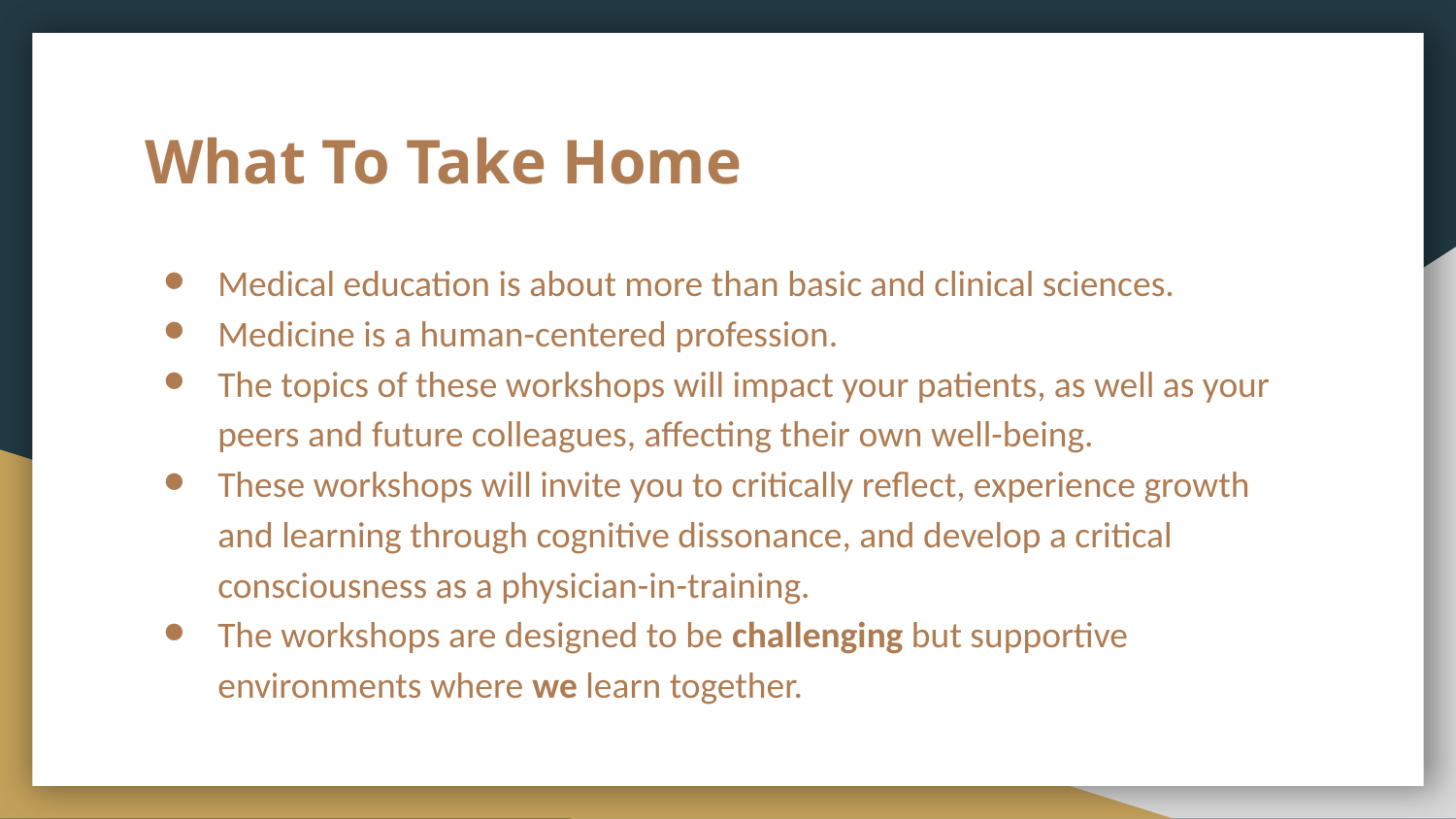

# What To Take Home
Medical education is about more than basic and clinical sciences.
Medicine is a human-centered profession.
The topics of these workshops will impact your patients, as well as your peers and future colleagues, affecting their own well-being.
These workshops will invite you to critically reflect, experience growth and learning through cognitive dissonance, and develop a critical consciousness as a physician-in-training.
The workshops are designed to be challenging but supportive environments where we learn together.

## Slide 22
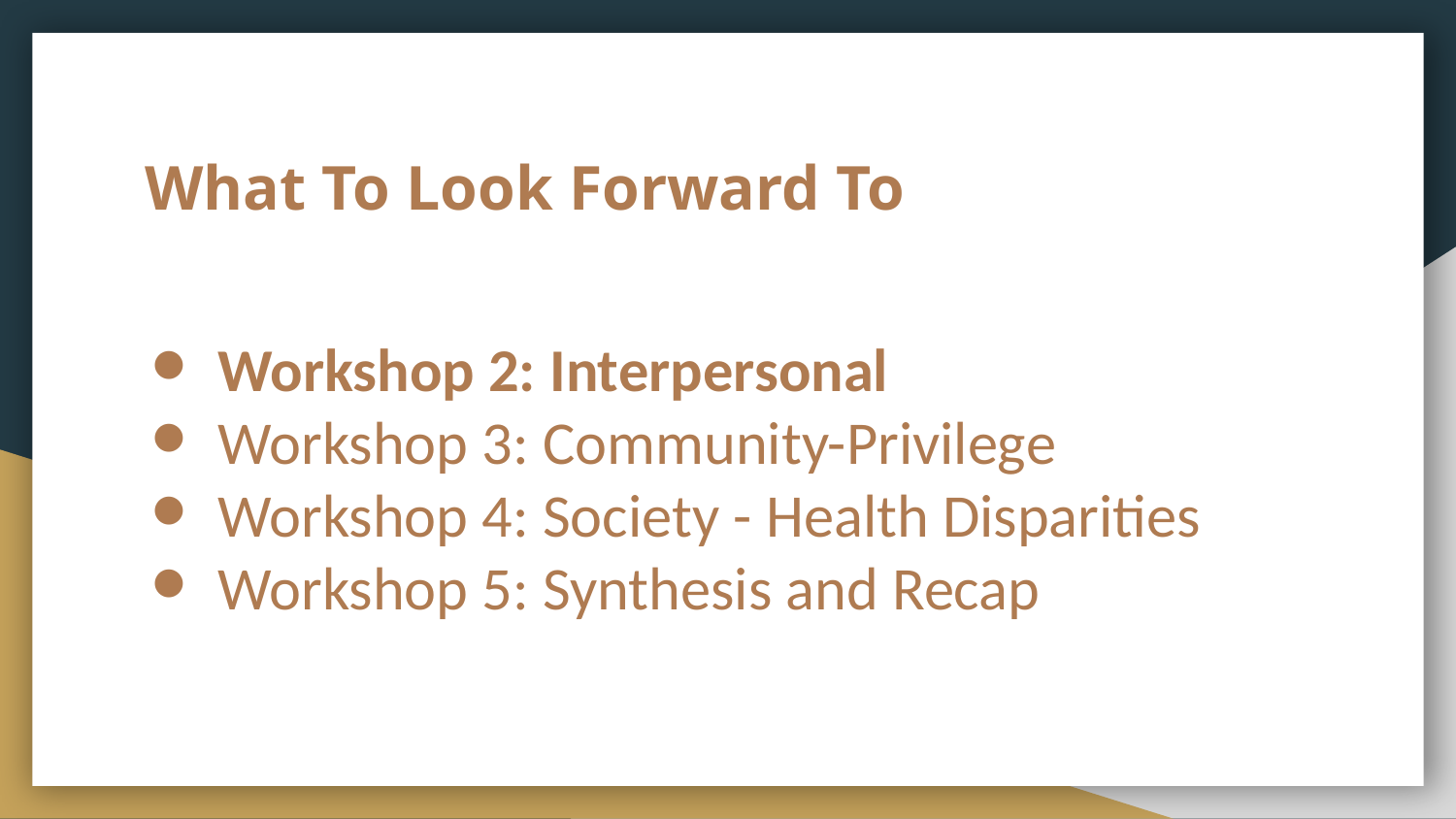

# What To Look Forward To
Workshop 2: Interpersonal
Workshop 3: Community-Privilege
Workshop 4: Society - Health Disparities
Workshop 5: Synthesis and Recap

## Slide 23
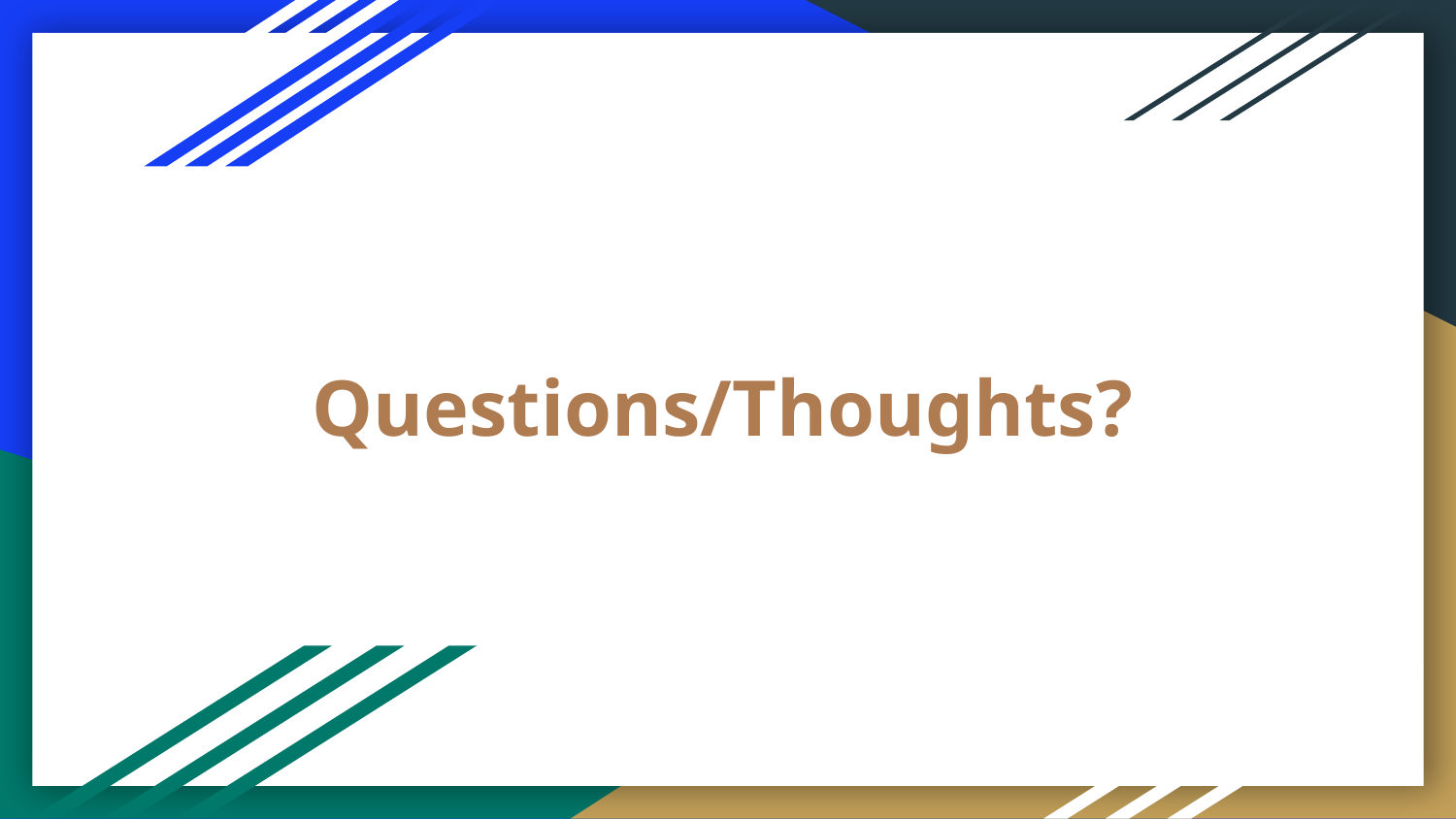

# Questions/Thoughts?
