# Supplementary material for: Critical Consciousness as a Framework for Health Equity–Focused Peer Learning
Source: MedEdPORTAL. 2021 Apr 28;17:11145. doi: 10.15766/mep_2374-8265.11145 (PMC8079426; doi:10.15766/mep_2374-8265.11145)
Supplement: Supplementary file 1 — Workshop 1 Presentation.pptxWorkshop 1 Student Handout.docxWorkshop 2 Presentation.pptxWorkshop 2 Student Handout.docxWorkshop 3 Presentation.pptxWorkshop 3 Student Handout.docxWorkshop 4 Presentation.pptxWorkshop 5 Presentation.pptxFacilitator Orientation.pptxWorkshop 1 Facilitator Guide.docxWorkshop 2 Facilitator Guide.docxWorkshop 3 Facilitator Guide.docxWorkshop 4 Facilitator Guide.docxWorkshop 5 Facilitator Guide.docxEvaluation Tools.docx [file mep_2374-8265.11145-s001.zip › B. Workshop 1 Student Handout.docx]

Critical Consciousness Workshop #1: Identity

Identity Charts Activity:

Inside the hand, write labels and descriptions you use to describe yourself. Outside of the outline, write labels and descriptions that reflect how you think others view you. In some cases, how the outside world sees us is the same as how we see ourselves, and in some cases, it is not. Therefore, words inside the outline and outside the outline may or may not overlap.


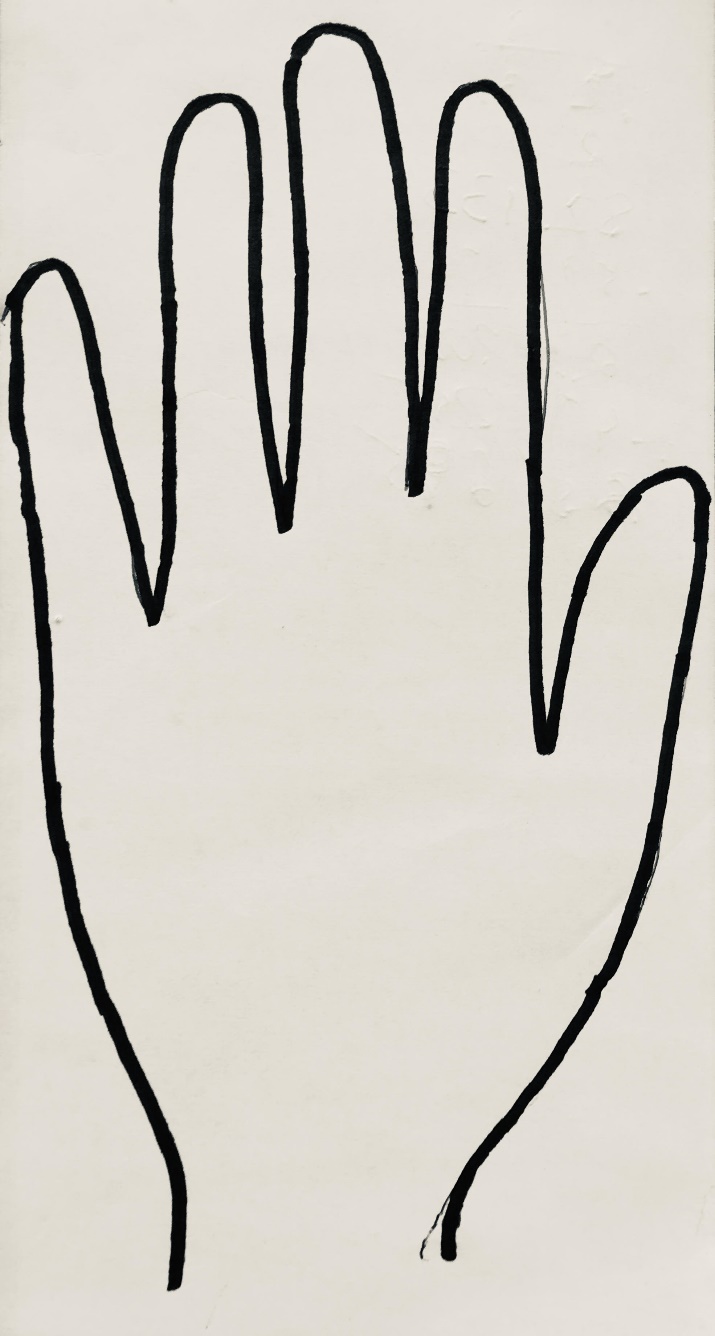


Image author owned.

**Notice the words inside and outside of your hand outline. Which ones are the same? Which ones are different?**

**Explain why some words might be the same, while others might be different.**

The source for this activity is: Facing History and Ourselves. Identity charts. Facing History and Ourselves web site. https://www.facinghistory.org/resource-library/teaching-strategies/identity-charts. Updated 2020. Accessed April 13, 2020.
