# Supplementary material for: Critical Consciousness as a Framework for Health Equity–Focused Peer Learning
Source: MedEdPORTAL. 2021 Apr 28;17:11145. doi: 10.15766/mep_2374-8265.11145 (PMC8079426; doi:10.15766/mep_2374-8265.11145)
Supplement: Supplementary file 1 — Workshop 1 Presentation.pptxWorkshop 1 Student Handout.docxWorkshop 2 Presentation.pptxWorkshop 2 Student Handout.docxWorkshop 3 Presentation.pptxWorkshop 3 Student Handout.docxWorkshop 4 Presentation.pptxWorkshop 5 Presentation.pptxFacilitator Orientation.pptxWorkshop 1 Facilitator Guide.docxWorkshop 2 Facilitator Guide.docxWorkshop 3 Facilitator Guide.docxWorkshop 4 Facilitator Guide.docxWorkshop 5 Facilitator Guide.docxEvaluation Tools.docx [file mep_2374-8265.11145-s001.zip › C. Workshop 2 Presentation.pptx]

## Slide 1
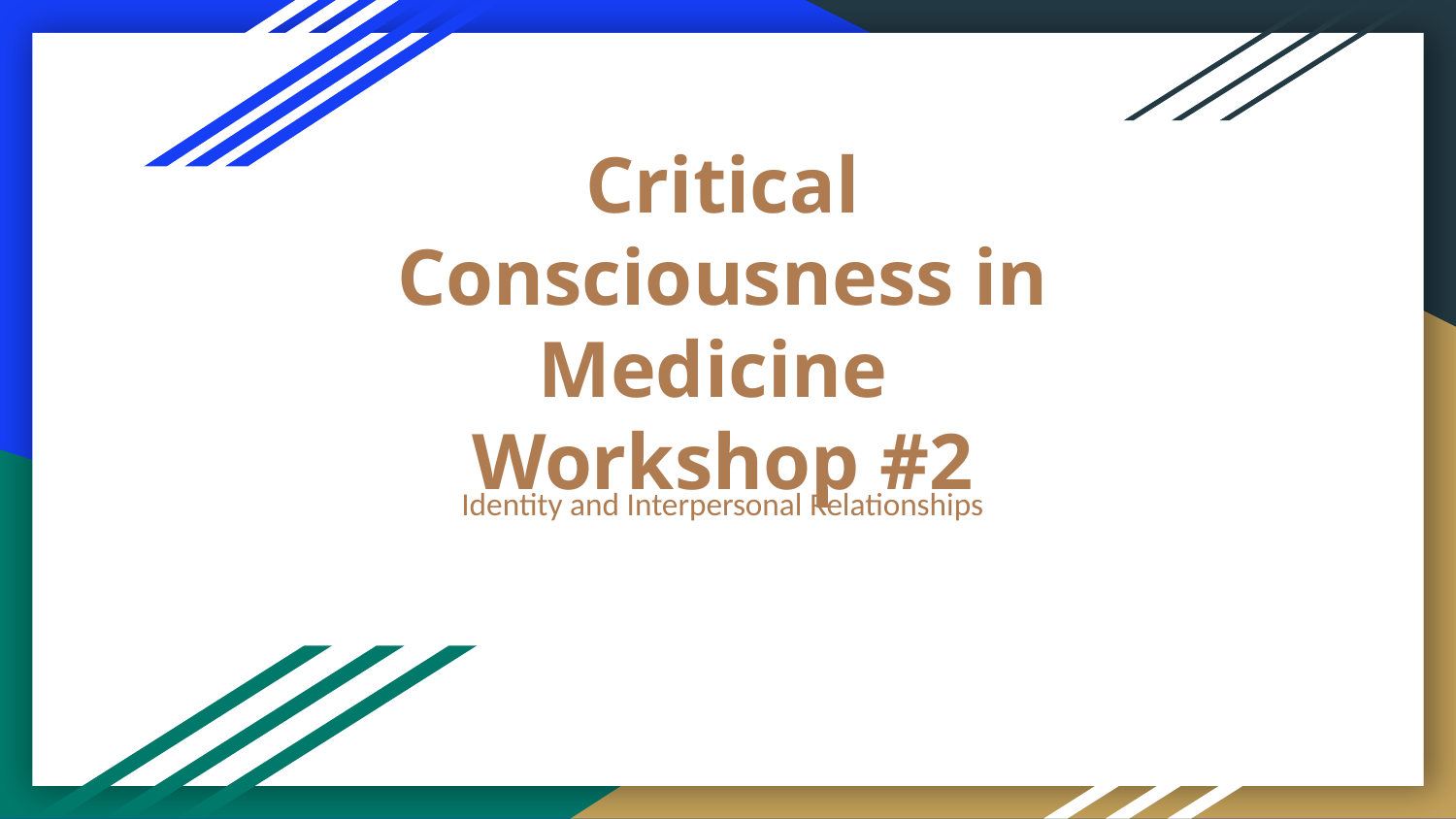

# Critical Consciousness in Medicine
Workshop #2
Identity and Interpersonal Relationships

## Slide 2
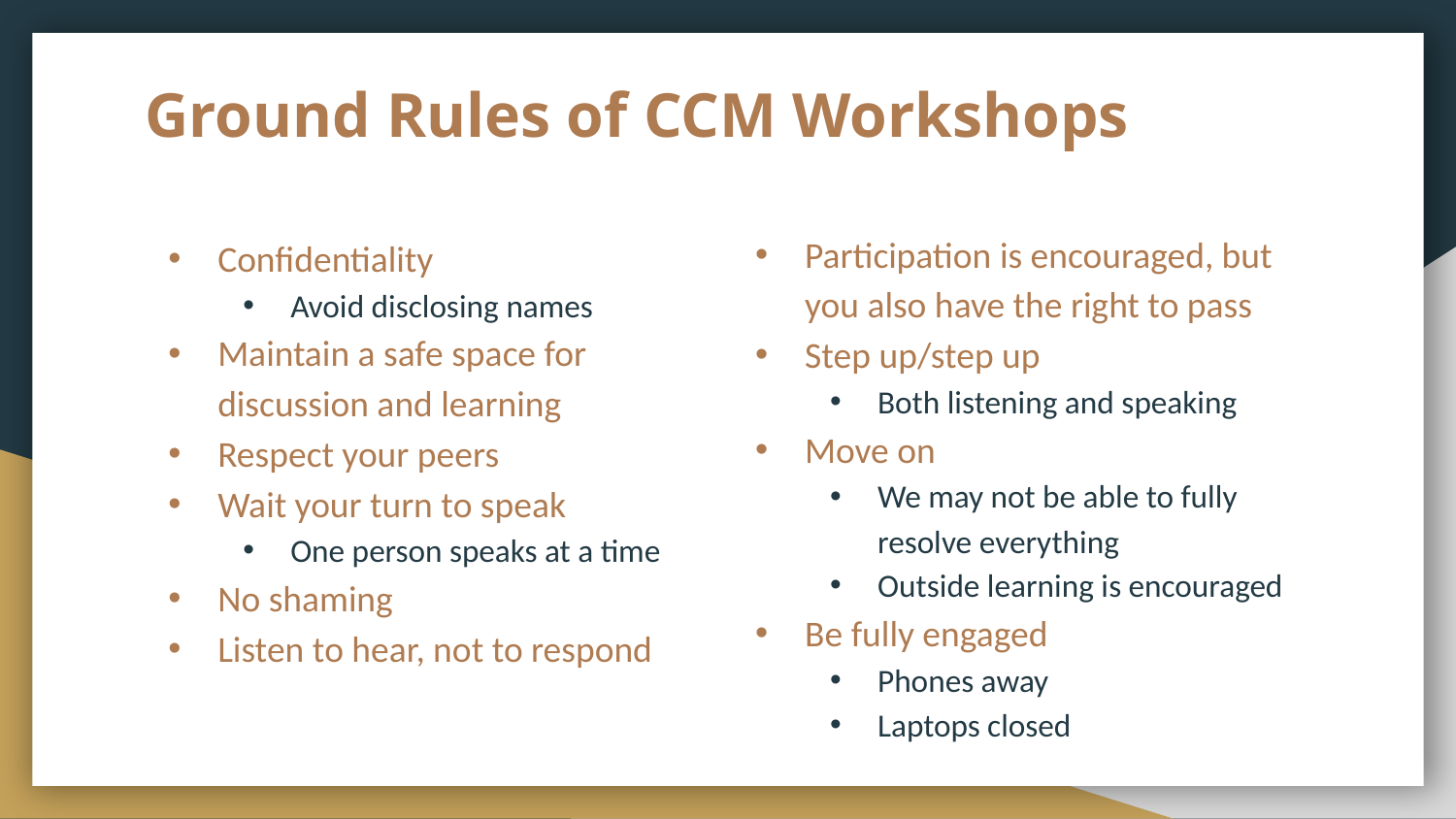

# Ground Rules of CCM Workshops
Participation is encouraged, but you also have the right to pass
Step up/step up
Both listening and speaking
Move on
We may not be able to fully resolve everything
Outside learning is encouraged
Be fully engaged
Phones away
Laptops closed
Confidentiality
Avoid disclosing names
Maintain a safe space for discussion and learning
Respect your peers
Wait your turn to speak
One person speaks at a time
No shaming
Listen to hear, not to respond

## Slide 3
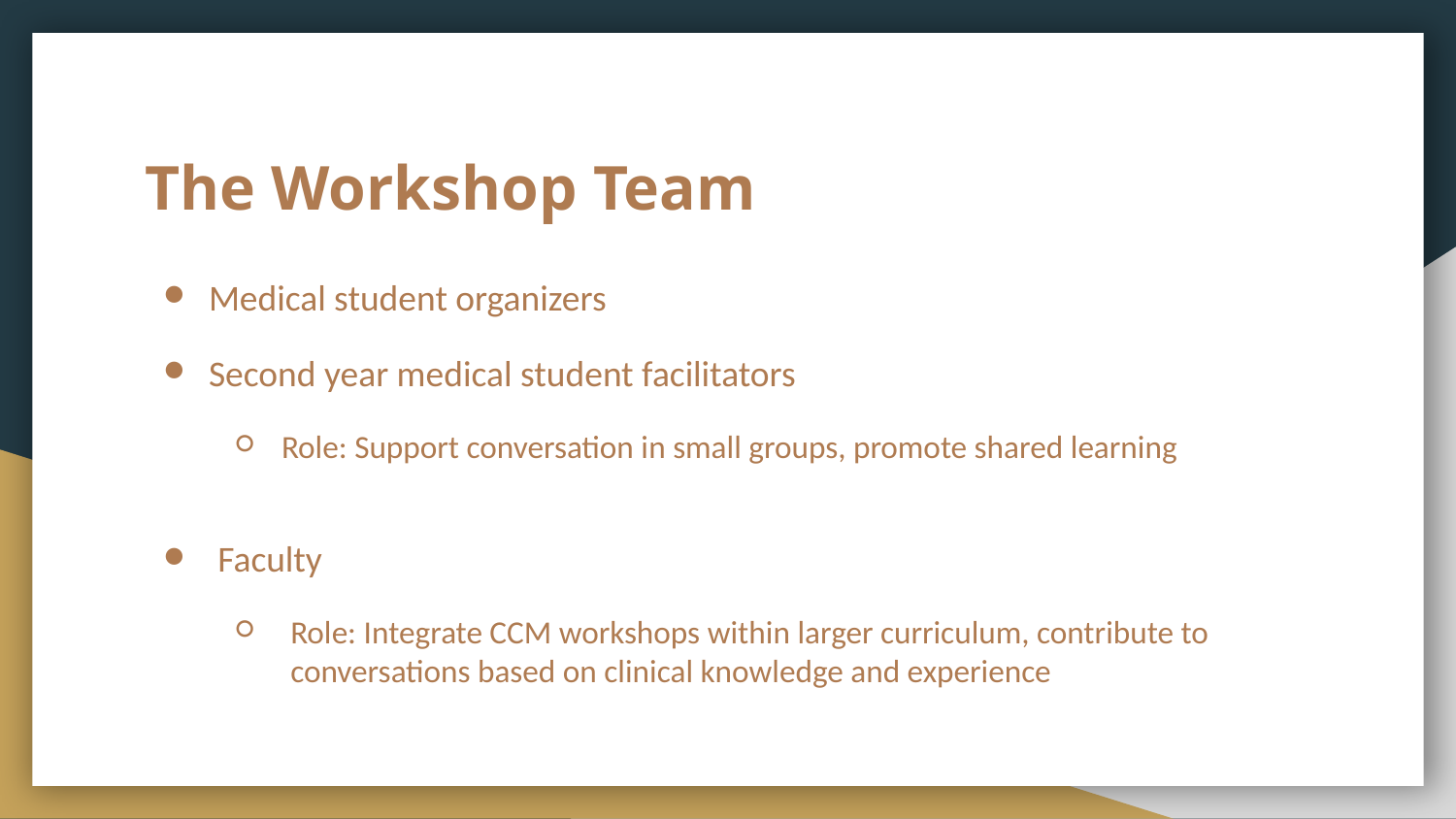

# The Workshop Team
Medical student organizers
Second year medical student facilitators
Role: Support conversation in small groups, promote shared learning
Faculty
Role: Integrate CCM workshops within larger curriculum, contribute to conversations based on clinical knowledge and experience

## Slide 4
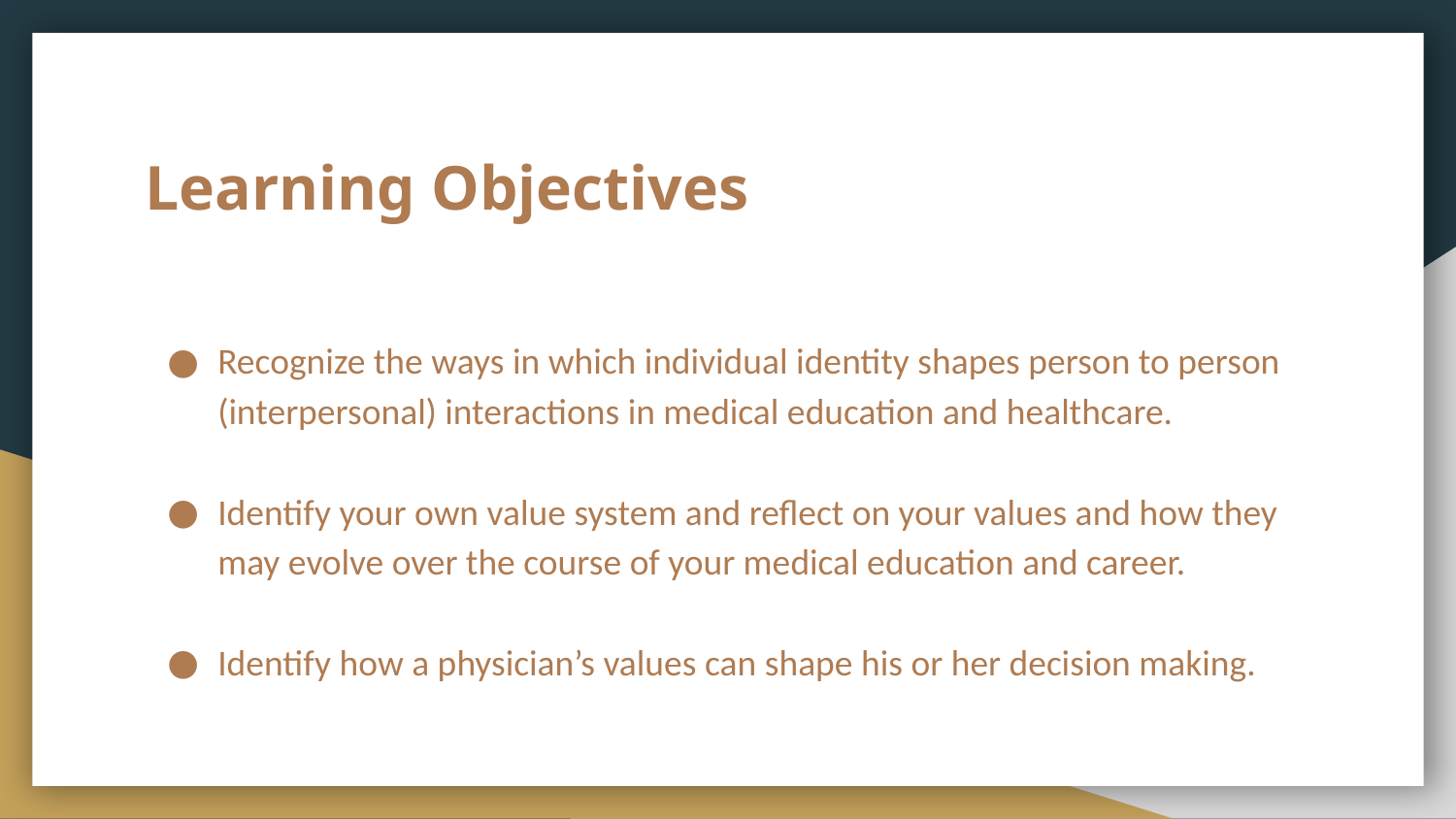

# Learning Objectives
Recognize the ways in which individual identity shapes person to person (interpersonal) interactions in medical education and healthcare.
Identify your own value system and reflect on your values and how they may evolve over the course of your medical education and career.
Identify how a physician’s values can shape his or her decision making.

## Slide 5
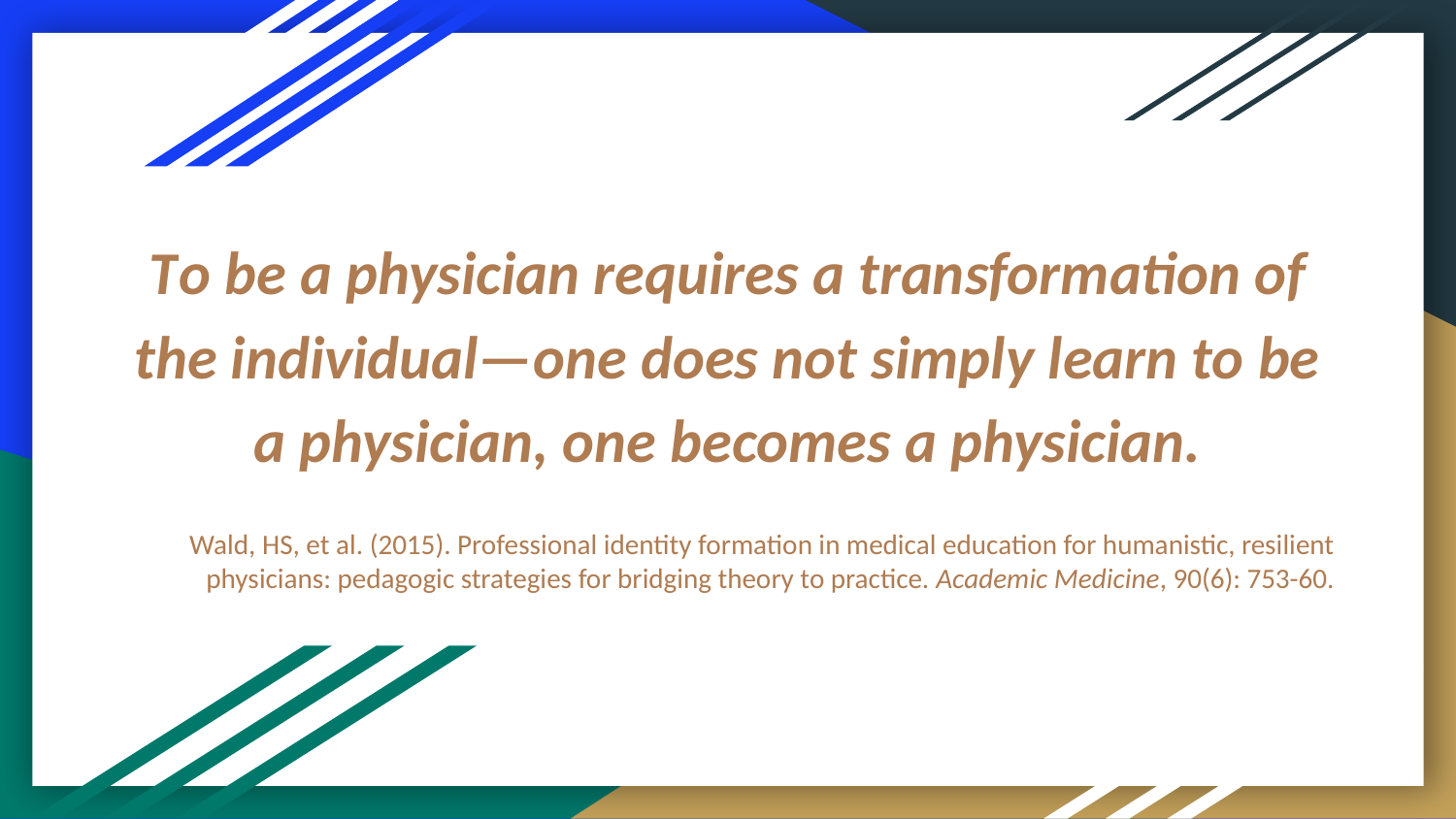

# To be a physician requires a transformation of the individual—one does not simply learn to be a physician, one becomes a physician.
Wald, HS, et al. (2015). Professional identity formation in medical education for humanistic, resilient physicians: pedagogic strategies for bridging theory to practice. Academic Medicine, 90(6): 753-60.

## Slide 6
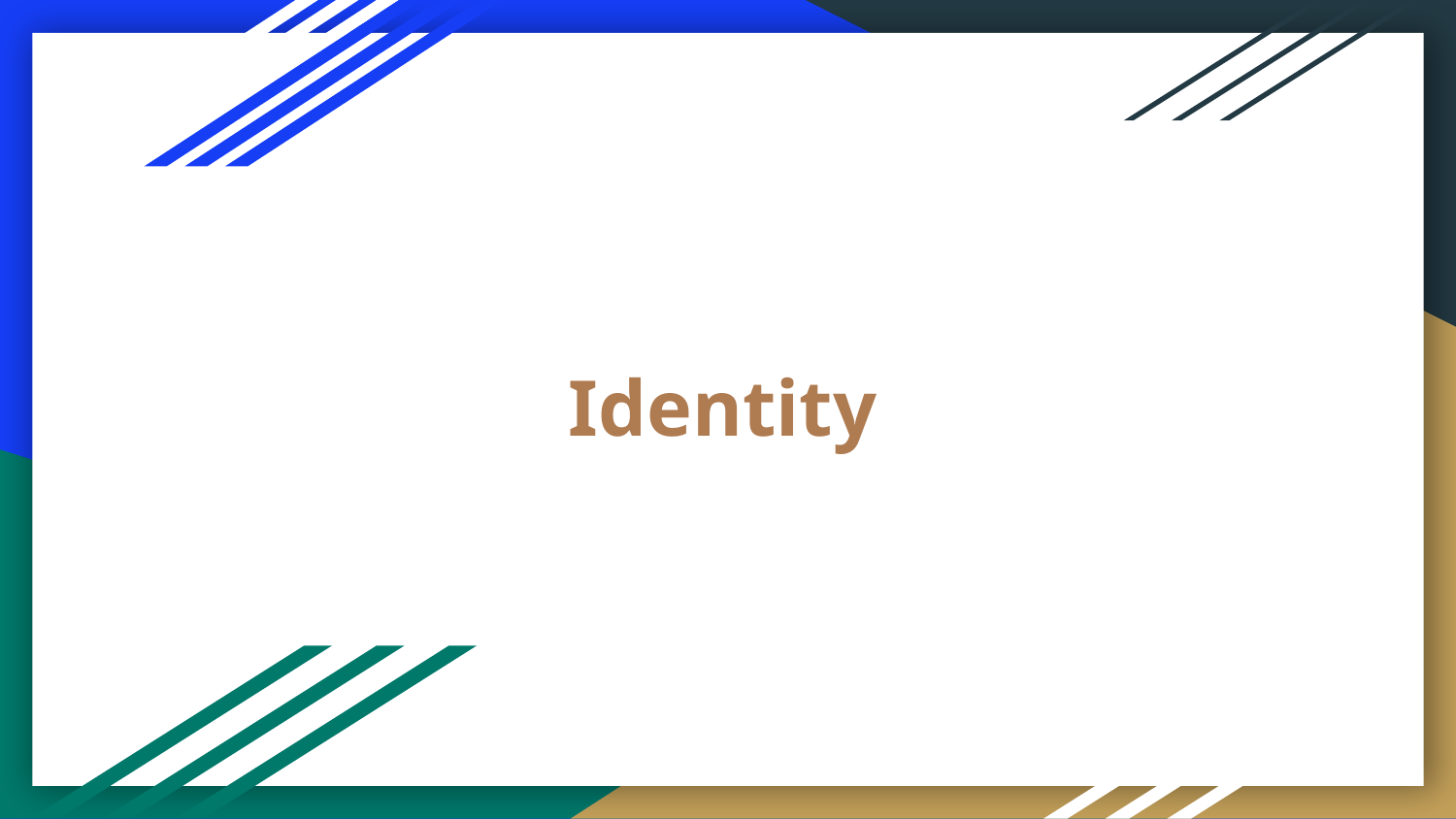

# Identity

## Slide 7
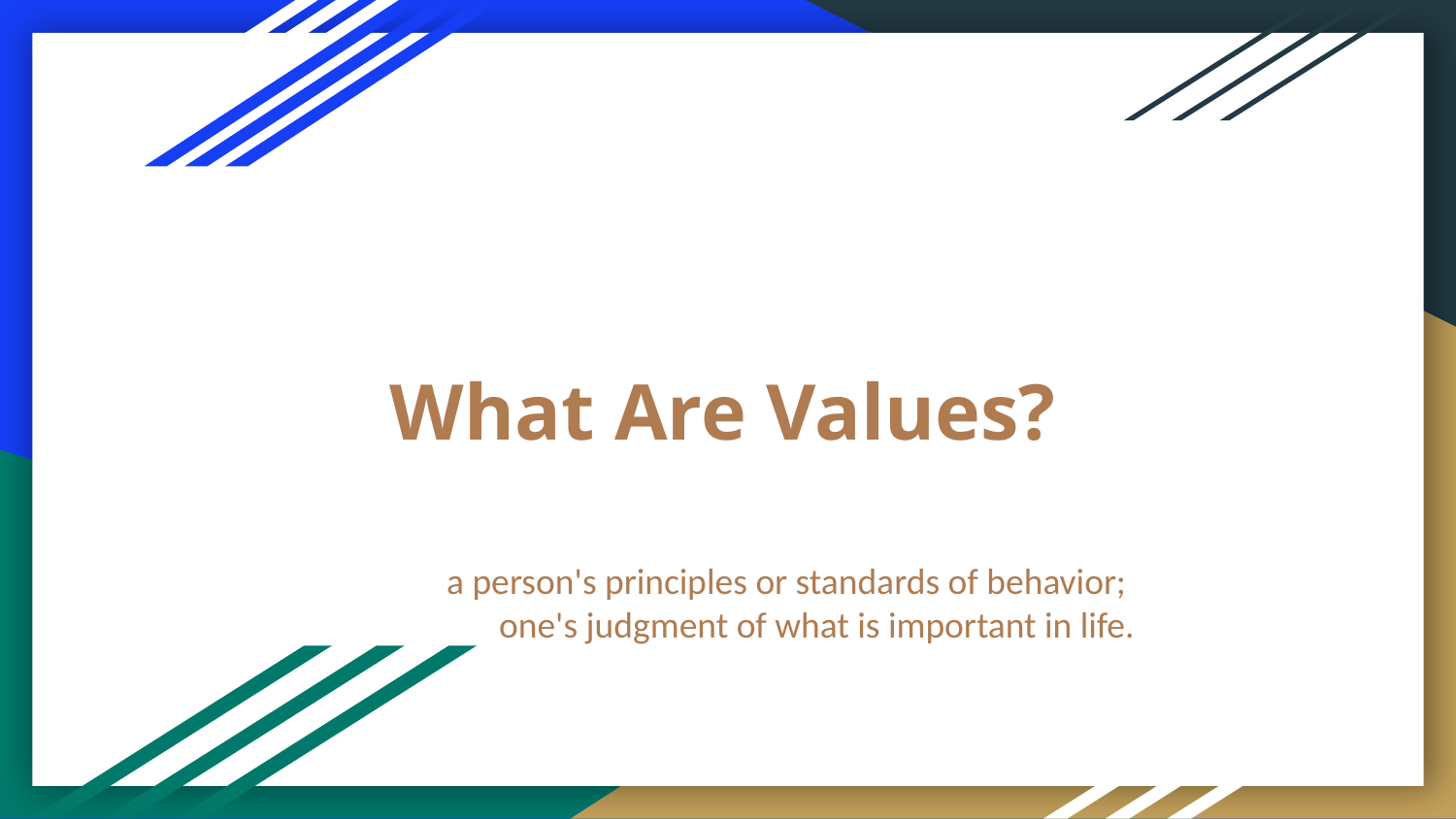

# What Are Values?
a person's principles or standards of behavior;
one's judgment of what is important in life.

## Slide 8
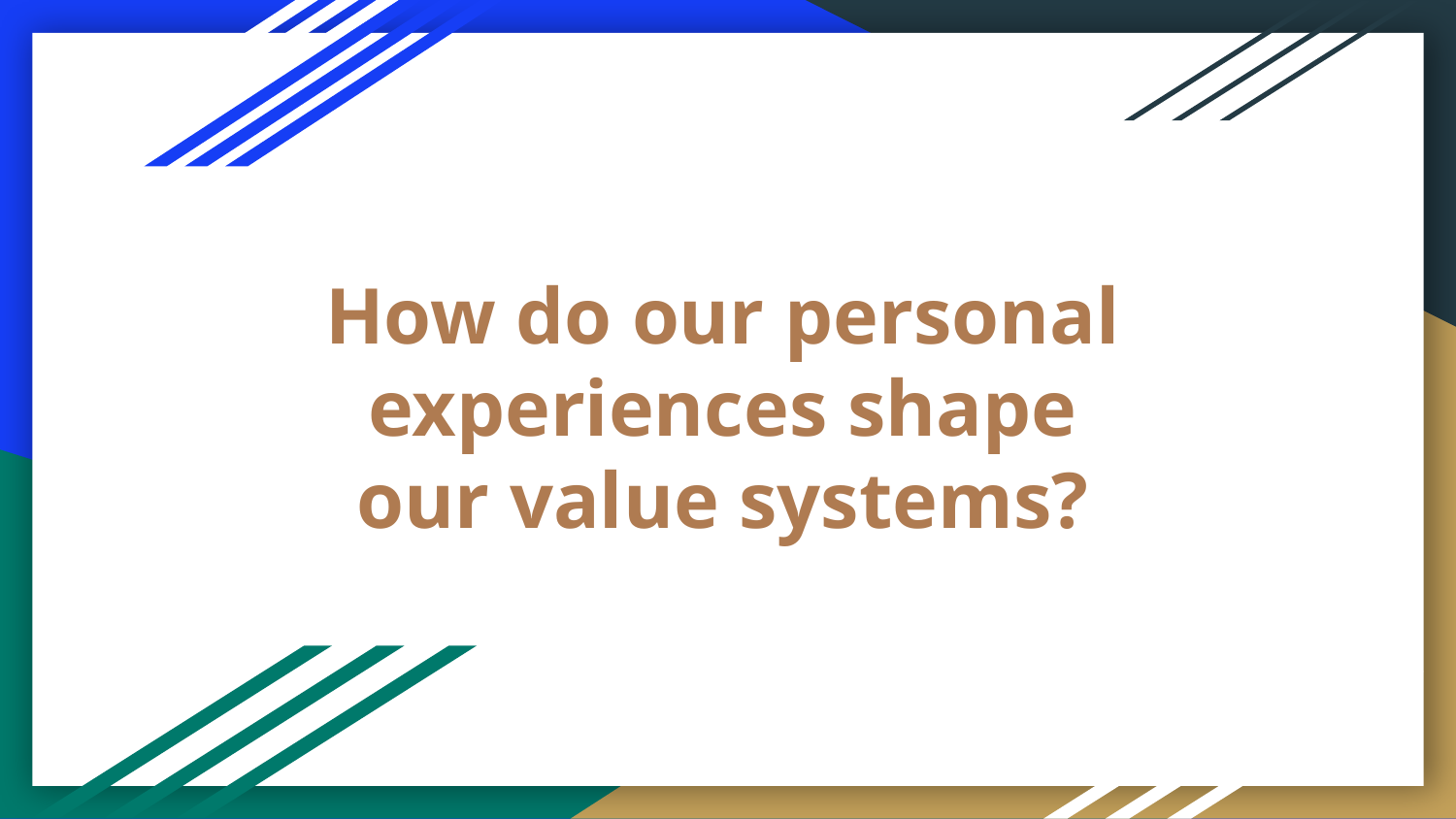

# How do our personal experiences shape our value systems?

## Slide 9
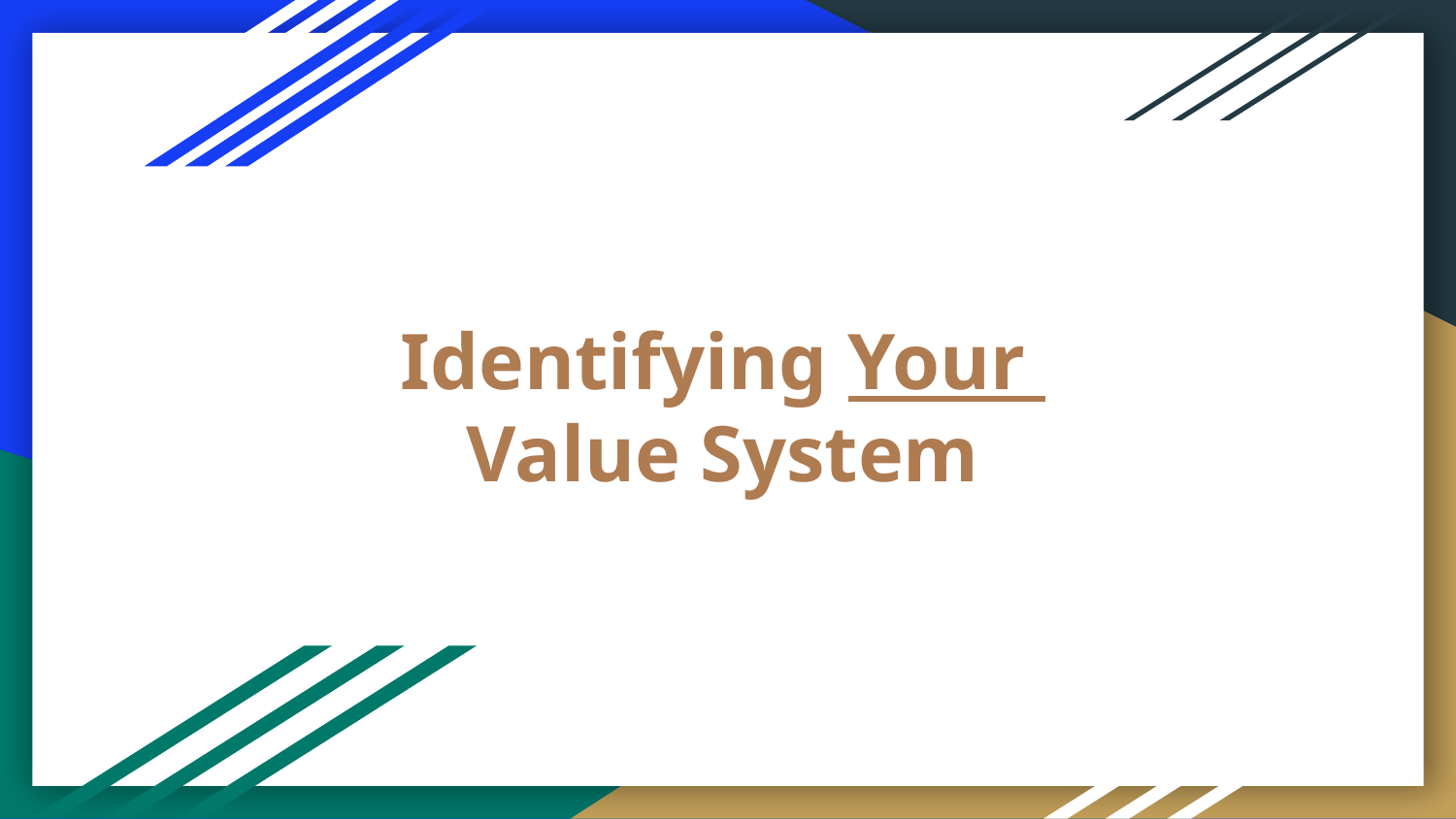

# Identifying Your Value System

## Slide 10
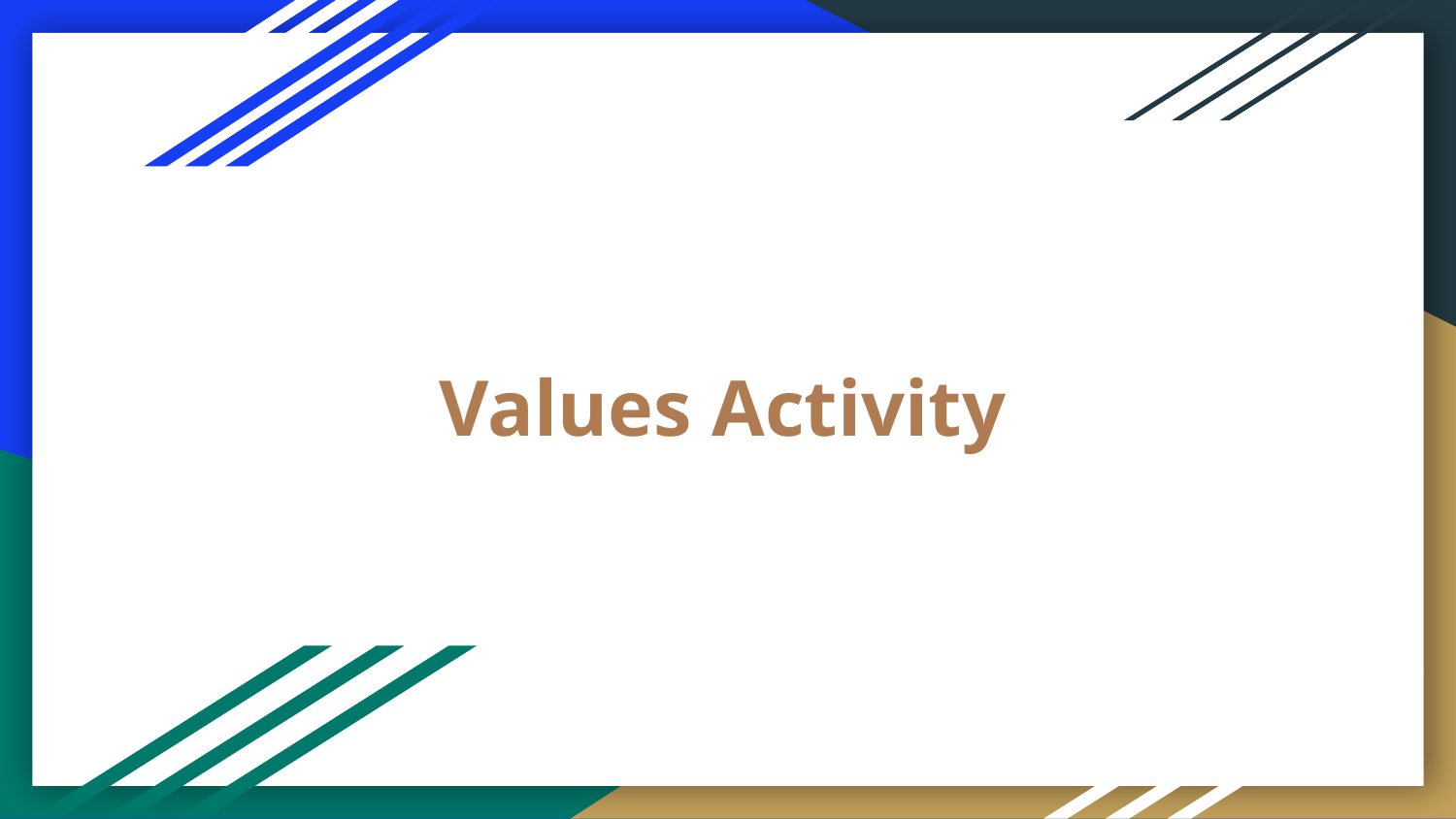

# Values Activity

## Slide 11
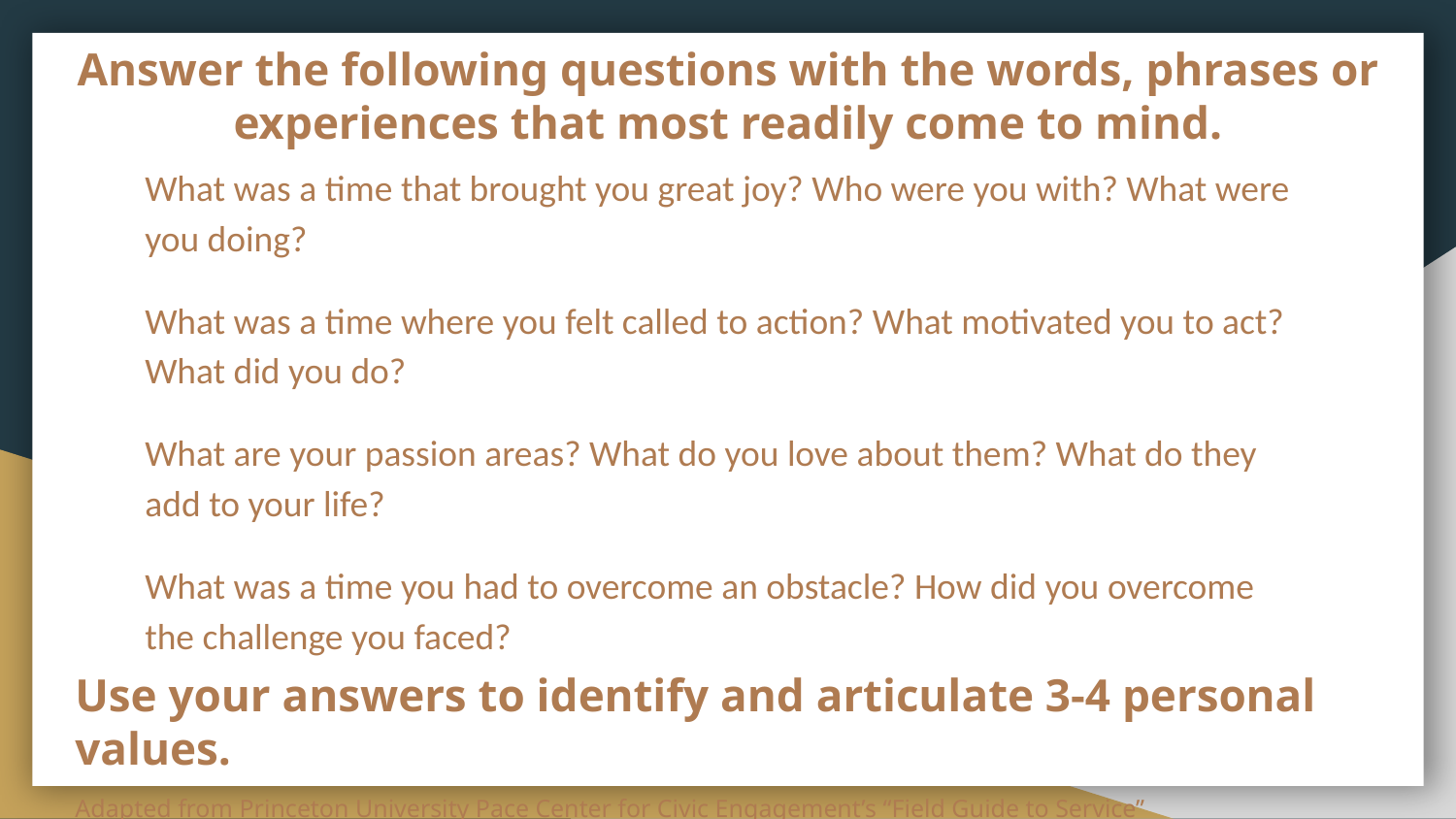

# Answer the following questions with the words, phrases or experiences that most readily come to mind.
What was a time that brought you great joy? Who were you with? What were you doing?
What was a time where you felt called to action? What motivated you to act? What did you do?
What are your passion areas? What do you love about them? What do they add to your life?
What was a time you had to overcome an obstacle? How did you overcome the challenge you faced?
Use your answers to identify and articulate 3-4 personal values.
Adapted from Princeton University Pace Center for Civic Engagement’s “Field Guide to Service”

## Slide 12
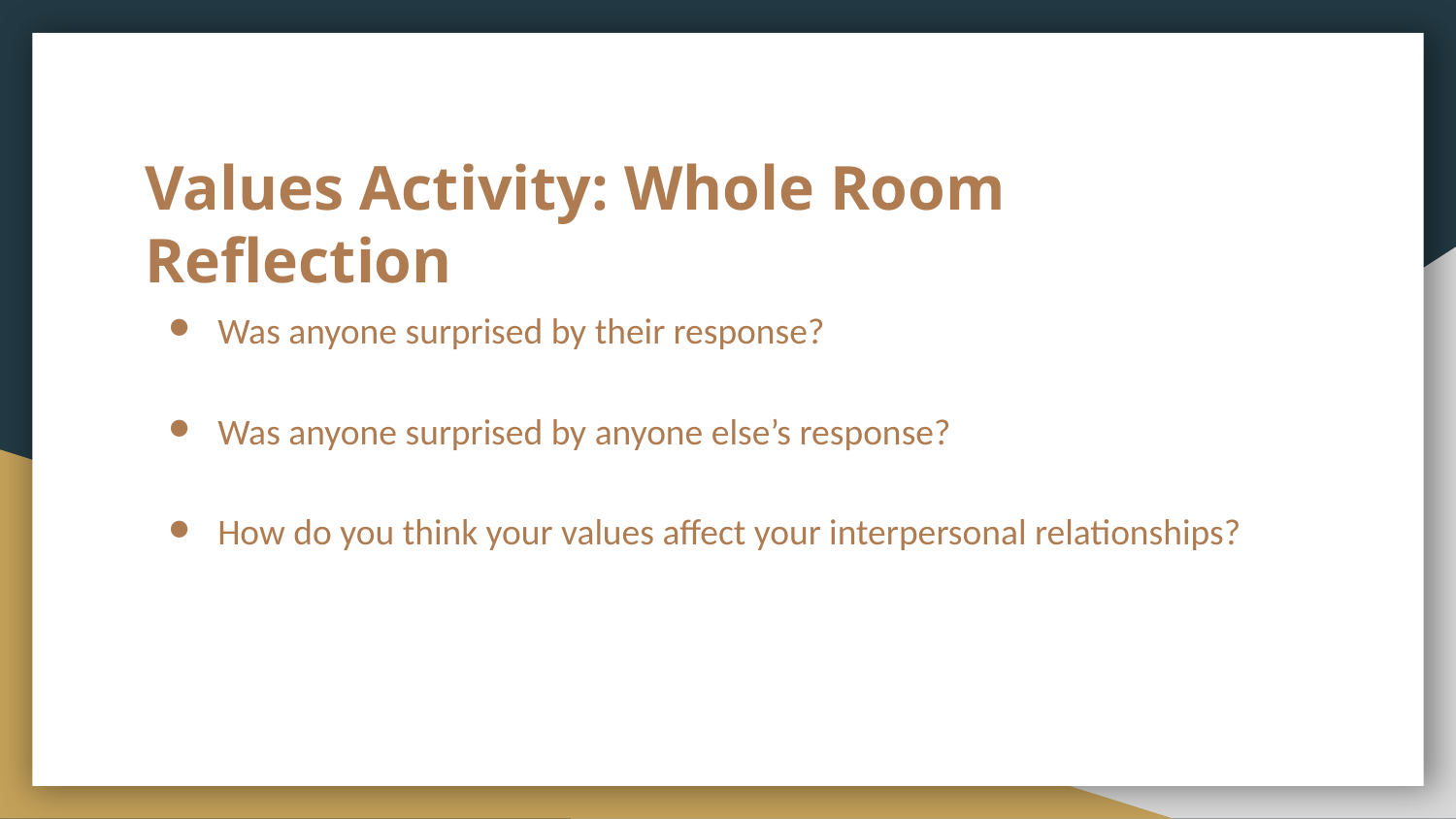

# Values Activity: Whole Room Reflection
Was anyone surprised by their response?
Was anyone surprised by anyone else’s response?
How do you think your values affect your interpersonal relationships?

## Slide 13
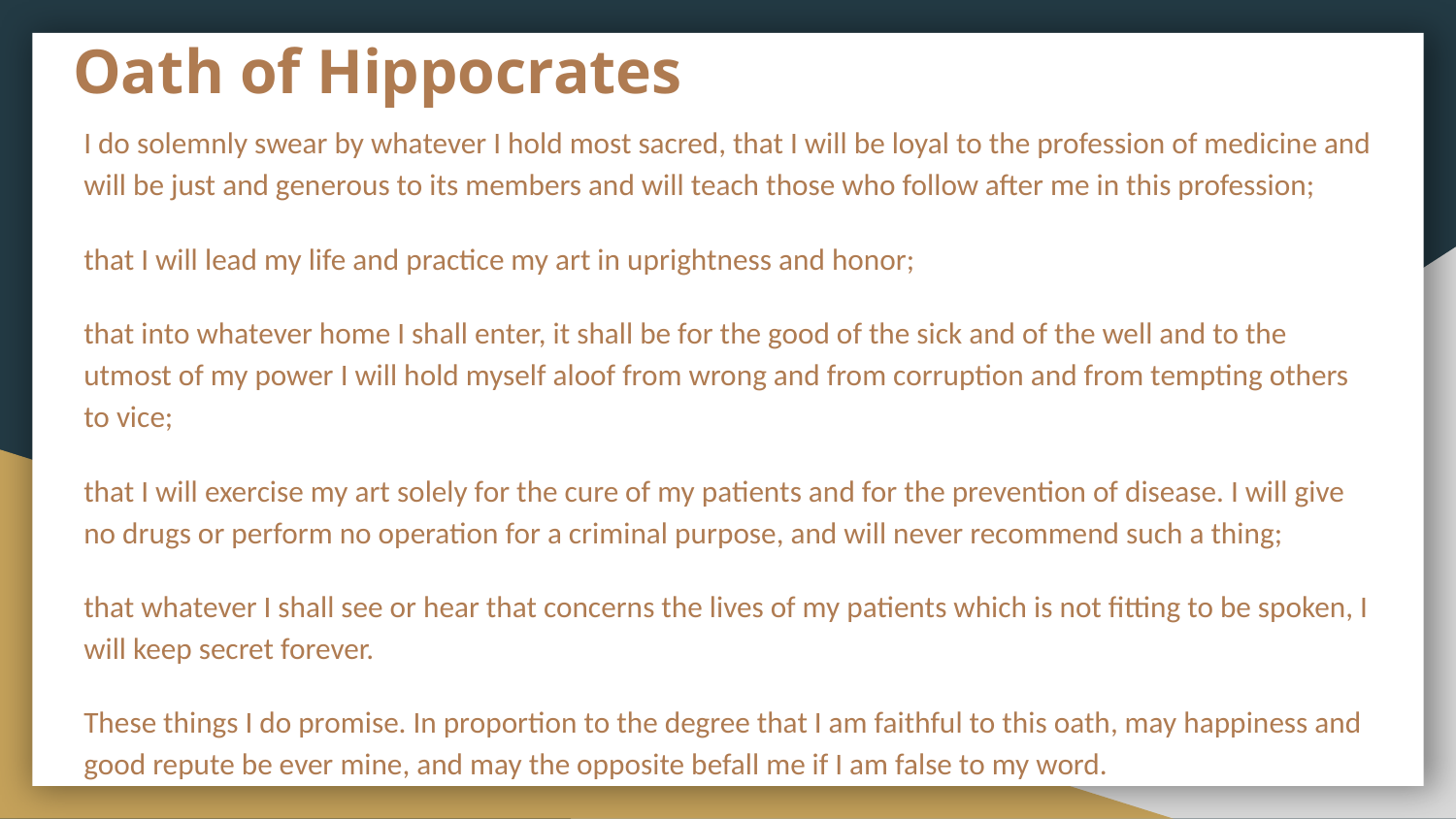

# Oath of Hippocrates
I do solemnly swear by whatever I hold most sacred, that I will be loyal to the profession of medicine and will be just and generous to its members and will teach those who follow after me in this profession;
that I will lead my life and practice my art in uprightness and honor;
that into whatever home I shall enter, it shall be for the good of the sick and of the well and to the utmost of my power I will hold myself aloof from wrong and from corruption and from tempting others to vice;
that I will exercise my art solely for the cure of my patients and for the prevention of disease. I will give no drugs or perform no operation for a criminal purpose, and will never recommend such a thing;
that whatever I shall see or hear that concerns the lives of my patients which is not fitting to be spoken, I will keep secret forever.
These things I do promise. In proportion to the degree that I am faithful to this oath, may happiness and good repute be ever mine, and may the opposite befall me if I am false to my word.

## Slide 14
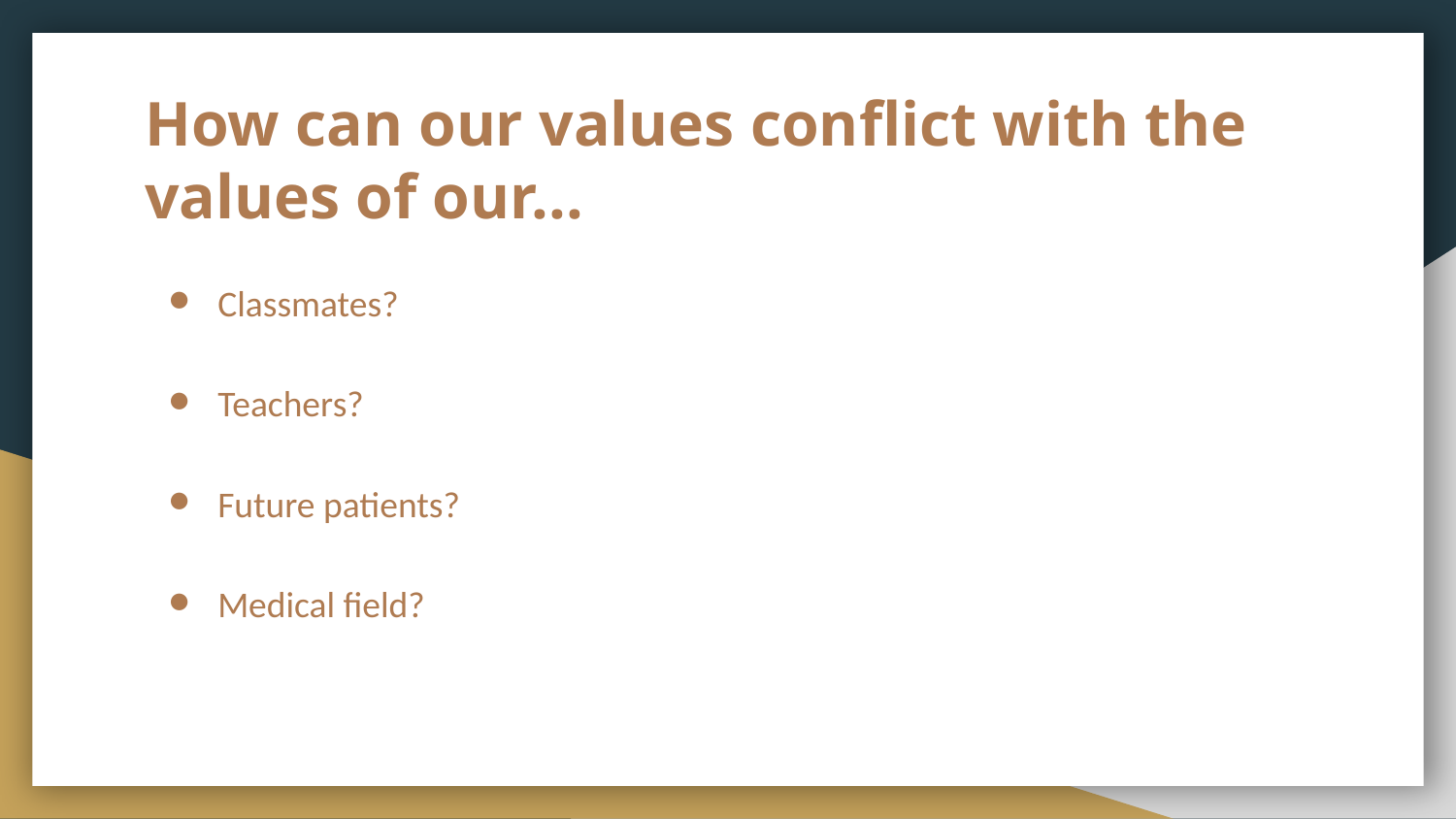

# How can our values conflict with the values of our...
Classmates?
Teachers?
Future patients?
Medical field?

## Slide 15
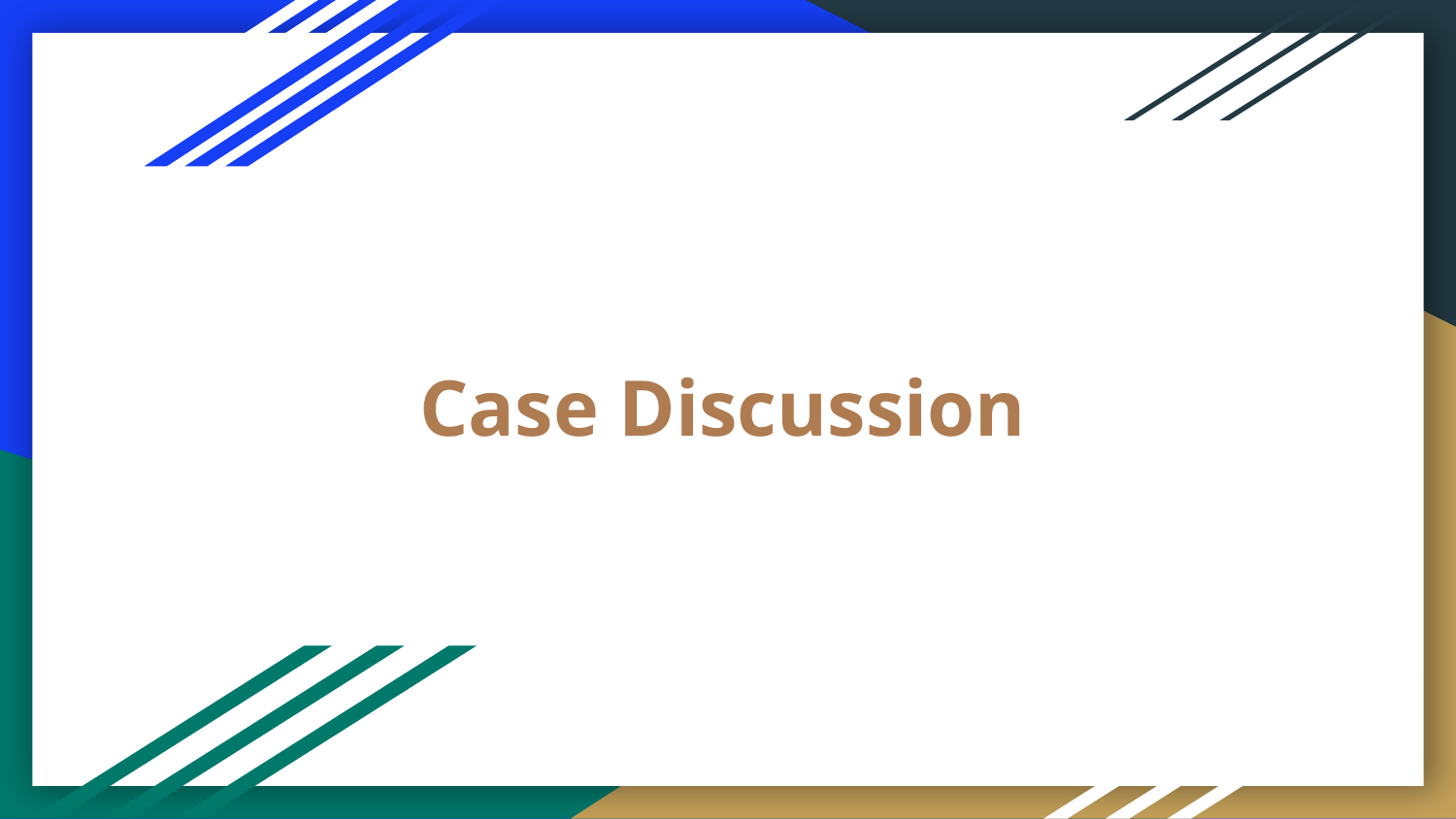

# Case Discussion

## Slide 16
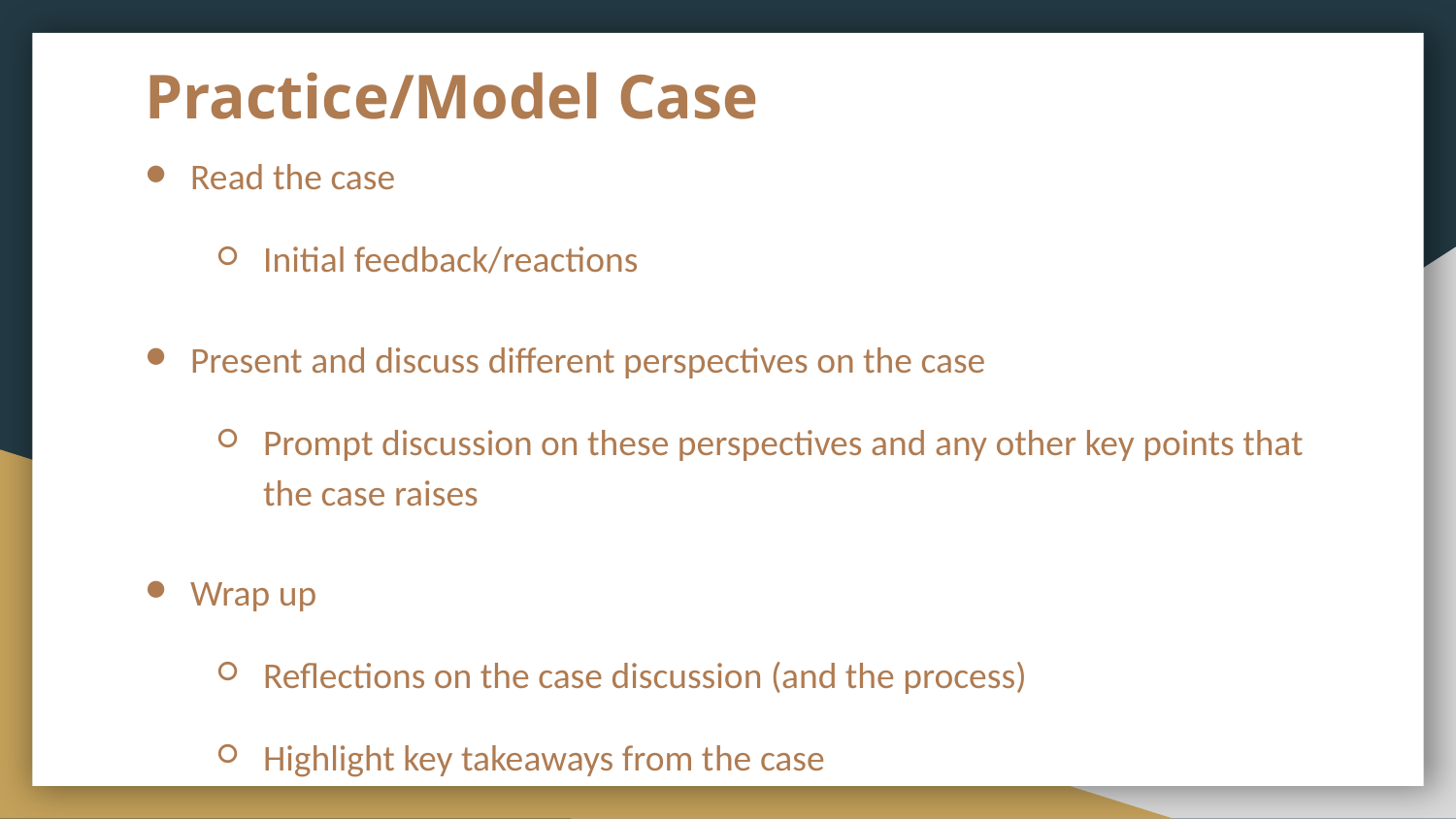

# Practice/Model Case
Read the case
Initial feedback/reactions
Present and discuss different perspectives on the case
Prompt discussion on these perspectives and any other key points that the case raises
Wrap up
Reflections on the case discussion (and the process)
Highlight key takeaways from the case

## Slide 17
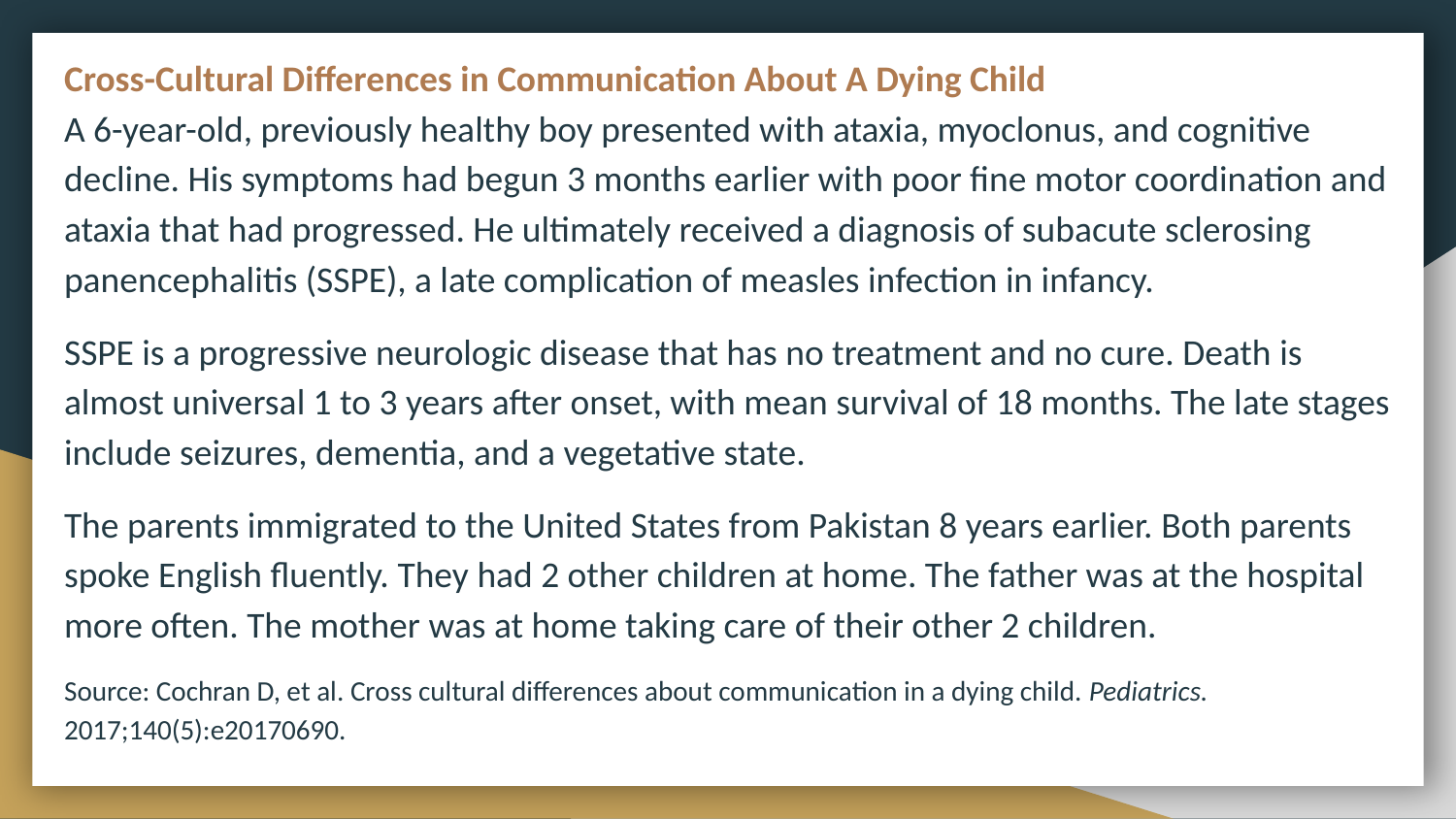

Cross-Cultural Differences in Communication About A Dying Child
A 6-year-old, previously healthy boy presented with ataxia, myoclonus, and cognitive decline. His symptoms had begun 3 months earlier with poor fine motor coordination and ataxia that had progressed. He ultimately received a diagnosis of subacute sclerosing panencephalitis (SSPE), a late complication of measles infection in infancy.
SSPE is a progressive neurologic disease that has no treatment and no cure. Death is almost universal 1 to 3 years after onset, with mean survival of 18 months. The late stages include seizures, dementia, and a vegetative state.
The parents immigrated to the United States from Pakistan 8 years earlier. Both parents spoke English fluently. They had 2 other children at home. The father was at the hospital more often. The mother was at home taking care of their other 2 children.
Source: Cochran D, et al. Cross cultural differences about communication in a dying child. Pediatrics. 2017;140(5):e20170690.

## Slide 18
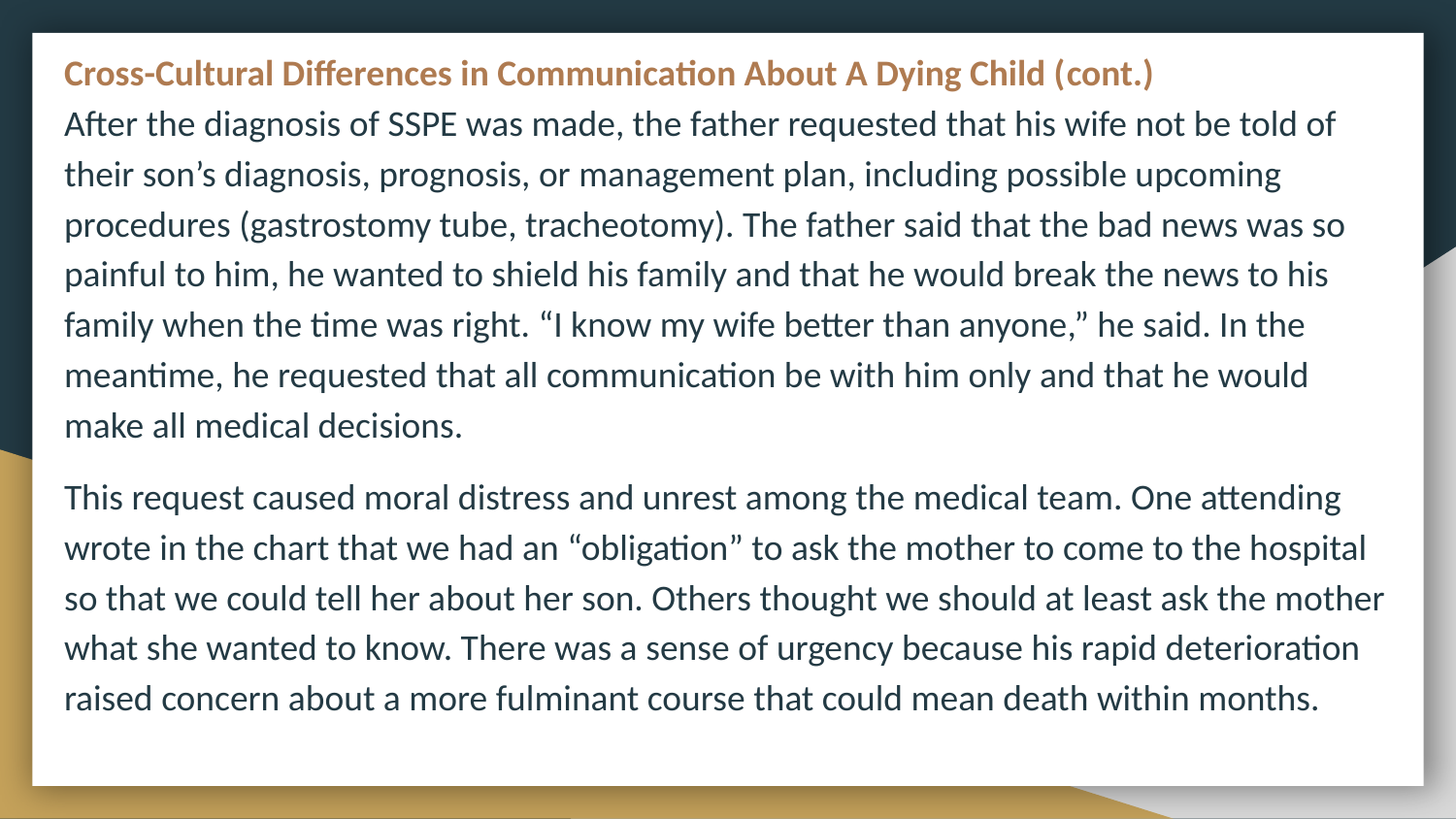

Cross-Cultural Differences in Communication About A Dying Child (cont.)
After the diagnosis of SSPE was made, the father requested that his wife not be told of their son’s diagnosis, prognosis, or management plan, including possible upcoming procedures (gastrostomy tube, tracheotomy). The father said that the bad news was so painful to him, he wanted to shield his family and that he would break the news to his family when the time was right. “I know my wife better than anyone,” he said. In the meantime, he requested that all communication be with him only and that he would make all medical decisions.
This request caused moral distress and unrest among the medical team. One attending wrote in the chart that we had an “obligation” to ask the mother to come to the hospital so that we could tell her about her son. Others thought we should at least ask the mother what she wanted to know. There was a sense of urgency because his rapid deterioration raised concern about a more fulminant course that could mean death within months.

## Slide 19
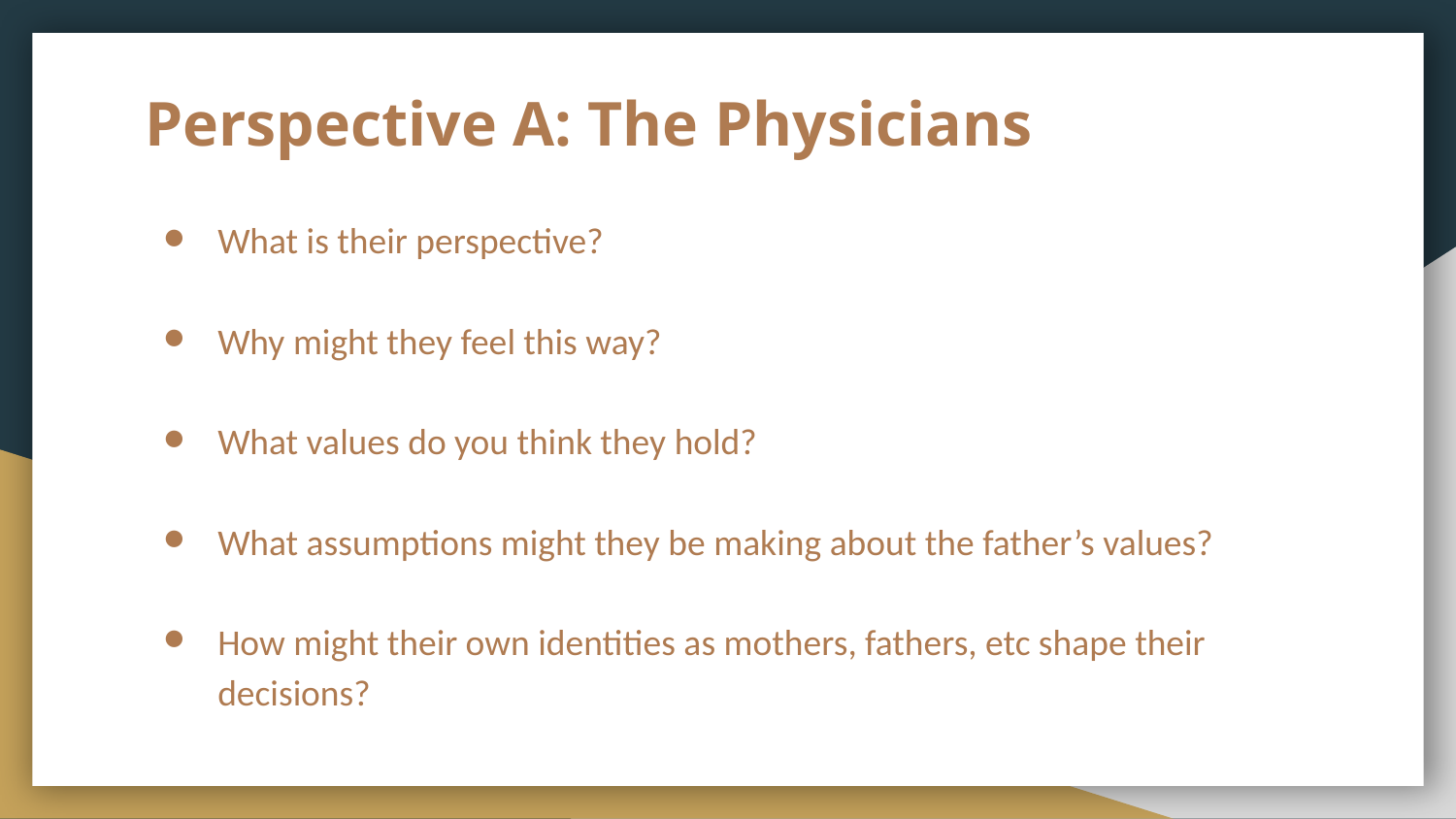

# Perspective A: The Physicians
What is their perspective?
Why might they feel this way?
What values do you think they hold?
What assumptions might they be making about the father’s values?
How might their own identities as mothers, fathers, etc shape their decisions?

## Slide 20
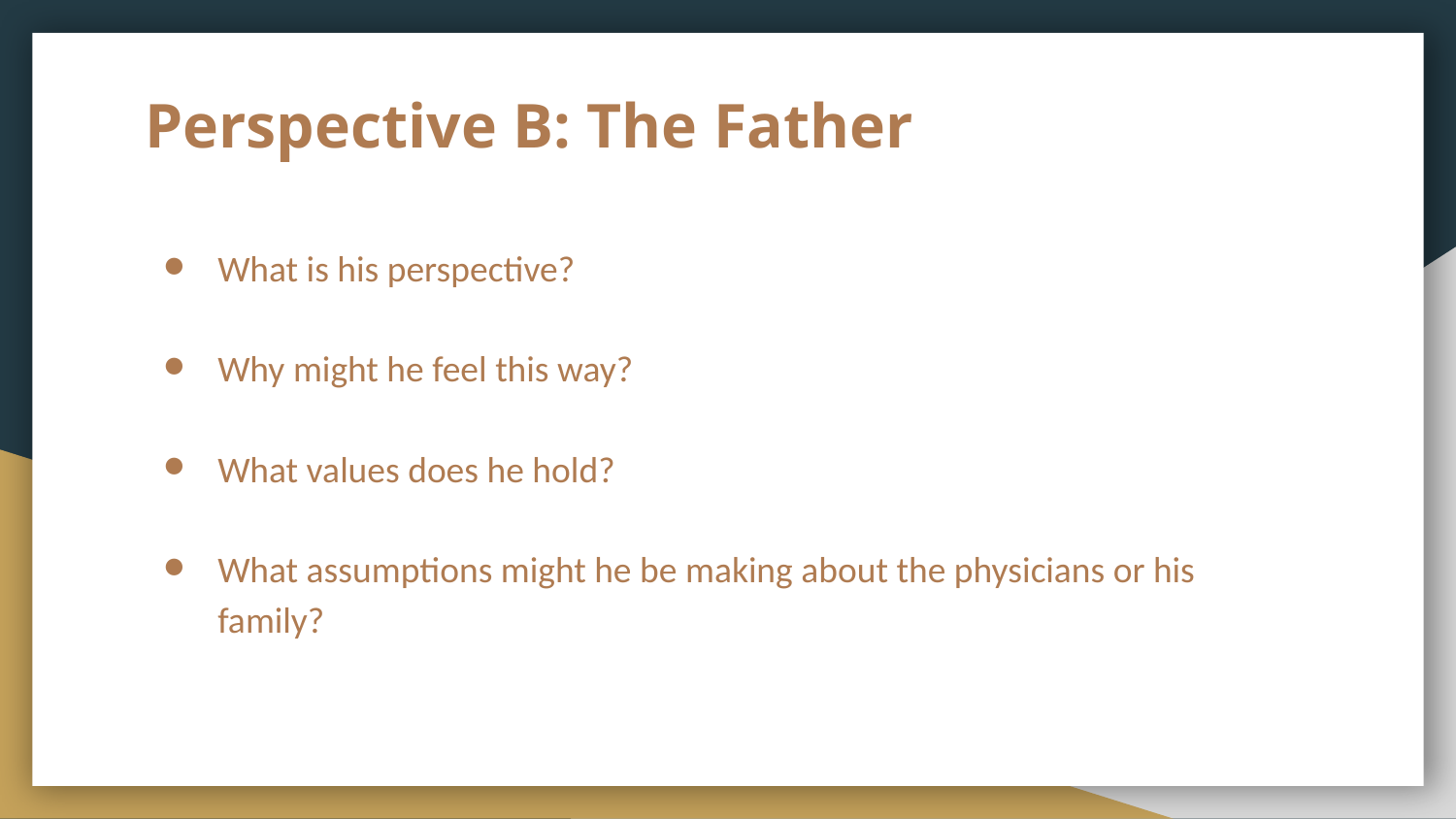

# Perspective B: The Father
What is his perspective?
Why might he feel this way?
What values does he hold?
What assumptions might he be making about the physicians or his family?

## Slide 21
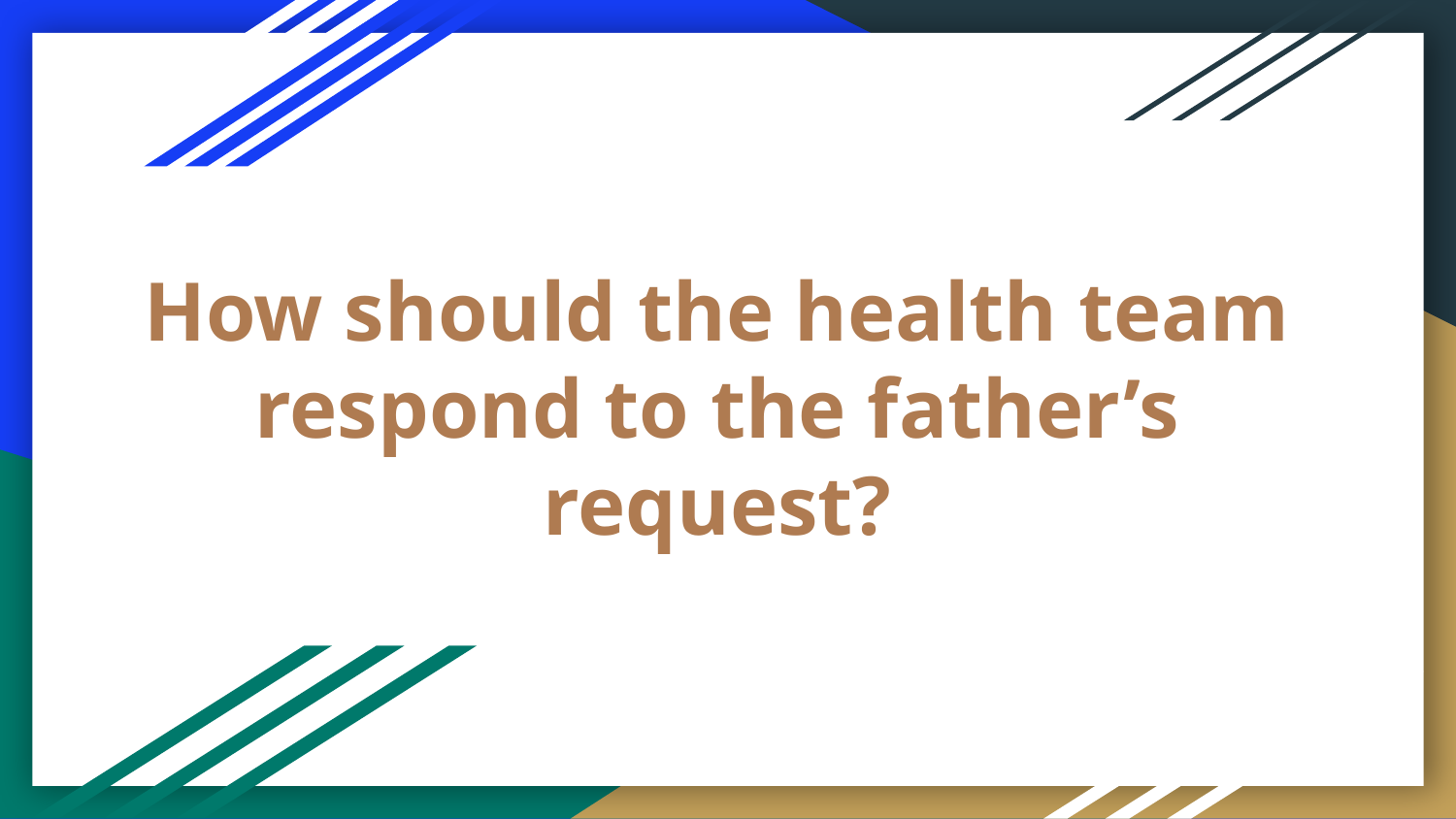

# How should the health team respond to the father’s request?

## Slide 22
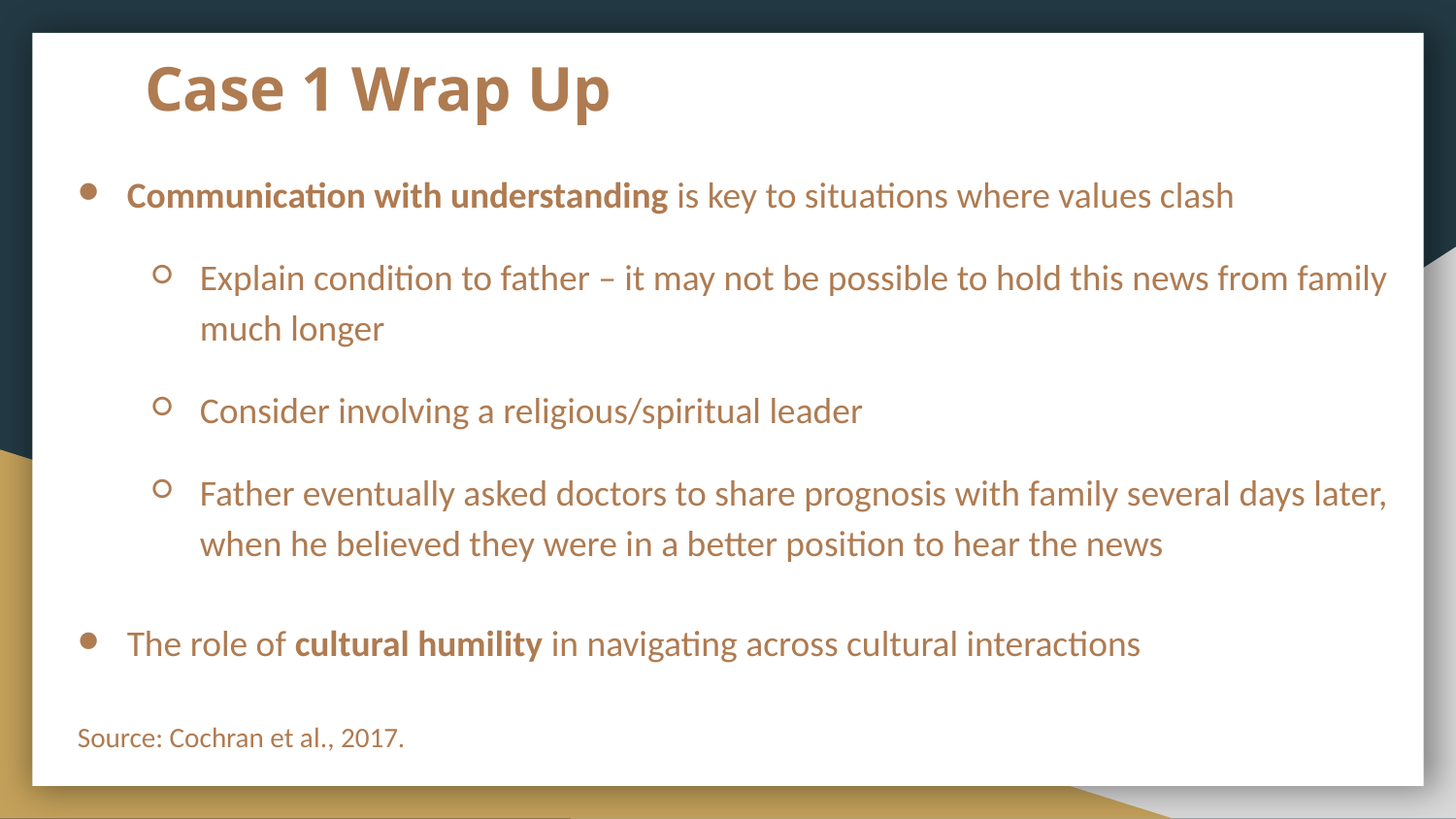

# Case 1 Wrap Up
Communication with understanding is key to situations where values clash
Explain condition to father – it may not be possible to hold this news from family much longer
Consider involving a religious/spiritual leader
Father eventually asked doctors to share prognosis with family several days later, when he believed they were in a better position to hear the news
The role of cultural humility in navigating across cultural interactions
Source: Cochran et al., 2017.

## Slide 23
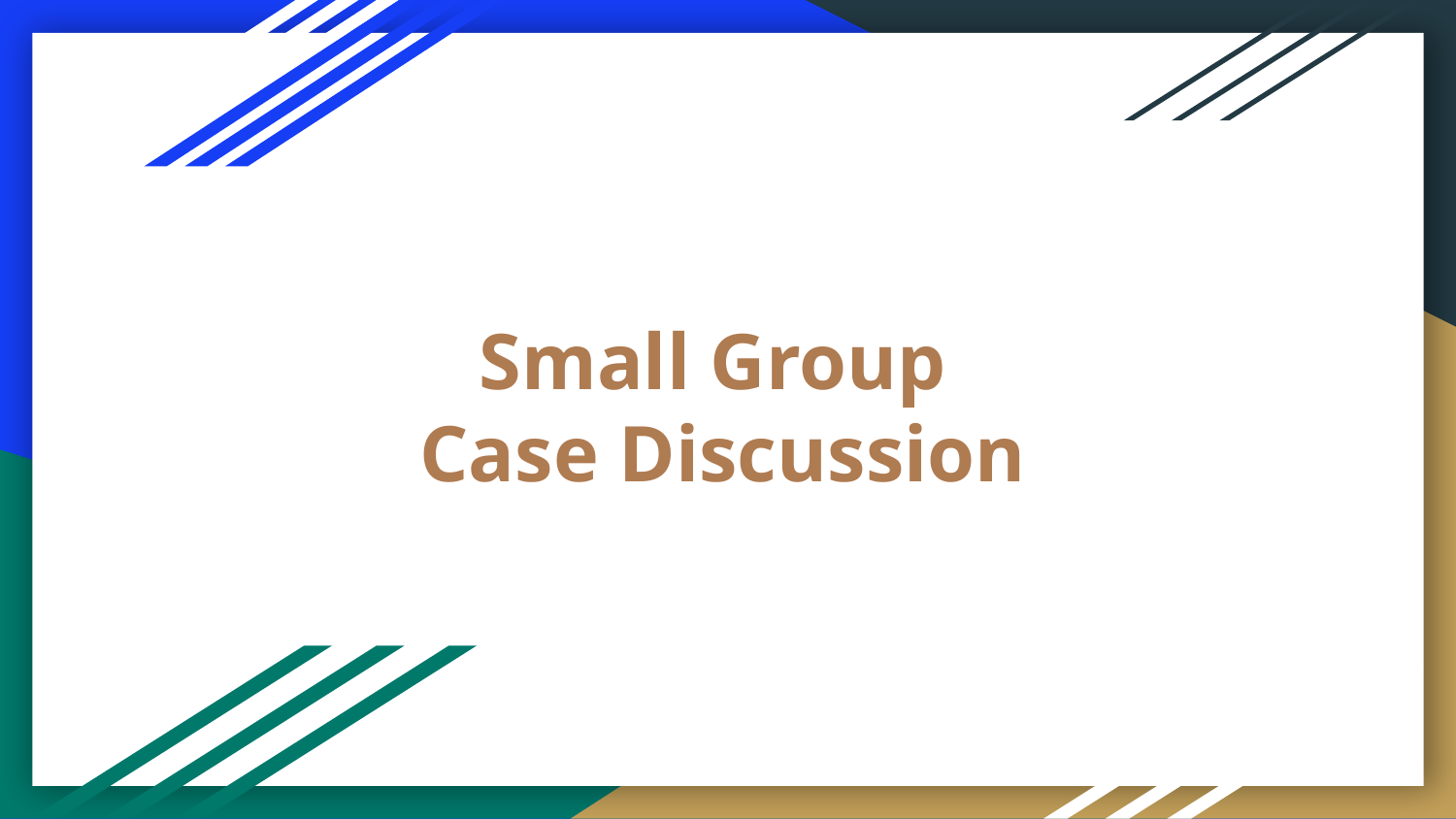

# Small Group Case Discussion

## Slide 24
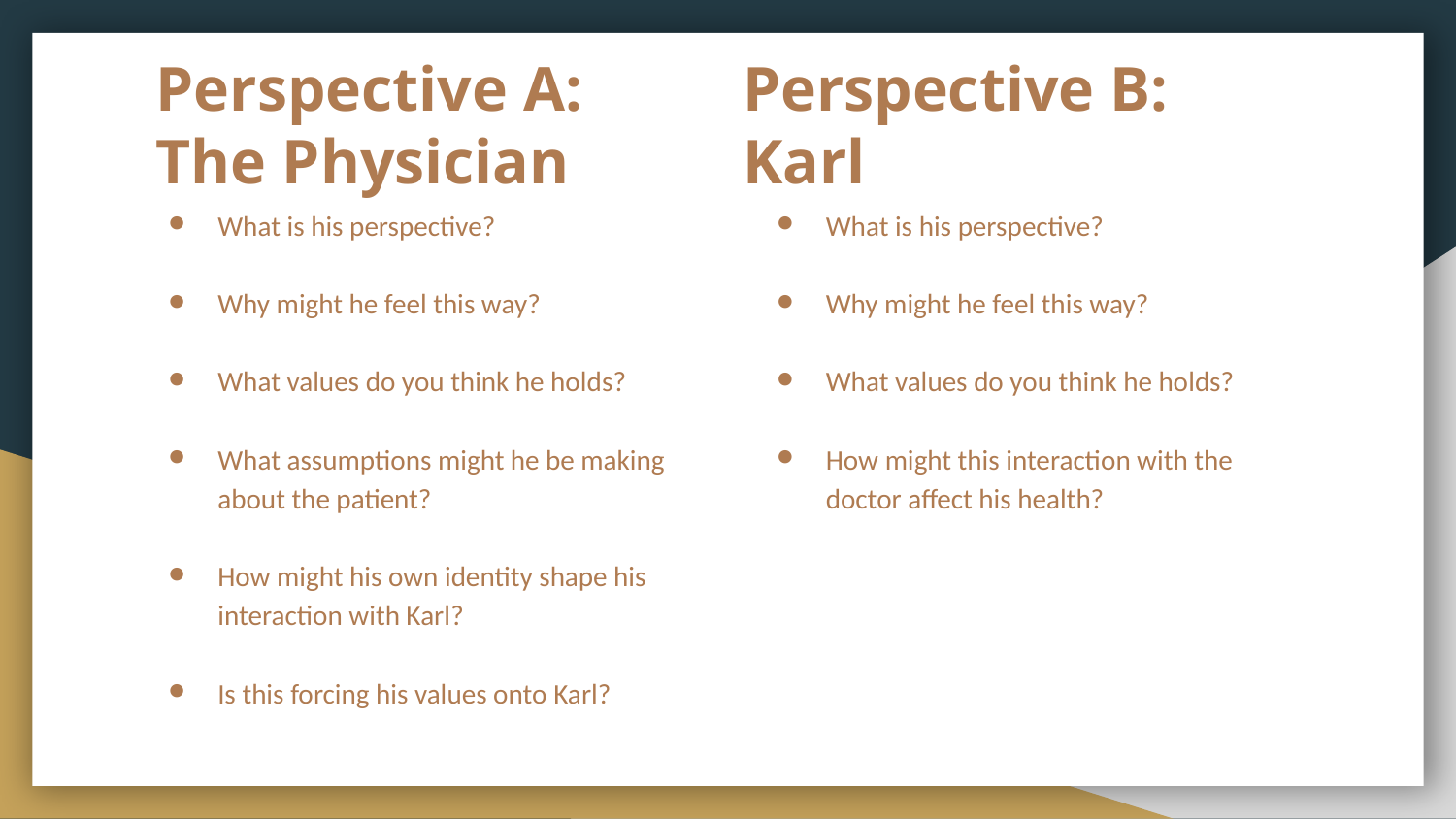

# Perspective A: The Physician
Perspective B:
Karl
What is his perspective?
Why might he feel this way?
What values do you think he holds?
What assumptions might he be making about the patient?
How might his own identity shape his interaction with Karl?
Is this forcing his values onto Karl?
What is his perspective?
Why might he feel this way?
What values do you think he holds?
How might this interaction with the doctor affect his health?

## Slide 25
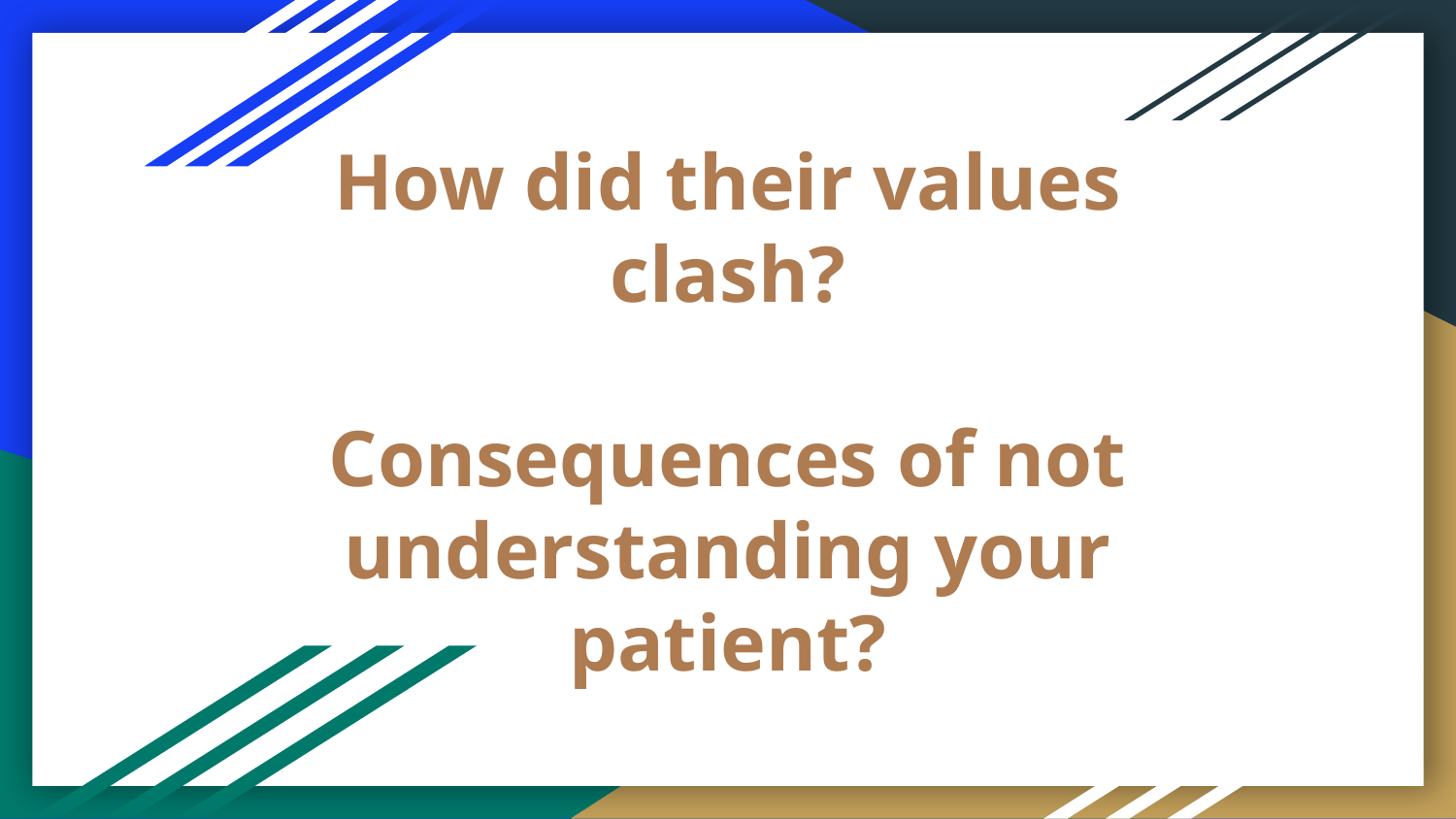

# How did their values clash? Consequences of not understanding your patient?

## Slide 26
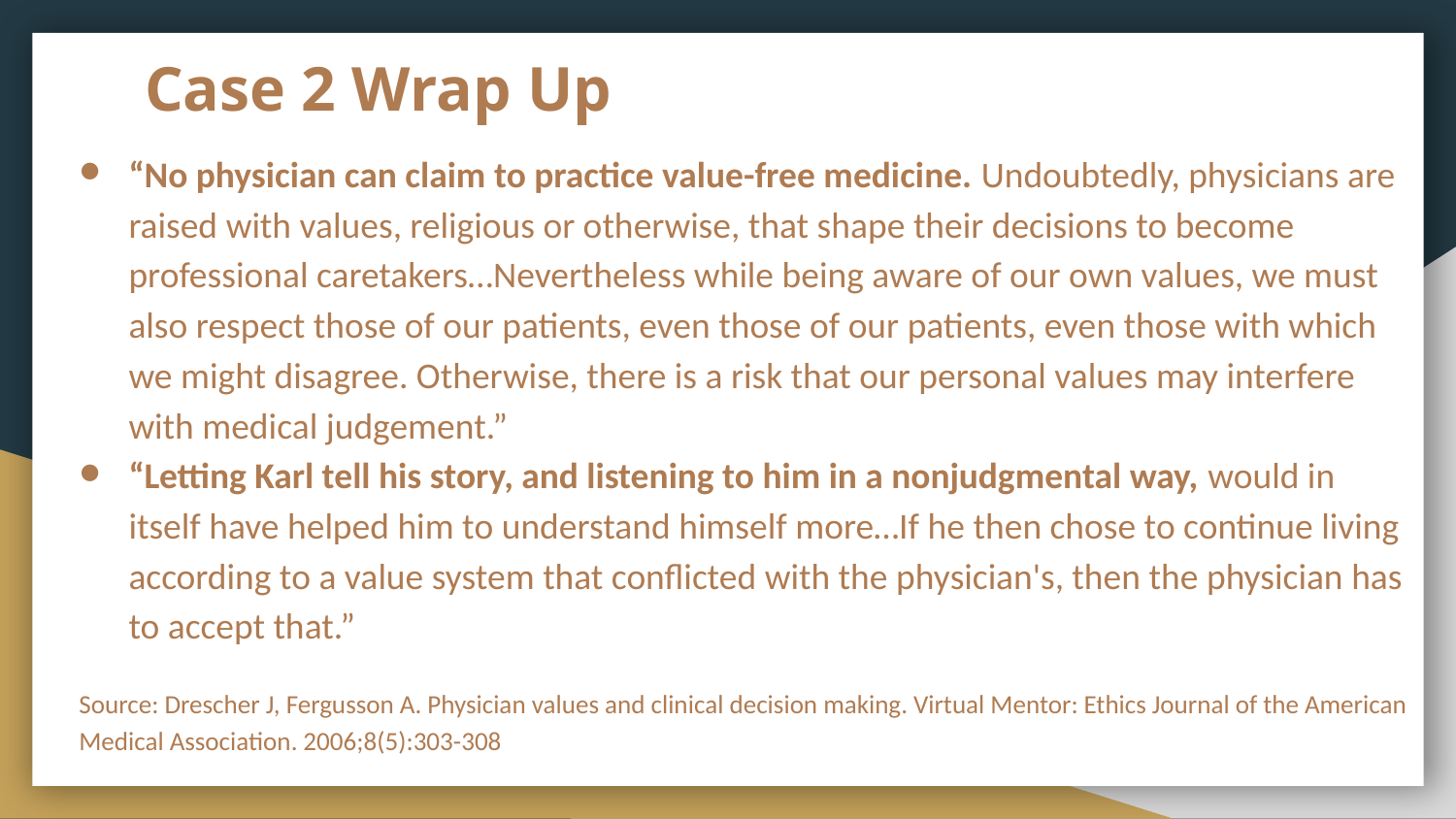

# Case 2 Wrap Up
“No physician can claim to practice value-free medicine. Undoubtedly, physicians are raised with values, religious or otherwise, that shape their decisions to become professional caretakers…Nevertheless while being aware of our own values, we must also respect those of our patients, even those of our patients, even those with which we might disagree. Otherwise, there is a risk that our personal values may interfere with medical judgement.”
“Letting Karl tell his story, and listening to him in a nonjudgmental way, would in itself have helped him to understand himself more…If he then chose to continue living according to a value system that conflicted with the physician's, then the physician has to accept that.”
Source: Drescher J, Fergusson A. Physician values and clinical decision making. Virtual Mentor: Ethics Journal of the American Medical Association. 2006;8(5):303-308

## Slide 27
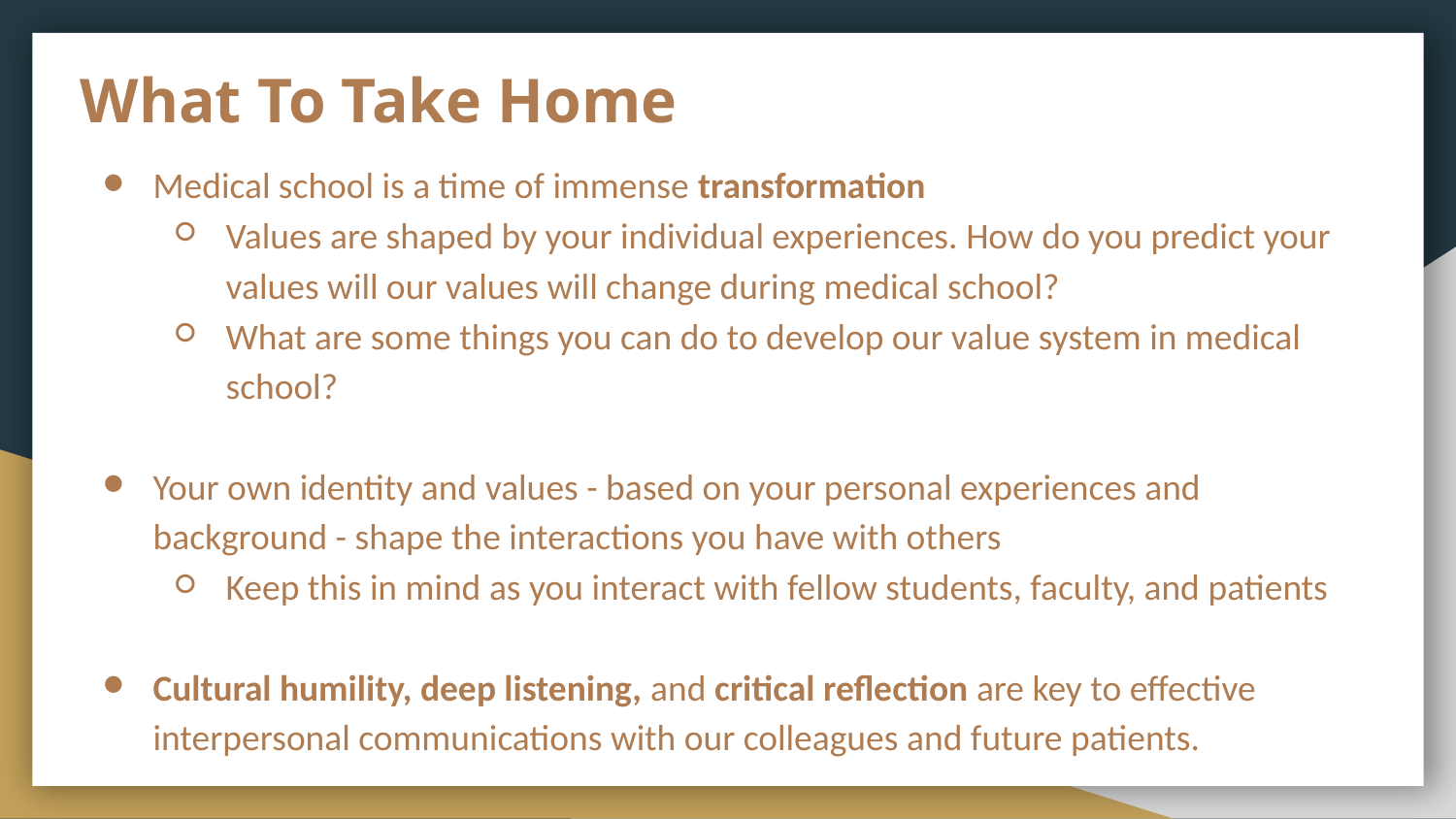

# What To Take Home
Medical school is a time of immense transformation
Values are shaped by your individual experiences. How do you predict your values will our values will change during medical school?
What are some things you can do to develop our value system in medical school?
Your own identity and values - based on your personal experiences and background - shape the interactions you have with others
Keep this in mind as you interact with fellow students, faculty, and patients
Cultural humility, deep listening, and critical reflection are key to effective interpersonal communications with our colleagues and future patients.

## Slide 28
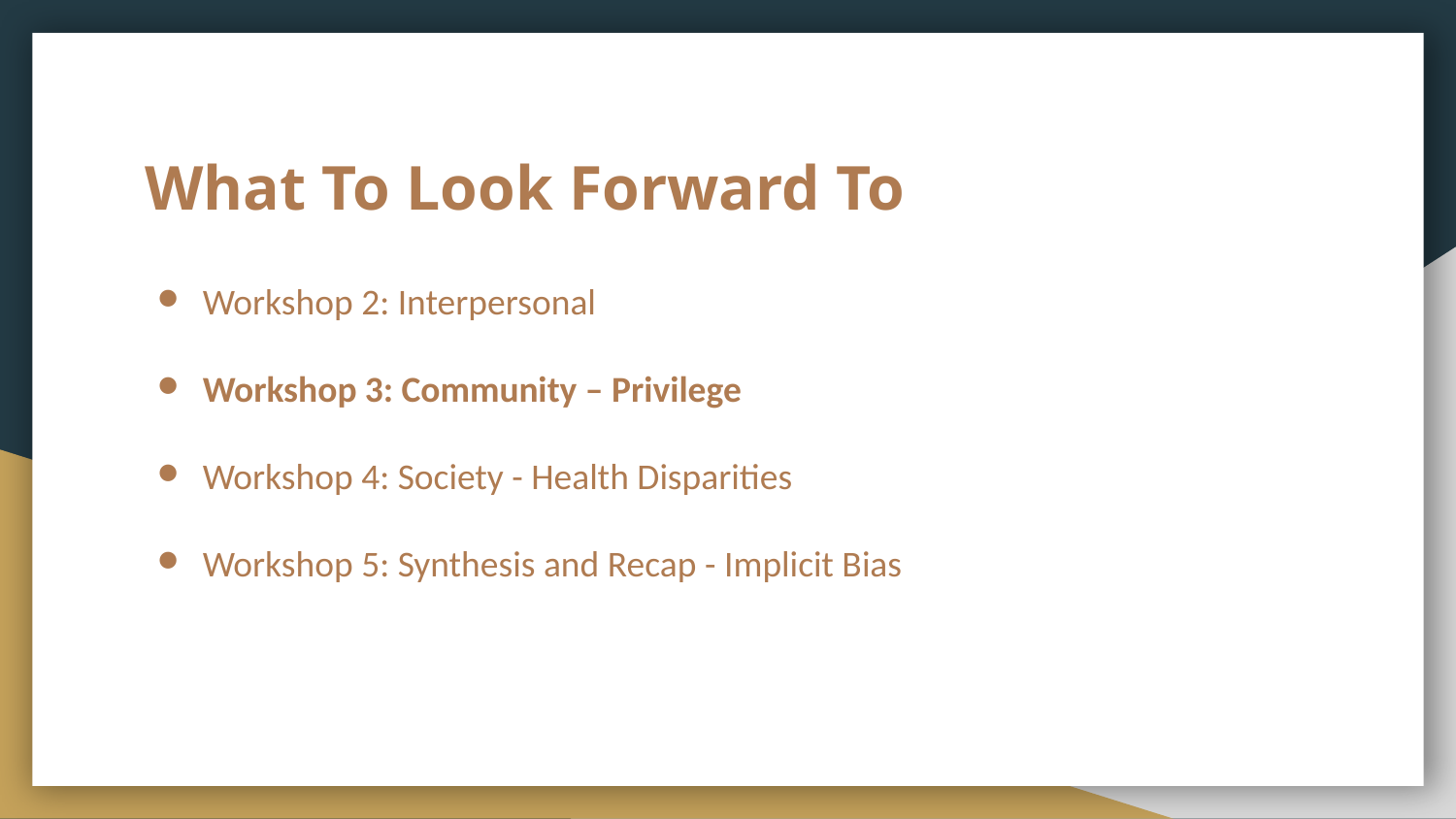

# What To Look Forward To
Workshop 2: Interpersonal
Workshop 3: Community – Privilege
Workshop 4: Society - Health Disparities
Workshop 5: Synthesis and Recap - Implicit Bias
