# Supplementary material for: Critical Consciousness as a Framework for Health Equity–Focused Peer Learning
Source: MedEdPORTAL. 2021 Apr 28;17:11145. doi: 10.15766/mep_2374-8265.11145 (PMC8079426; doi:10.15766/mep_2374-8265.11145)
Supplement: Supplementary file 1 — Workshop 1 Presentation.pptxWorkshop 1 Student Handout.docxWorkshop 2 Presentation.pptxWorkshop 2 Student Handout.docxWorkshop 3 Presentation.pptxWorkshop 3 Student Handout.docxWorkshop 4 Presentation.pptxWorkshop 5 Presentation.pptxFacilitator Orientation.pptxWorkshop 1 Facilitator Guide.docxWorkshop 2 Facilitator Guide.docxWorkshop 3 Facilitator Guide.docxWorkshop 4 Facilitator Guide.docxWorkshop 5 Facilitator Guide.docxEvaluation Tools.docx [file mep_2374-8265.11145-s001.zip › D. Workshop 2 Student Handout.docx]

**Critical Consciousness in Medicine Workshop #2
Identity and Interpersonal Relationships**

**VALUES ACTIVITY**^1^

*Answer the following questions with the words, phrases or experiences that most readily come to mind.*

What was a time that brought you great joy? Who were you with? What were you doing?

What was a time where you felt called to action? What motivated you to act? What did you do?

What are your passion areas? What do you love about them? What do they add to your life?

What was a time you had to overcome an obstacle? How did you overcome the challenge you faced?

**CASE DISCUSSION #1: Cross-Cultural Differences in Communication About a Dying Child**^2^

A 6-year-old, previously healthy boy presented with ataxia, myoclonus, and cognitive decline. His symptoms had begun 3 months earlier with poor fine motor coordination and ataxia that had progressed. He ultimately received a diagnosis of subacute sclerosing panencephalitis (SSPE), a late complication of measles infection in infancy.

SSPE is a progressive neurologic disease that has no treatment and no cure. Death is almost universal 1 to 3 years after onset, with mean survival of 18 months. The late stages include seizures, dementia, and a vegetative state.

The parents immigrated to the United States from Pakistan 8 years earlier. Both parents spoke English fluently. They had 2 other children at home. The father was at the hospital more often. The mother was at home taking care of their other 2 children.

After the diagnosis of SSPE was made, the father requested that his wife not be told of their son’s diagnosis, prognosis, or management plan, including possible upcoming procedures (gastrostomy tube, tracheotomy). The father said that the bad news was so painful to him, he wanted to shield his family and that he would break the news to his family when the time was right. “I know my wife better than anyone,” he said. In the meantime, he requested that all communication be with him only and that he would make all medical decisions.

This request caused moral distress and unrest among the medical team. One attending wrote in the chart that we had an “obligation” to ask the mother to come to the hospital so that we could tell her about her son. Others thought we should at least ask the mother what she wanted to know. There was a sense of urgency because his rapid deterioration raised concern about a more fulminant course that could mean death within months.

**CASE DISCUSSION #2: Physician Values and Decision Making**^3^

Karl is a relatively new patient in Dr. Breck’s practice. Dr. Breck knows that Karl, who is 20 years old, moved to the city from his rural hometown just over a year ago. Karl has been waiting tables and has talked about pursuing a college degree. He comes to Dr. Breck’s office complaining of a burning sensation when he urinates, but seems uncomfortable speaking about his chief symptoms.

During the course of the history, Dr. Breck asks Karl about his sexual interactions. Karl is very hesitant to speak about this, but eventually admits that he has had several unprotected homosexual encounters in the past year. Dr. Breck also asks Karl about his obvious anxiety, and Karl eventually opens up about how he left home soon after telling his family that he was homosexual. Karl states that his family was not at all supportive and that he immediately felt ostracized by his friends. He admits that much of their rejection was based on religious ideology. “I just couldn’t take their constant judgment anymore, so I decided to leave,” he says.

The physical examination leads Dr. Breck to suspect an infection, possibly a form of gonorrhea. He takes a few samples for culture to confirm his clinical suspicion and places Karl on a course of ceftriaxone and azithromycin as initial therapy. Dr. Breck schedules Karl for a follow-up visit to go over the lab results and “talk about some of the issues that might be affecting your physical, emotional, and spiritual health.”

When the results of the cultures return, Dr. Breck finds that Karl did have a gonoccocal infection with a strain that is responsive to the antibiotic therapy he prescribed. Nonetheless, Dr. Breck has his office staff confirm the follow-up appointment with Karl.

At that next appointment, Karl is relieved that his symptoms are resolving. At that point, Dr. Breck brings up his concerns about Karl’s sexual behavior and speaks about blood testing for HIV and hepatitis C. Karl seems hesitant to have any blood tests, stating that “no one I have been with would have any of those diseases.”

Dr. Breck then brings up the issue of Karl’s family and their response to his sexuality. “I understand that your experimentation with homosexuality has caused a major rift between you and your family,” Dr. Breck says, suggesting that his parents’ reaction was most likely “one of shock at seeing a child lose his way.” Dr. Breck then recommends that Karl see Dr. Talbert, a local psychotherapist and personal friend of Dr. Breck’s, well known for his work in “conversion therapy”—counseling interventions focused on eliminating homosexual thoughts and behaviors.

^1^ Adapted from Princeton University Pace Center for Civic Engagement. *Field Guide to Service*. Princeton, NJ: Trustees of Princeton University; 2017.

^2^ Excerpted from Cochran D, Saleem S, Khowaja-Punjwani S, Lantos JD. Cross cultural differences about communication in a dying child. *Pediatrics*. 2017;140(5):e20170690.

^3^ Excerpted from Drescher J, Fergusson A. Physician values and clinical decision making. *Virtual Mentor: Ethics Journal of the American Medical Association*. 2006;8(5):303-308
