# Supplementary material for: Critical Consciousness as a Framework for Health Equity–Focused Peer Learning
Source: MedEdPORTAL. 2021 Apr 28;17:11145. doi: 10.15766/mep_2374-8265.11145 (PMC8079426; doi:10.15766/mep_2374-8265.11145)
Supplement: Supplementary file 1 — Workshop 1 Presentation.pptxWorkshop 1 Student Handout.docxWorkshop 2 Presentation.pptxWorkshop 2 Student Handout.docxWorkshop 3 Presentation.pptxWorkshop 3 Student Handout.docxWorkshop 4 Presentation.pptxWorkshop 5 Presentation.pptxFacilitator Orientation.pptxWorkshop 1 Facilitator Guide.docxWorkshop 2 Facilitator Guide.docxWorkshop 3 Facilitator Guide.docxWorkshop 4 Facilitator Guide.docxWorkshop 5 Facilitator Guide.docxEvaluation Tools.docx [file mep_2374-8265.11145-s001.zip › E. Workshop 3 Presentation.pptx]

## Slide 1
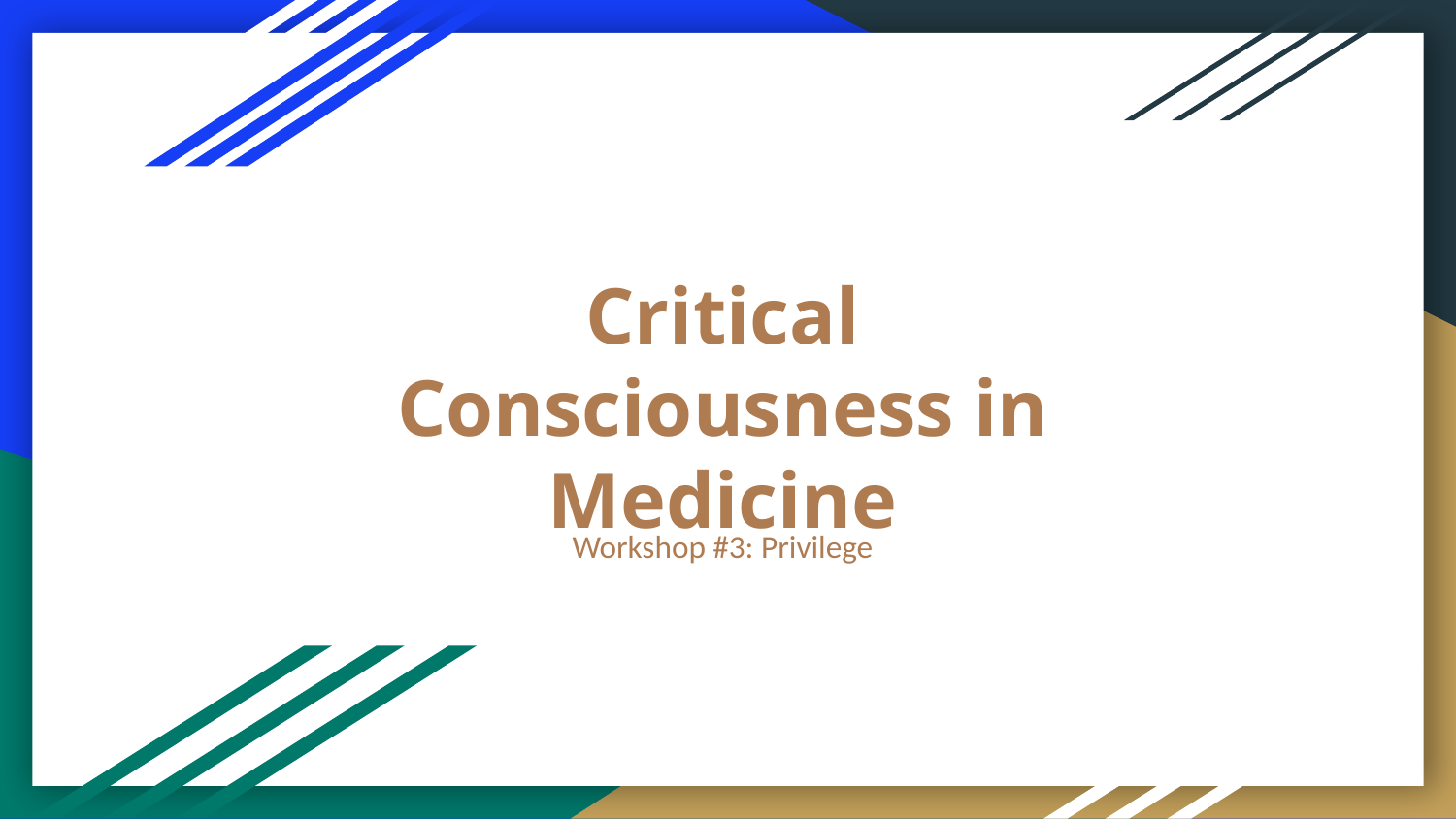

# Critical Consciousness in Medicine
Workshop #3: Privilege

## Slide 2
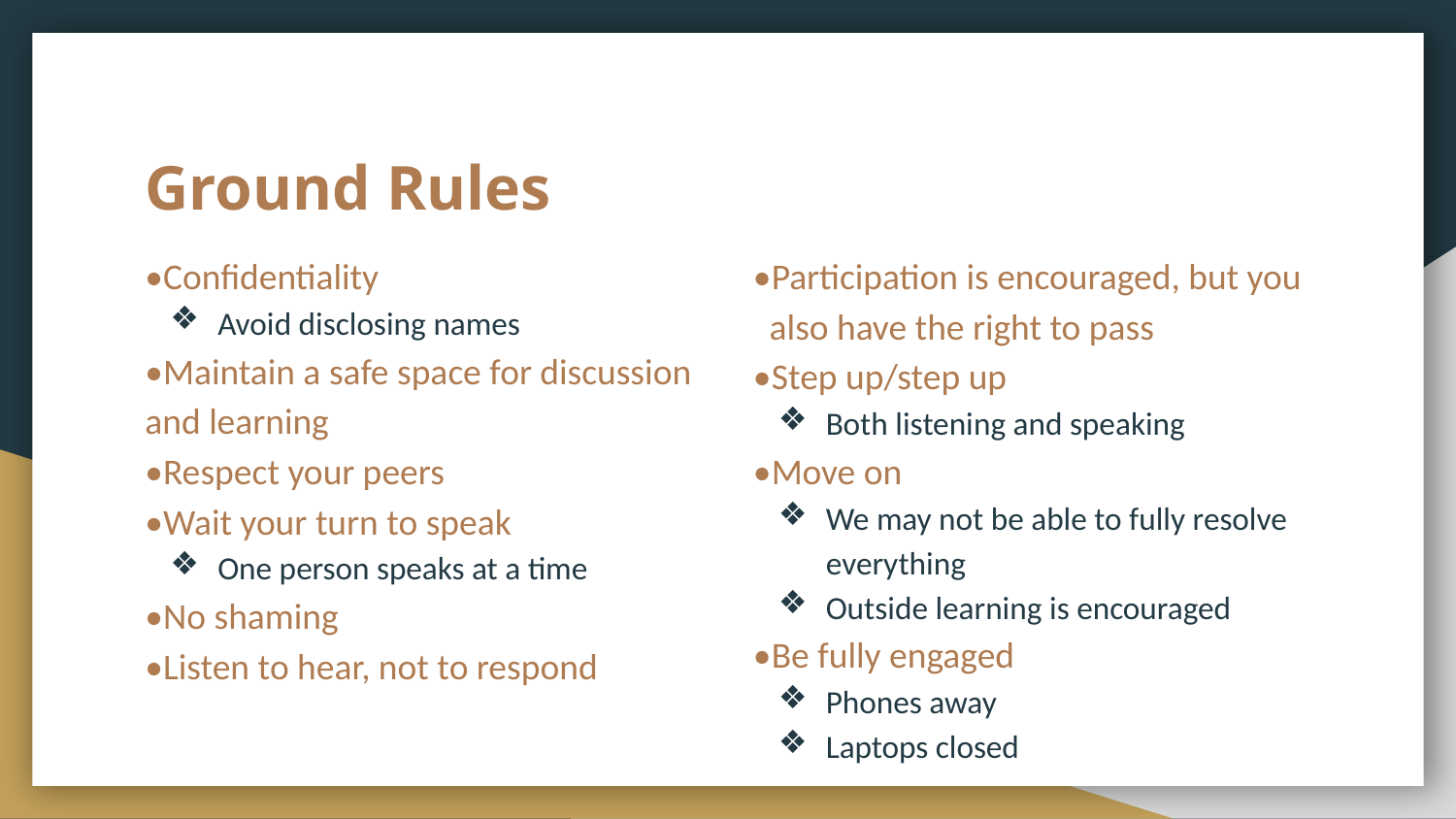

# Ground Rules
•Confidentiality
Avoid disclosing names
•Maintain a safe space for discussion and learning
•Respect your peers
•Wait your turn to speak
One person speaks at a time
•No shaming
•Listen to hear, not to respond
•Participation is encouraged, but you also have the right to pass
•Step up/step up
Both listening and speaking
•Move on
We may not be able to fully resolve everything
Outside learning is encouraged
•Be fully engaged
Phones away
Laptops closed

## Slide 3
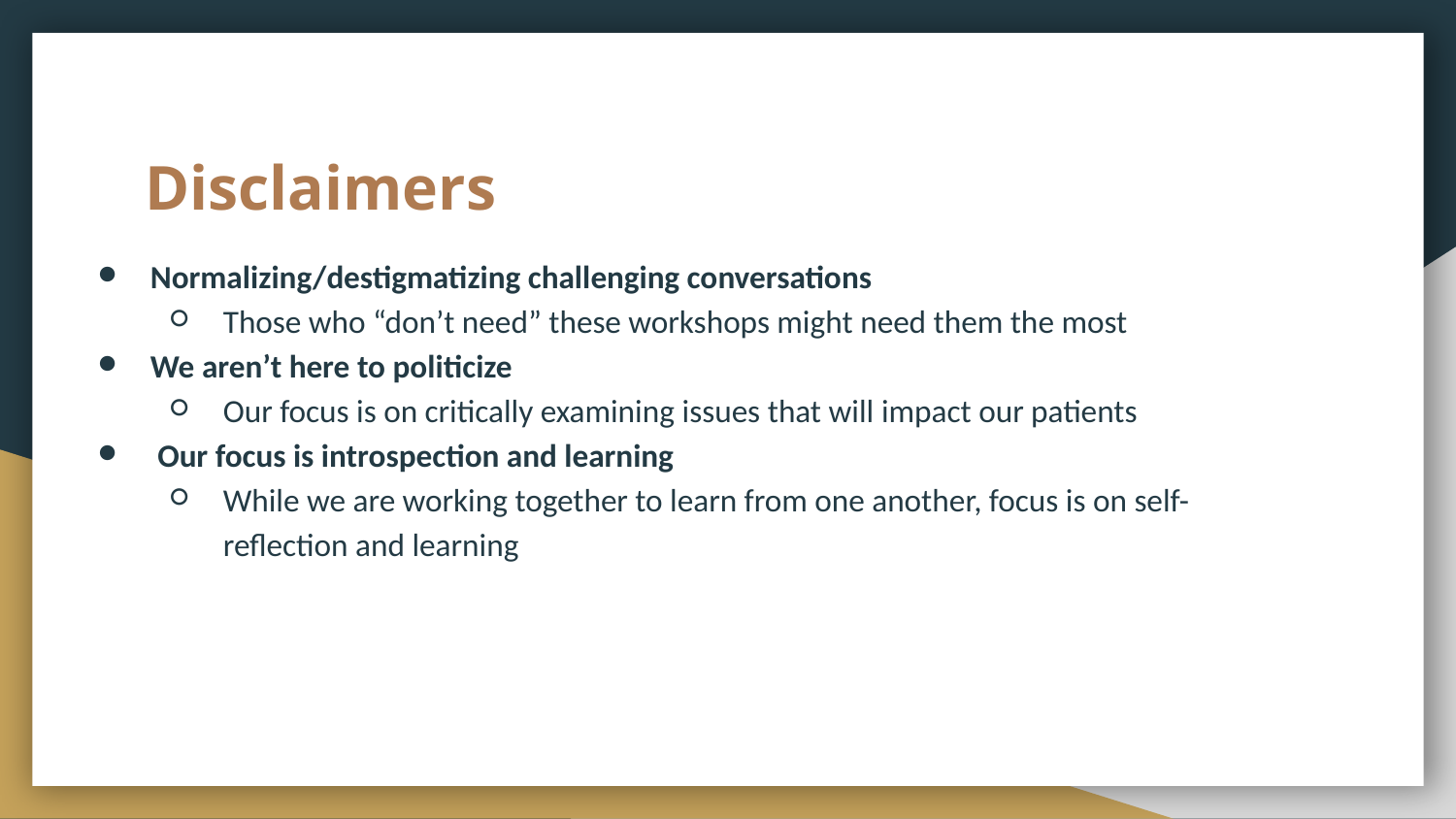

# Disclaimers
Normalizing/destigmatizing challenging conversations
Those who “don’t need” these workshops might need them the most
We aren’t here to politicize
Our focus is on critically examining issues that will impact our patients
 Our focus is introspection and learning
While we are working together to learn from one another, focus is on self-reflection and learning

## Slide 4
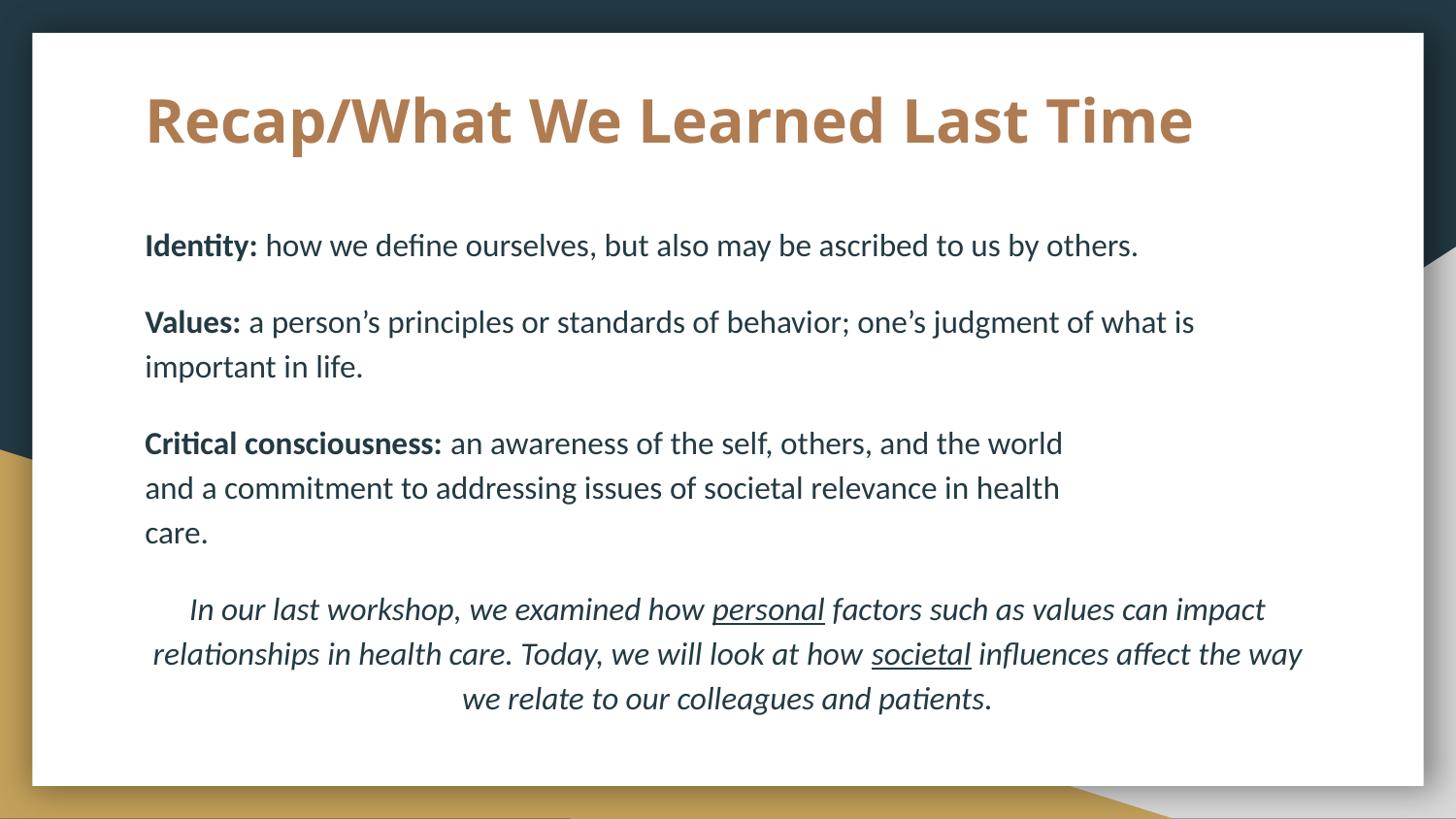

# Recap/What We Learned Last Time
Identity: how we define ourselves, but also may be ascribed to us by others.
Values: a person’s principles or standards of behavior; one’s judgment of what is important in life.
Critical consciousness: an awareness of the self, others, and the world
and a commitment to addressing issues of societal relevance in health
care.
In our last workshop, we examined how personal factors such as values can impact relationships in health care. Today, we will look at how societal influences affect the way we relate to our colleagues and patients.

## Slide 5
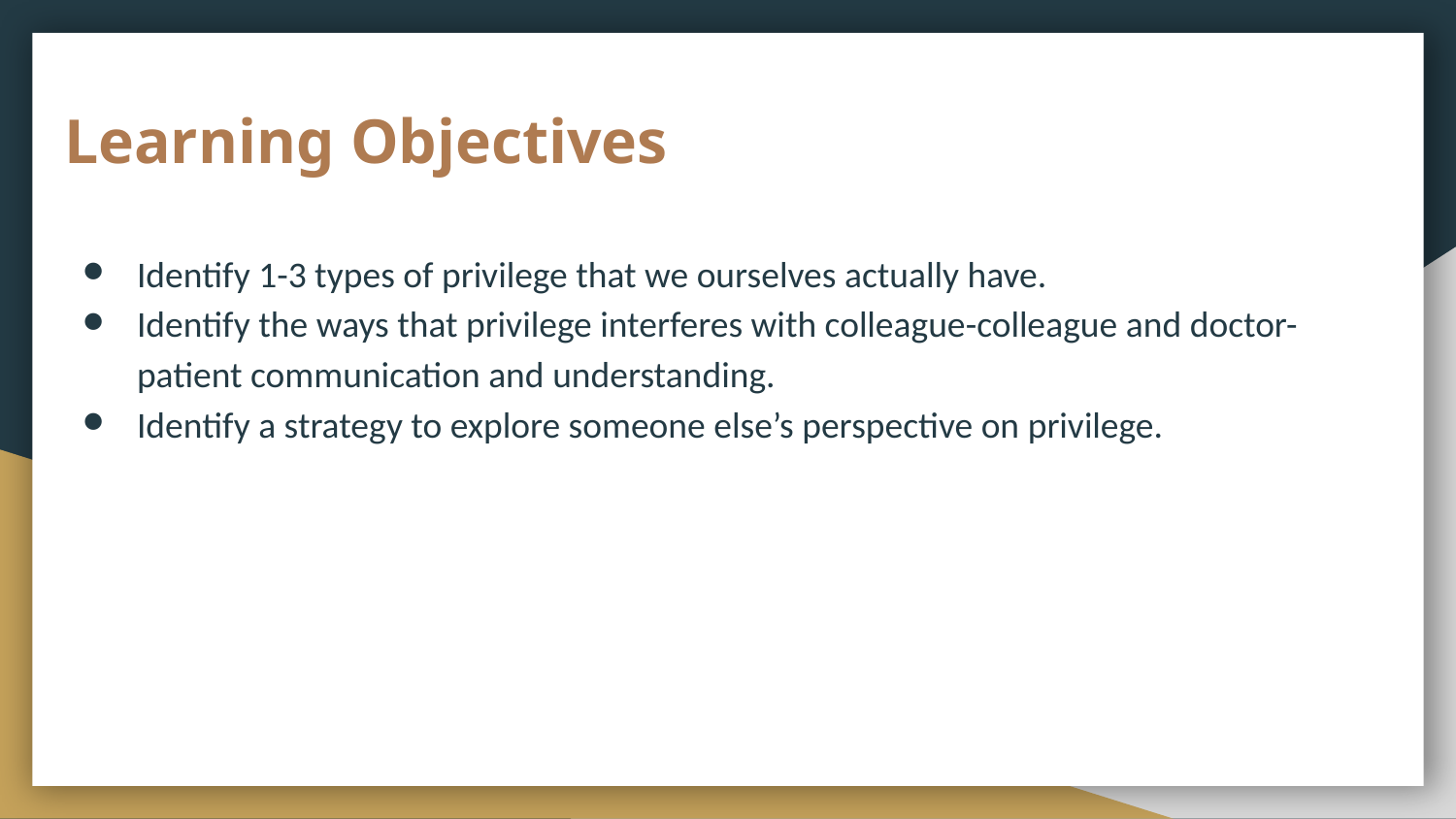

# Learning Objectives
Identify 1-3 types of privilege that we ourselves actually have.
Identify the ways that privilege interferes with colleague-colleague and doctor-patient communication and understanding.
Identify a strategy to explore someone else’s perspective on privilege.

## Slide 6
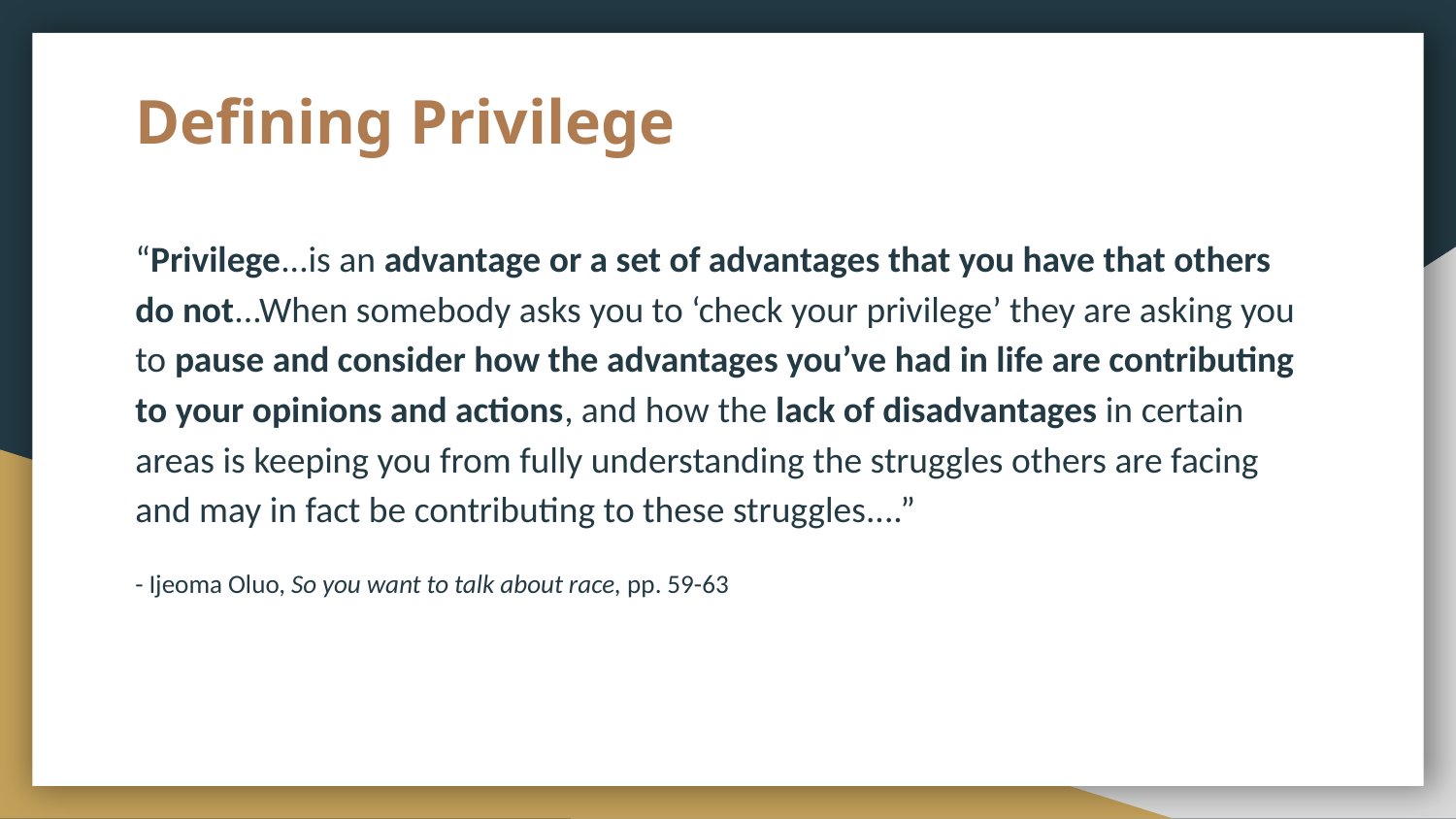

# Defining Privilege
“Privilege...is an advantage or a set of advantages that you have that others do not...When somebody asks you to ‘check your privilege’ they are asking you to pause and consider how the advantages you’ve had in life are contributing to your opinions and actions, and how the lack of disadvantages in certain areas is keeping you from fully understanding the struggles others are facing and may in fact be contributing to these struggles....”
- Ijeoma Oluo, So you want to talk about race, pp. 59-63

## Slide 7
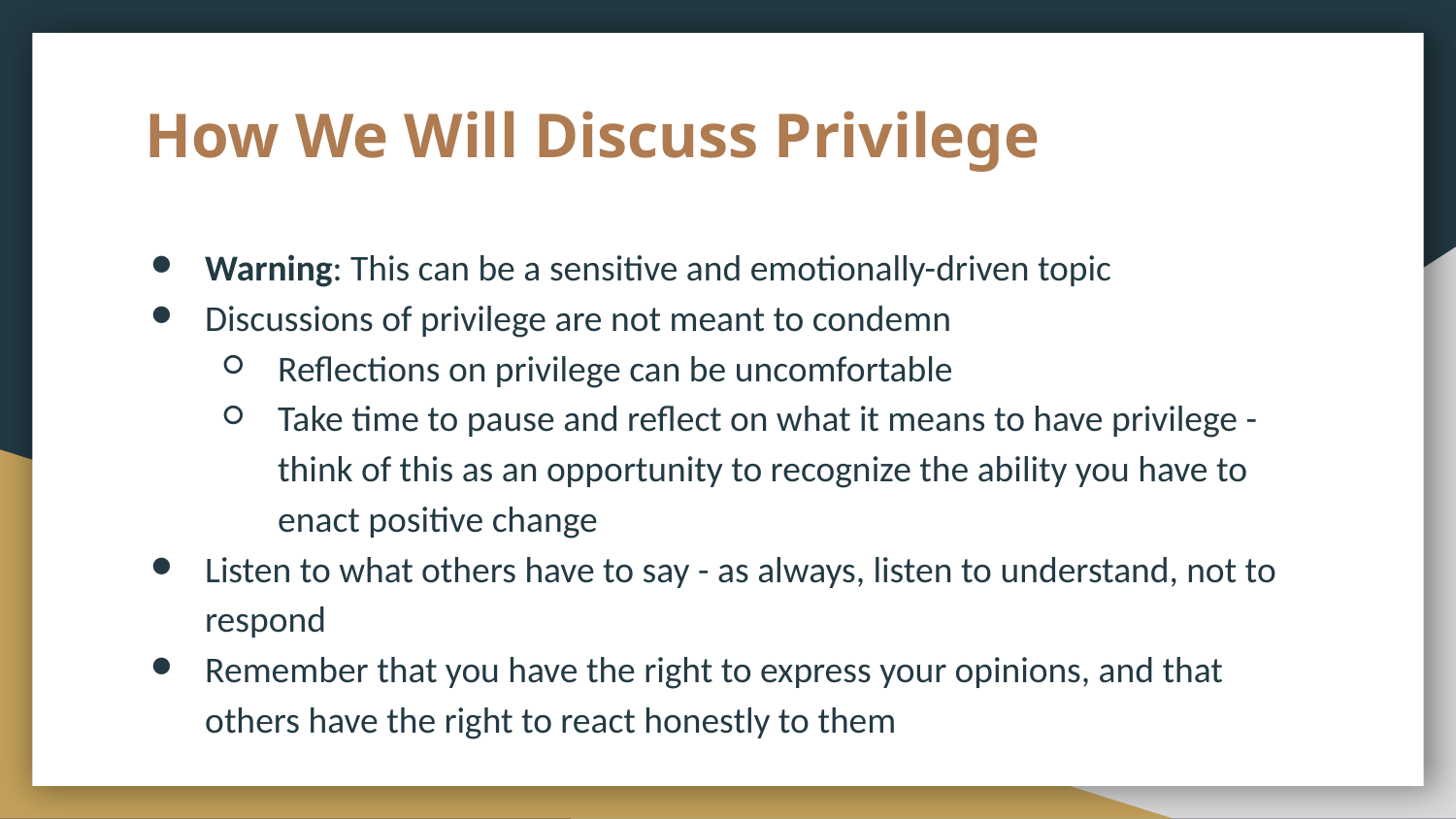

# How We Will Discuss Privilege
Warning: This can be a sensitive and emotionally-driven topic
Discussions of privilege are not meant to condemn
Reflections on privilege can be uncomfortable
Take time to pause and reflect on what it means to have privilege - think of this as an opportunity to recognize the ability you have to enact positive change
Listen to what others have to say - as always, listen to understand, not to respond
Remember that you have the right to express your opinions, and that others have the right to react honestly to them

## Slide 8
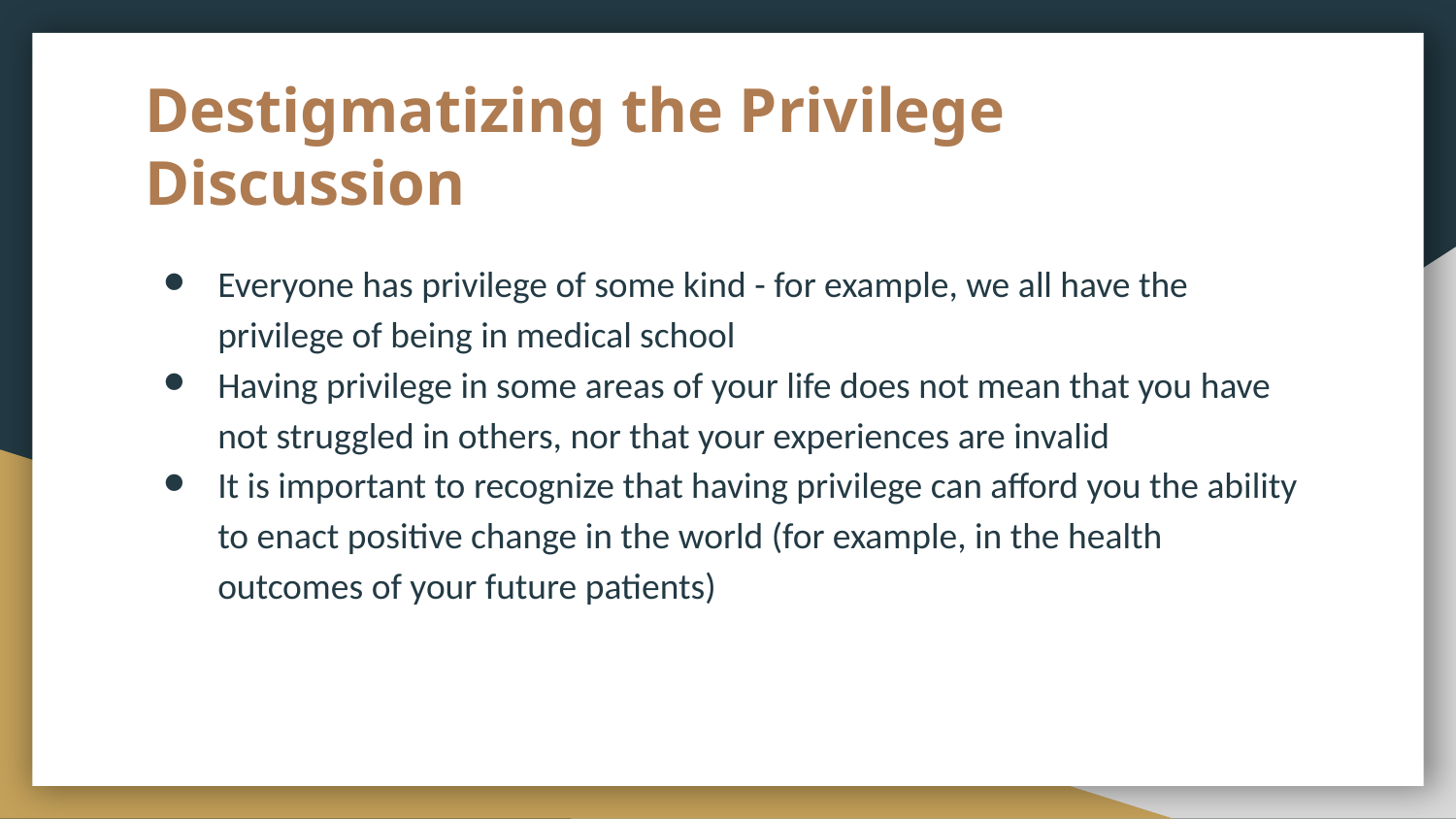

# Destigmatizing the Privilege Discussion
Everyone has privilege of some kind - for example, we all have the privilege of being in medical school
Having privilege in some areas of your life does not mean that you have not struggled in others, nor that your experiences are invalid
It is important to recognize that having privilege can afford you the ability to enact positive change in the world (for example, in the health outcomes of your future patients)

## Slide 9
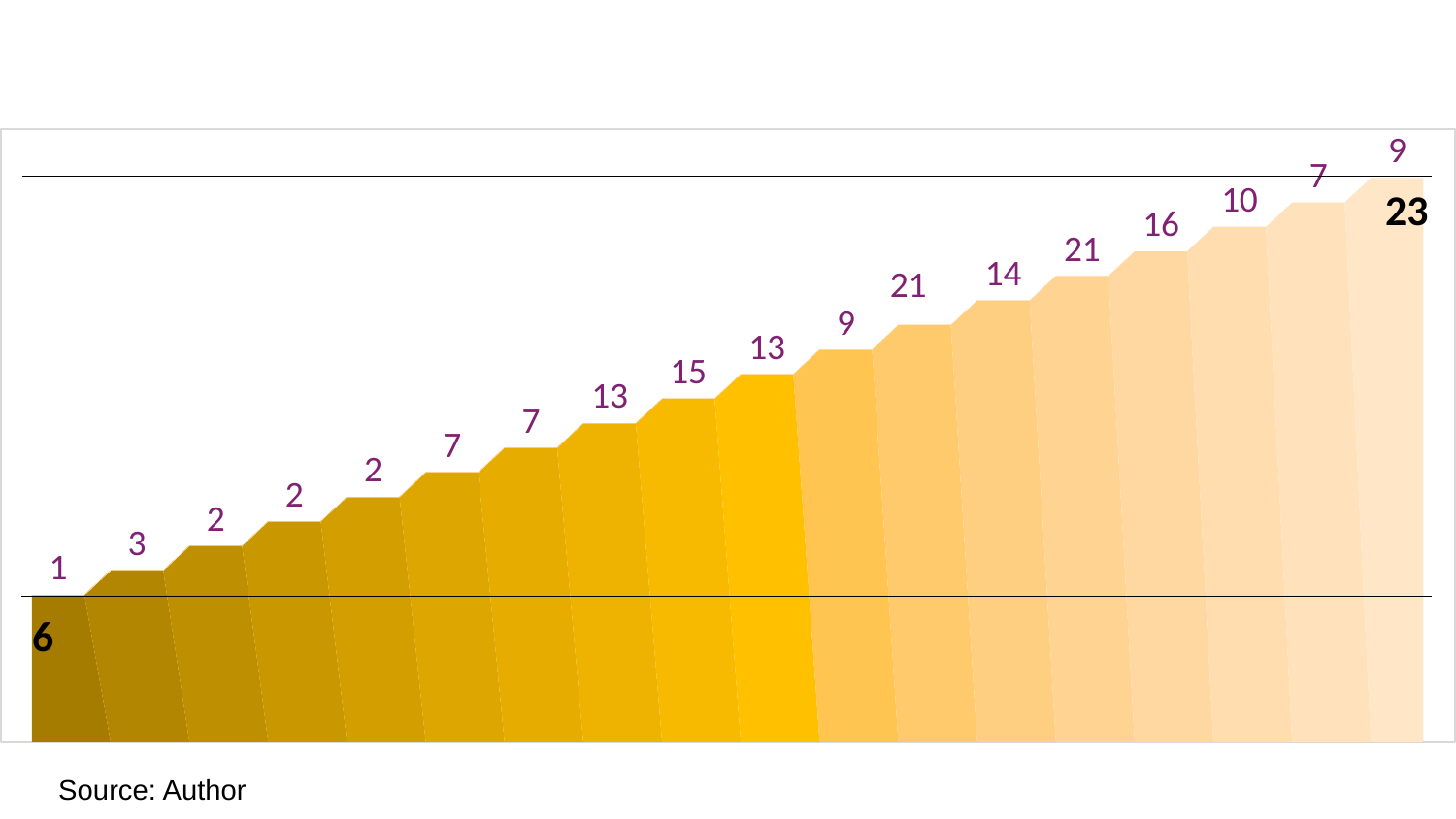

### Chart
| Category | | | | | | | | | | | | | | | | | | | |
|---|---|---|---|---|---|---|---|---|---|---|---|---|---|---|---|---|---|---|---|
| 0 | 6.0 | None | None | None | None | None | None | None | None | None | None | None | None | None | None | None | None | None | None |
| 0.5 | 6.0 | None | None | None | None | None | None | None | None | None | None | None | None | None | None | None | None | None | 6.0 |
| 1 | 6.0 | None | None | None | None | None | None | None | None | None | None | None | None | None | None | None | None | None | None |
| 1 | None | 7.0 | None | None | None | None | None | None | None | None | None | None | None | None | None | None | None | None | None |
| 2.5 | None | 7.0 | None | None | None | None | None | None | None | None | None | None | None | None | None | None | None | None | 7.0 |
| 4 | None | 7.0 | None | None | None | None | None | None | None | None | None | None | None | None | None | None | None | None | None |
| 4 | None | None | 8.0 | None | None | None | None | None | None | None | None | None | None | None | None | None | None | None | None |
| 5 | None | None | 8.0 | None | None | None | None | None | None | None | None | None | None | None | None | None | None | None | 8.0 |
| 6 | None | None | 8.0 | None | None | None | None | None | None | None | None | None | None | None | None | None | None | None | None |
| 6 | None | None | None | 9.0 | None | None | None | None | None | None | None | None | None | None | None | None | None | None | None |
| 7 | None | None | None | 9.0 | None | None | None | None | None | None | None | None | None | None | None | None | None | None | 9.0 |
| 8 | None | None | None | 9.0 | None | None | None | None | None | None | None | None | None | None | None | None | None | None | None |
| 8 | None | None | None | None | 10.0 | None | None | None | None | None | None | None | None | None | None | None | None | None | None |
| 9 | None | None | None | None | 10.0 | None | None | None | None | None | None | None | None | None | None | None | None | None | 10.0 |
| 10 | None | None | None | None | 10.0 | None | None | None | None | None | None | None | None | None | None | None | None | None | None |
| 10 | None | None | None | None | None | 11.0 | None | None | None | None | None | None | None | None | None | None | None | None | None |
| 13.5 | None | None | None | None | None | 11.0 | None | None | None | None | None | None | None | None | None | None | None | None | 11.0 |
| 17 | None | None | None | None | None | 11.0 | None | None | None | None | None | None | None | None | None | None | None | None | None |
| 17 | None | None | None | None | None | None | 12.0 | None | None | None | None | None | None | None | None | None | None | None | None |
| 20.5 | None | None | None | None | None | None | 12.0 | None | None | None | None | None | None | None | None | None | None | None | 12.0 |
| 24 | None | None | None | None | None | None | 12.0 | None | None | None | None | None | None | None | None | None | None | None | None |
| 24 | None | None | None | None | None | None | None | 13.0 | None | None | None | None | None | None | None | None | None | None | None |
| 30.5 | None | None | None | None | None | None | None | 13.0 | None | None | None | None | None | None | None | None | None | None | 13.0 |
| 37 | None | None | None | None | None | None | None | 13.0 | None | None | None | None | None | None | None | None | None | None | None |
| 37 | None | None | None | None | None | None | None | None | 14.0 | None | None | None | None | None | None | None | None | None | None |
| 44.5 | None | None | None | None | None | None | None | None | 14.0 | None | None | None | None | None | None | None | None | None | 14.0 |
| 52 | None | None | None | None | None | None | None | None | 14.0 | None | None | None | None | None | None | None | None | None | None |
| 52 | None | None | None | None | None | None | None | None | None | 15.0 | None | None | None | None | None | None | None | None | None |
| 58.5 | None | None | None | None | None | None | None | None | None | 15.0 | None | None | None | None | None | None | None | None | 15.0 |
| 65 | None | None | None | None | None | None | None | None | None | 15.0 | None | None | None | None | None | None | None | None | None |
| 65 | None | None | None | None | None | None | None | None | None | None | 16.0 | None | None | None | None | None | None | None | None |
| 69.5 | None | None | None | None | None | None | None | None | None | None | 16.0 | None | None | None | None | None | None | None | 16.0 |
| 74 | None | None | None | None | None | None | None | None | None | None | 16.0 | None | None | None | None | None | None | None | None |
| 74 | None | None | None | None | None | None | None | None | None | None | None | 17.0 | None | None | None | None | None | None | None |
| 84.5 | None | None | None | None | None | None | None | None | None | None | None | 17.0 | None | None | None | None | None | None | 17.0 |
| 95 | None | None | None | None | None | None | None | None | None | None | None | 17.0 | None | None | None | None | None | None | None |
| 95 | None | None | None | None | None | None | None | None | None | None | None | None | 18.0 | None | None | None | None | None | None |
| 102 | None | None | None | None | None | None | None | None | None | None | None | None | 18.0 | None | None | None | None | None | 18.0 |
| 109 | None | None | None | None | None | None | None | None | None | None | None | None | 18.0 | None | None | None | None | None | None |
| 109 | None | None | None | None | None | None | None | None | None | None | None | None | None | 19.0 | None | None | None | None | None |
| 119.5 | None | None | None | None | None | None | None | None | None | None | None | None | None | 19.0 | None | None | None | None | 19.0 |
| 130 | None | None | None | None | None | None | None | None | None | None | None | None | None | 19.0 | None | None | None | None | None |
| 130 | None | None | None | None | None | None | None | None | None | None | None | None | None | None | 20.0 | None | None | None | None |
| 138 | None | None | None | None | None | None | None | None | None | None | None | None | None | None | 20.0 | None | None | None | 20.0 |
| 146 | None | None | None | None | None | None | None | None | None | None | None | None | None | None | 20.0 | None | None | None | None |
| 146 | None | None | None | None | None | None | None | None | None | None | None | None | None | None | None | 21.0 | None | None | None |
| 151 | None | None | None | None | None | None | None | None | None | None | None | None | None | None | None | 21.0 | None | None | 21.0 |
| 156 | None | None | None | None | None | None | None | None | None | None | None | None | None | None | None | 21.0 | None | None | None |
| 156 | None | None | None | None | None | None | None | None | None | None | None | None | None | None | None | None | 22.0 | None | None |
| 159.5 | None | None | None | None | None | None | None | None | None | None | None | None | None | None | None | None | 22.0 | None | 22.0 |
| 163 | None | None | None | None | None | None | None | None | None | None | None | None | None | None | None | None | 22.0 | None | None |
| 163 | None | None | None | None | None | None | None | None | None | None | None | None | None | None | None | None | None | 23.0 | None |
| 167.5 | None | None | None | None | None | None | None | None | None | None | None | None | None | None | None | None | None | 23.0 | 23.0 |
| 172 | None | None | None | None | None | None | None | None | None | None | None | None | None | None | None | None | None | 23.0 | None |23
6
23
6
17
Source: Author

## Slide 10
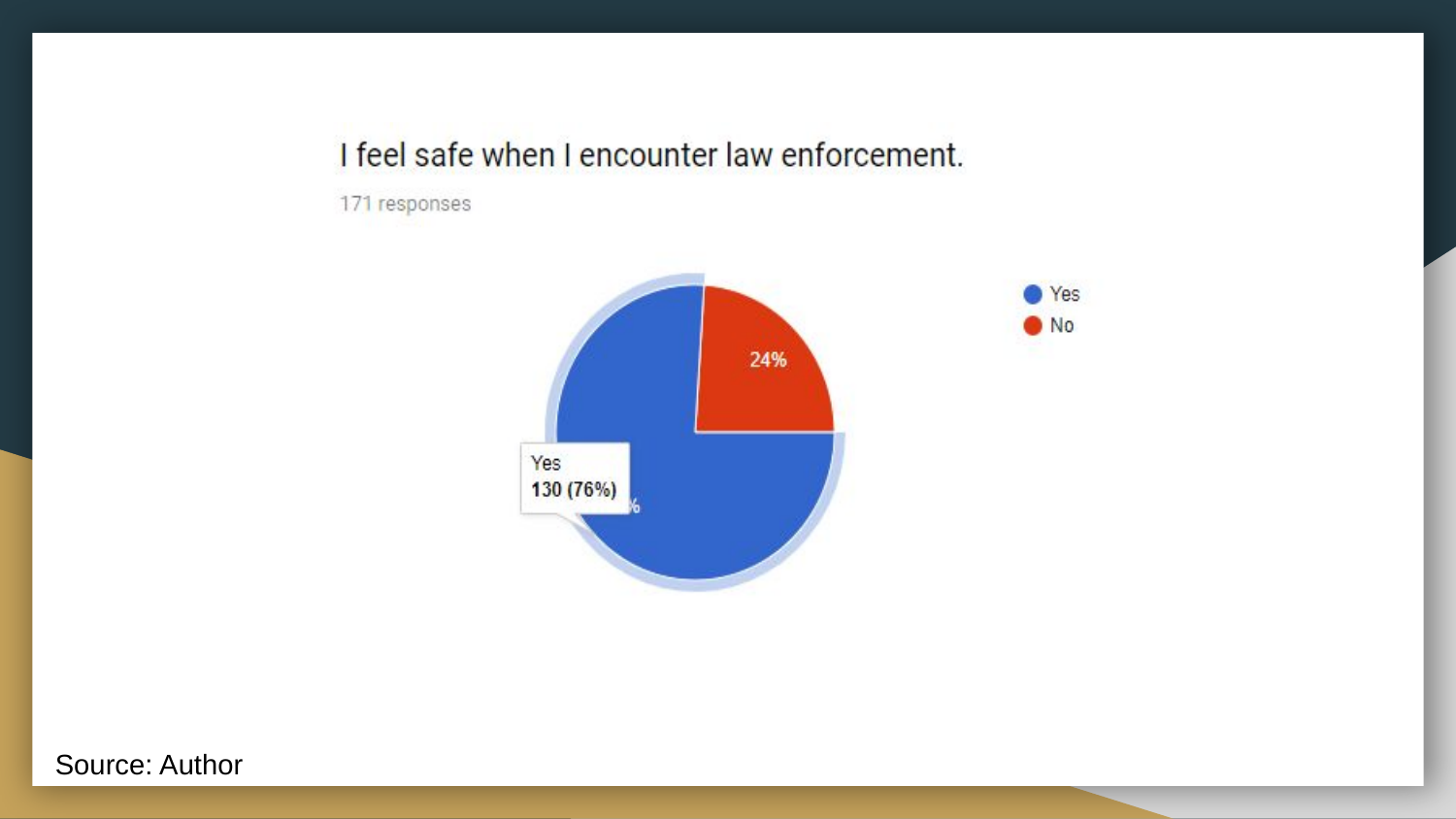

Source: Author

## Slide 11
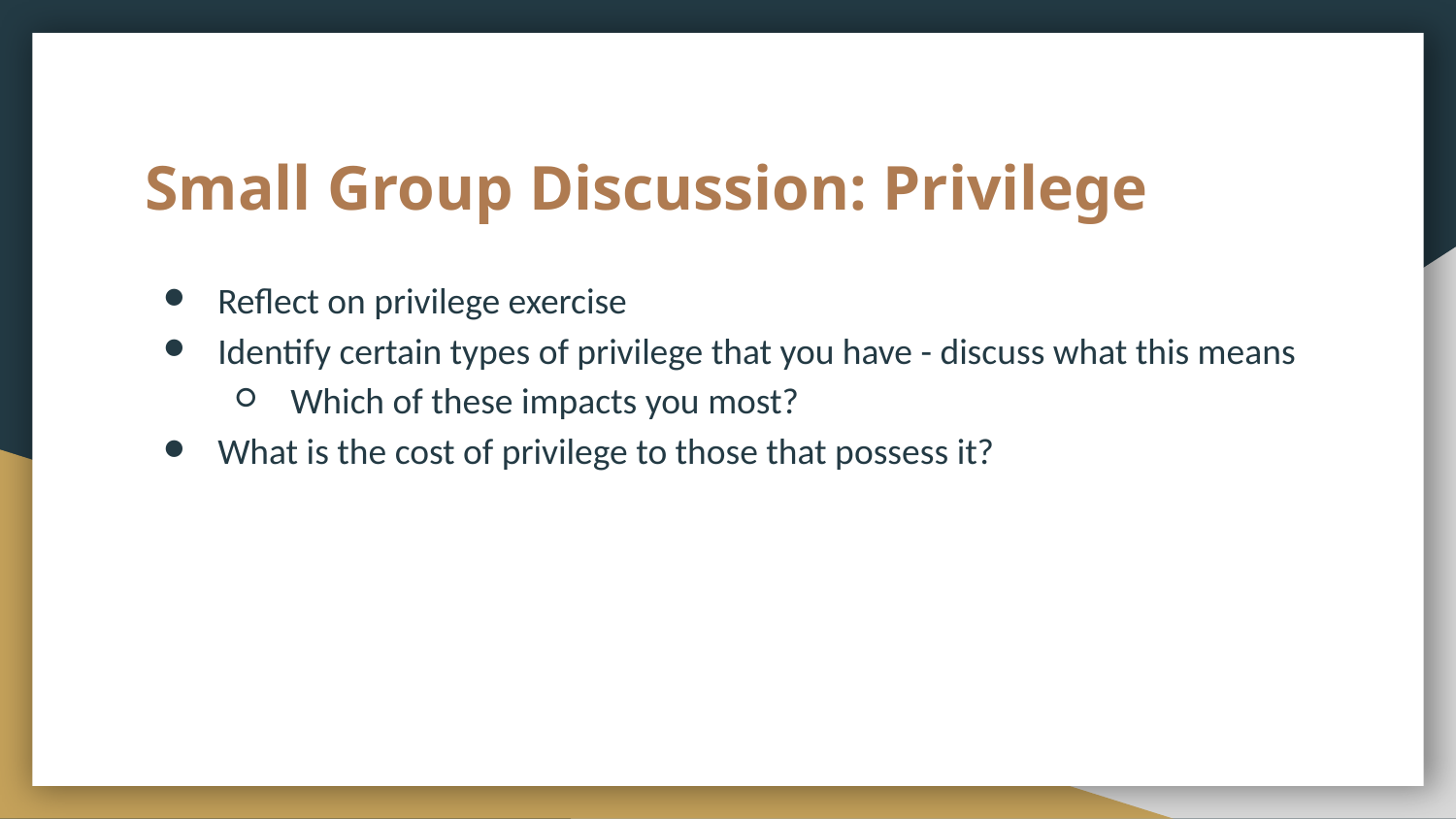

# Small Group Discussion: Privilege
Reflect on privilege exercise
Identify certain types of privilege that you have - discuss what this means
Which of these impacts you most?
What is the cost of privilege to those that possess it?

## Slide 12
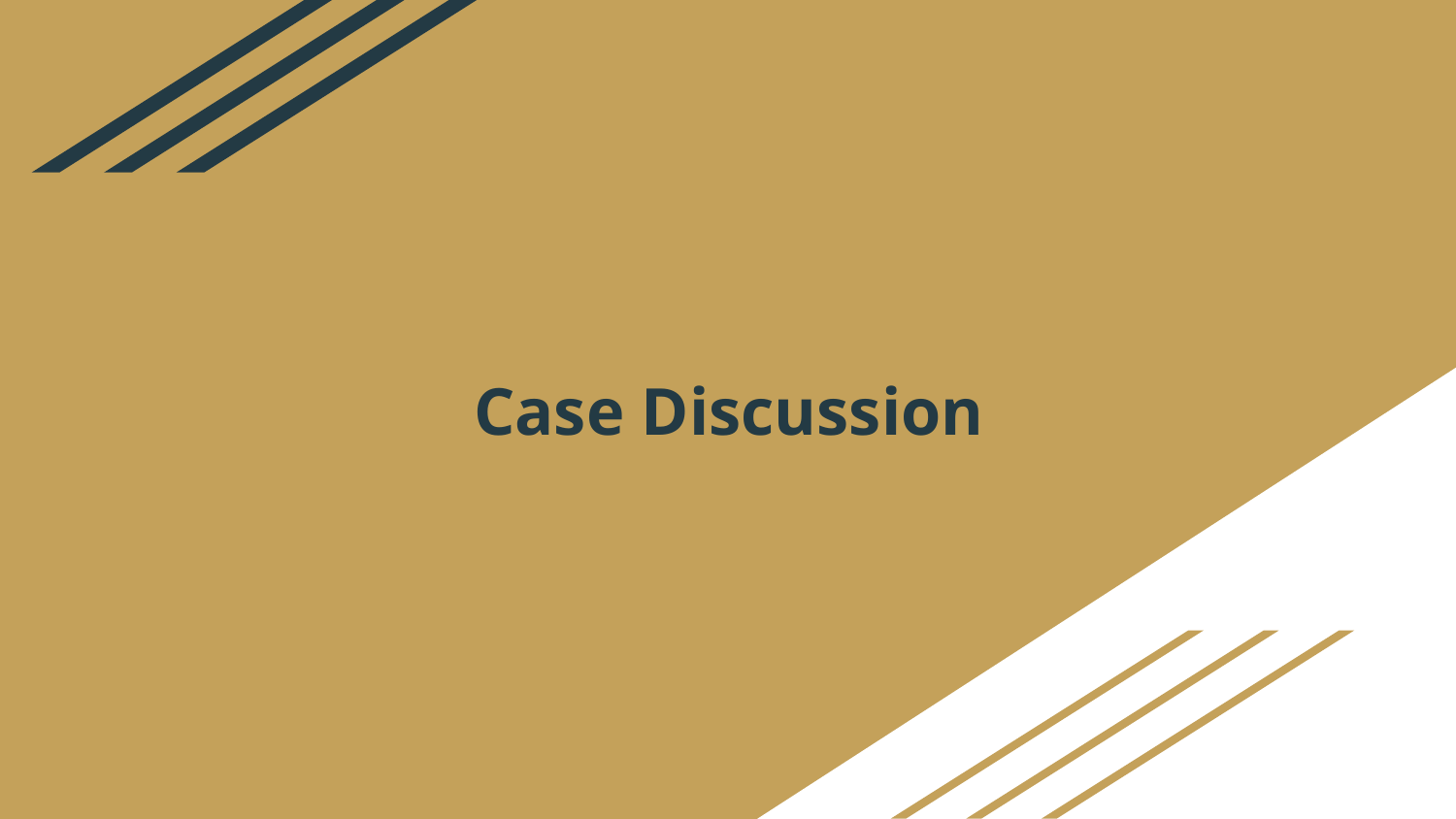

# Case Discussion

## Slide 13
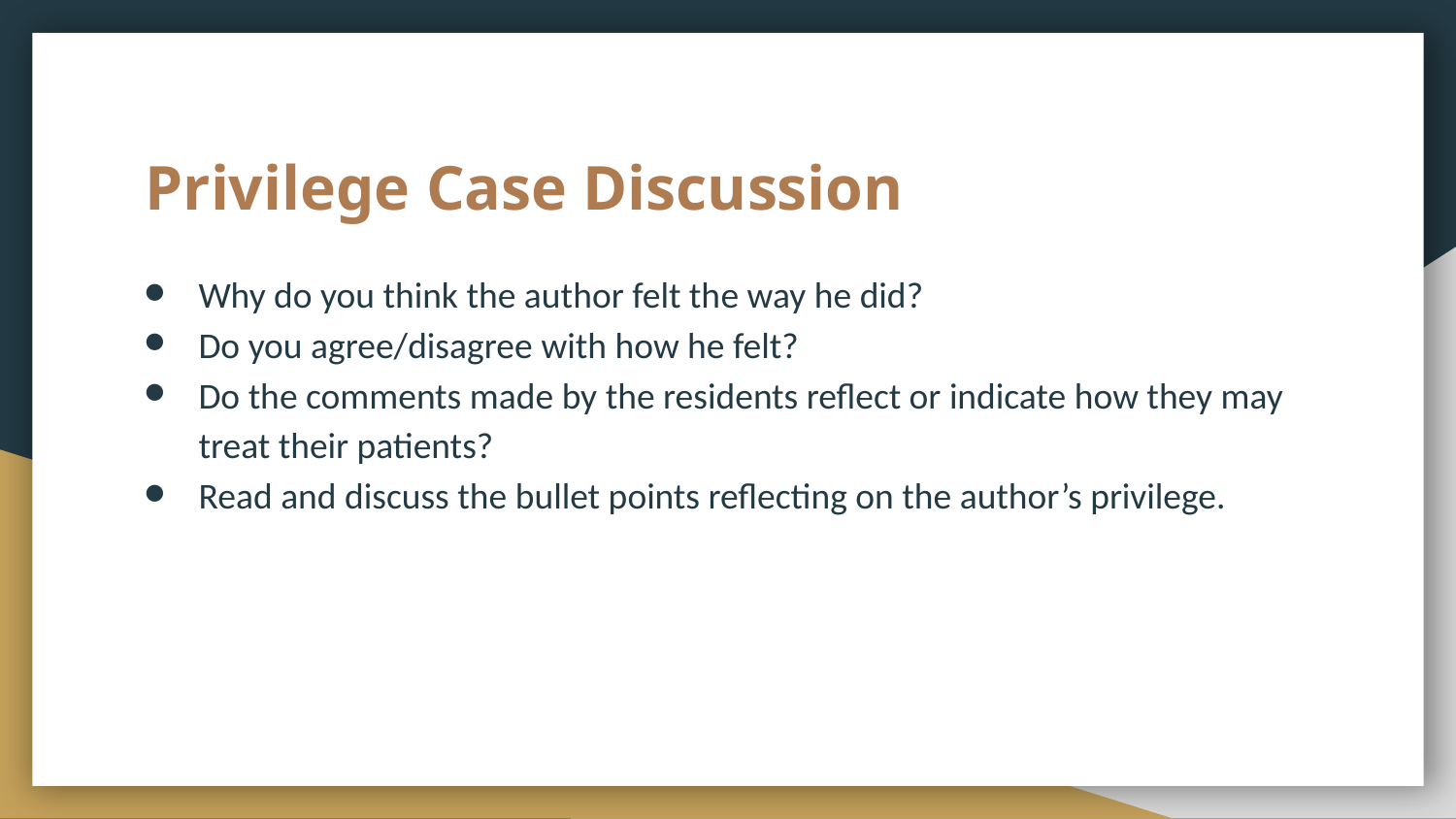

# Privilege Case Discussion
Why do you think the author felt the way he did?
Do you agree/disagree with how he felt?
Do the comments made by the residents reflect or indicate how they may treat their patients?
Read and discuss the bullet points reflecting on the author’s privilege.

## Slide 14
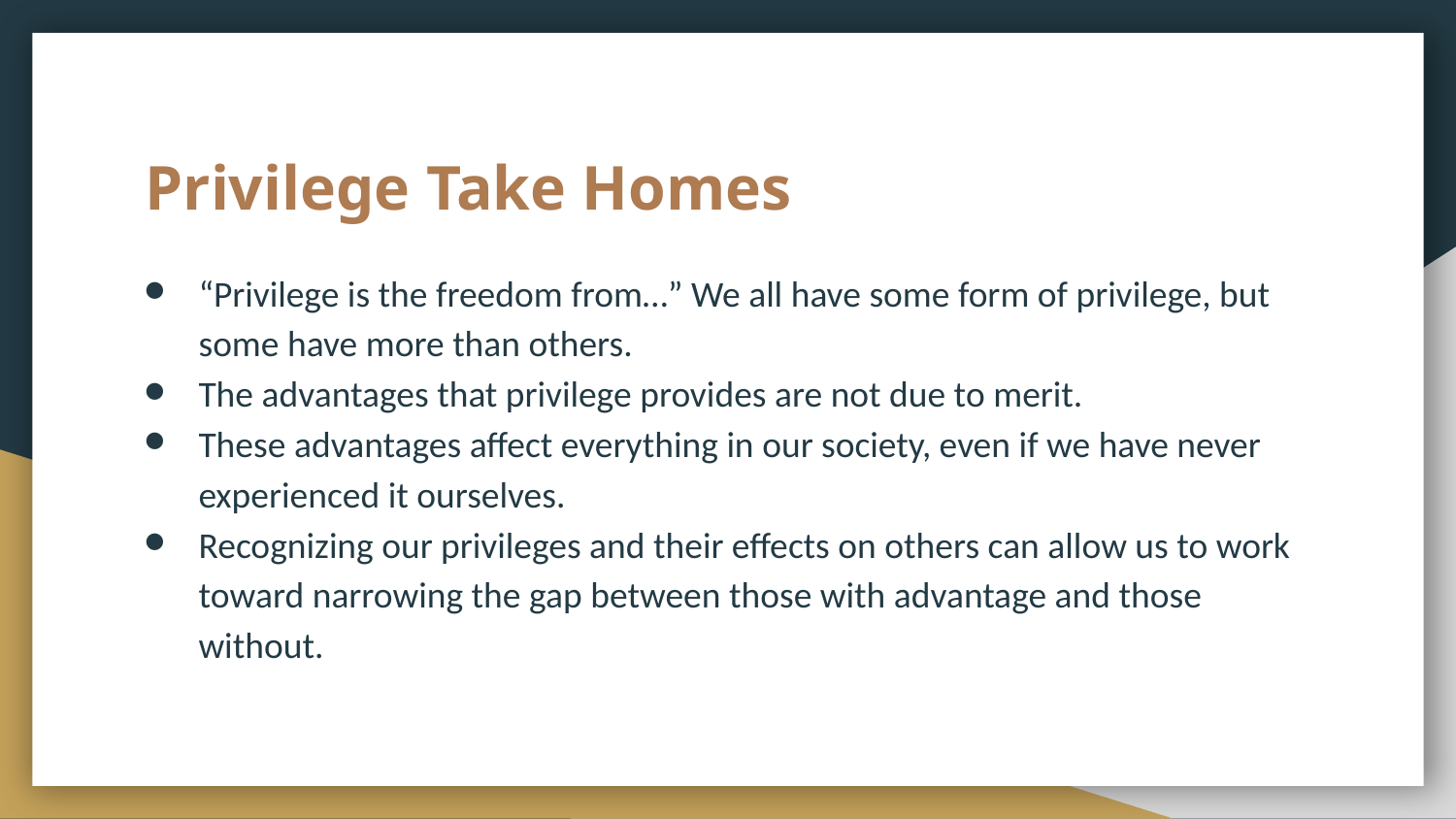

# Privilege Take Homes
“Privilege is the freedom from…” We all have some form of privilege, but some have more than others.
The advantages that privilege provides are not due to merit.
These advantages affect everything in our society, even if we have never experienced it ourselves.
Recognizing our privileges and their effects on others can allow us to work toward narrowing the gap between those with advantage and those without.

## Slide 15
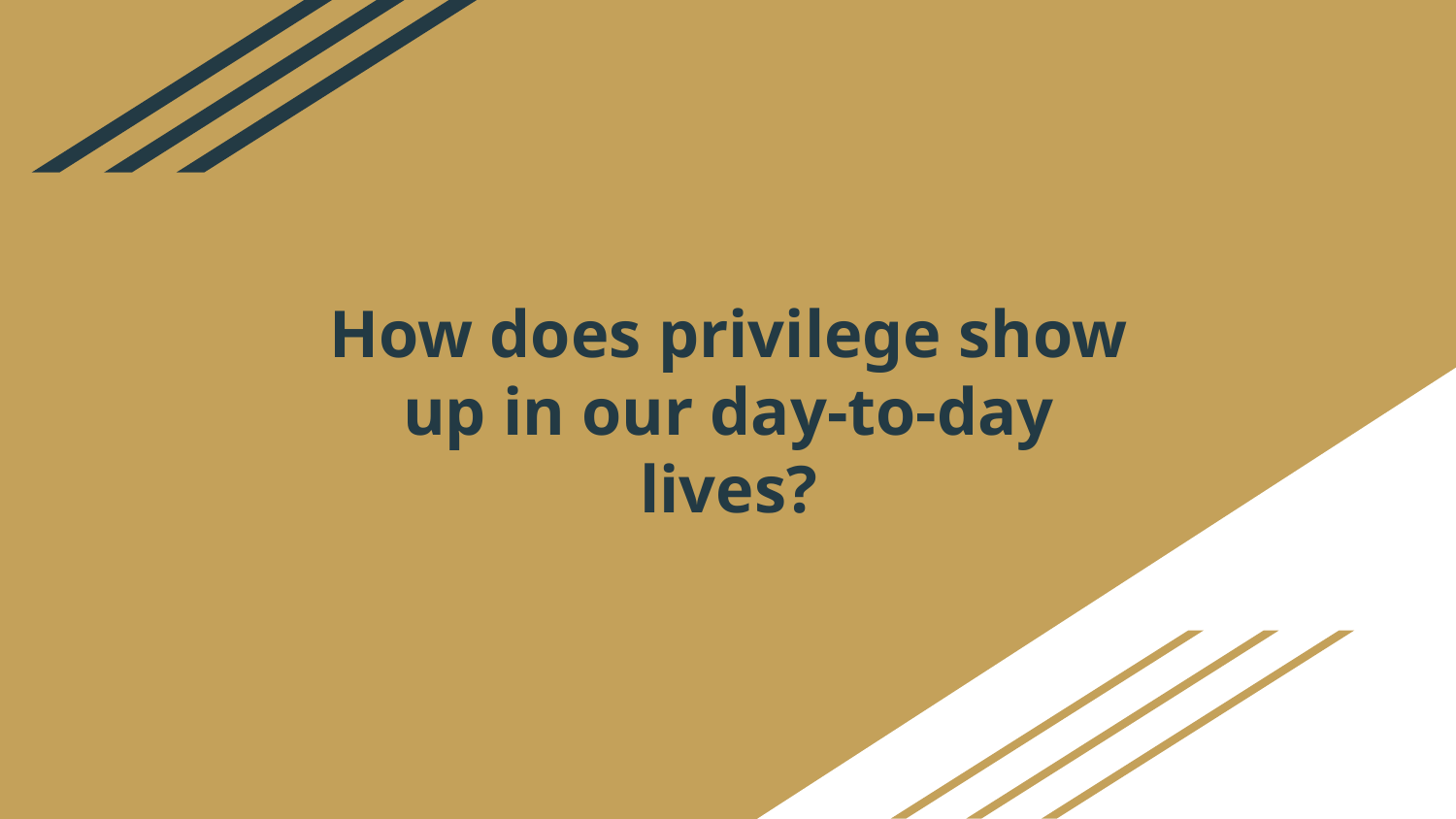

# How does privilege show up in our day-to-day lives?

## Slide 16
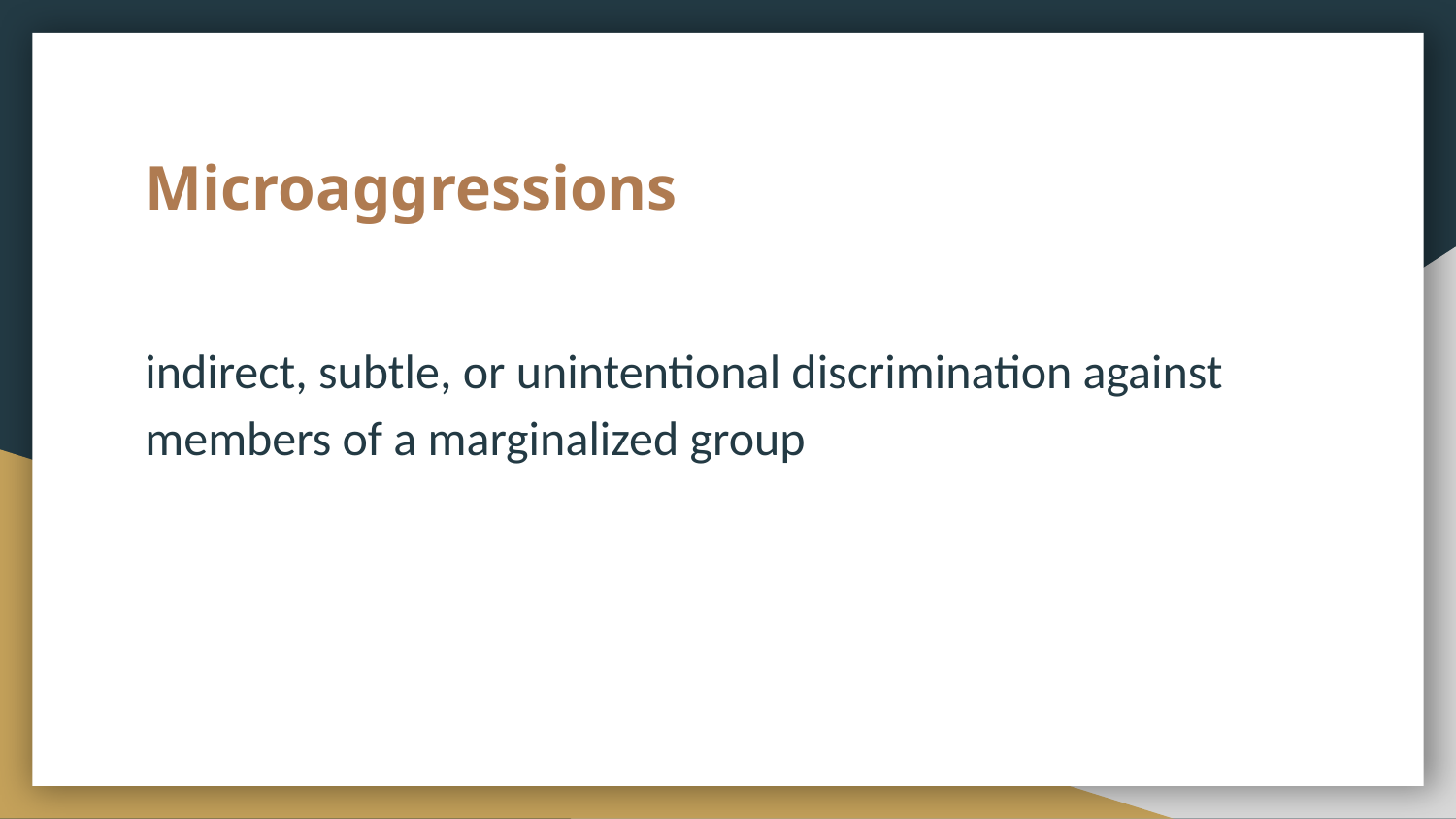

# Microaggressions
indirect, subtle, or unintentional discrimination against members of a marginalized group

## Slide 17
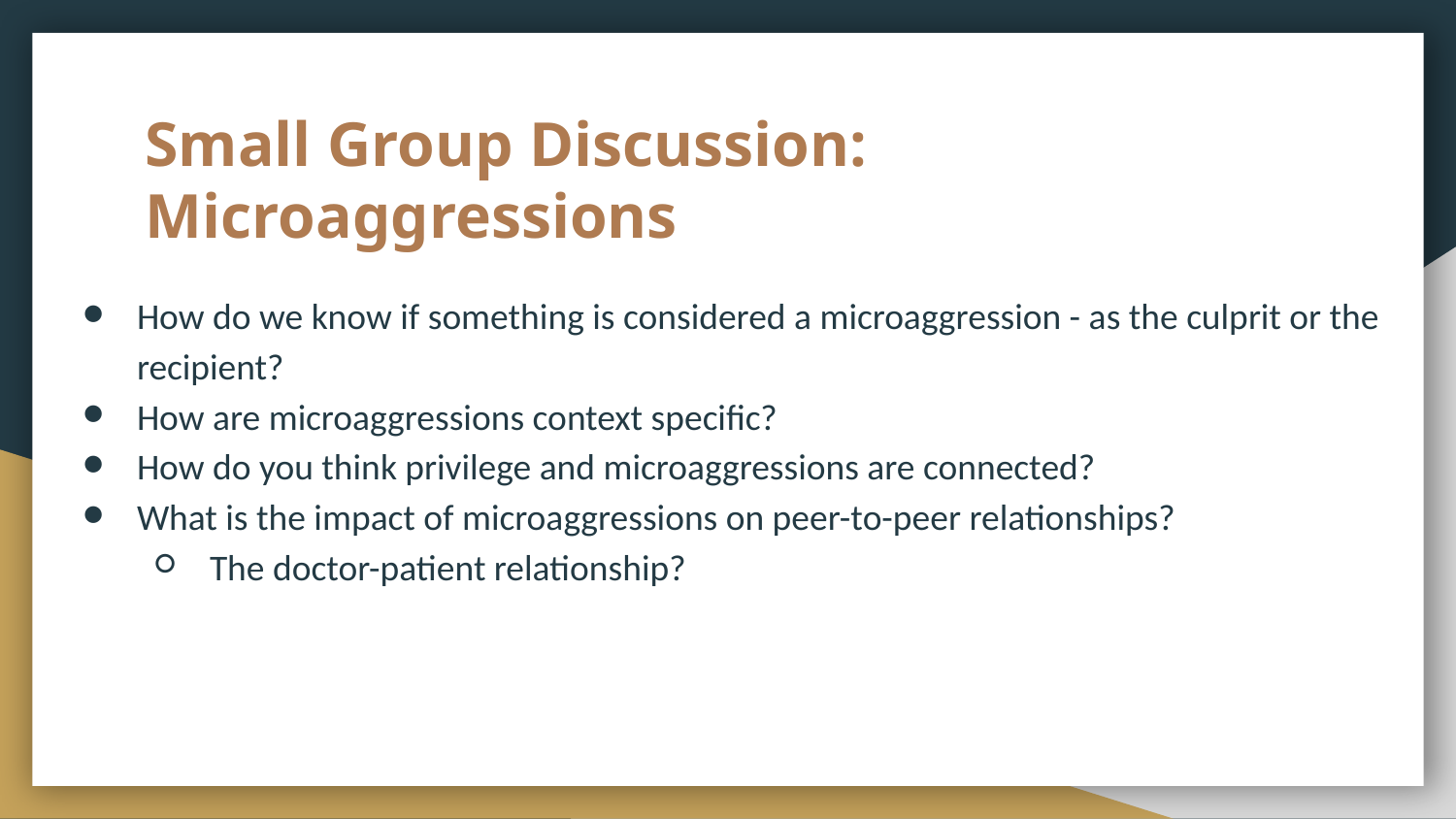

# Small Group Discussion: Microaggressions
How do we know if something is considered a microaggression - as the culprit or the recipient?
How are microaggressions context specific?
How do you think privilege and microaggressions are connected?
What is the impact of microaggressions on peer-to-peer relationships?
The doctor-patient relationship?

## Slide 18
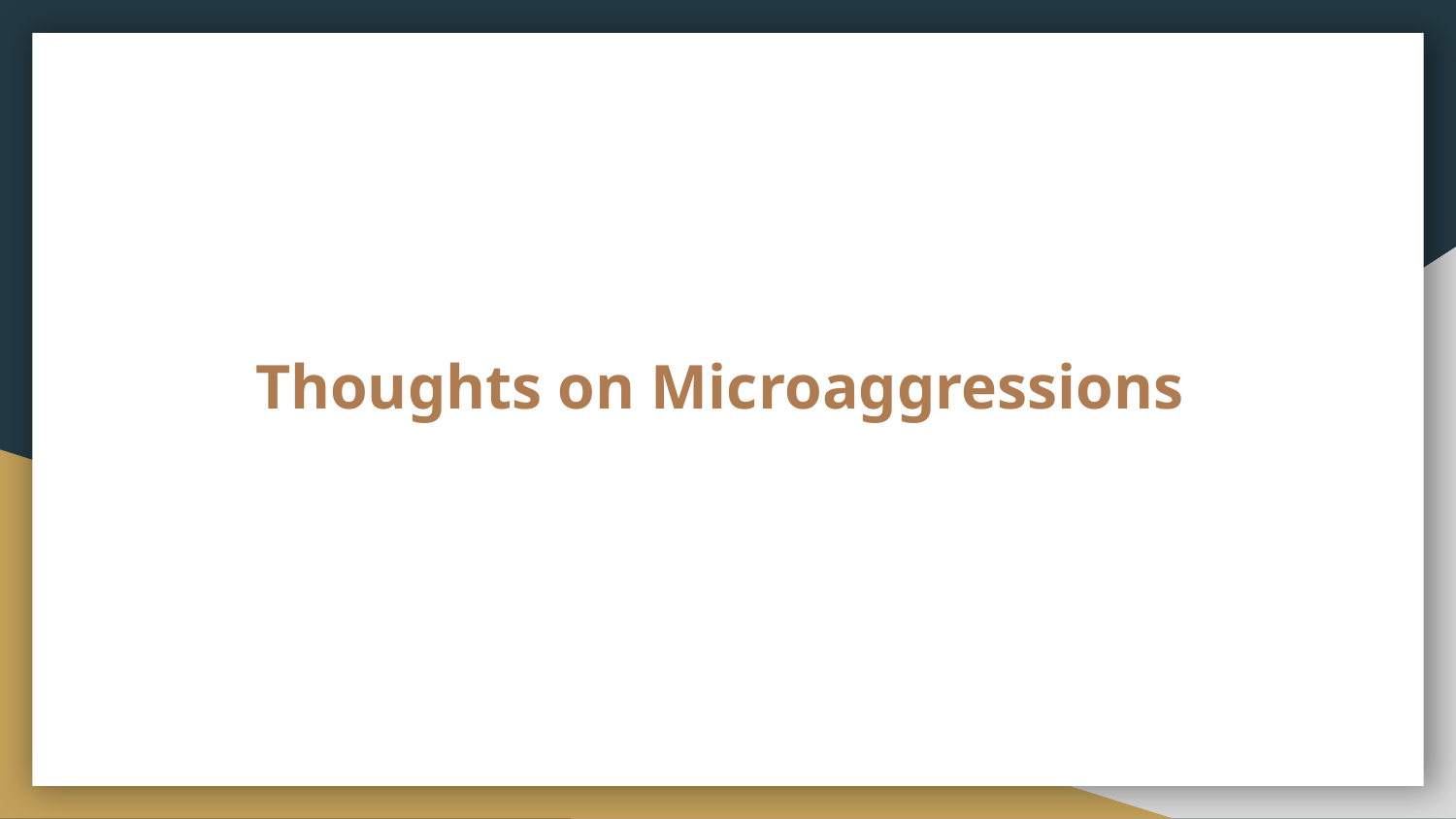

# Thoughts on Microaggressions

## Slide 19
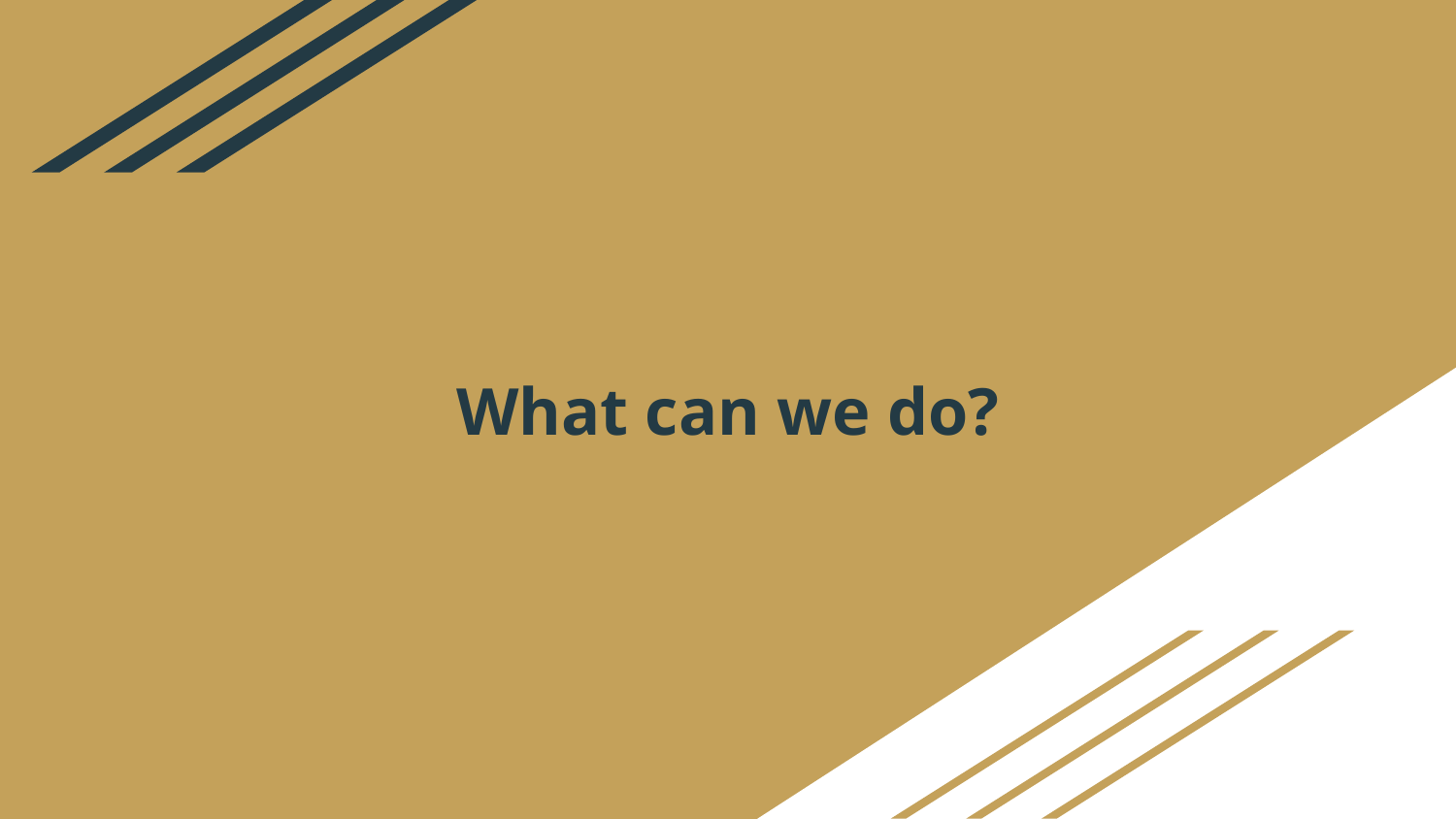

# What can we do?

## Slide 20
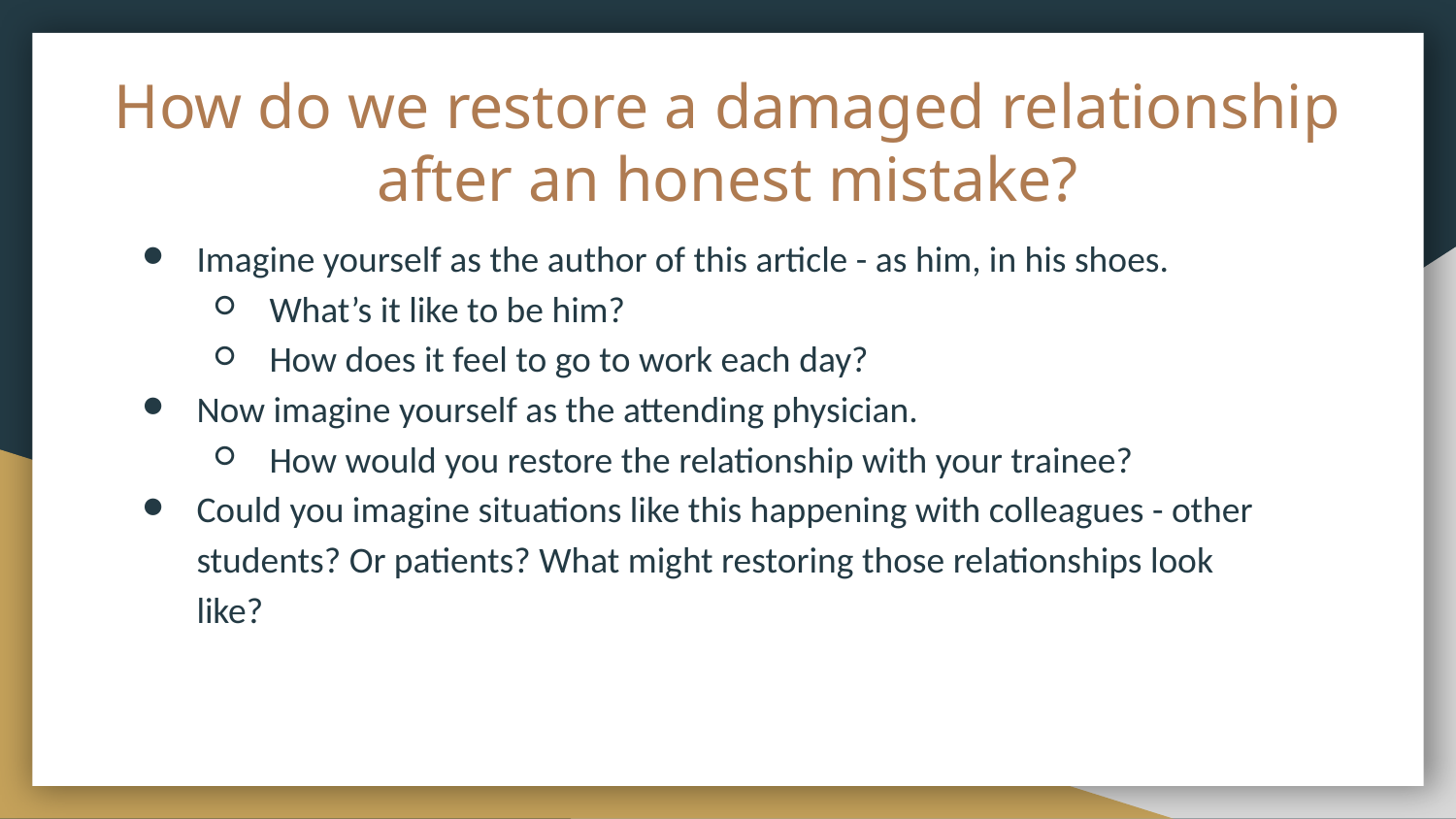

# How do we restore a damaged relationship after an honest mistake?
Imagine yourself as the author of this article - as him, in his shoes.
What’s it like to be him?
How does it feel to go to work each day?
Now imagine yourself as the attending physician.
How would you restore the relationship with your trainee?
Could you imagine situations like this happening with colleagues - other students? Or patients? What might restoring those relationships look like?

## Slide 21
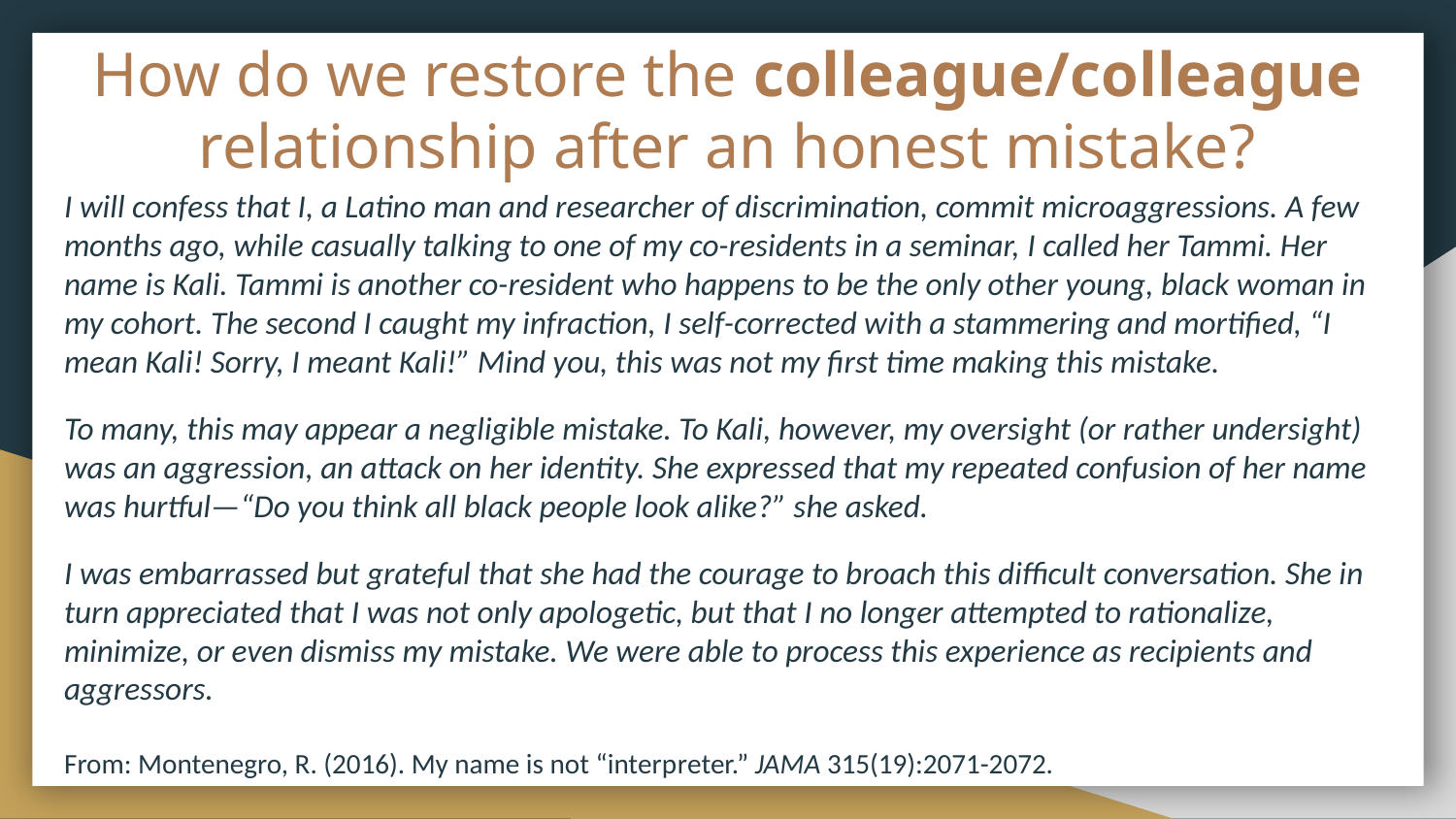

# How do we restore the colleague/colleague relationship after an honest mistake?
I will confess that I, a Latino man and researcher of discrimination, commit microaggressions. A few months ago, while casually talking to one of my co-residents in a seminar, I called her Tammi. Her name is Kali. Tammi is another co-resident who happens to be the only other young, black woman in my cohort. The second I caught my infraction, I self-corrected with a stammering and mortified, “I mean Kali! Sorry, I meant Kali!” Mind you, this was not my first time making this mistake.
To many, this may appear a negligible mistake. To Kali, however, my oversight (or rather undersight) was an aggression, an attack on her identity. She expressed that my repeated confusion of her name was hurtful—“Do you think all black people look alike?” she asked.
I was embarrassed but grateful that she had the courage to broach this difficult conversation. She in turn appreciated that I was not only apologetic, but that I no longer attempted to rationalize, minimize, or even dismiss my mistake. We were able to process this experience as recipients and aggressors.
From: Montenegro, R. (2016). My name is not “interpreter.” JAMA 315(19):2071-2072.

## Slide 22
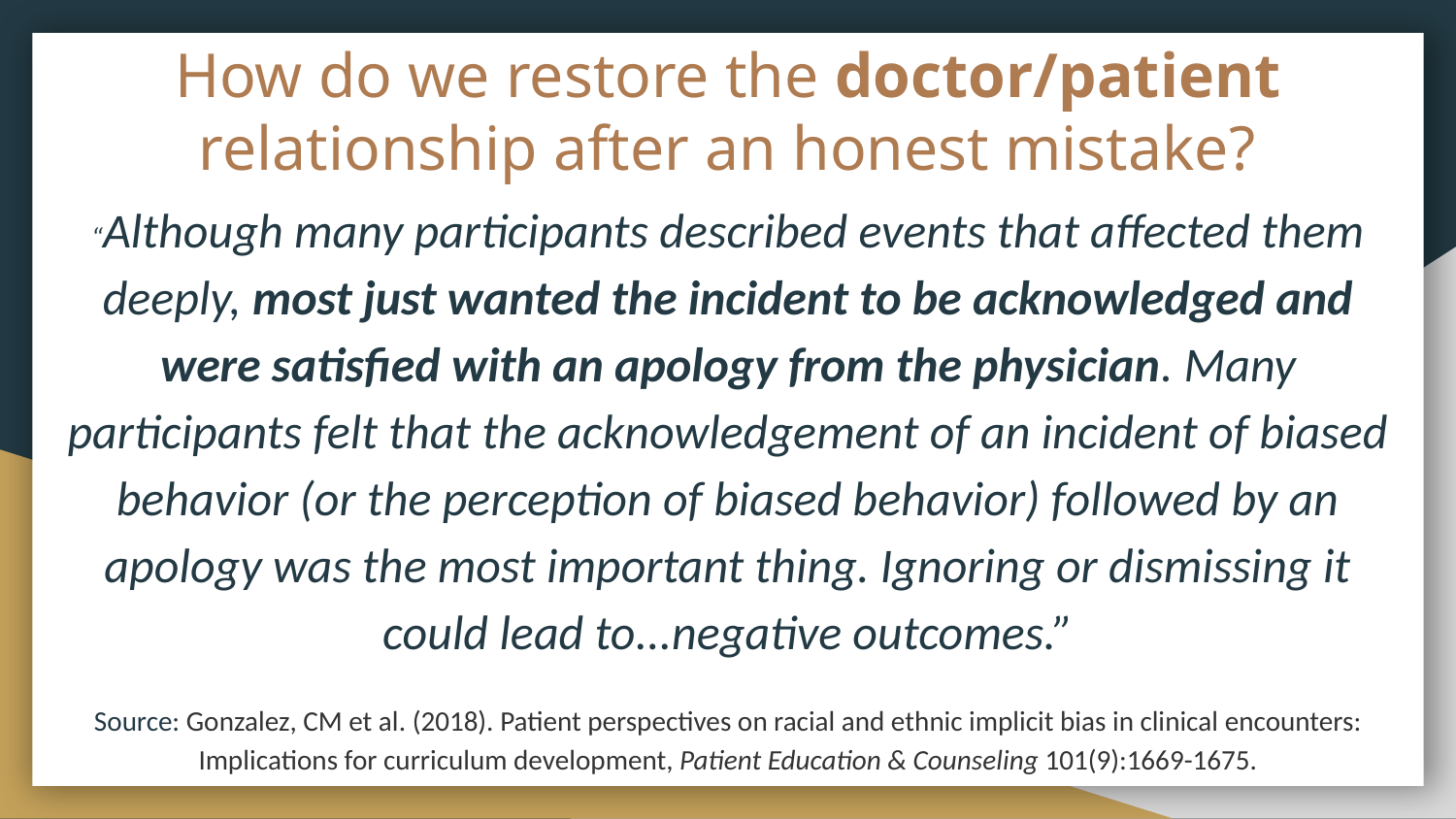

# How do we restore the doctor/patient relationship after an honest mistake?
“Although many participants described events that affected them deeply, most just wanted the incident to be acknowledged and were satisfied with an apology from the physician. Many participants felt that the acknowledgement of an incident of biased behavior (or the perception of biased behavior) followed by an apology was the most important thing. Ignoring or dismissing it could lead to...negative outcomes.”
Source: Gonzalez, CM et al. (2018). Patient perspectives on racial and ethnic implicit bias in clinical encounters:
Implications for curriculum development, Patient Education & Counseling 101(9):1669-1675.

## Slide 23
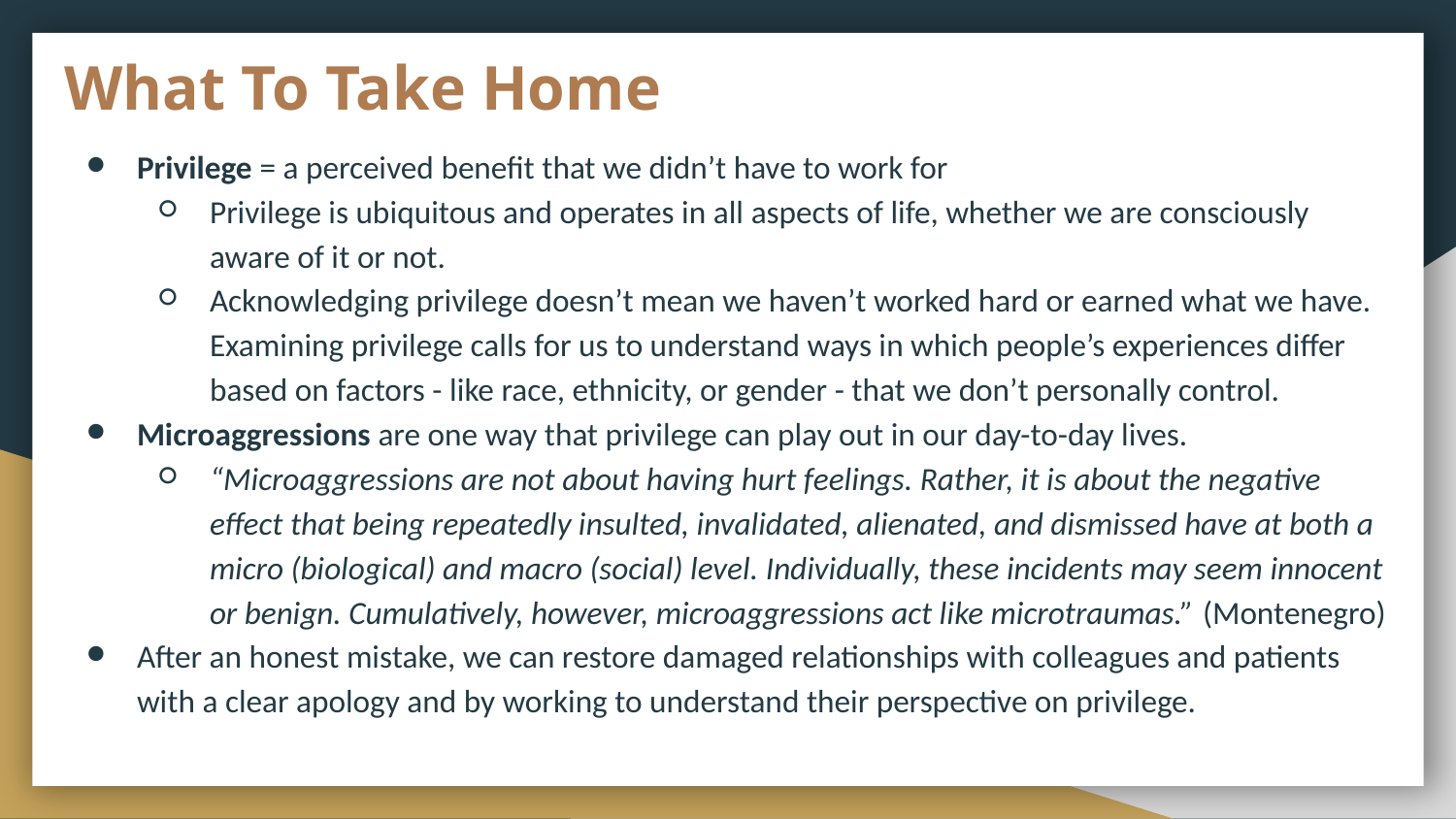

# What To Take Home
Privilege = a perceived benefit that we didn’t have to work for
Privilege is ubiquitous and operates in all aspects of life, whether we are consciously aware of it or not.
Acknowledging privilege doesn’t mean we haven’t worked hard or earned what we have. Examining privilege calls for us to understand ways in which people’s experiences differ based on factors - like race, ethnicity, or gender - that we don’t personally control.
Microaggressions are one way that privilege can play out in our day-to-day lives.
“Microaggressions are not about having hurt feelings. Rather, it is about the negative effect that being repeatedly insulted, invalidated, alienated, and dismissed have at both a micro (biological) and macro (social) level. Individually, these incidents may seem innocent or benign. Cumulatively, however, microaggressions act like microtraumas.” (Montenegro)
After an honest mistake, we can restore damaged relationships with colleagues and patients with a clear apology and by working to understand their perspective on privilege.

## Slide 24
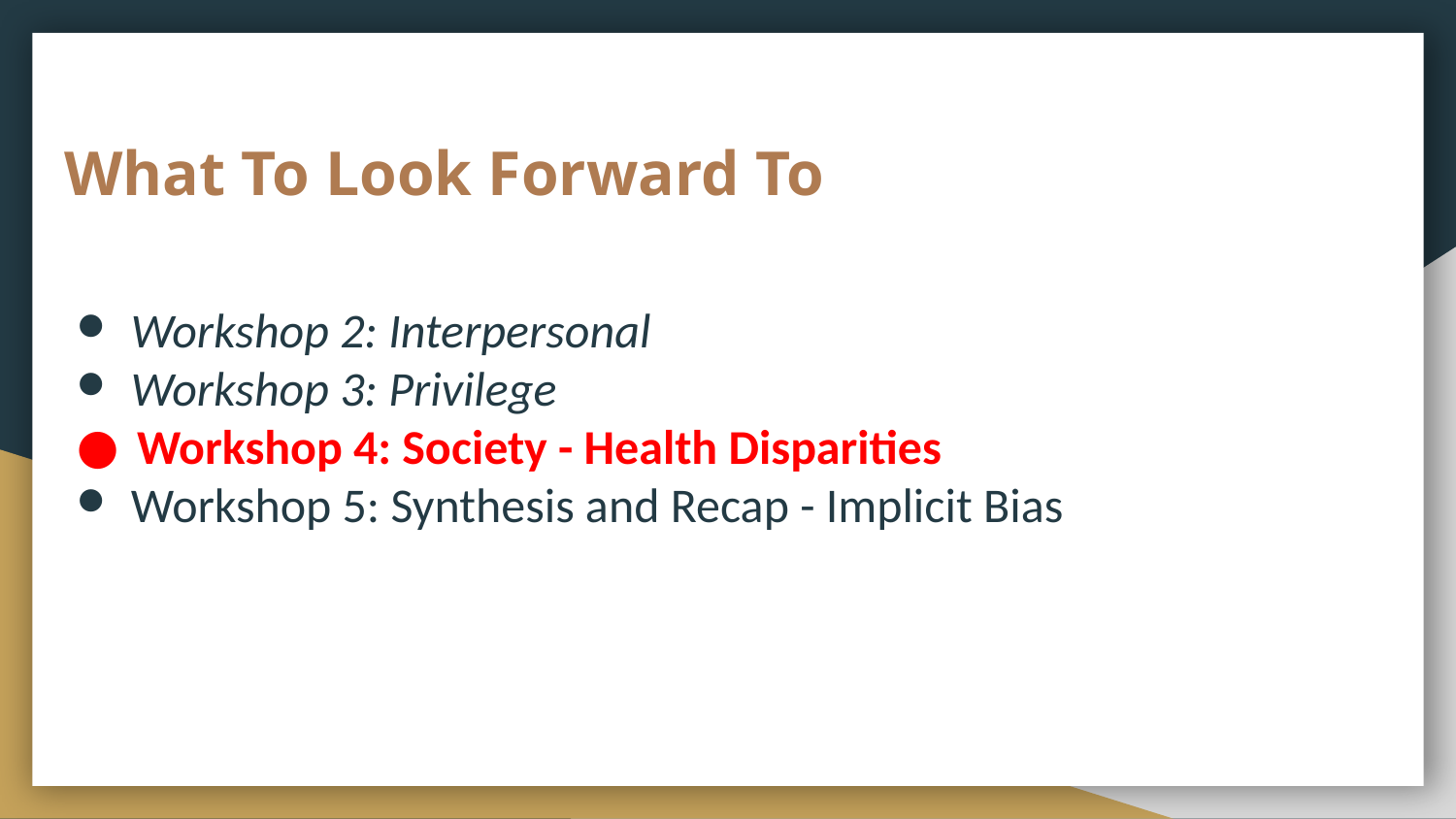

# What To Look Forward To
Workshop 2: Interpersonal
Workshop 3: Privilege
Workshop 4: Society - Health Disparities
Workshop 5: Synthesis and Recap - Implicit Bias
