# Supplementary material for: Critical Consciousness as a Framework for Health Equity–Focused Peer Learning
Source: MedEdPORTAL. 2021 Apr 28;17:11145. doi: 10.15766/mep_2374-8265.11145 (PMC8079426; doi:10.15766/mep_2374-8265.11145)
Supplement: Supplementary file 1 — Workshop 1 Presentation.pptxWorkshop 1 Student Handout.docxWorkshop 2 Presentation.pptxWorkshop 2 Student Handout.docxWorkshop 3 Presentation.pptxWorkshop 3 Student Handout.docxWorkshop 4 Presentation.pptxWorkshop 5 Presentation.pptxFacilitator Orientation.pptxWorkshop 1 Facilitator Guide.docxWorkshop 2 Facilitator Guide.docxWorkshop 3 Facilitator Guide.docxWorkshop 4 Facilitator Guide.docxWorkshop 5 Facilitator Guide.docxEvaluation Tools.docx [file mep_2374-8265.11145-s001.zip › F. Workshop 3 Student Handout.docx]

**CCM WORKSHOP #3: Privilege**

**SMALL GROUP DISCUSSION: Reflecting on Privilege Exercise**

*Below is the list of prompts from the pre-workshop survey.^[[1]](#footnote-1)^*

1. I can move about in public without fear of being harassed or physically attacked because of my sexual/gender identity.
2. I can assume that I will easily have physical access to any building.
3. I am never asked to speak for all the people of my racial/gender/other identifying group.
4. I never worry about being recognized as the sex/gender with which I identify.
5. I am less likely to be sexually harassed at work than persons of other gender identities.
6. I do not have to think about the message my wardrobe sends about my sexual availability.
7. In general, I am not under much pressure to be thin or to worry about how people will respond to me if I’m overweight.
8. I can do well in a challenging situation without being called a credit to my race/gender/other identifying group.
9. Mainstream media routinely depict people of my race in a wide range of roles.
10. If I have children and a successful career, few people will ask me how I balance work and home.
11. I feel safe walking by myself at night.
12. My parents went to college.
13. My family is able to help support me during school.
14. When I speak in a room full of my peers people listen.
15. I feel safe when I encounter law enforcement.
16. I can be sure that mainstream media will celebrate the holidays of my religion.
17. I do not need to worry about negative consequences of disclosing my religious identity to others.
18. I can be sure that my social class will be an advantage when I seek medical or legal help.
19. I don’t have to rely on public transportation; I can afford my own vehicle.
20. I have a savings account with at least a month’s expenses in case of emergency.
21. I am in medical school.
22. If I were a victim of a crime, I wouldn’t think twice about seeking police assistance due to my citizenship status.
23. If I wanted to, I could travel freely to almost any country and be admitted back into the U.S.

*****

**CASE DISCUSSION: Privilege**

*Excerpts from: Romano, MJ. (2018). White privilege in a white coat. Annals of Family Medicine 16(3):261-263.*

He was a young man in his late teens or early twenties, African American, and no longer alive. He entered the Baltimore hospital on a stretcher with multiple gunshot wounds piercing his torso and head with paramedics compressing his chest while pushing oxygen into his lungs. Within minutes of arrival, it was clear that further attempts at resuscitation were futile.

In the suddenly quiet moments following the pronouncement of death, the medical staff busied their restless hands cleaning the trauma bay and arranging the body for family members to pay their respects. Hastily torn sterile wrappers were brushed aside, torn and bloody clothing was removed, and a semblance of order was recreated in preparation for a suddenly grieving family.

One hospital staff member removed a cell phone from the patient’s pocket and then removed a second phone from the same pocket. A medical resident, wearing three pagers and two phones as part of his responsibilities on a busy night in the hospital, joked, “Maybe he was on call.”

Another resident corrected him, “No, I’ve seen *The Wire*, I know what this is about,” referencing a fictional television series set in Baltimore about drug-dealers who use multiple cell phones to evade police wire-taps.

In an instant, the room switched from nervous cleaning to nervous laughter. The humor resided in its improbability: a young black man shot in the chest and head was unlikely to be a physician on overnight call, instead he was immediately implicated as a criminal drug dealer based on his age, race, gender, manner of death, and the contents of his pockets, all substantiated by the house staff’s expertise in television crime dramas. That young man’s death has haunted me for years. I’ve turned it over again and again, troubled by those residents’ jokes and how they reflected the deeply ingrained racism of our medical system. Their mockery reinforced my implicit racist attitudes as an impressionable white medical student standing at the bedside. I failed to speak up and name the racist jokes for what they were, which I now deeply regret.

(excerpt continues on next page)

Inspired by the work of Peggy McIntosh, who cataloged the contents of her “invisible knapsack” of unearned white privilege, I set out to catalog some of the ways I accrue unearned privilege in medical training as a consequence of my white skin color:

- I have been taught since an early age that people of my own race can become doctors.
- Throughout my education, I could succeed academically without people questioning whether my accomplishments were attributable to affirmative action or my own abilities.
- During college and medical school, I never struggled to find professors and academic role models who shared my race.
- When I applied to medical school, I could choose from many elite institutions that were founded to train inexperienced doctors of my race by “practicing” medicine on urban and poor people of color.
- I am reminded daily that my medical knowledge is based on the discoveries made by people who looked like me without being reminded that some of the most painful discoveries were made through inhumane and nonconsensual experimentation on people of color.
- When I walk into an exam room with a person of color, patients invariably assume I am the doctor in charge, even if the person of color is my attending.
- If I respond to a call for medical assistance on an airplane, people will assume I am really a physician because of my race.
- Every American hospital I have ever entered contained portraits of department chairs and hospital presidents who are physicians of my race, reminding me of my race’s importance since the founding of these institutions.
- Even if I forget my identification badge, I can walk into the hospital and know that security guards will probably not stop me because of the color of my skin.
- When I travel to and from the hospital late at night as required by my job, I do not fear that I will be stopped, delayed, unjustly detained, inappropriately touched, injured, or killed by the police because of my race.
- I can attend most professional meetings confident that I will be surrounded by physicians who look like me, and that we will likely have mutual acquaintances who also share our race.
- I can speak my native language in my own dialect in professional settings without being viewed as uneducated or out-of-place.
- I know that I can leave the impoverished area where I work without being accused of abandoning my community.
- I can criticize medical institutions without being cast as a cultural outsider.
- I can name racism in my professional workspace and not be accused of being angry, potentially violent, or excessively emotional.
- When patients tell me they are “glad to have a white doctor,” I am not personally threatened, and I can choose to confront their racism or ignore it.
- I can pretend that health disparities don’t affect me or my family without acknowledging that we accrue benefits from a system that systematically favors our skin color.

*****

**CASE DISCUSSION: How do we restore a damaged relationship after an honest mistake?**

*Excerpts from: Montenegro, R. (2016). My name is not “interpreter.” JAMA 315(19):2071-2072.*

Medical institutions can create environments that either facilitate or curb microaggressions. As a medical student, I often felt marginalized from my medical community. I have been told that my name is “not American,” fallen prey to being confused for support staff such as a janitor (even while wearing my white coat), and been asked questions like “Where are you really from?” or “How old were you when you moved to the United States?” or “When you’re done with your training, are you going back to your country?” The greatest barb, however, was being summoned as “interpreter” by an attending physician during my surgery rounds.

“Interpreter!” he would bark out. I instantly appeared before the group and would begin to interpret.

I dreaded the days I had to work with this attending. Like most other recipients of microaggressions, I was stuck trying to decide if and how I would respond to these comments, especially in such a disproportionate power dynamic. Is he trying to help me by showcasing my language skills? Is he just trying to be funny? Am I overreacting? Will I be perceived as “the angry minority” if I speak up? At these instances, I felt helpless and powerless—there were no open conversations about racism, and I was left alone to deal with these difficult situations.

As a resident, I naively thought these unfortunate life circumstances would become less frequent. They have not—they just morph into other forms of microaggressions.

While on my internal medicine rotation, for instance, after presenting my assessment and plan to an attending physician, he responded with a football analogy that I did not understand. His reply: “Oh, I’m sorry. That’s right. In your country it’s the other football. Soccer, right?” I had never worked with this attending; he knew nothing about me other than my name and what I looked like.

*****

1. Survey prompts adapted from Holm AL, Gorosh MR, Brady M, White-Perkins, D. Recognizing privilege and bias: An interactive exercise to expand health care providers’ personal awareness. *Academic Medicine*. 2017;92(3)360-364. [↑](#footnote-ref-1)
