# Supplementary material for: Critical Consciousness as a Framework for Health Equity–Focused Peer Learning
Source: MedEdPORTAL. 2021 Apr 28;17:11145. doi: 10.15766/mep_2374-8265.11145 (PMC8079426; doi:10.15766/mep_2374-8265.11145)
Supplement: Supplementary file 1 — Workshop 1 Presentation.pptxWorkshop 1 Student Handout.docxWorkshop 2 Presentation.pptxWorkshop 2 Student Handout.docxWorkshop 3 Presentation.pptxWorkshop 3 Student Handout.docxWorkshop 4 Presentation.pptxWorkshop 5 Presentation.pptxFacilitator Orientation.pptxWorkshop 1 Facilitator Guide.docxWorkshop 2 Facilitator Guide.docxWorkshop 3 Facilitator Guide.docxWorkshop 4 Facilitator Guide.docxWorkshop 5 Facilitator Guide.docxEvaluation Tools.docx [file mep_2374-8265.11145-s001.zip › G. Workshop 4 Presentation.pptx]

## Slide 1
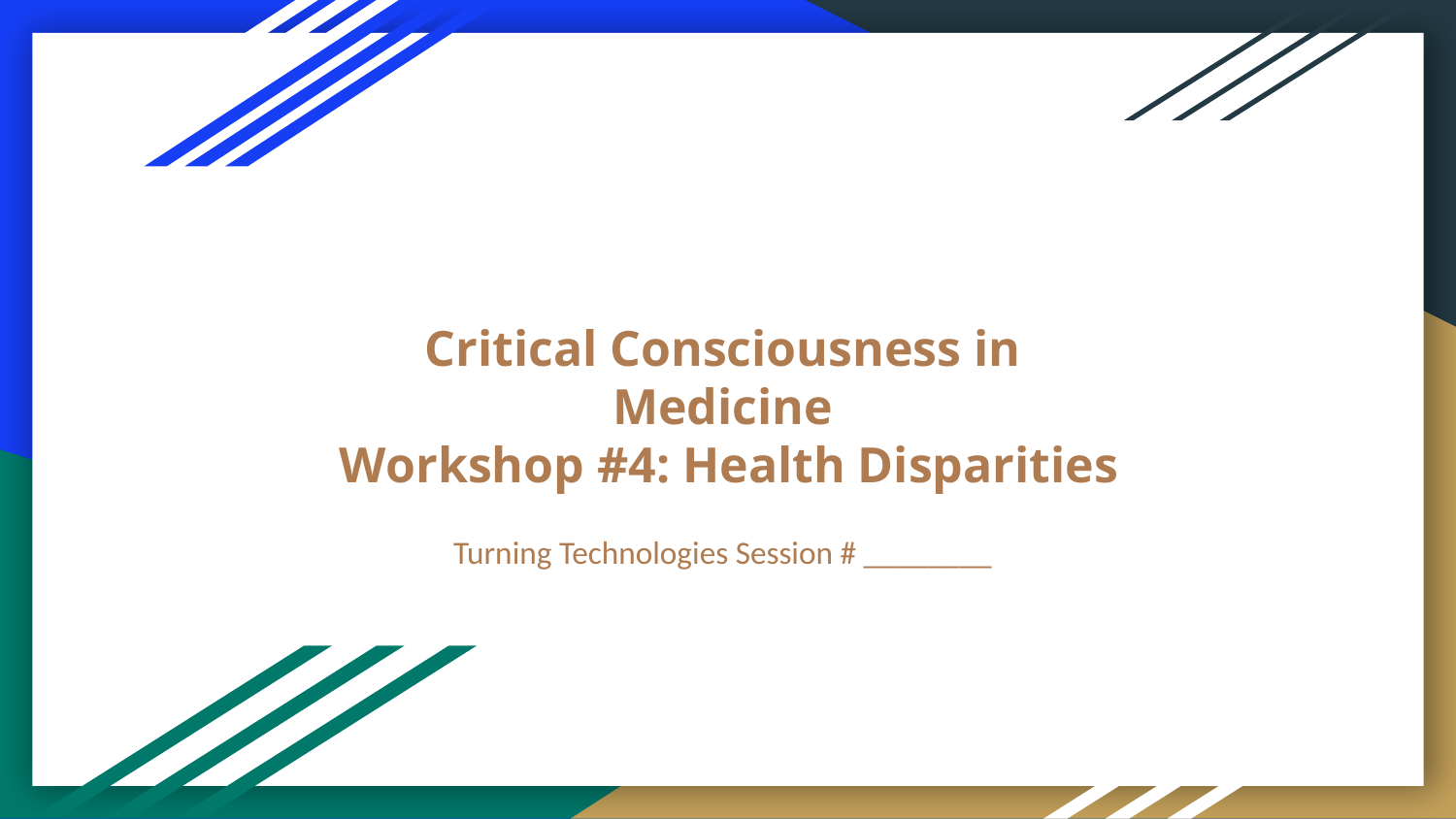

# Critical Consciousness in Medicine Workshop #4: Health Disparities
Turning Technologies Session # ________

## Slide 2
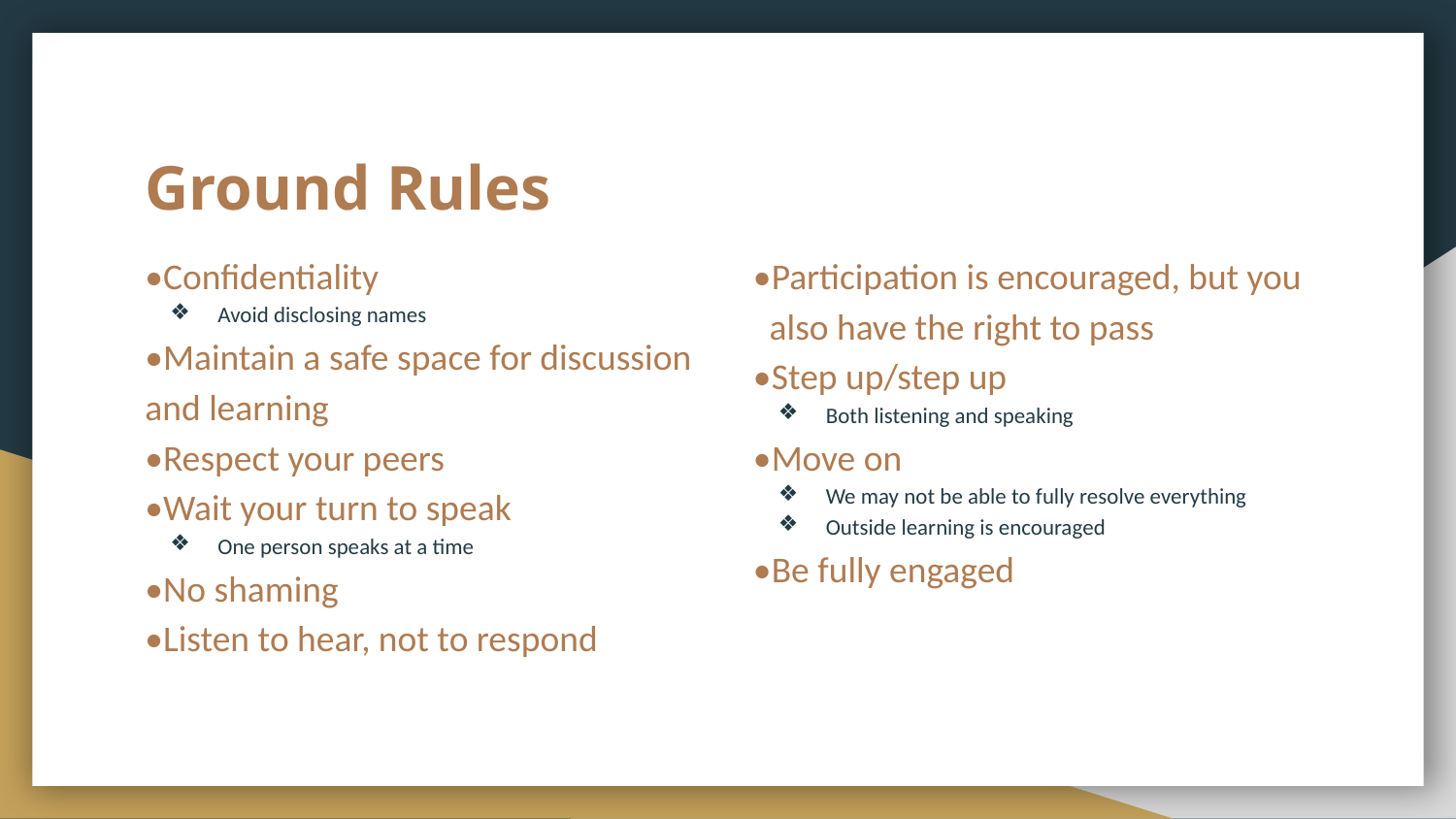

# Ground Rules
•Confidentiality
Avoid disclosing names
•Maintain a safe space for discussion and learning
•Respect your peers
•Wait your turn to speak
One person speaks at a time
•No shaming
•Listen to hear, not to respond
•Participation is encouraged, but you also have the right to pass
•Step up/step up
Both listening and speaking
•Move on
We may not be able to fully resolve everything
Outside learning is encouraged
•Be fully engaged

## Slide 3
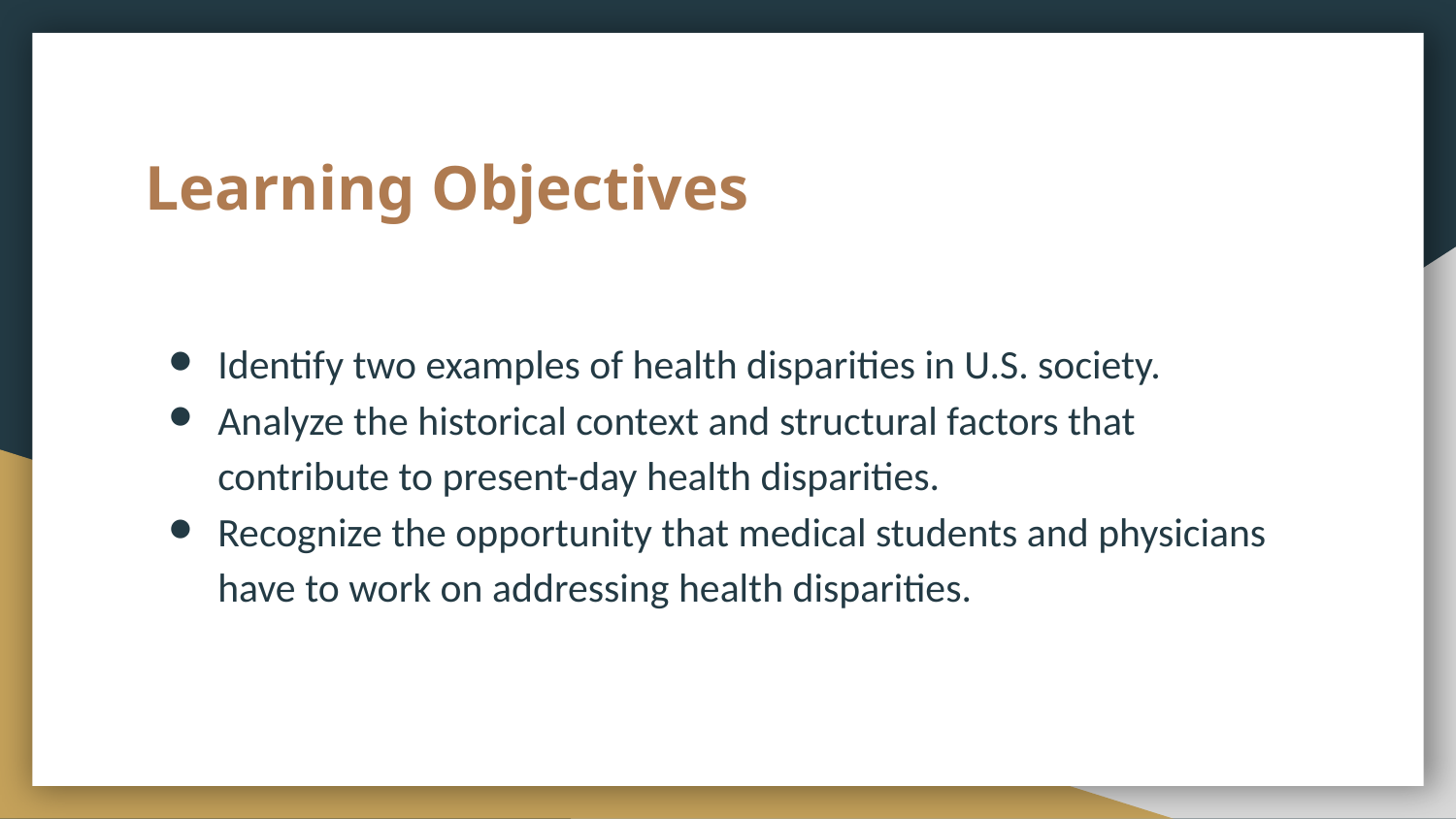

# Learning Objectives
Identify two examples of health disparities in U.S. society.
Analyze the historical context and structural factors that contribute to present-day health disparities.
Recognize the opportunity that medical students and physicians have to work on addressing health disparities.

## Slide 4
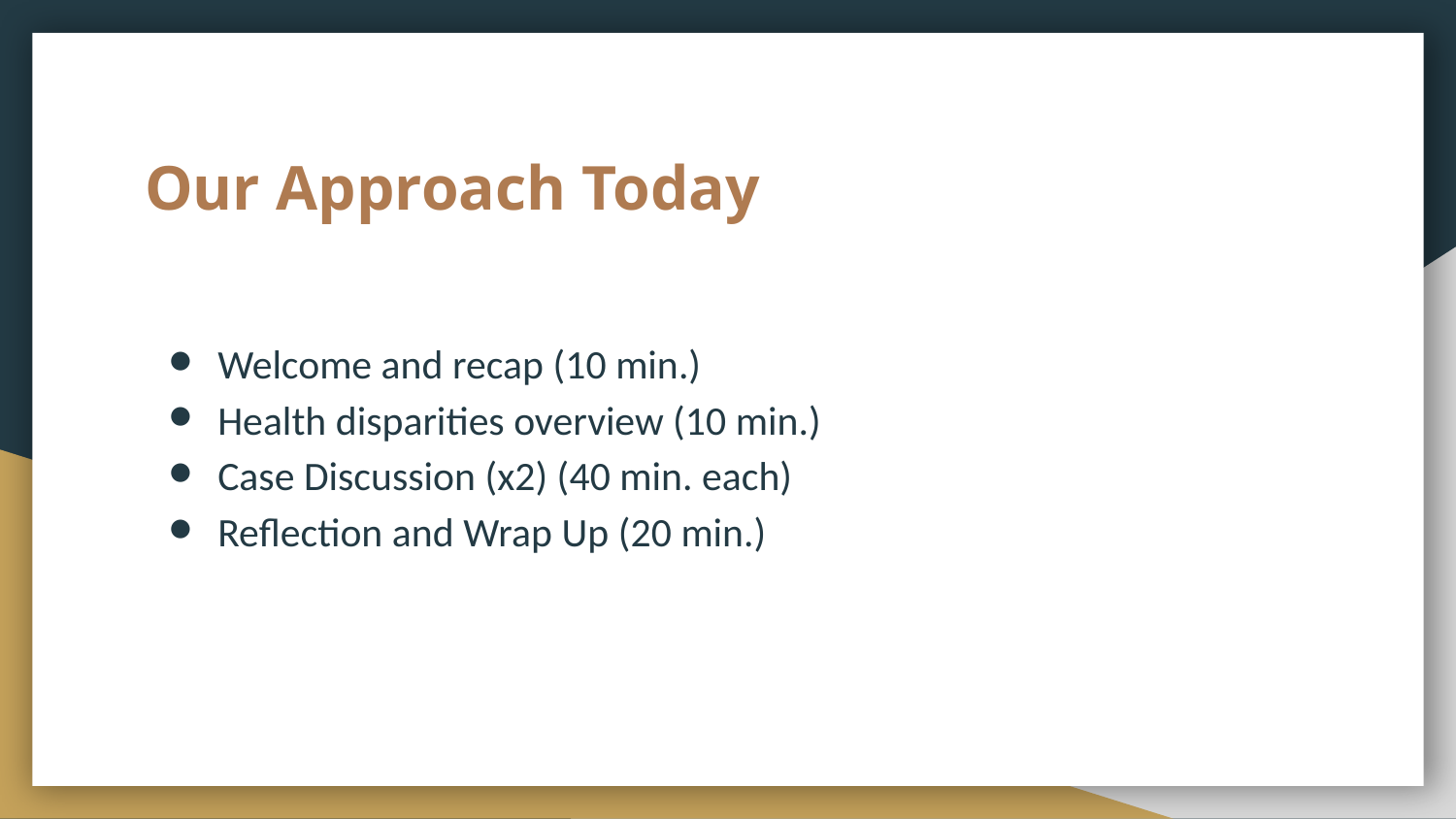

# Our Approach Today
Welcome and recap (10 min.)
Health disparities overview (10 min.)
Case Discussion (x2) (40 min. each)
Reflection and Wrap Up (20 min.)

## Slide 5
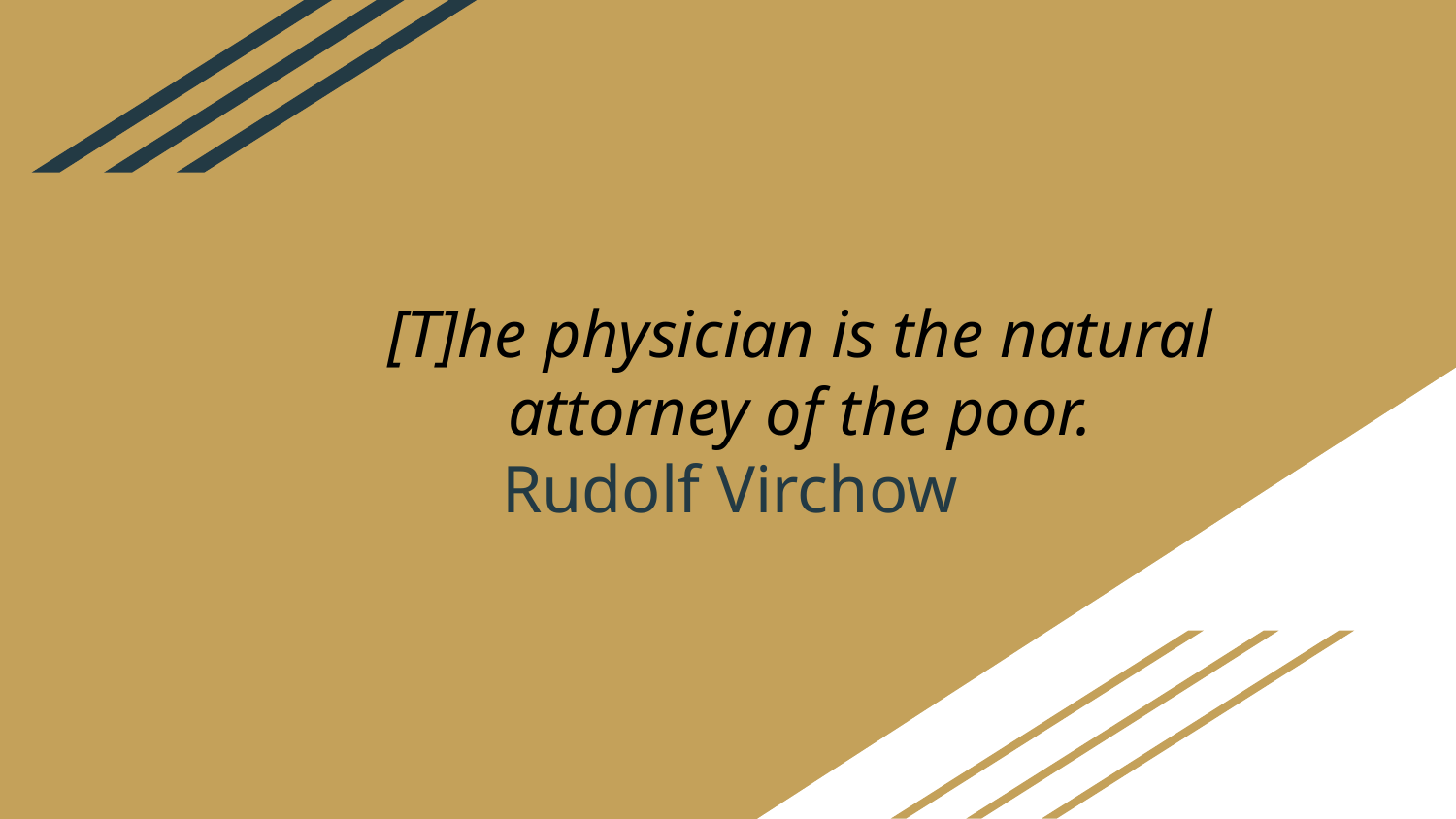

# [T]he physician is the natural attorney of the poor.
Rudolf Virchow

## Slide 6
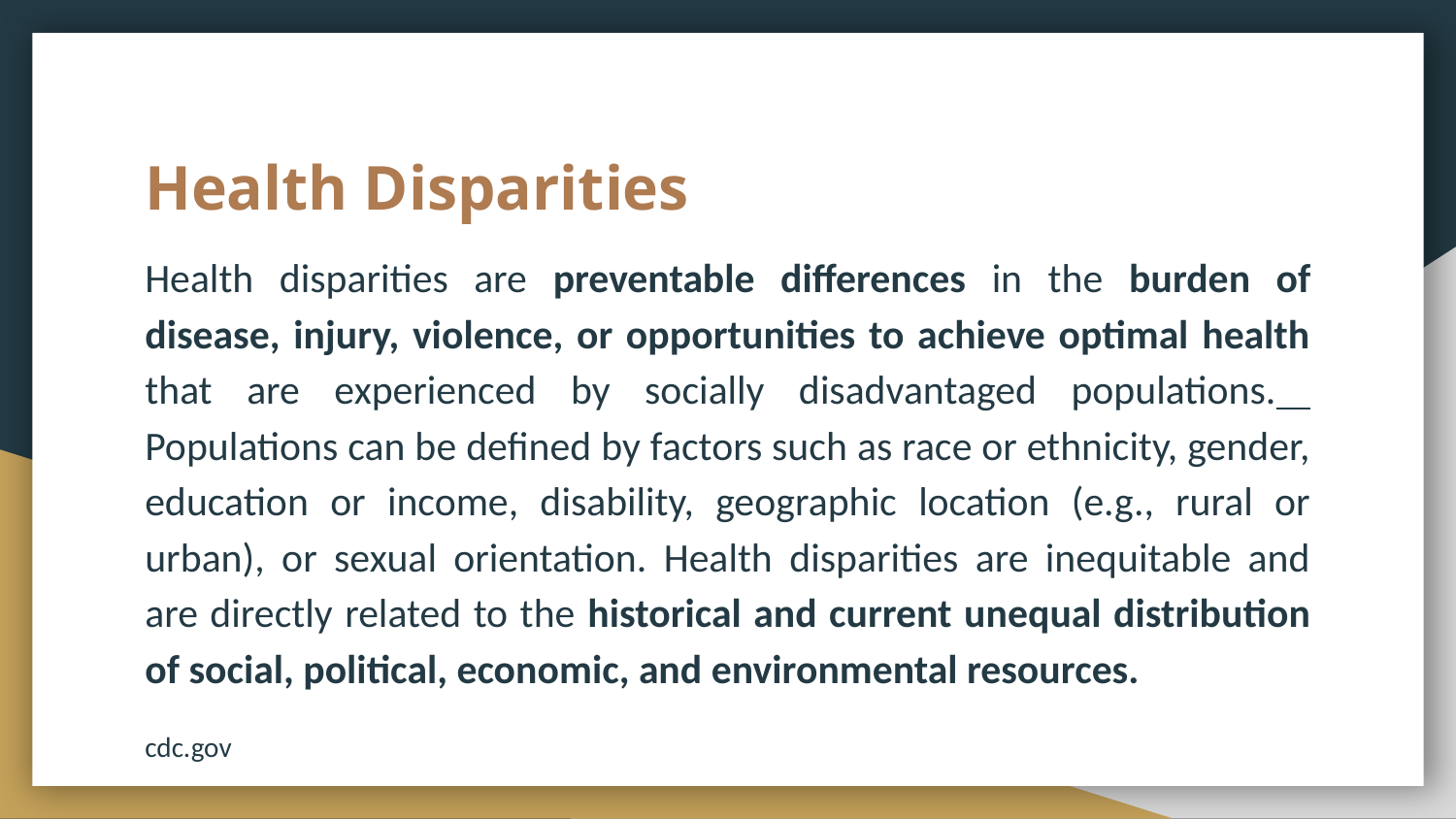

# Health Disparities
Health disparities are preventable differences in the burden of disease, injury, violence, or opportunities to achieve optimal health that are experienced by socially disadvantaged populations. Populations can be defined by factors such as race or ethnicity, gender, education or income, disability, geographic location (e.g., rural or urban), or sexual orientation. Health disparities are inequitable and are directly related to the historical and current unequal distribution of social, political, economic, and environmental resources.
cdc.gov

## Slide 7
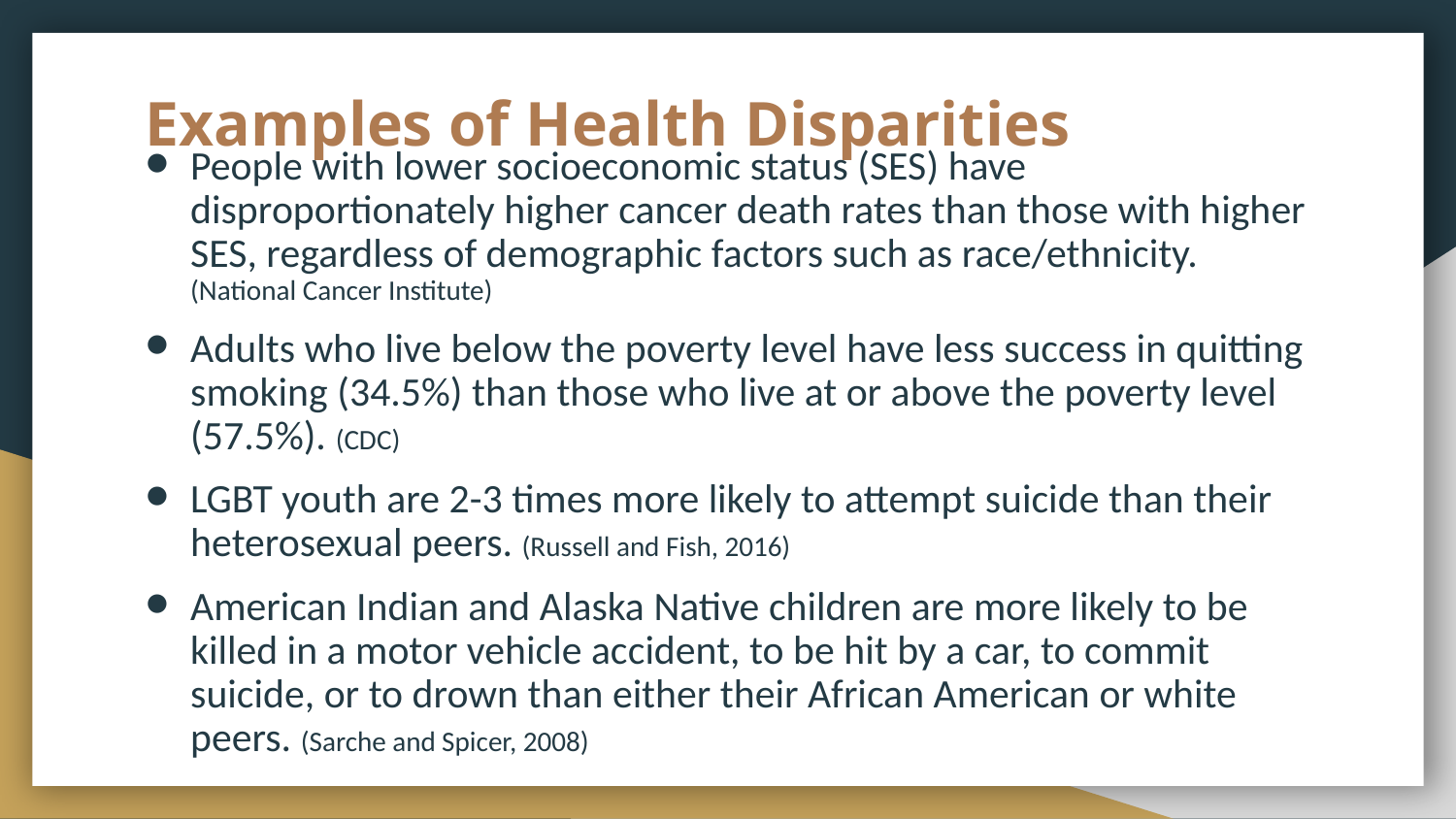

# Examples of Health Disparities
People with lower socioeconomic status (SES) have disproportionately higher cancer death rates than those with higher SES, regardless of demographic factors such as race/ethnicity. (National Cancer Institute)
Adults who live below the poverty level have less success in quitting smoking (34.5%) than those who live at or above the poverty level (57.5%). (CDC)
LGBT youth are 2-3 times more likely to attempt suicide than their heterosexual peers. (Russell and Fish, 2016)
American Indian and Alaska Native children are more likely to be killed in a motor vehicle accident, to be hit by a car, to commit suicide, or to drown than either their African American or white peers. (Sarche and Spicer, 2008)

## Slide 8
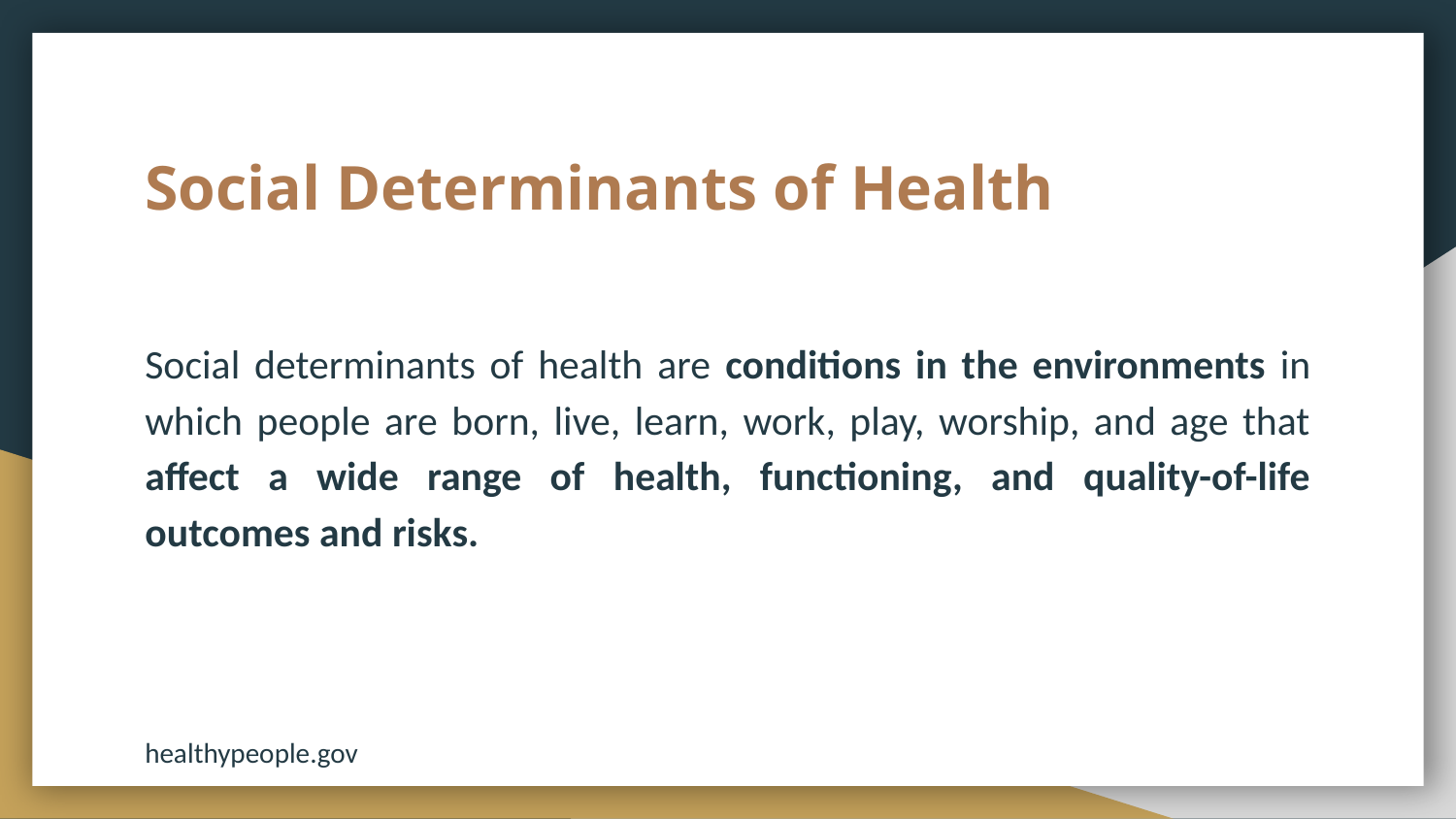

# Social Determinants of Health
Social determinants of health are conditions in the environments in which people are born, live, learn, work, play, worship, and age that affect a wide range of health, functioning, and quality-of-life outcomes and risks.
healthypeople.gov

## Slide 9
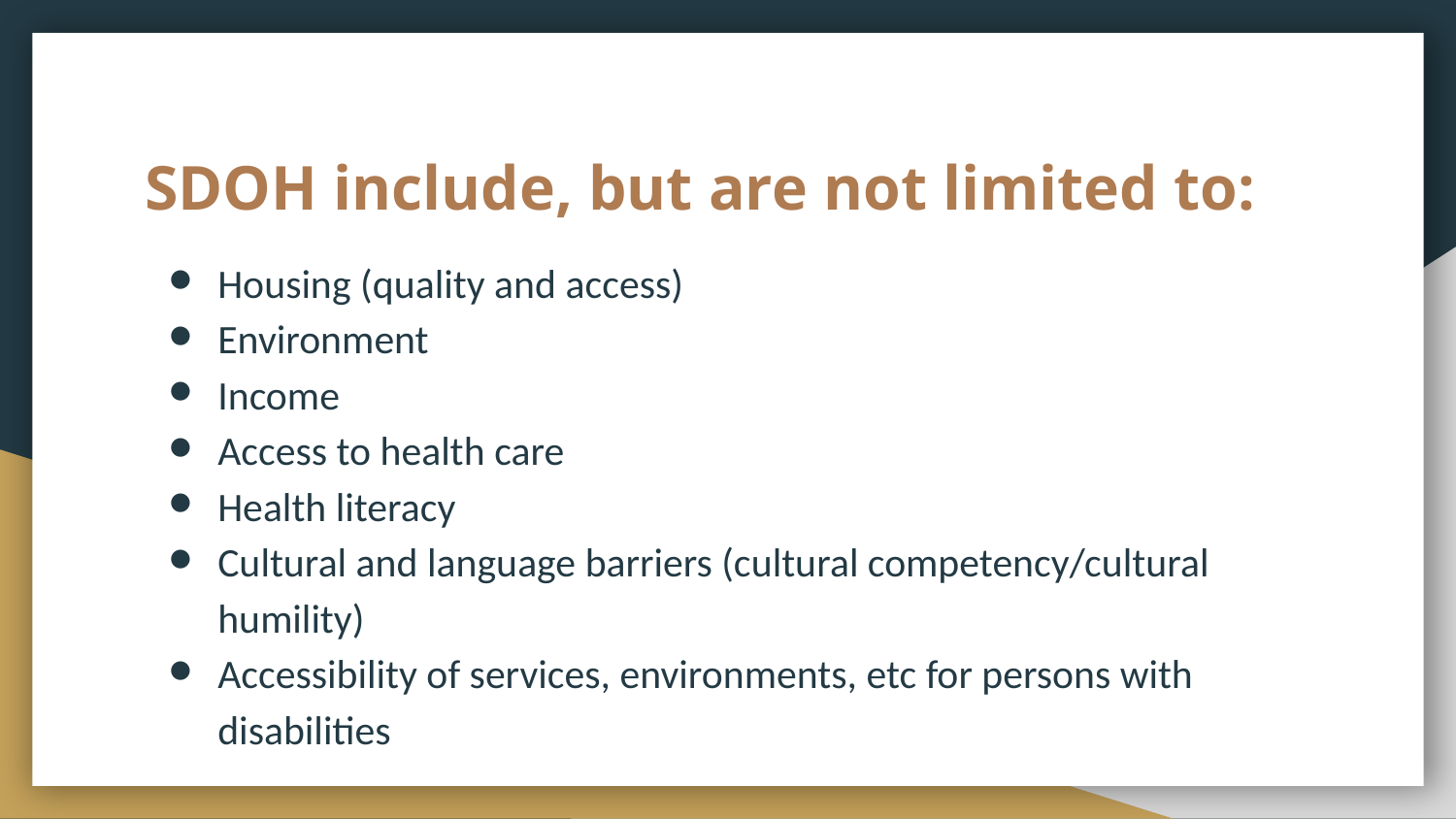

# SDOH include, but are not limited to:
Housing (quality and access)
Environment
Income
Access to health care
Health literacy
Cultural and language barriers (cultural competency/cultural humility)
Accessibility of services, environments, etc for persons with disabilities

## Slide 10
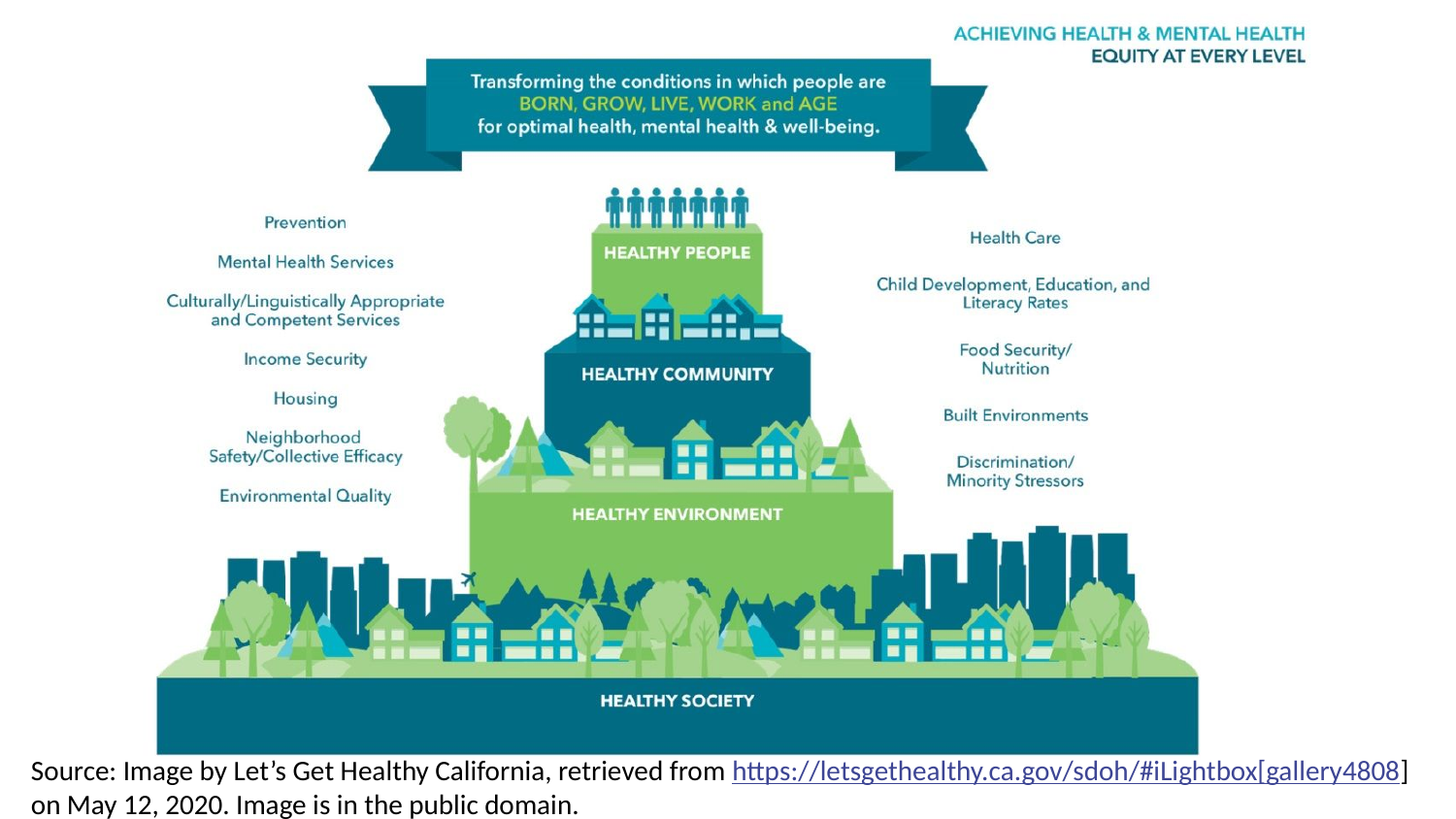

Source: Image by Let’s Get Healthy California, retrieved from https://letsgethealthy.ca.gov/sdoh/#iLightbox[gallery4808] on May 12, 2020. Image is in the public domain.

## Slide 11
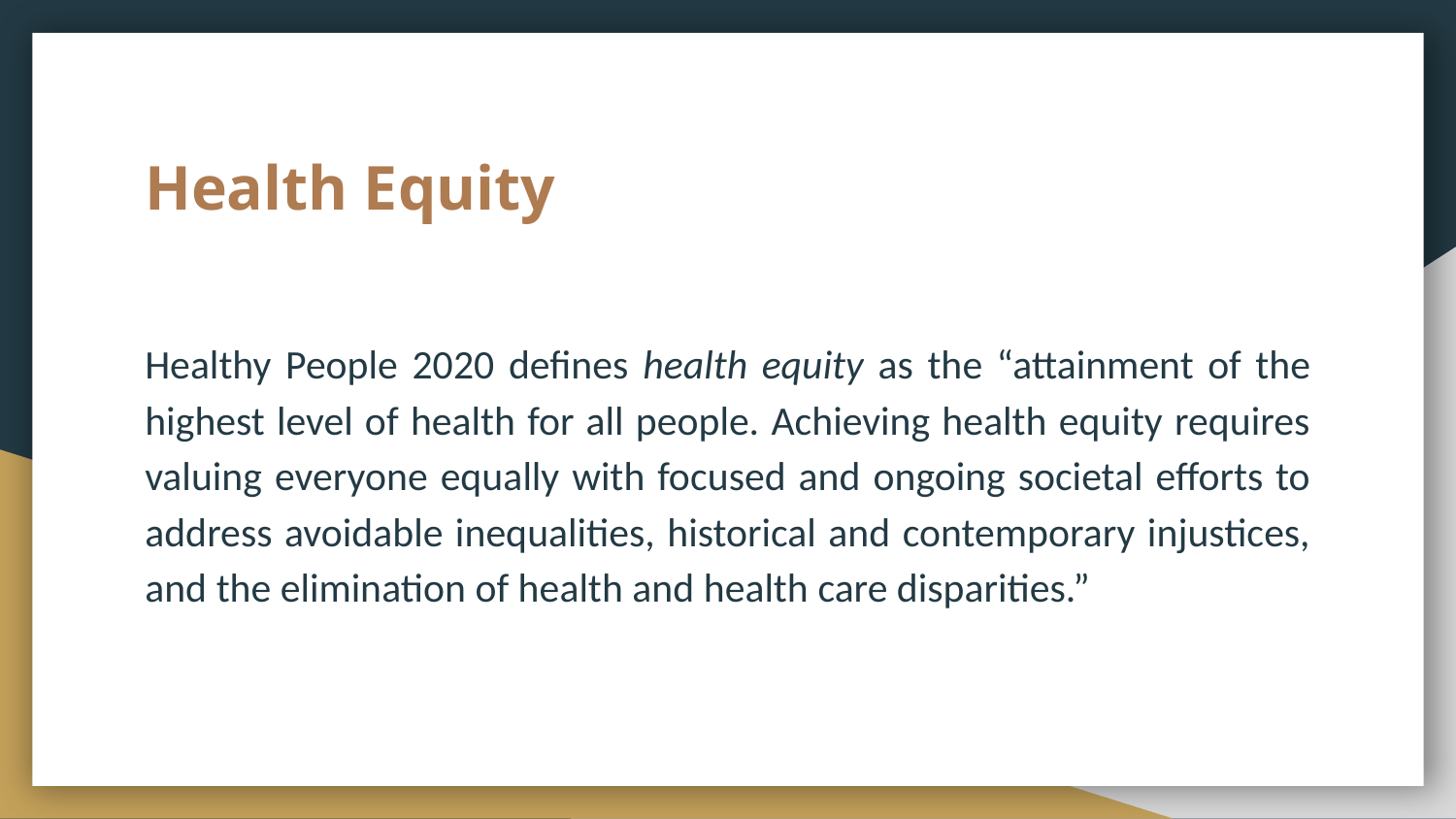

# Health Equity
Healthy People 2020 defines health equity as the “attainment of the highest level of health for all people. Achieving health equity requires valuing everyone equally with focused and ongoing societal efforts to address avoidable inequalities, historical and contemporary injustices, and the elimination of health and health care disparities.”

## Slide 12
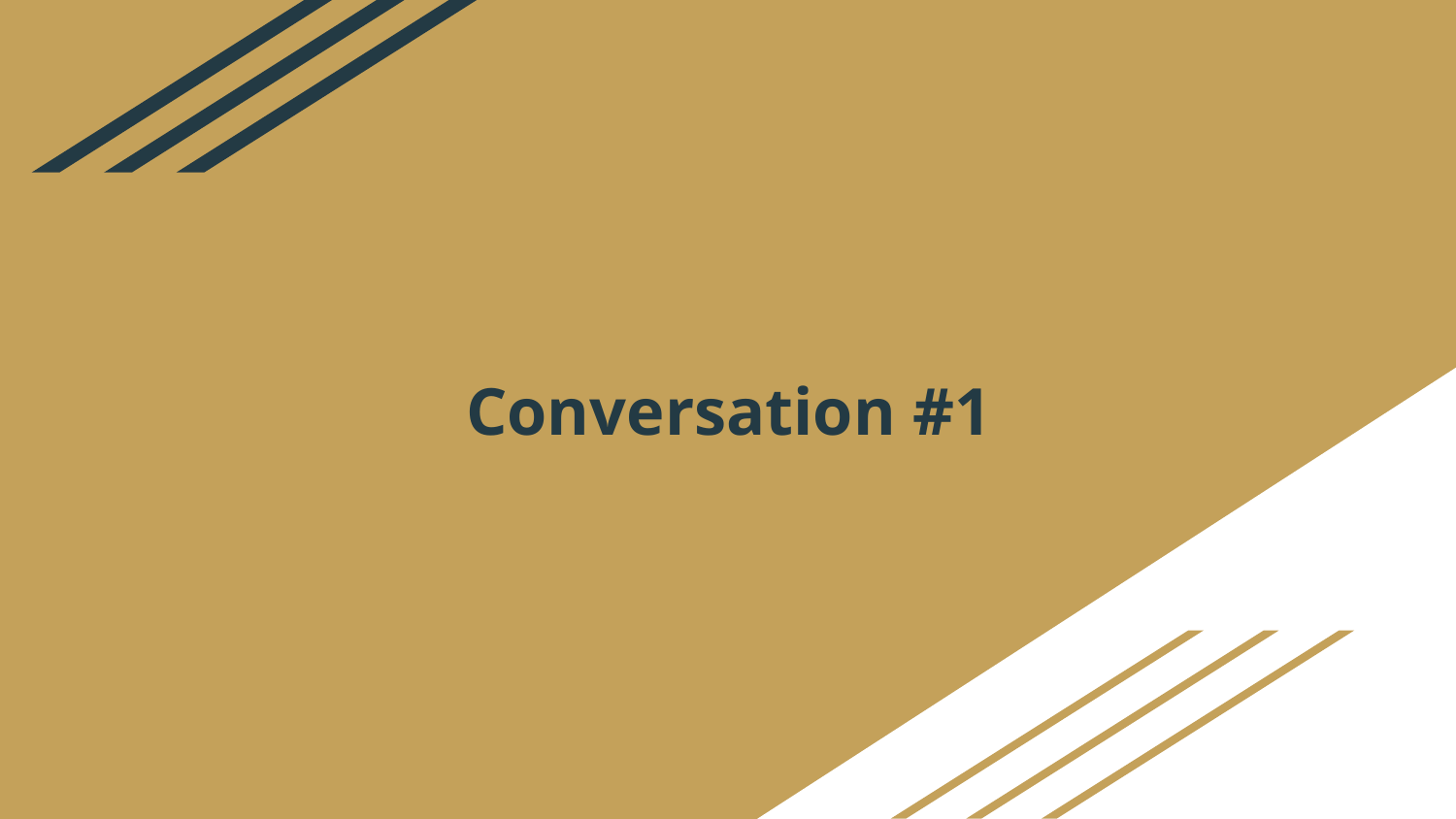

# Conversation #1

## Slide 13
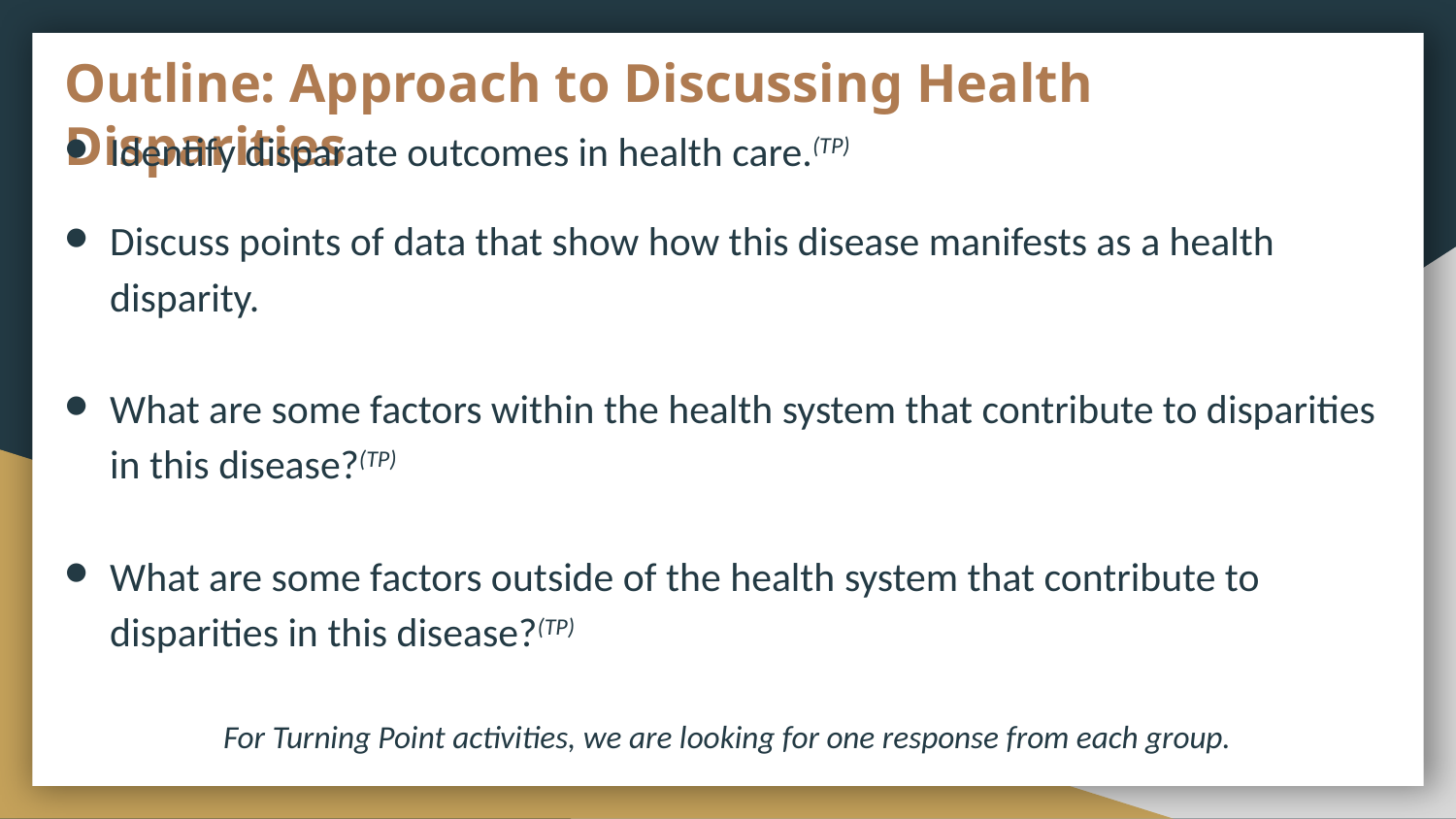

# Outline: Approach to Discussing Health Disparities
Identify disparate outcomes in health care.(TP)
Discuss points of data that show how this disease manifests as a health disparity.
What are some factors within the health system that contribute to disparities in this disease?(TP)
What are some factors outside of the health system that contribute to disparities in this disease?(TP)
For Turning Point activities, we are looking for one response from each group.

## Slide 14
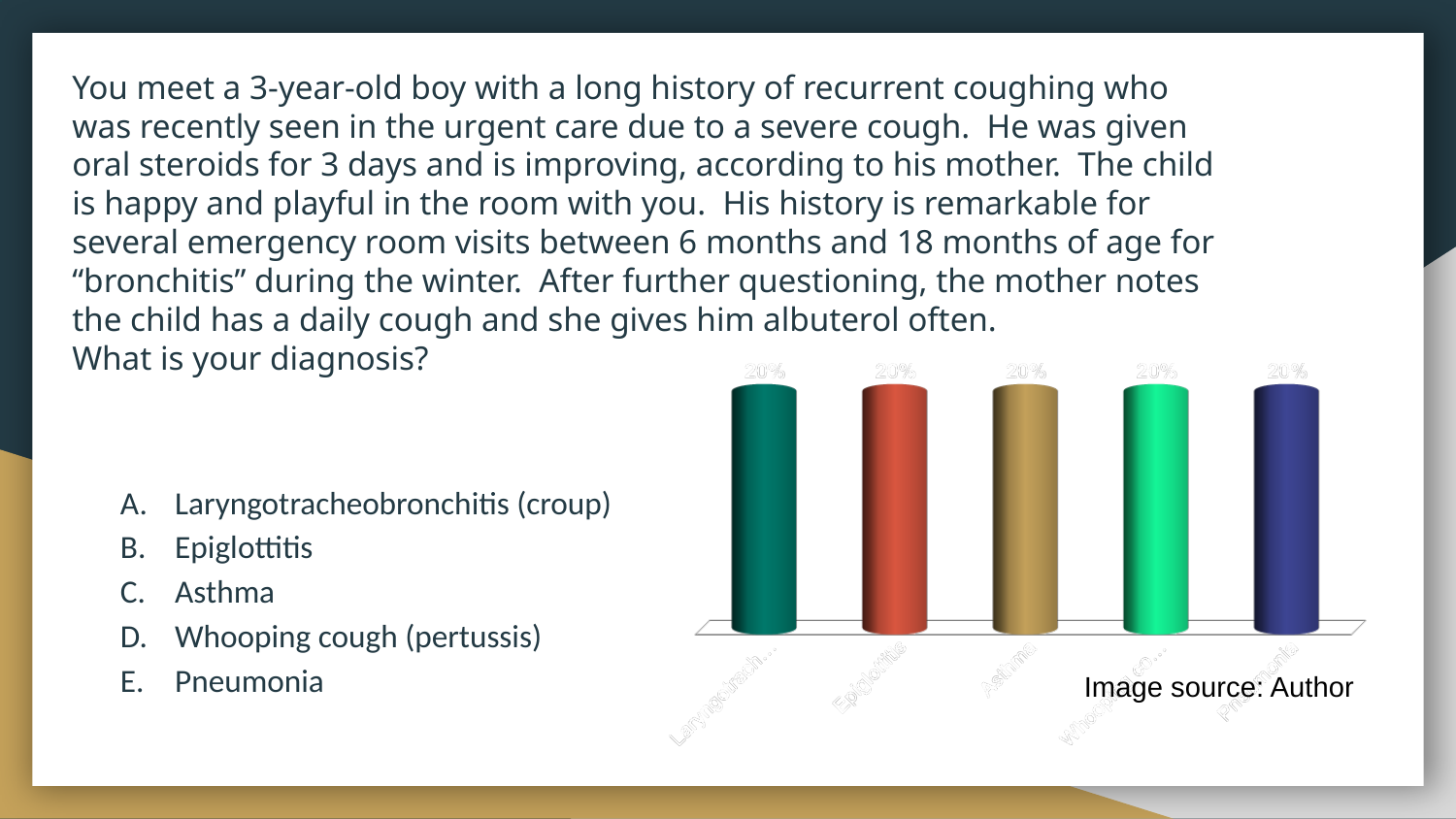

# You meet a 3-year-old boy with a long history of recurrent coughing who was recently seen in the urgent care due to a severe cough. He was given oral steroids for 3 days and is improving, according to his mother. The child is happy and playful in the room with you. His history is remarkable for several emergency room visits between 6 months and 18 months of age for “bronchitis” during the winter. After further questioning, the mother notes the child has a daily cough and she gives him albuterol often. What is your diagnosis?
Laryngotracheobronchitis (croup)
Epiglottitis
Asthma
Whooping cough (pertussis)
Pneumonia
Image source: Author

## Slide 15
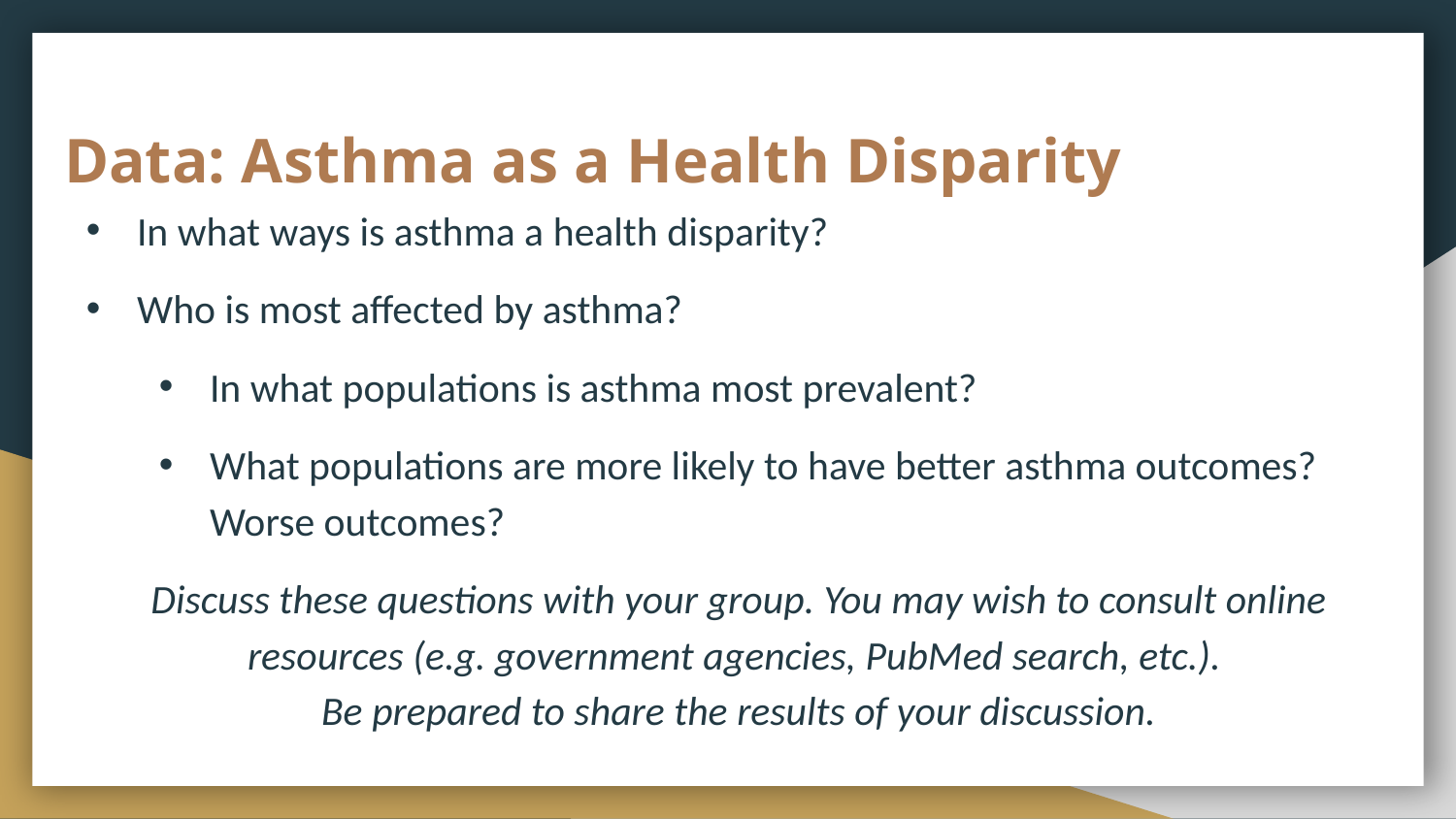

# Data: Asthma as a Health Disparity
In what ways is asthma a health disparity?
Who is most affected by asthma?
In what populations is asthma most prevalent?
What populations are more likely to have better asthma outcomes? Worse outcomes?
Discuss these questions with your group. You may wish to consult online resources (e.g. government agencies, PubMed search, etc.).
Be prepared to share the results of your discussion.

## Slide 16
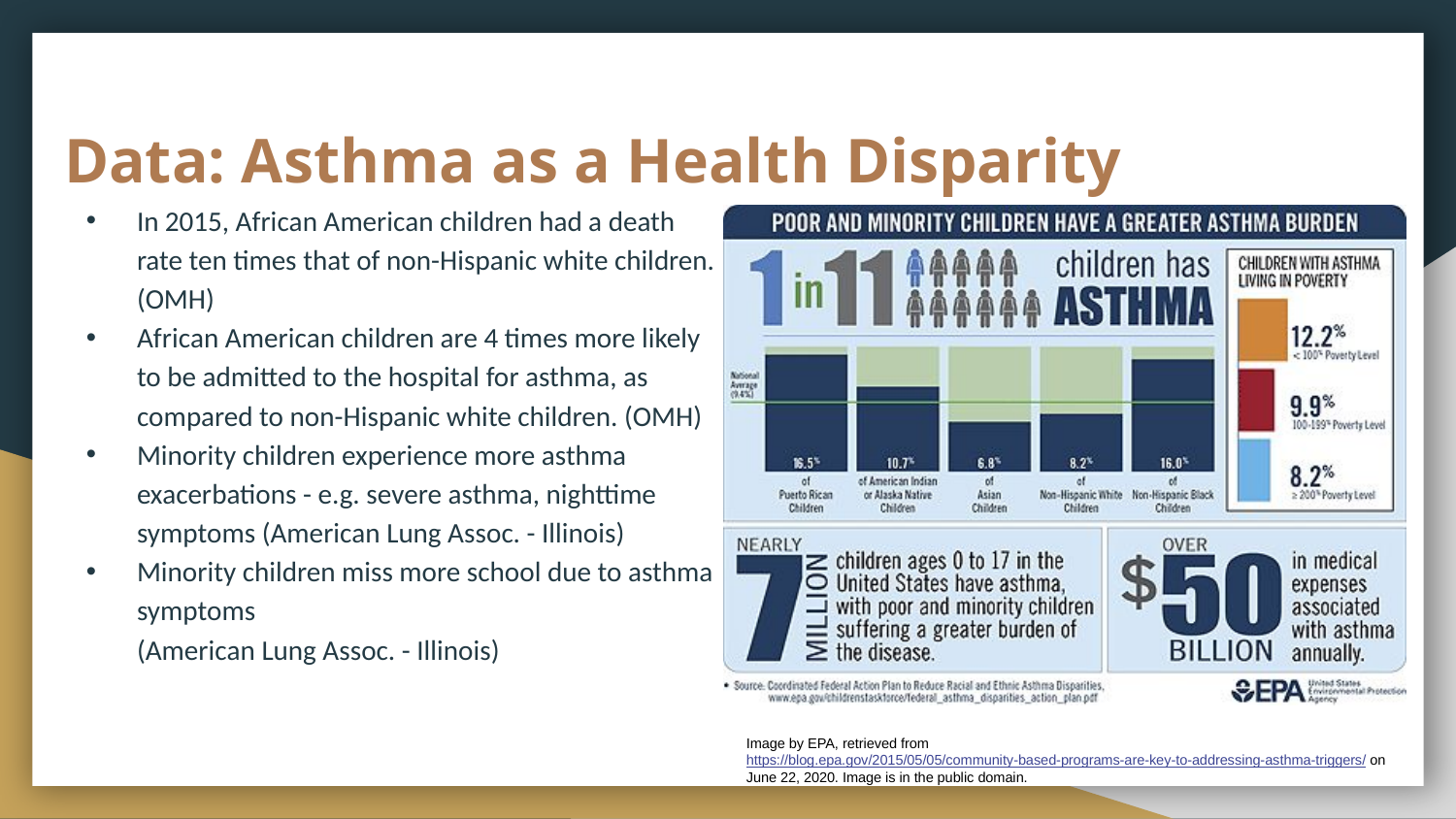

# Data: Asthma as a Health Disparity
In 2015, African American children had a death rate ten times that of non-Hispanic white children. (OMH)
African American children are 4 times more likely to be admitted to the hospital for asthma, as compared to non-Hispanic white children. (OMH)
Minority children experience more asthma exacerbations - e.g. severe asthma, nighttime symptoms (American Lung Assoc. - Illinois)
Minority children miss more school due to asthma symptoms
(American Lung Assoc. - Illinois)
Image by EPA, retrieved from https://blog.epa.gov/2015/05/05/community-based-programs-are-key-to-addressing-asthma-triggers/ on June 22, 2020. Image is in the public domain.

## Slide 17
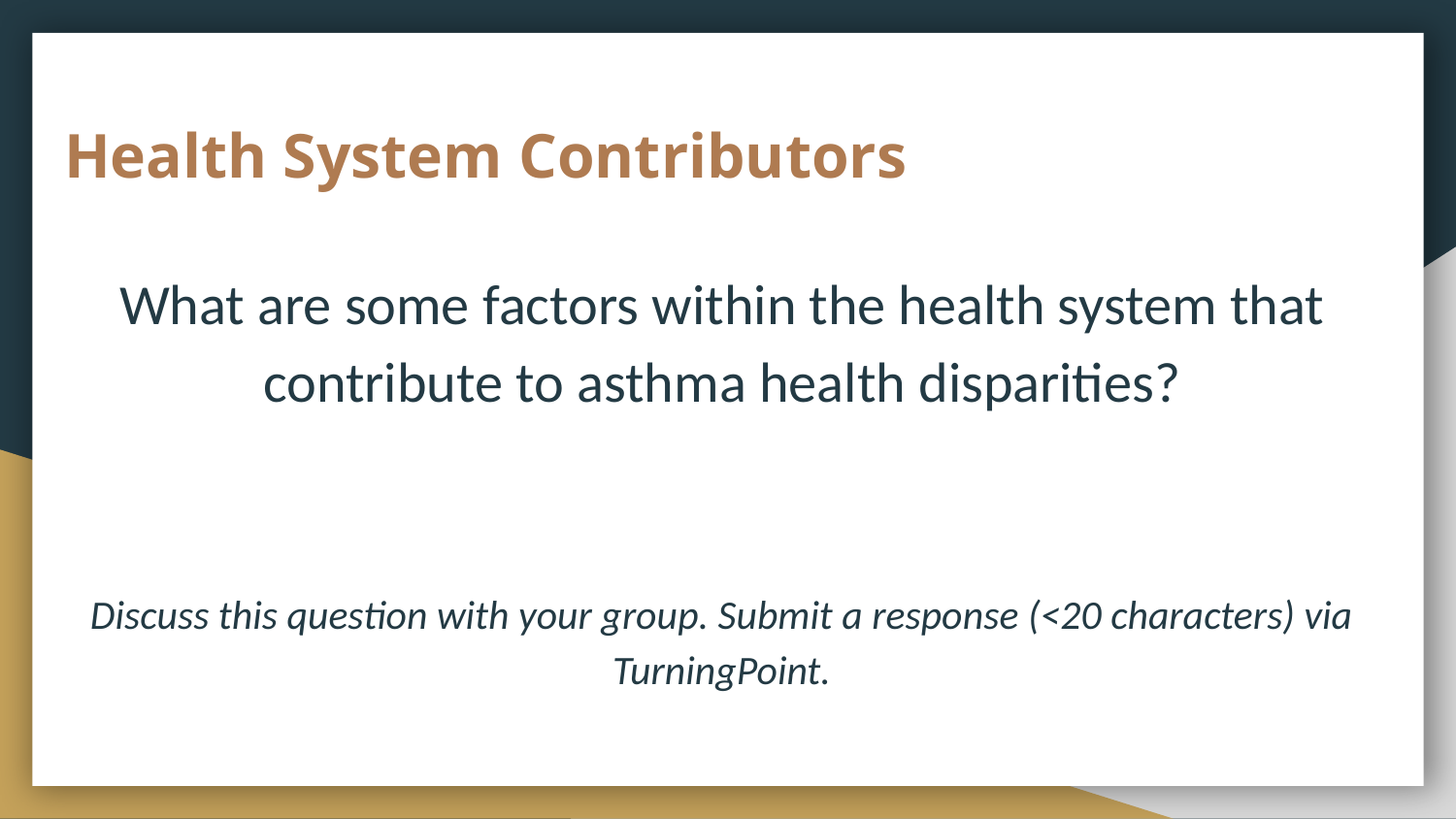

# Health System Contributors
What are some factors within the health system that contribute to asthma health disparities?
Discuss this question with your group. Submit a response (<20 characters) via TurningPoint.

## Slide 18
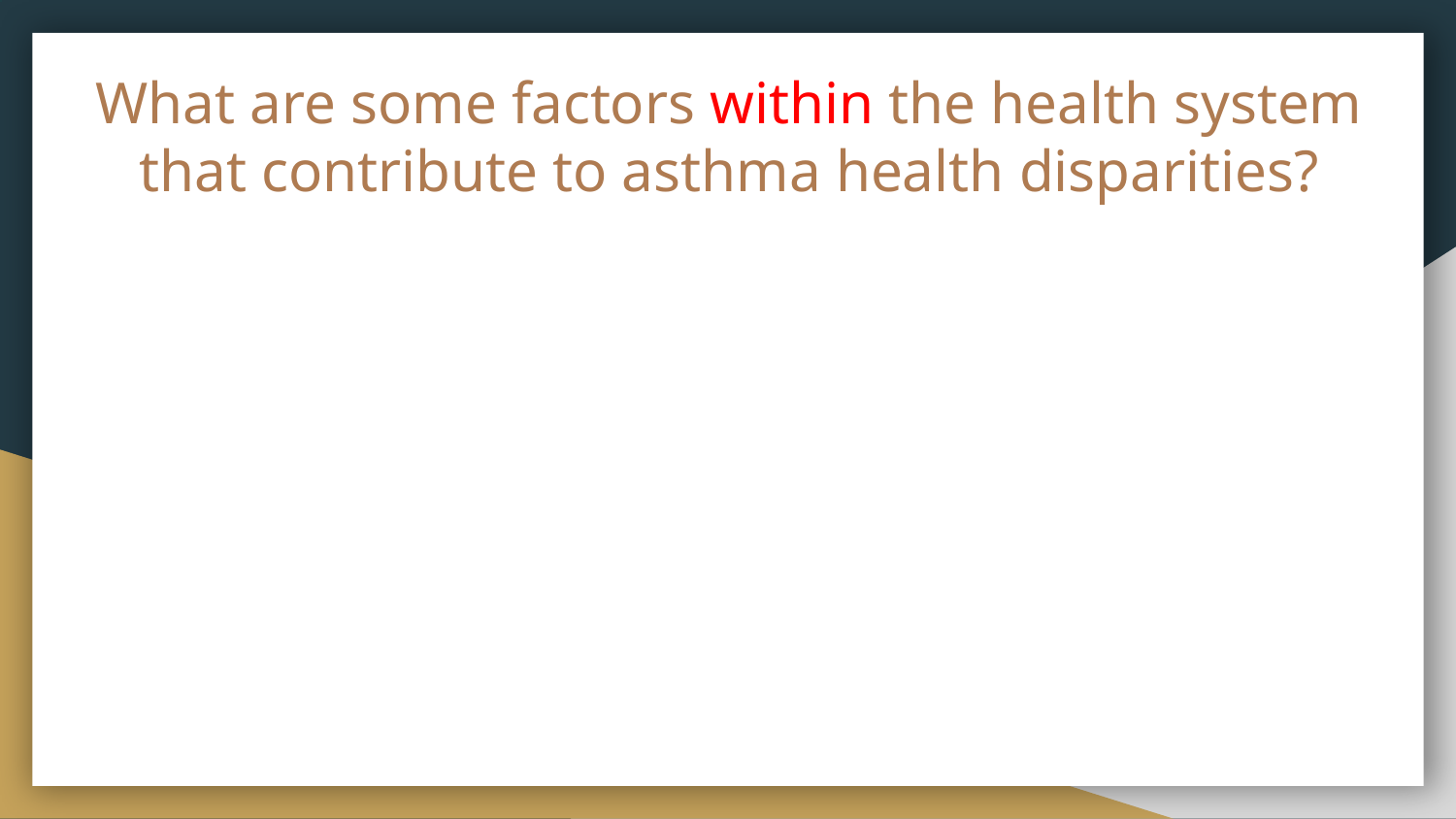

# What are some factors within the health system that contribute to asthma health disparities?

## Slide 19
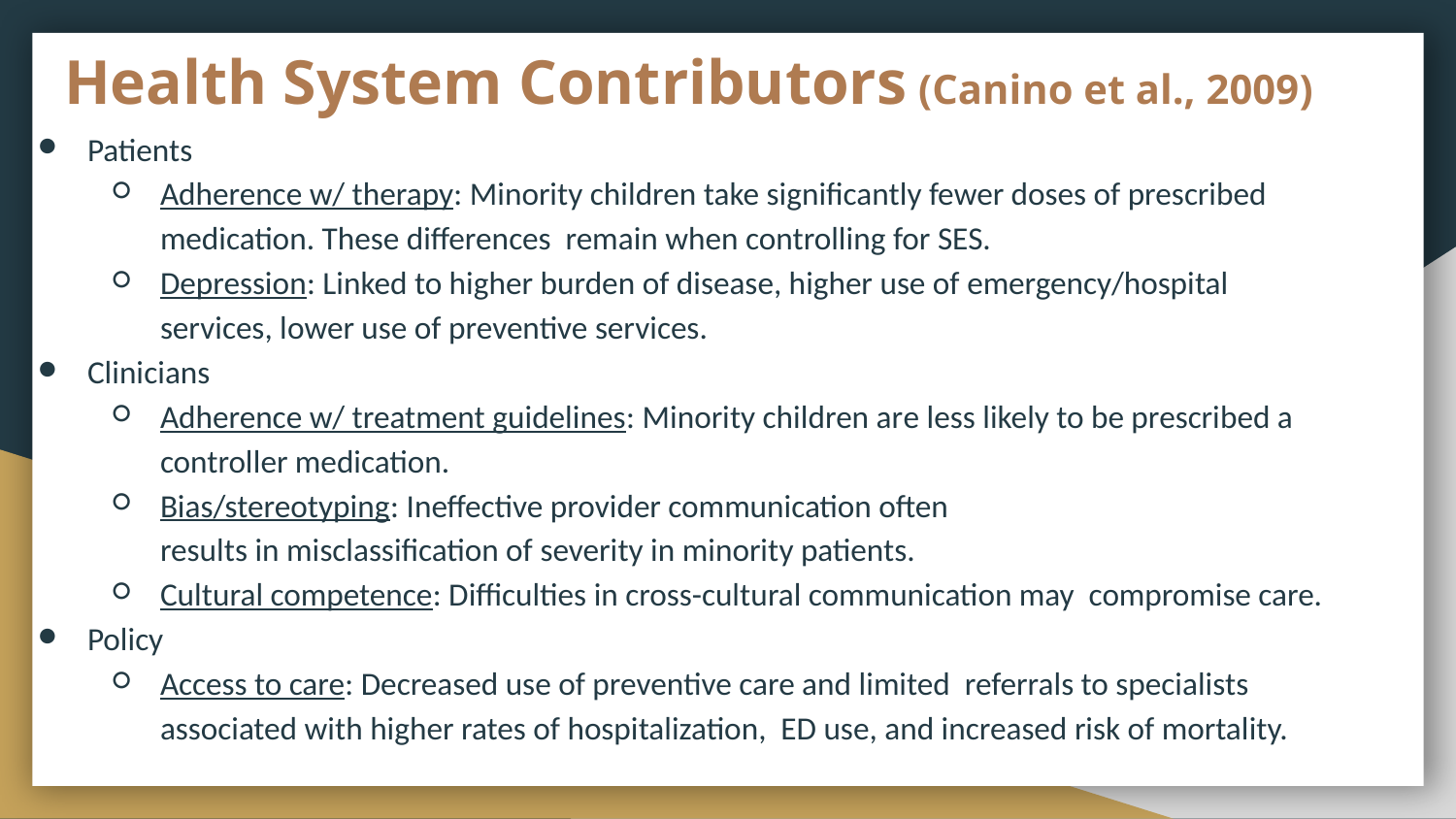

# Health System Contributors (Canino et al., 2009)
Patients
Adherence w/ therapy: Minority children take significantly fewer doses of prescribed medication. These differences remain when controlling for SES.
Depression: Linked to higher burden of disease, higher use of emergency/hospital services, lower use of preventive services.
Clinicians
Adherence w/ treatment guidelines: Minority children are less likely to be prescribed a controller medication.
Bias/stereotyping: Ineffective provider communication often
results in misclassification of severity in minority patients.
Cultural competence: Difficulties in cross-cultural communication may compromise care.
Policy
Access to care: Decreased use of preventive care and limited referrals to specialists associated with higher rates of hospitalization, ED use, and increased risk of mortality.

## Slide 20
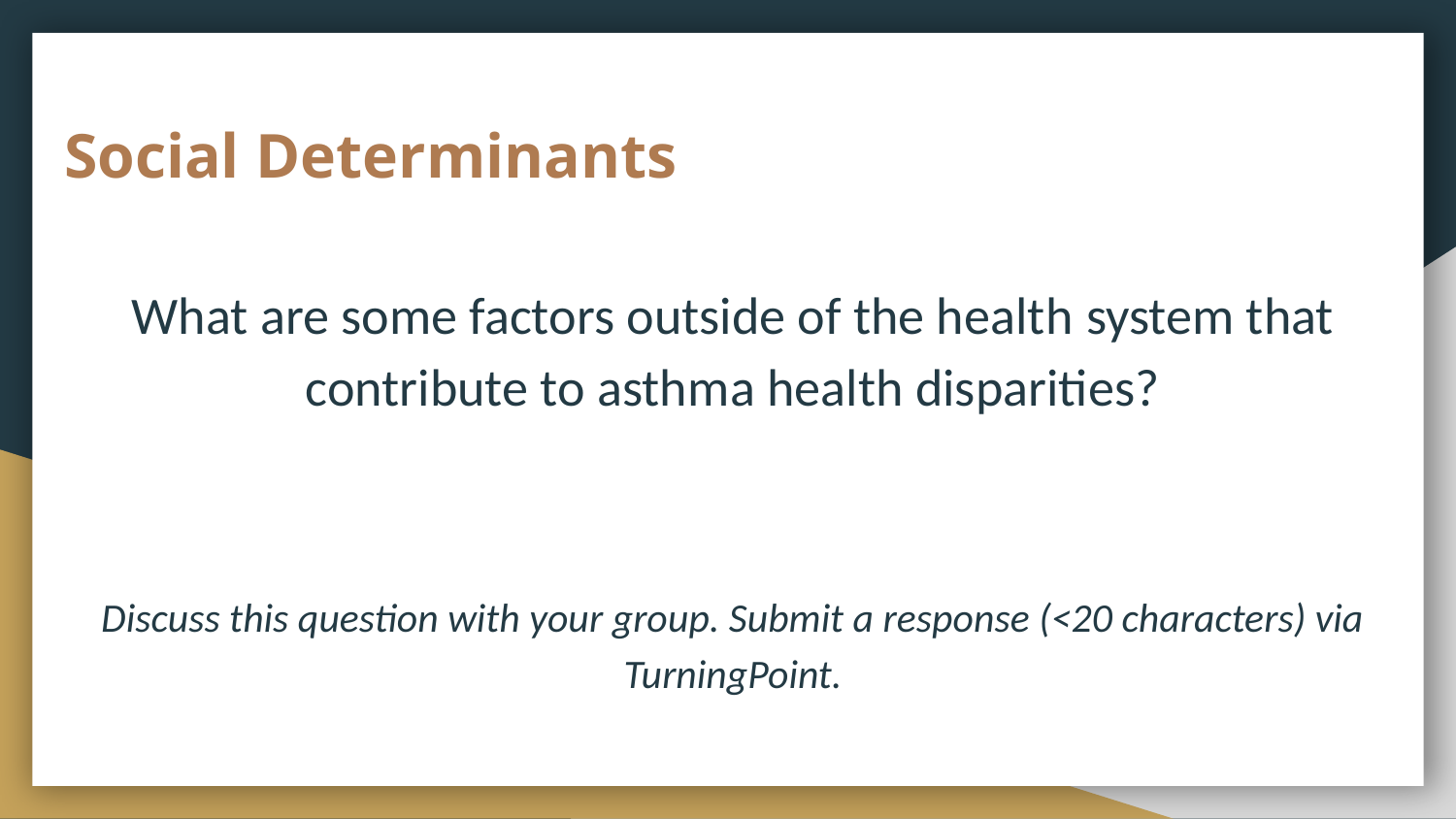

# Social Determinants
What are some factors outside of the health system that contribute to asthma health disparities?
Discuss this question with your group. Submit a response (<20 characters) via TurningPoint.

## Slide 21
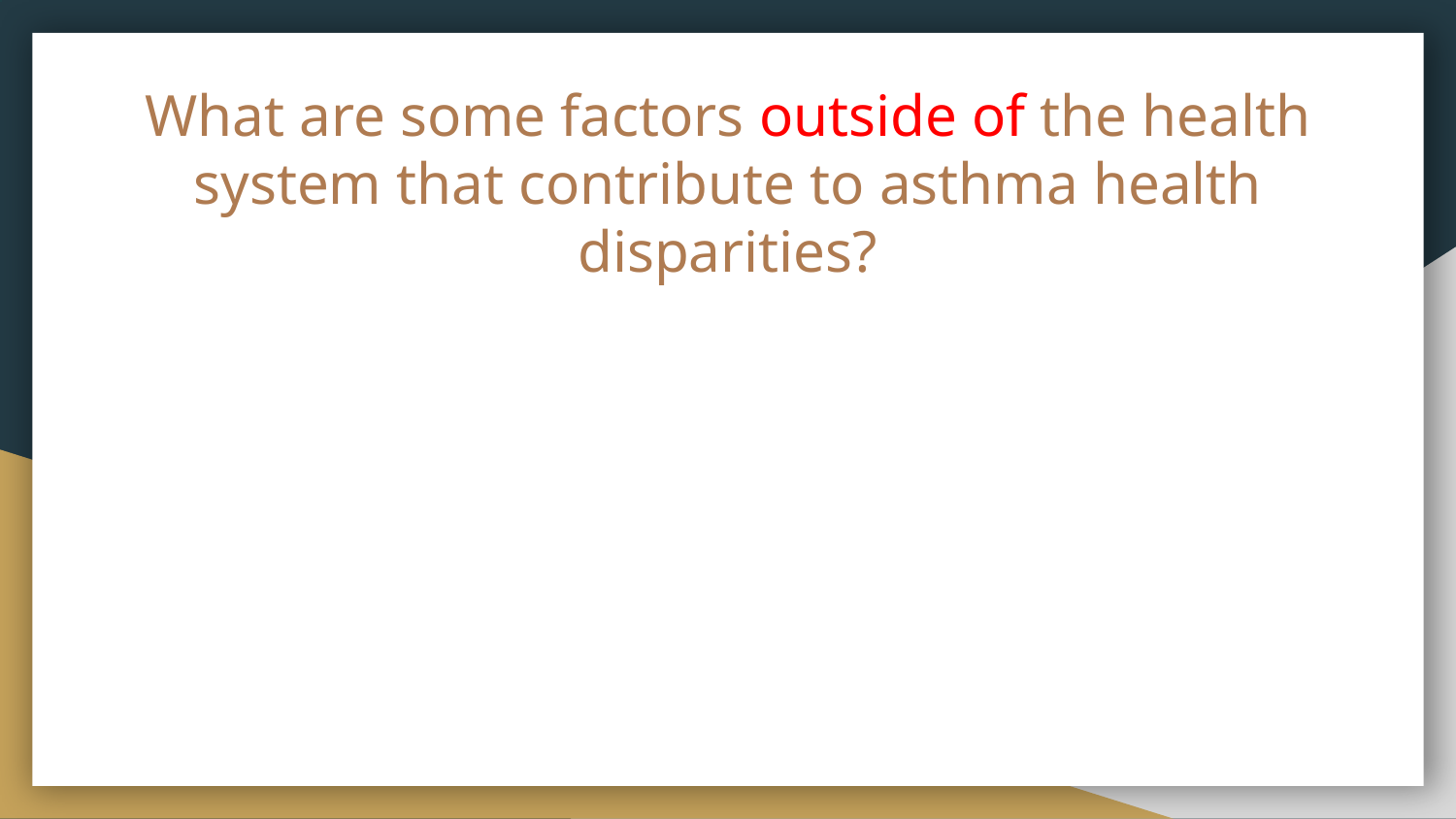

# What are some factors outside of the health system that contribute to asthma health disparities?

## Slide 22
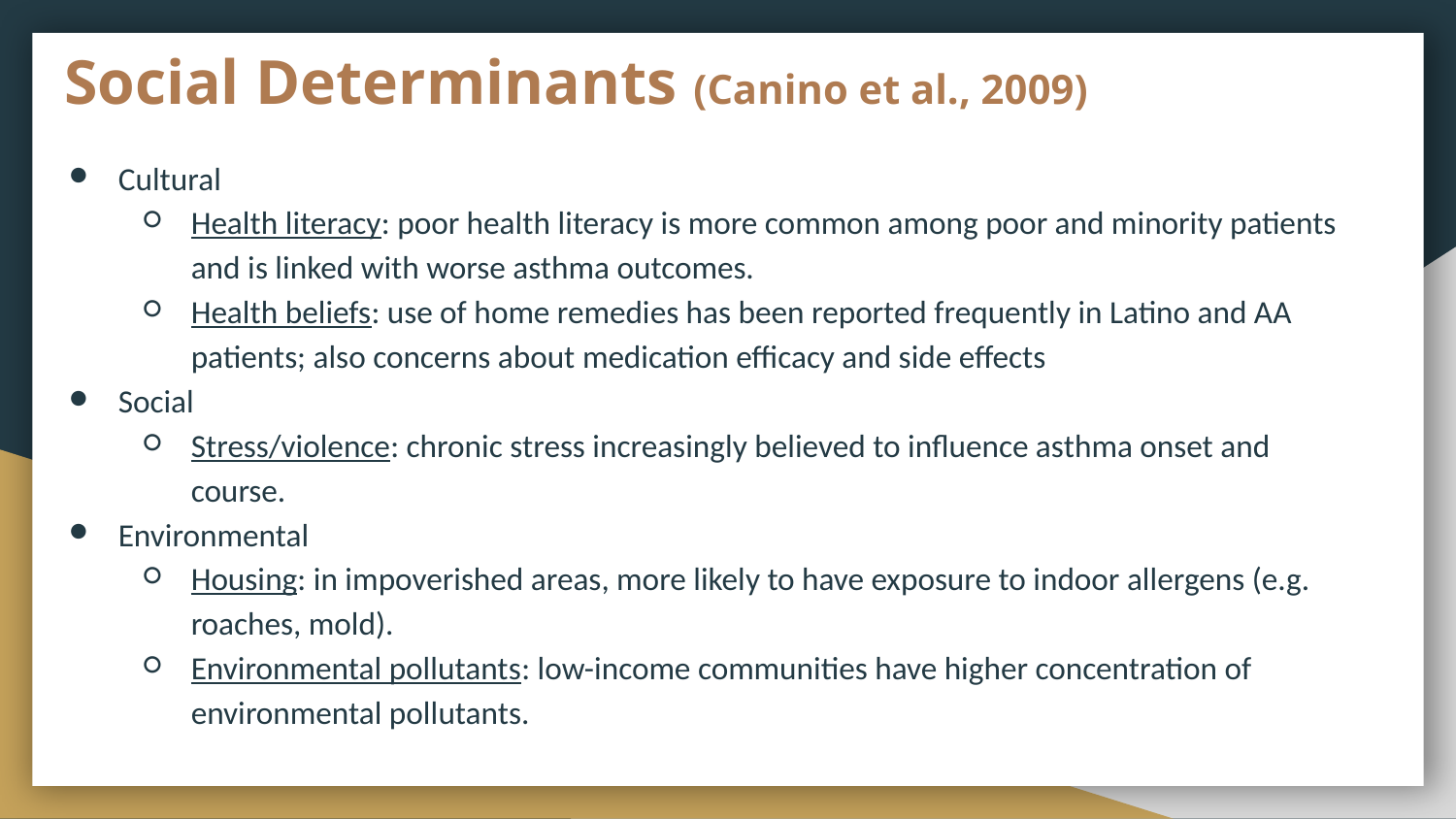

# Social Determinants (Canino et al., 2009)
Cultural
Health literacy: poor health literacy is more common among poor and minority patients and is linked with worse asthma outcomes.
Health beliefs: use of home remedies has been reported frequently in Latino and AA patients; also concerns about medication efficacy and side effects
Social
Stress/violence: chronic stress increasingly believed to influence asthma onset and course.
Environmental
Housing: in impoverished areas, more likely to have exposure to indoor allergens (e.g. roaches, mold).
Environmental pollutants: low-income communities have higher concentration of environmental pollutants.

## Slide 23
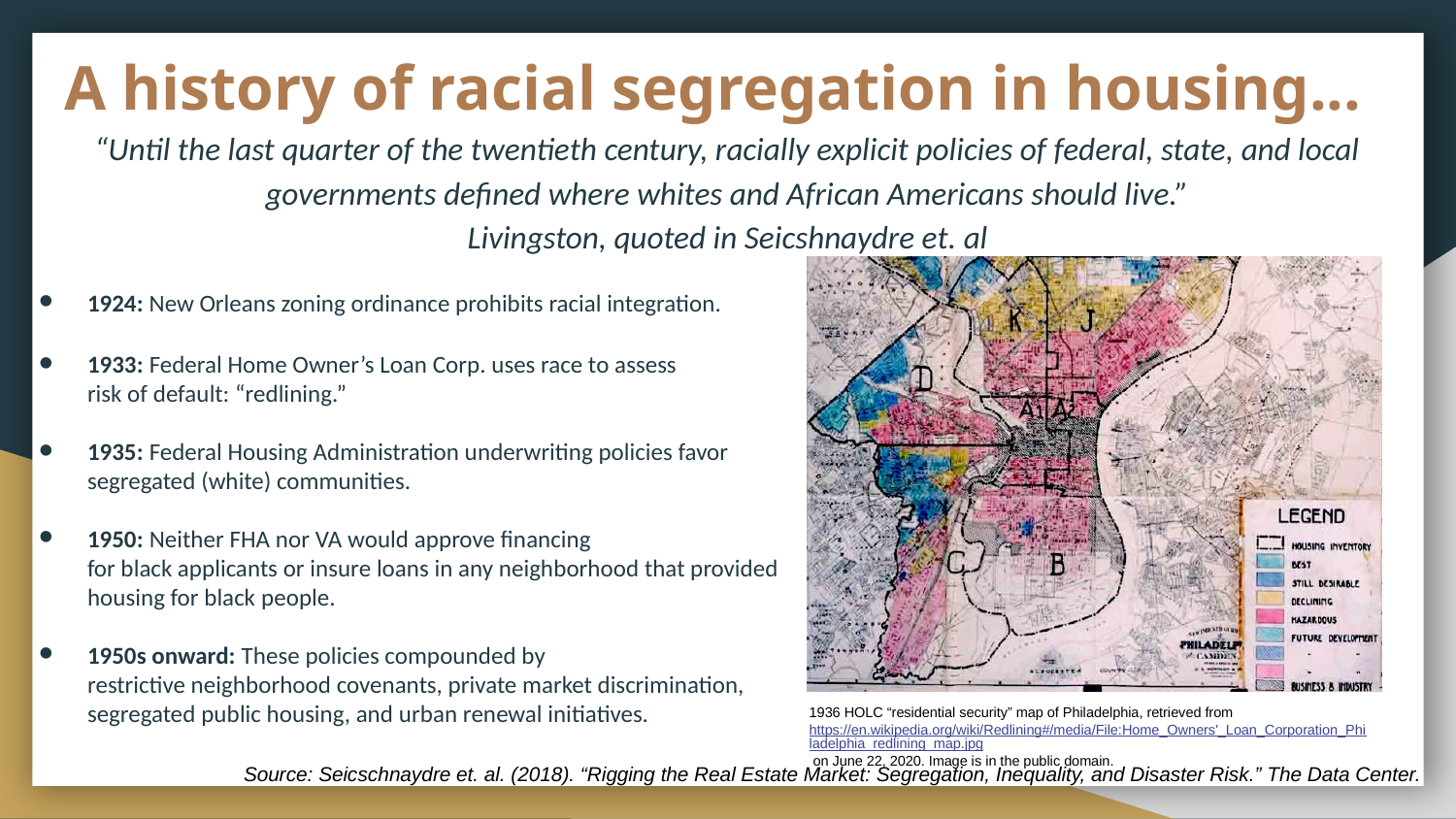

# A history of racial segregation in housing...
“Until the last quarter of the twentieth century, racially explicit policies of federal, state, and local governments defined where whites and African Americans should live.”
Livingston, quoted in Seicshnaydre et. al
1924: New Orleans zoning ordinance prohibits racial integration.
1933: Federal Home Owner’s Loan Corp. uses race to assess
risk of default: “redlining.”
1935: Federal Housing Administration underwriting policies favor
segregated (white) communities.
1950: Neither FHA nor VA would approve financing
for black applicants or insure loans in any neighborhood that provided
housing for black people.
1950s onward: These policies compounded by
restrictive neighborhood covenants, private market discrimination,
segregated public housing, and urban renewal initiatives.
1936 HOLC “residential security” map of Philadelphia, retrieved from https://en.wikipedia.org/wiki/Redlining#/media/File:Home_Owners'_Loan_Corporation_Philadelphia_redlining_map.jpg on June 22, 2020. Image is in the public domain.
Source: Seicschnaydre et. al. (2018). “Rigging the Real Estate Market: Segregation, Inequality, and Disaster Risk.” The Data Center.

## Slide 24
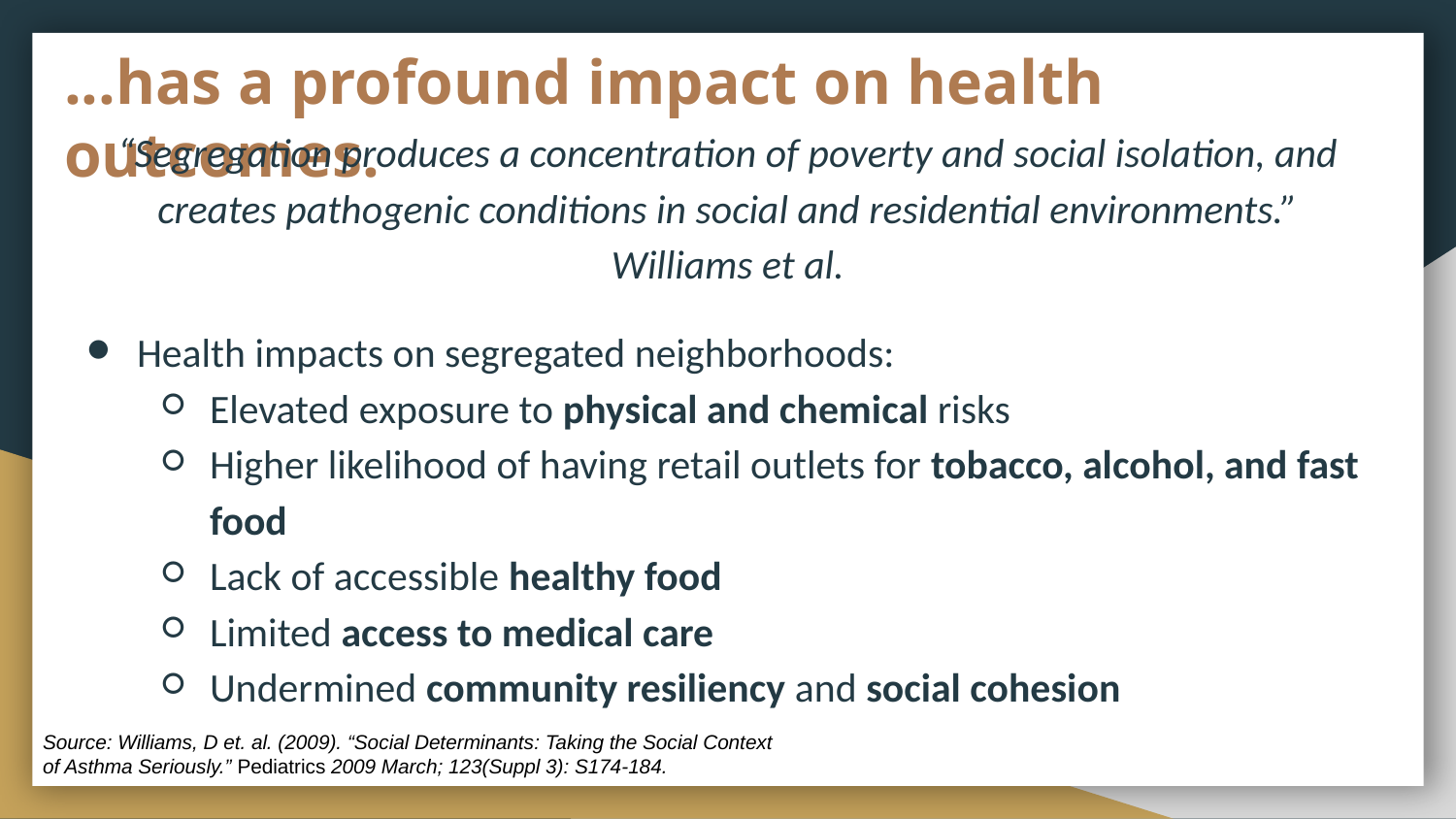

# ...has a profound impact on health outcomes.
“Segregation produces a concentration of poverty and social isolation, and creates pathogenic conditions in social and residential environments.”
Williams et al.
Health impacts on segregated neighborhoods:
Elevated exposure to physical and chemical risks
Higher likelihood of having retail outlets for tobacco, alcohol, and fast food
Lack of accessible healthy food
Limited access to medical care
Undermined community resiliency and social cohesion
Source: Williams, D et. al. (2009). “Social Determinants: Taking the Social Context of Asthma Seriously.” Pediatrics 2009 March; 123(Suppl 3): S174-184.

## Slide 25
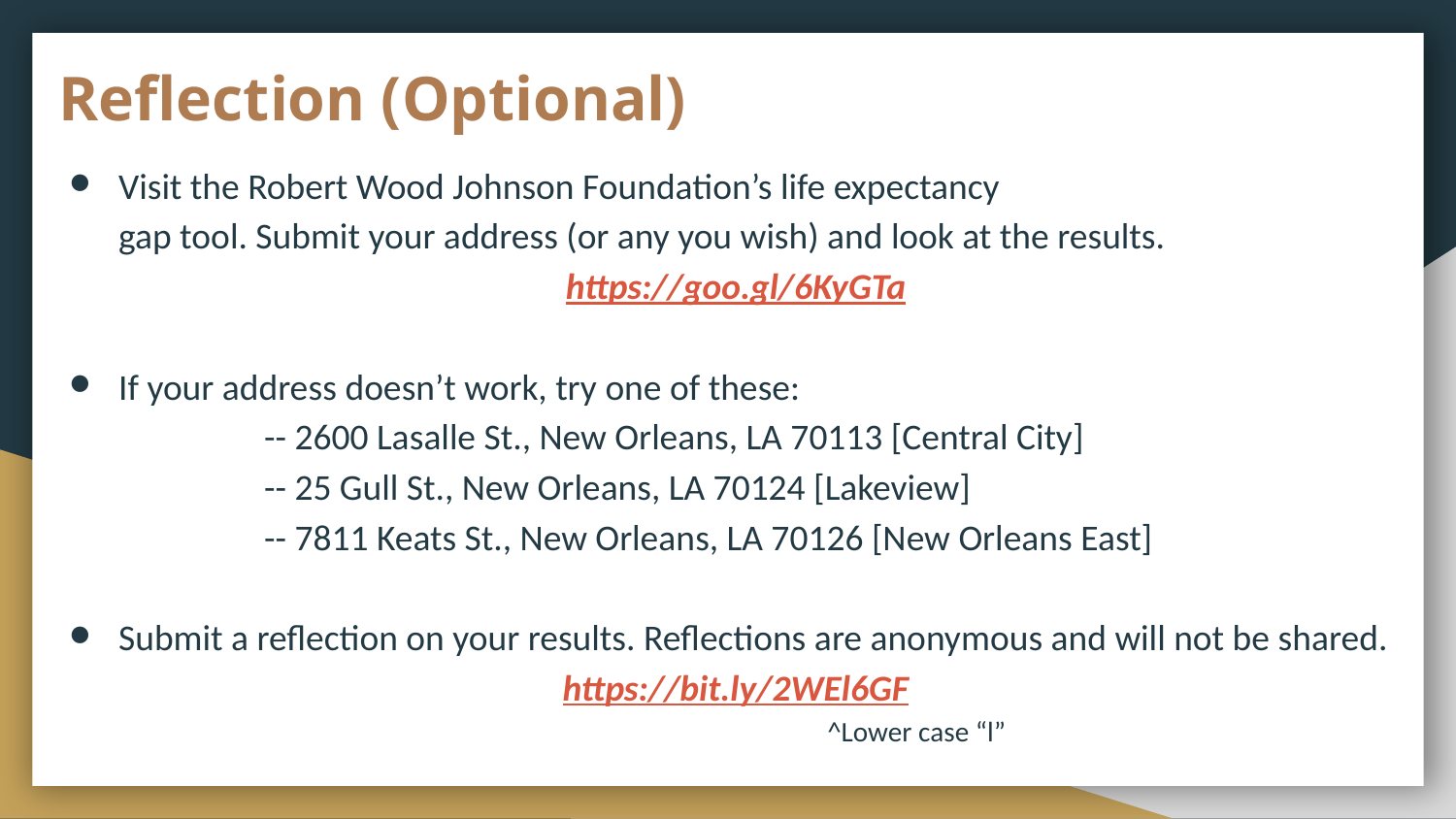

# Reflection (Optional)
Visit the Robert Wood Johnson Foundation’s life expectancy
gap tool. Submit your address (or any you wish) and look at the results.
https://goo.gl/6KyGTa
If your address doesn’t work, try one of these:
	-- 2600 Lasalle St., New Orleans, LA 70113 [Central City]
 	-- 25 Gull St., New Orleans, LA 70124 [Lakeview]
	-- 7811 Keats St., New Orleans, LA 70126 [New Orleans East]
Submit a reflection on your results. Reflections are anonymous and will not be shared.
https://bit.ly/2WEl6GF
 ^Lower case “l”

## Slide 26
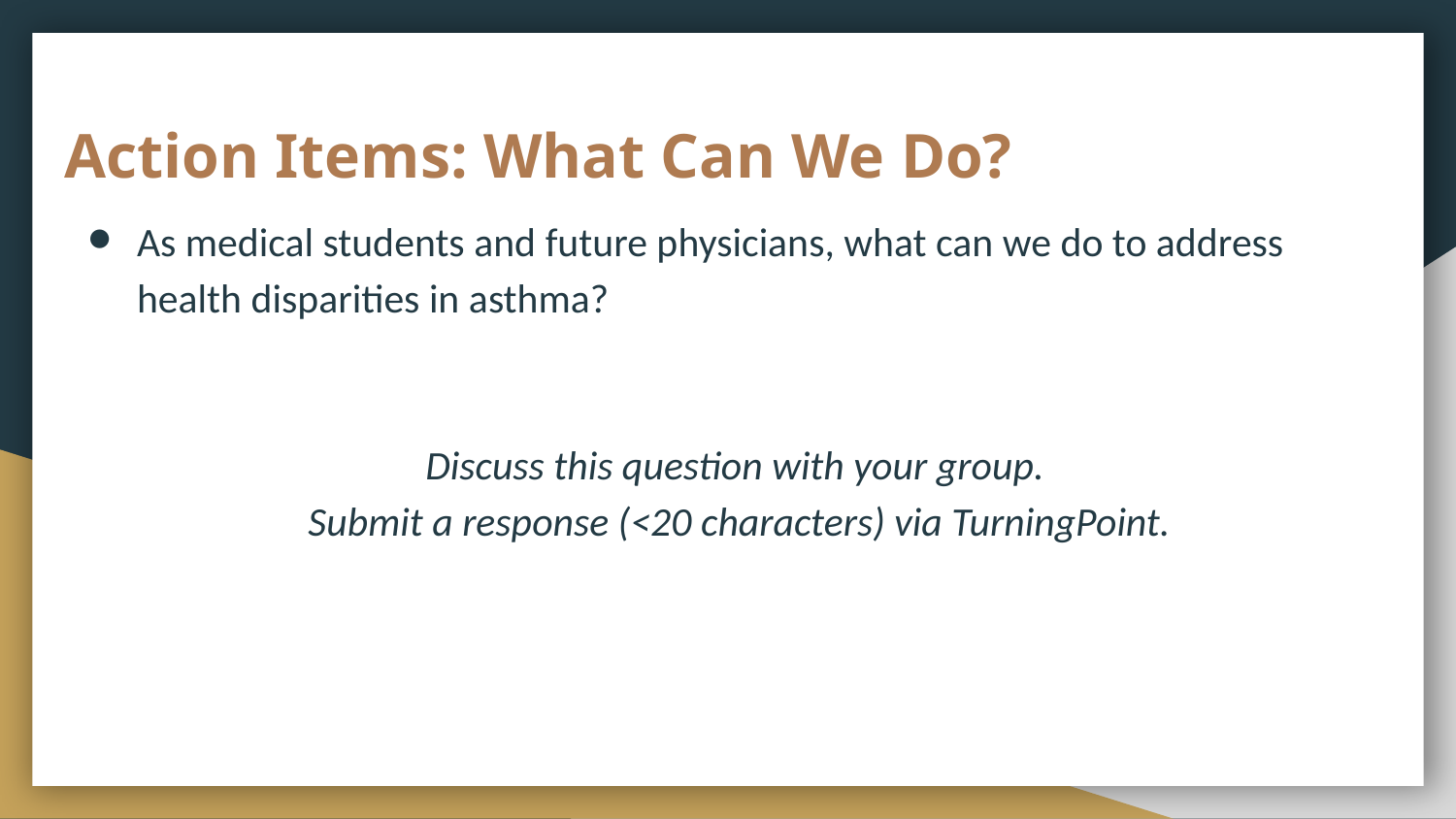

# Action Items: What Can We Do?
As medical students and future physicians, what can we do to address health disparities in asthma?
Discuss this question with your group.
Submit a response (<20 characters) via TurningPoint.

## Slide 27
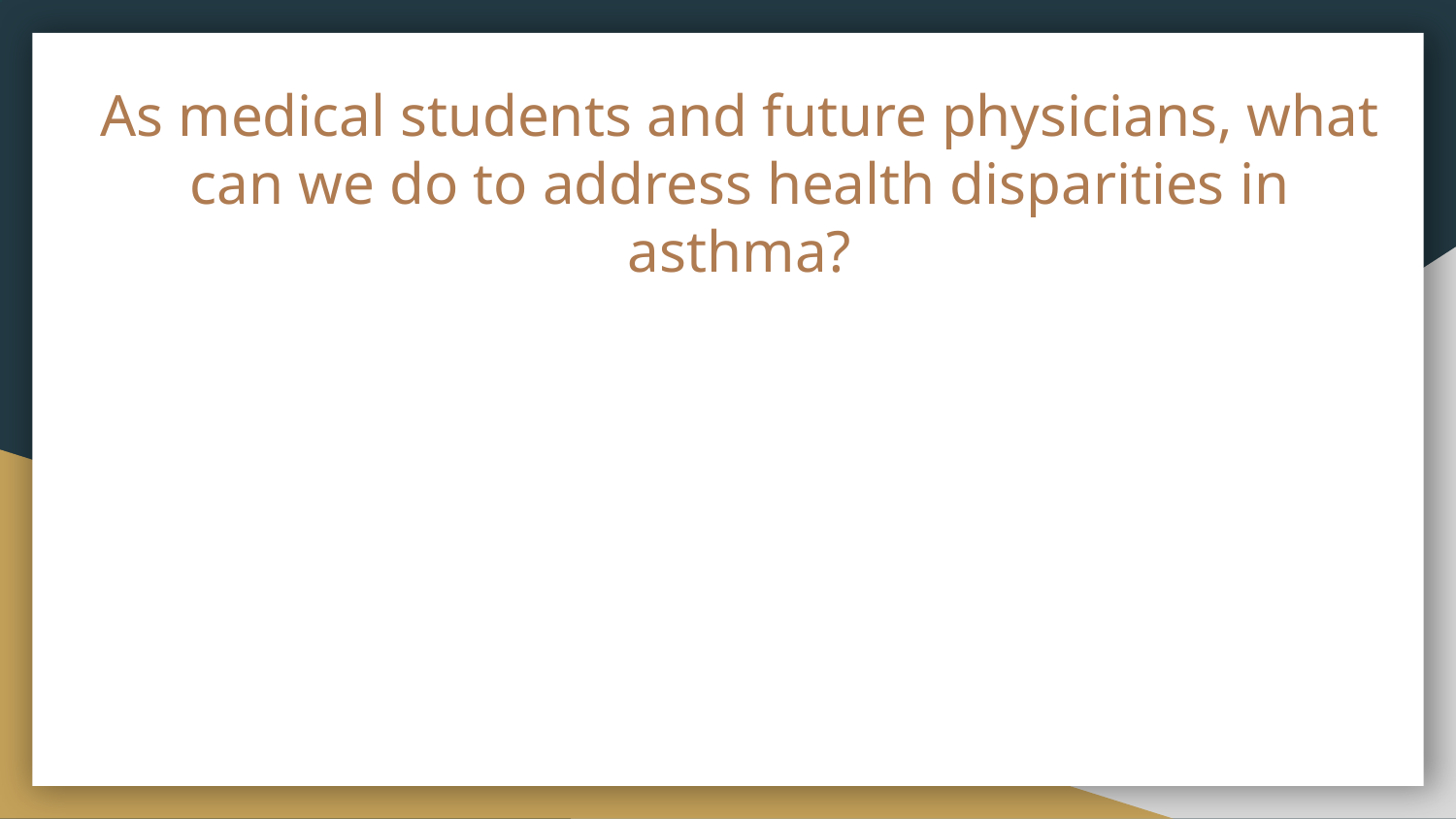

# As medical students and future physicians, what can we do to address health disparities in asthma?

## Slide 28
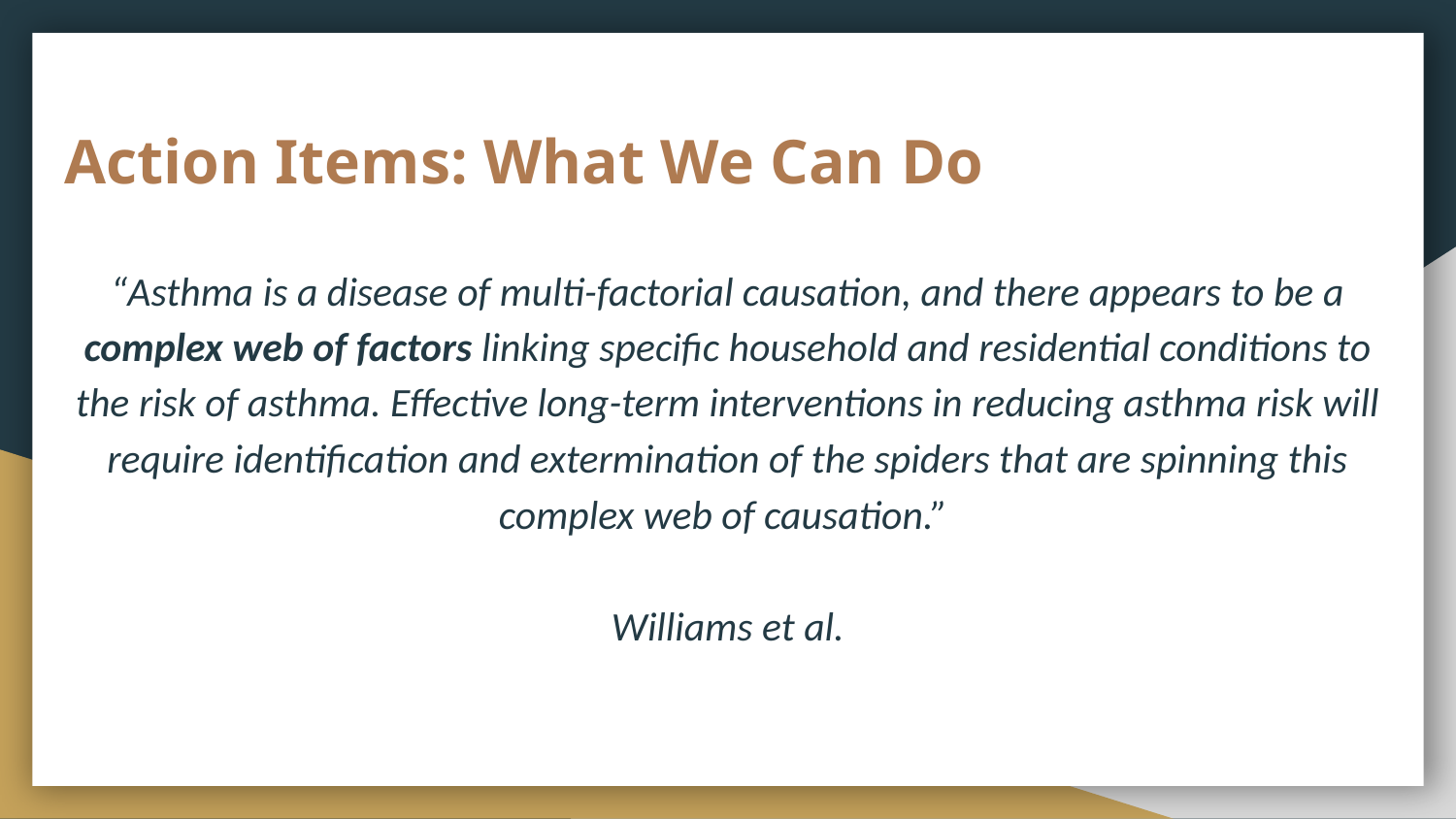

# Action Items: What We Can Do
“Asthma is a disease of multi-factorial causation, and there appears to be a complex web of factors linking specific household and residential conditions to the risk of asthma. Effective long-term interventions in reducing asthma risk will require identification and extermination of the spiders that are spinning this complex web of causation.”
Williams et al.

## Slide 29
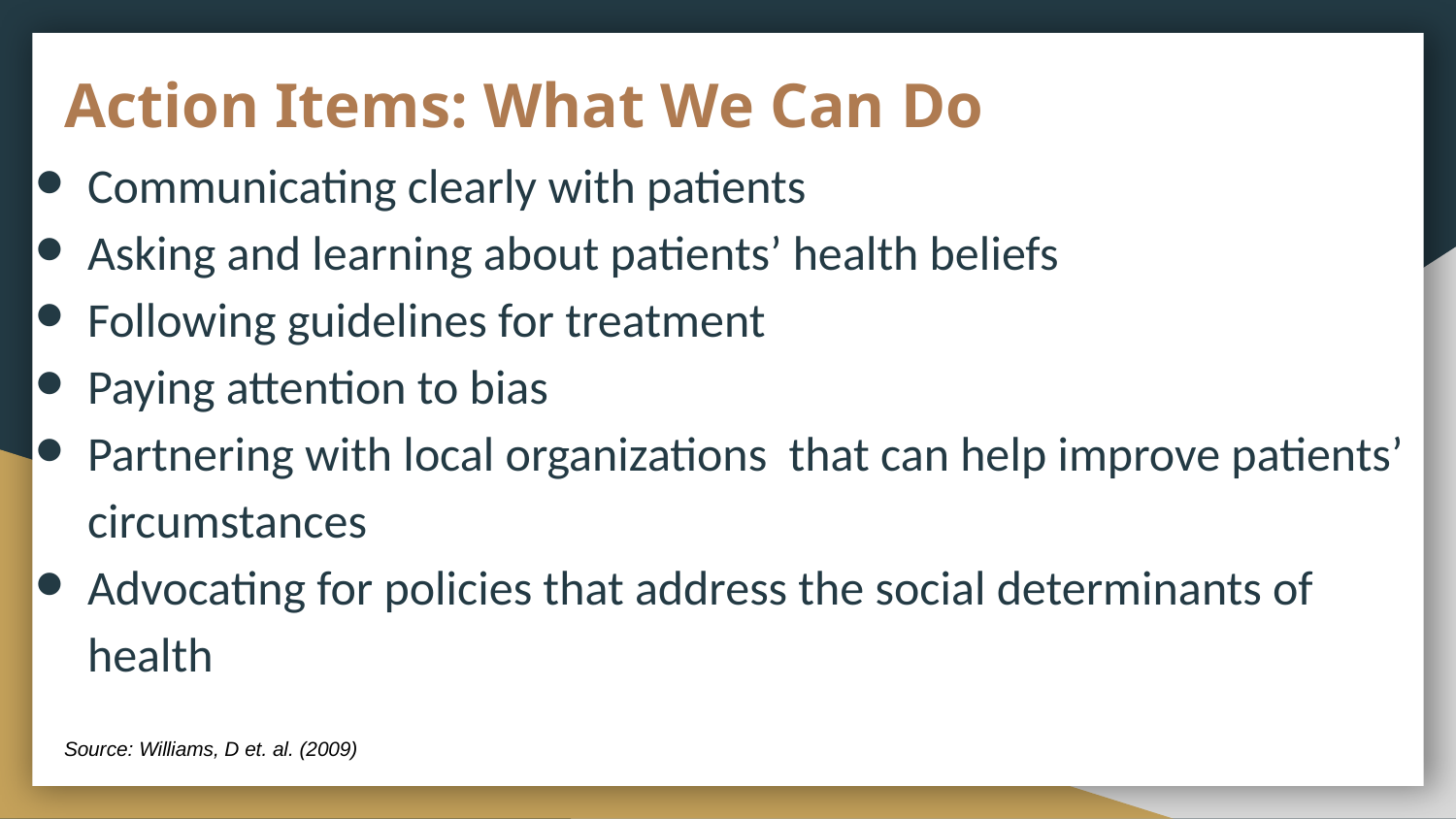

# Action Items: What We Can Do
Communicating clearly with patients
Asking and learning about patients’ health beliefs
Following guidelines for treatment
Paying attention to bias
Partnering with local organizations that can help improve patients’ circumstances
Advocating for policies that address the social determinants of health
Source: Williams, D et. al. (2009)

## Slide 30
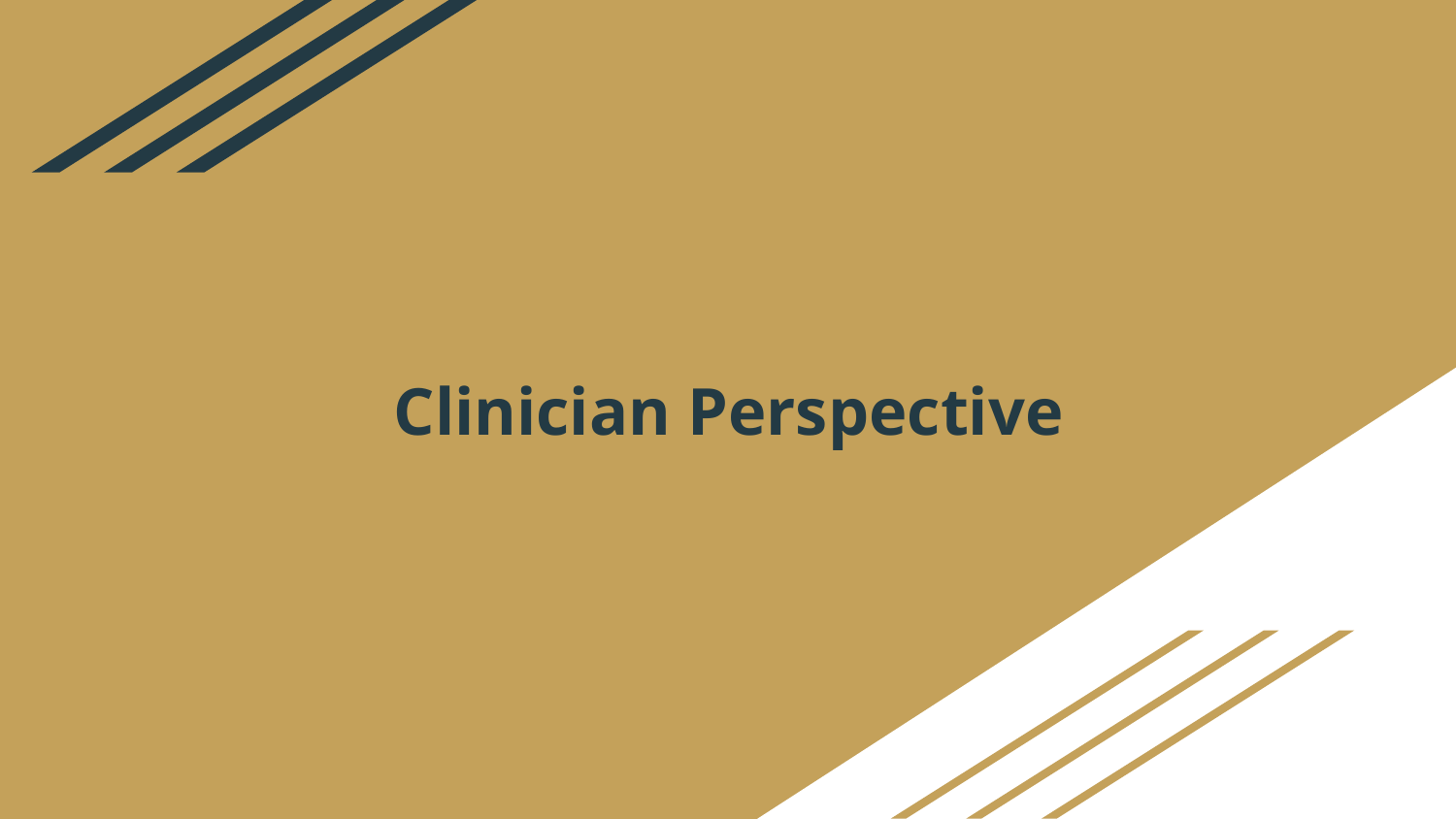

# Clinician Perspective

## Slide 31
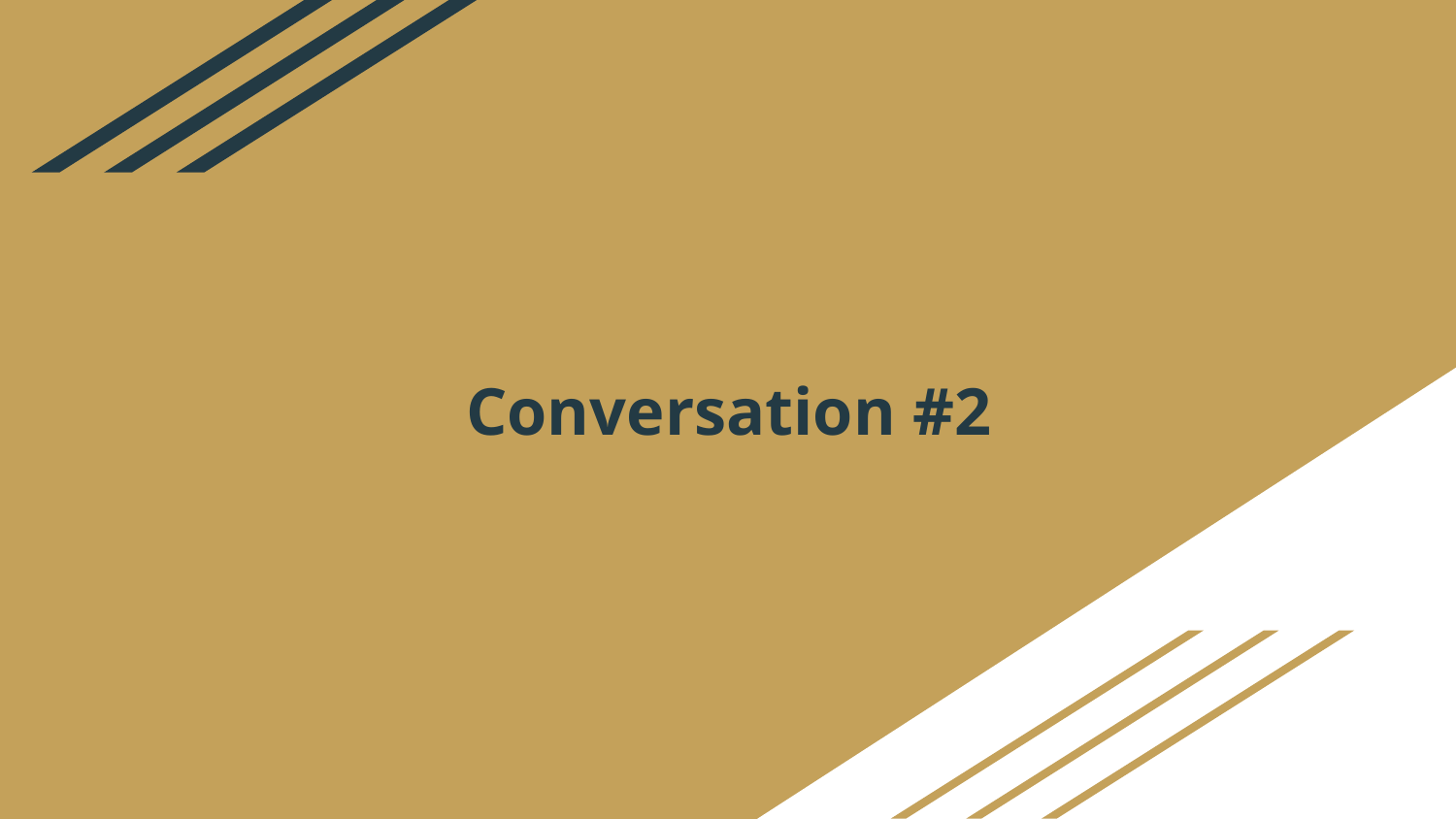

# Conversation #2

## Slide 32
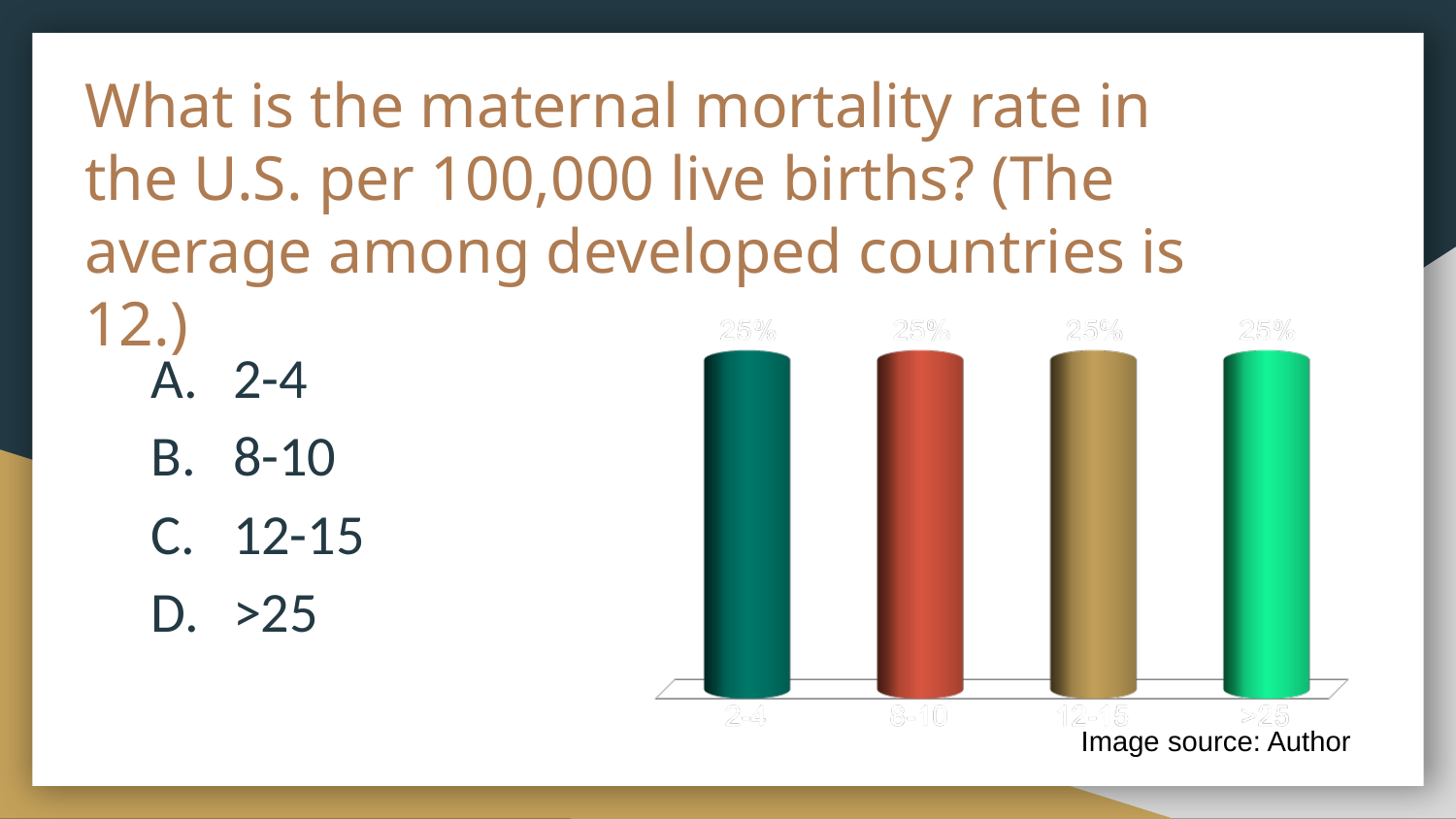

# What is the maternal mortality rate in the U.S. per 100,000 live births? (The average among developed countries is 12.)
2-4
8-10
12-15
>25
Image source: Author

## Slide 33
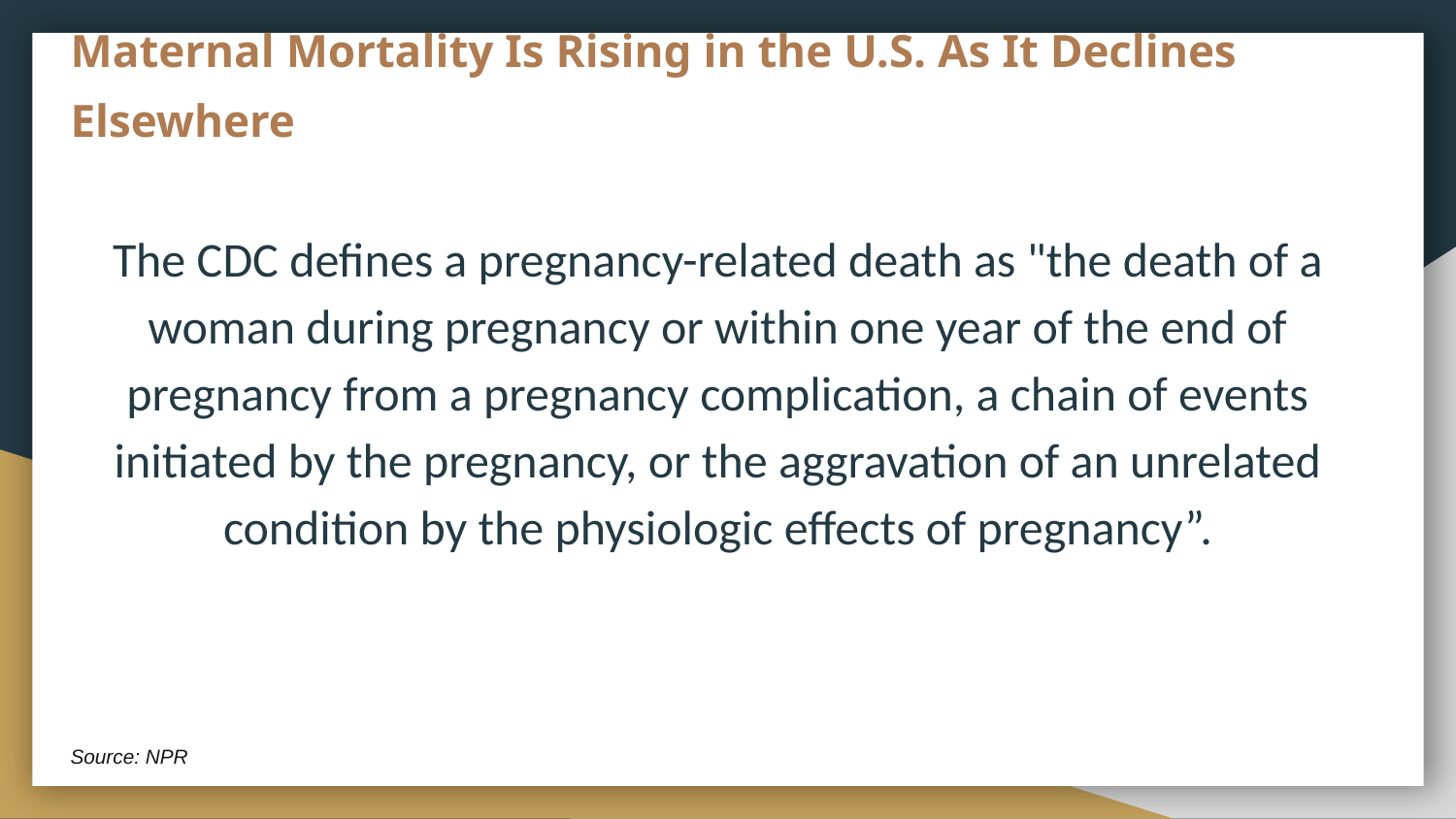

# Maternal Mortality Is Rising in the U.S. As It Declines Elsewhere
The CDC defines a pregnancy-related death as "the death of a woman during pregnancy or within one year of the end of pregnancy from a pregnancy complication, a chain of events initiated by the pregnancy, or the aggravation of an unrelated condition by the physiologic effects of pregnancy”.
Source: NPR

## Slide 34
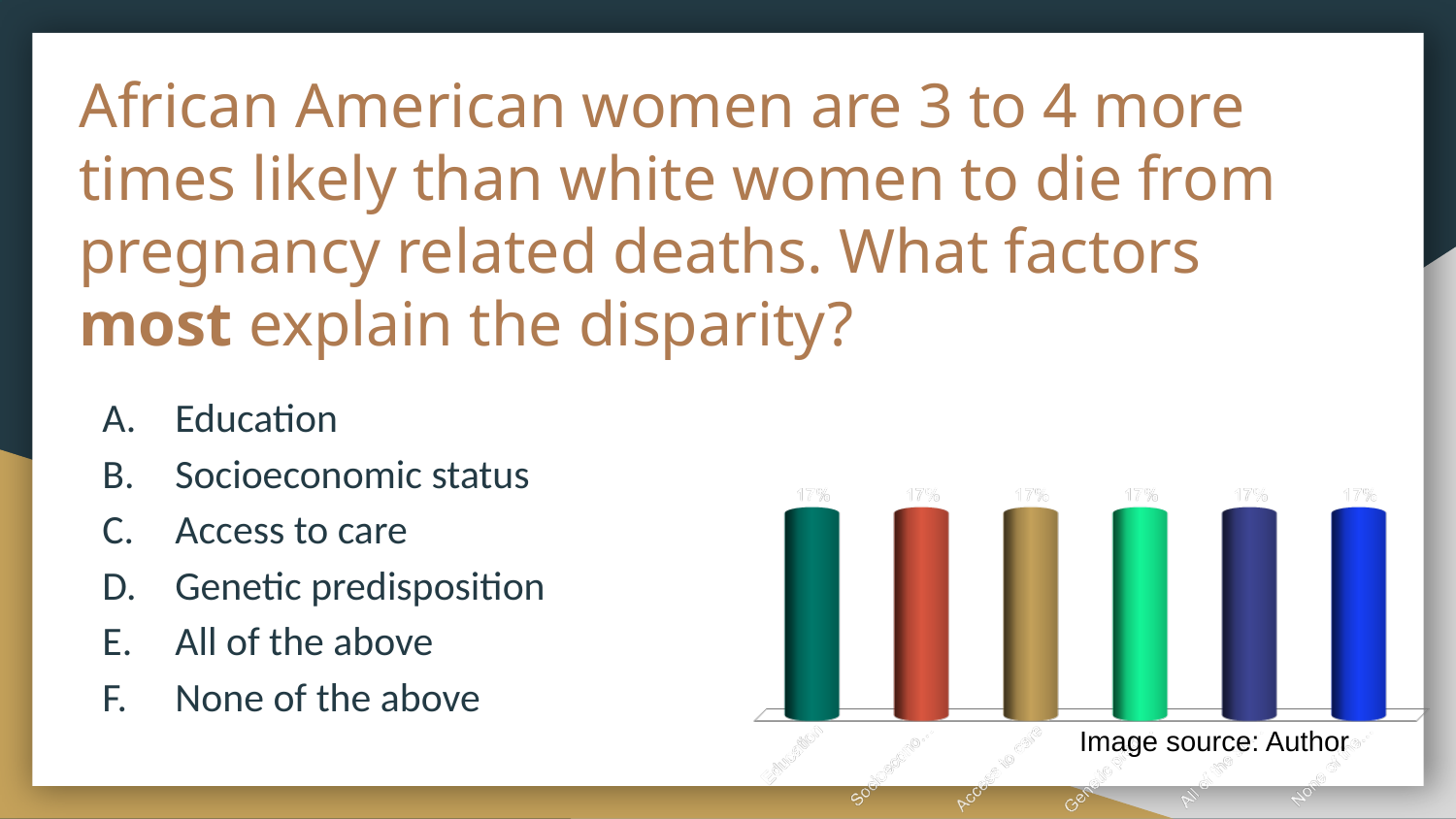

# African American women are 3 to 4 more times likely than white women to die from pregnancy related deaths. What factors most explain the disparity?
Education
Socioeconomic status
Access to care
Genetic predisposition
All of the above
None of the above
Image source: Author

## Slide 35
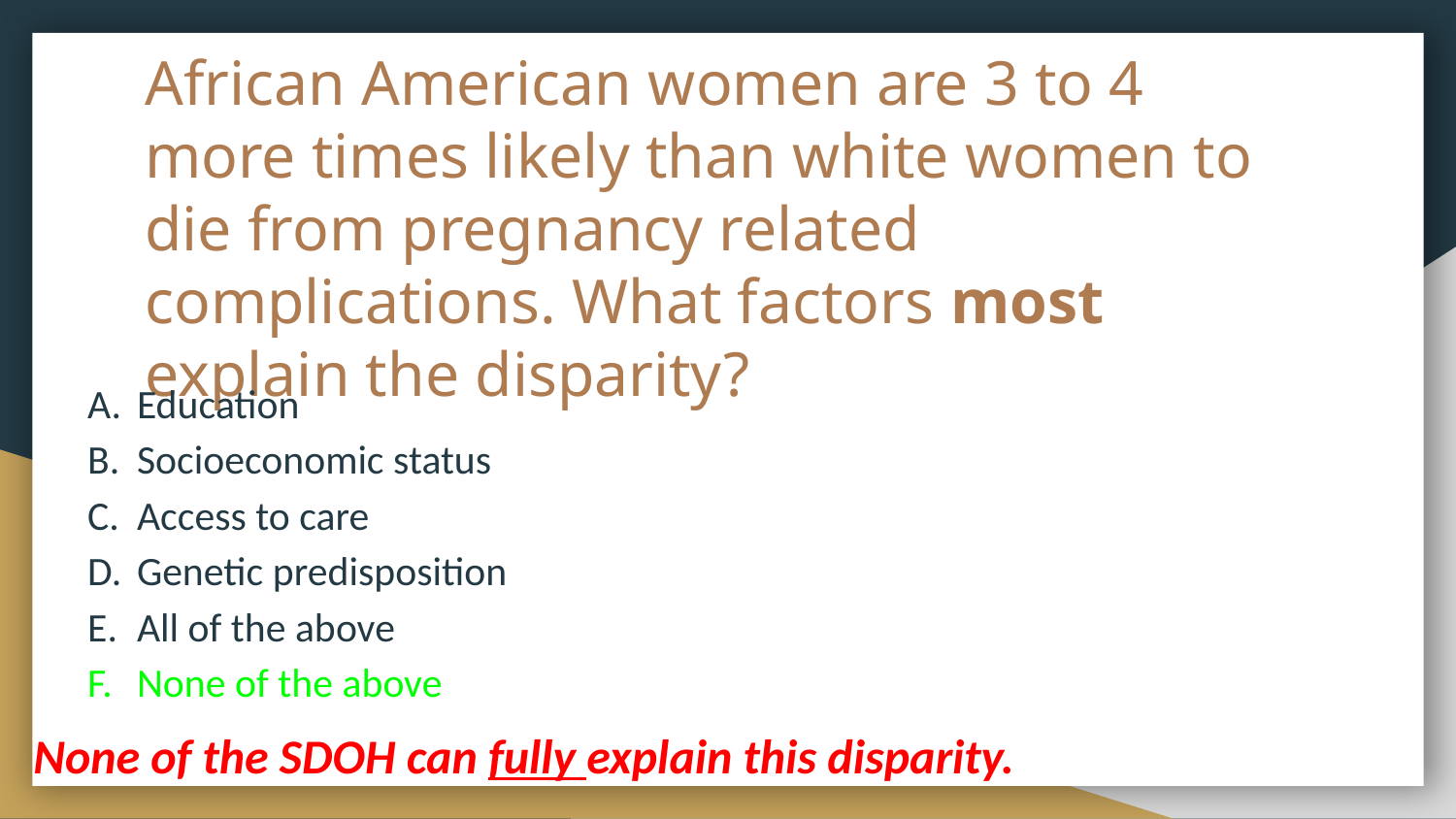

# African American women are 3 to 4 more times likely than white women to die from pregnancy related complications. What factors most explain the disparity?
Education
Socioeconomic status
Access to care
Genetic predisposition
All of the above
None of the above
None of the SDOH can fully explain this disparity.

## Slide 36
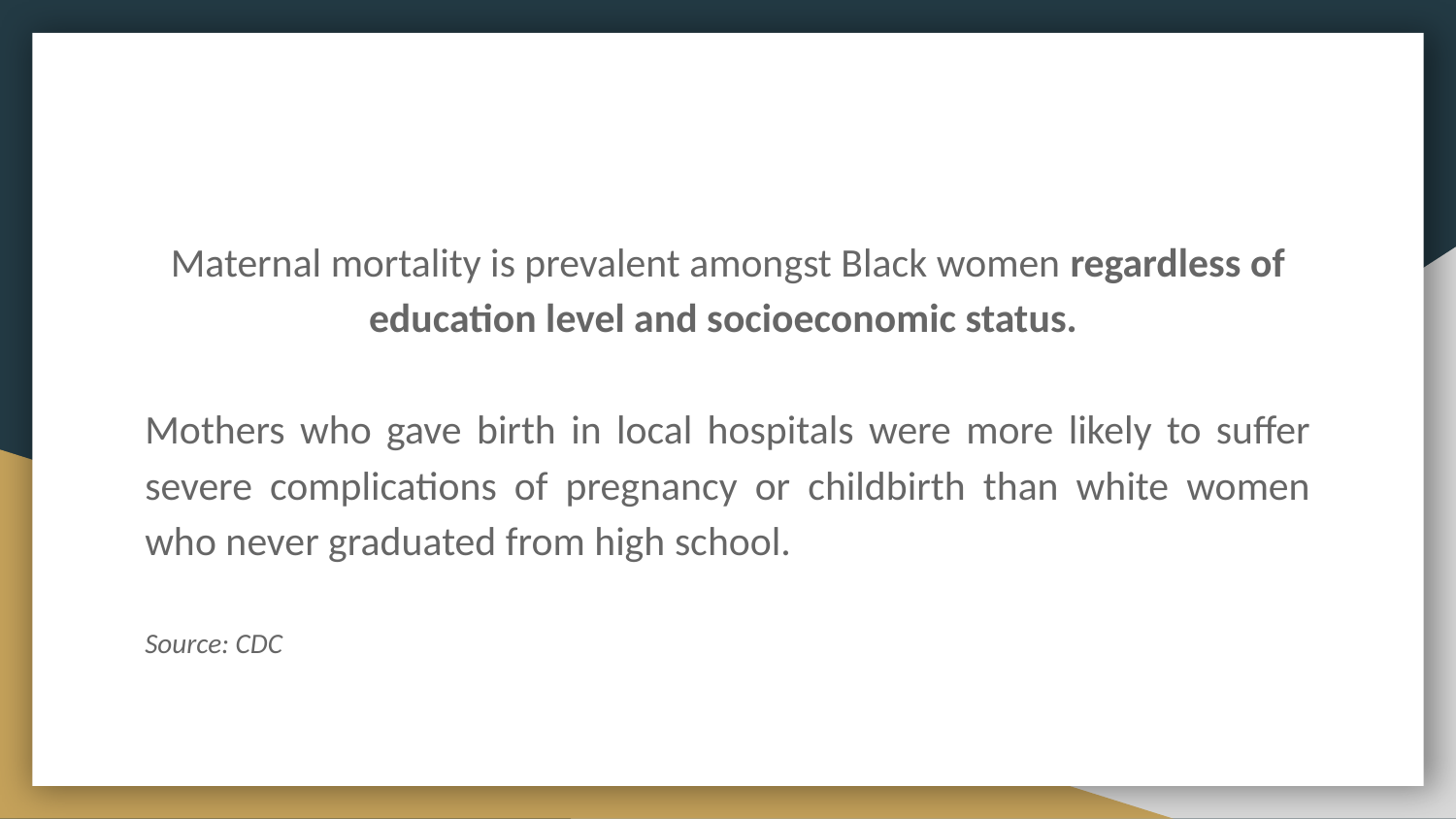

Maternal mortality is prevalent amongst Black women regardless of education level and socioeconomic status.
Mothers who gave birth in local hospitals were more likely to suffer severe complications of pregnancy or childbirth than white women who never graduated from high school.
Source: CDC

## Slide 37
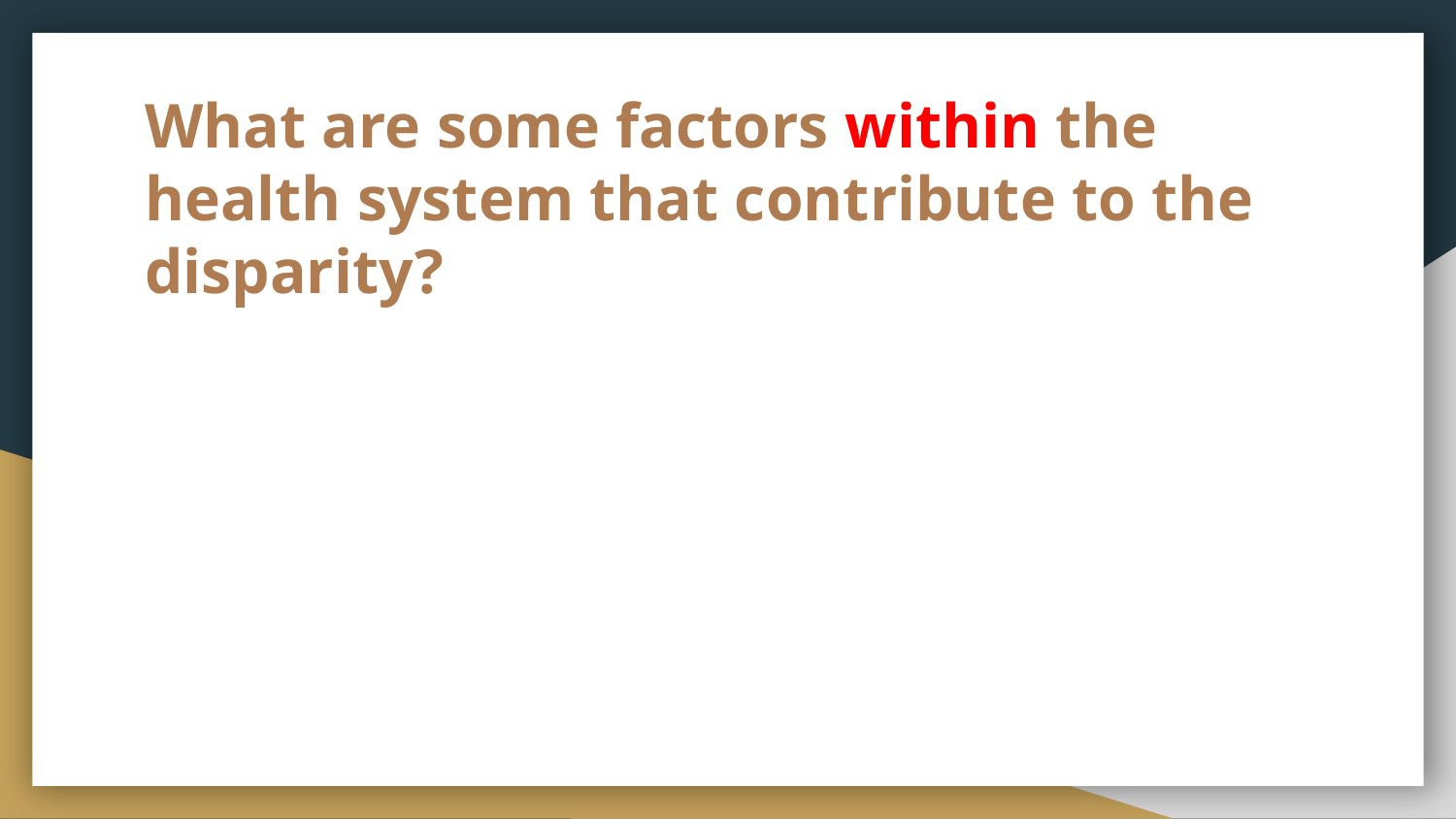

# What are some factors within the health system that contribute to the disparity?

## Slide 38
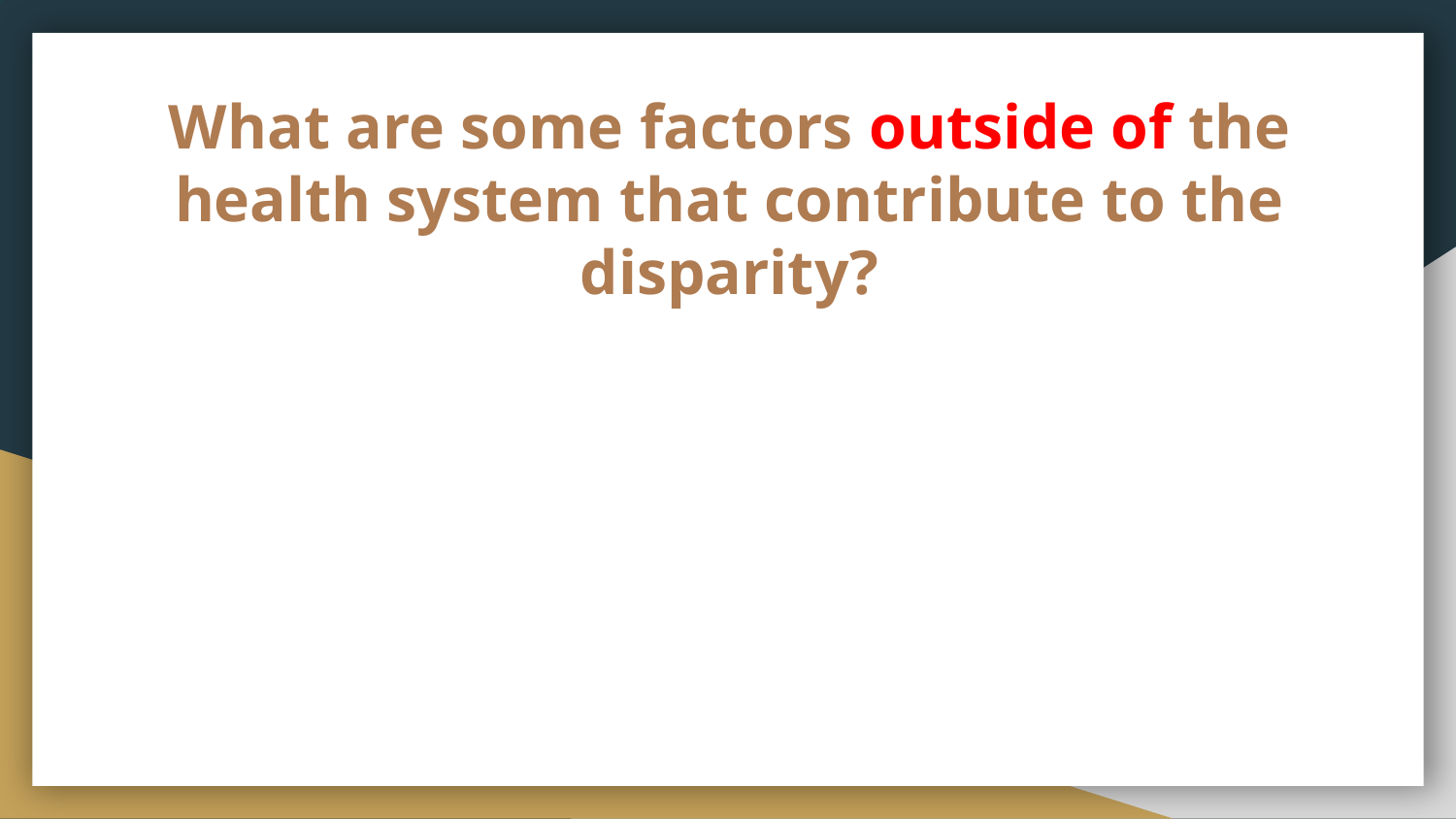

# What are some factors outside of the health system that contribute to the disparity?

## Slide 39
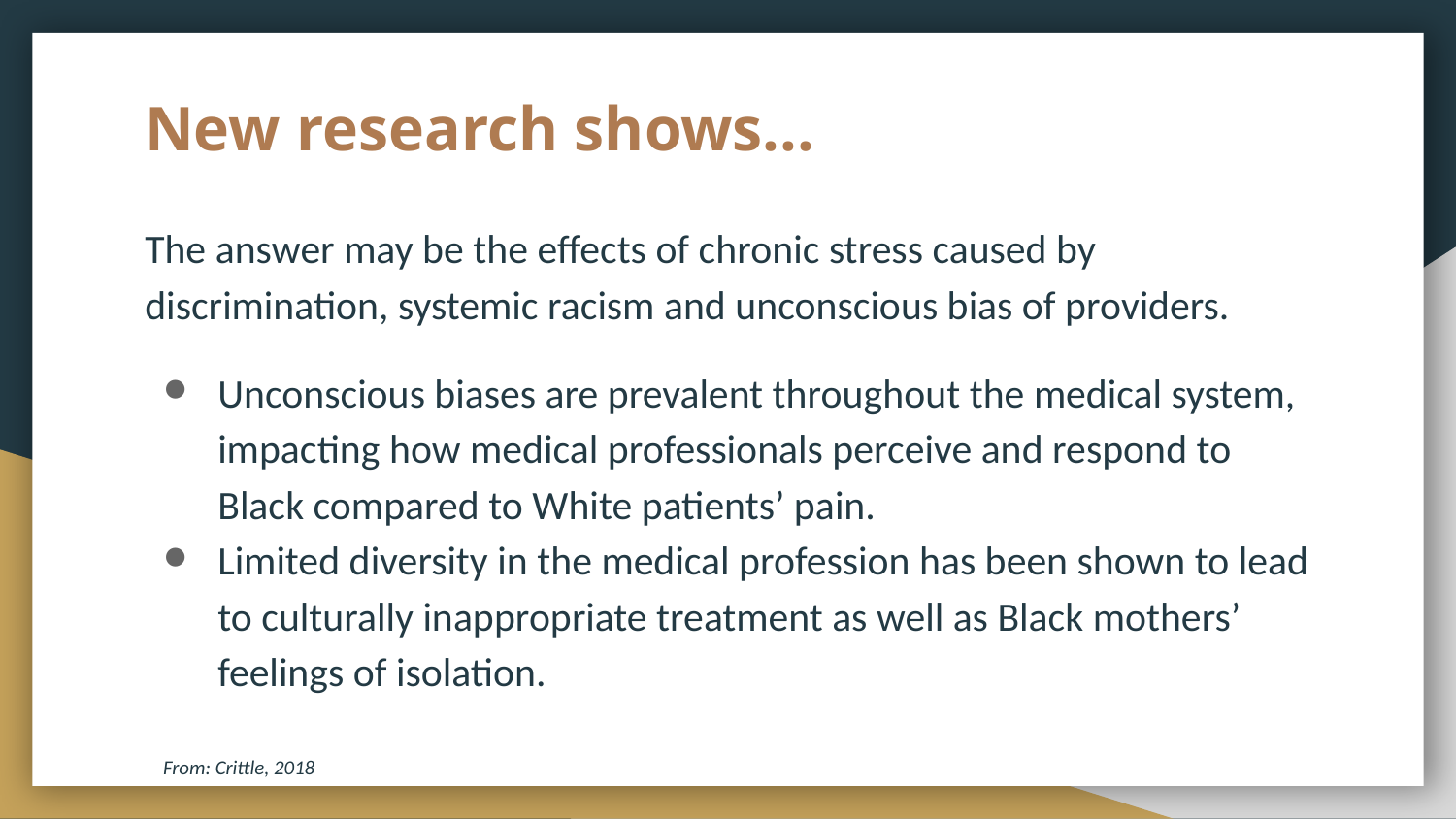

# New research shows...
The answer may be the effects of chronic stress caused by discrimination, systemic racism and unconscious bias of providers.
Unconscious biases are prevalent throughout the medical system, impacting how medical professionals perceive and respond to Black compared to White patients’ pain.
Limited diversity in the medical profession has been shown to lead to culturally inappropriate treatment as well as Black mothers’ feelings of isolation.
From: Crittle, 2018

## Slide 40
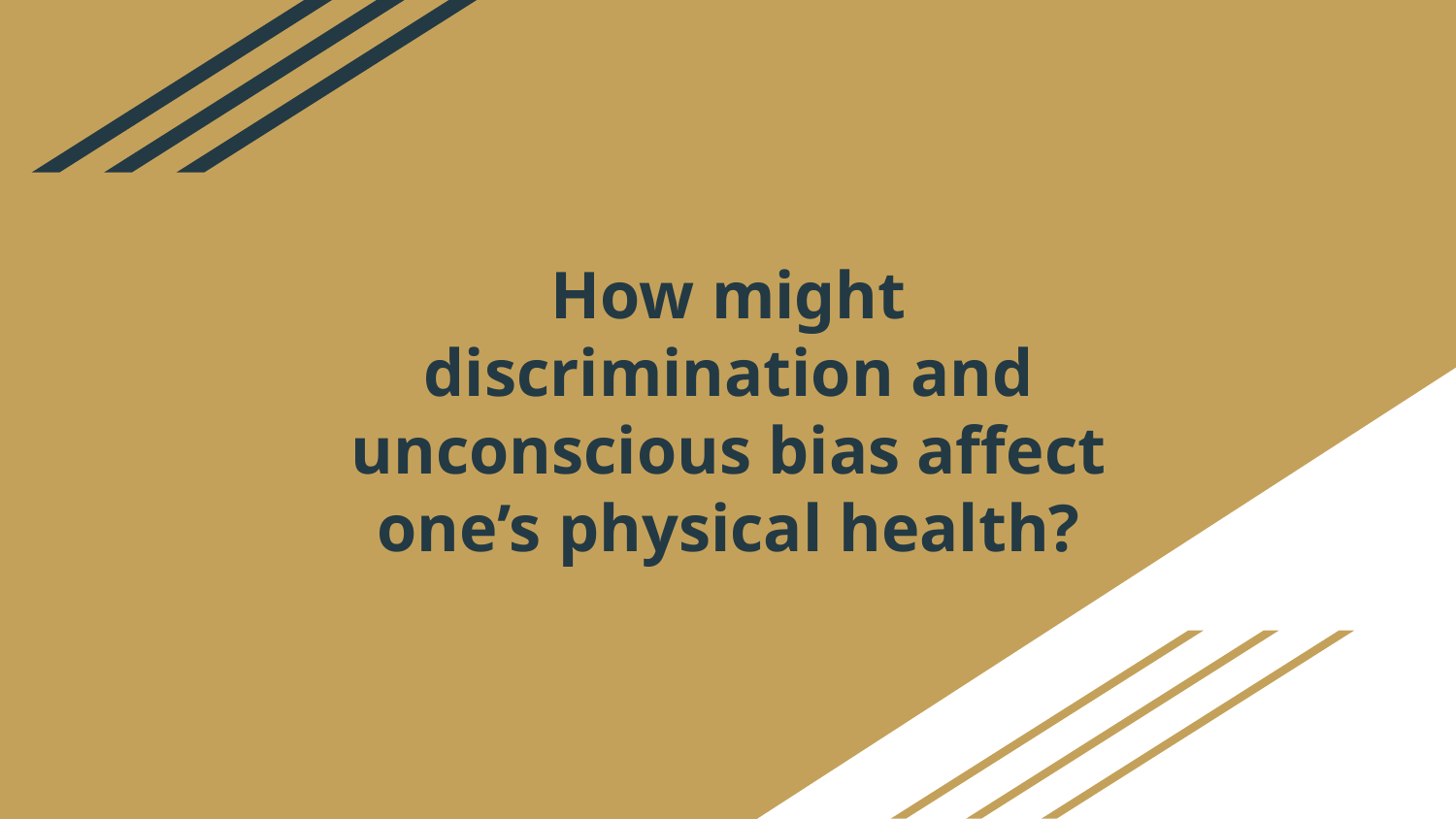

# How might discrimination and unconscious bias affect one’s physical health?

## Slide 41
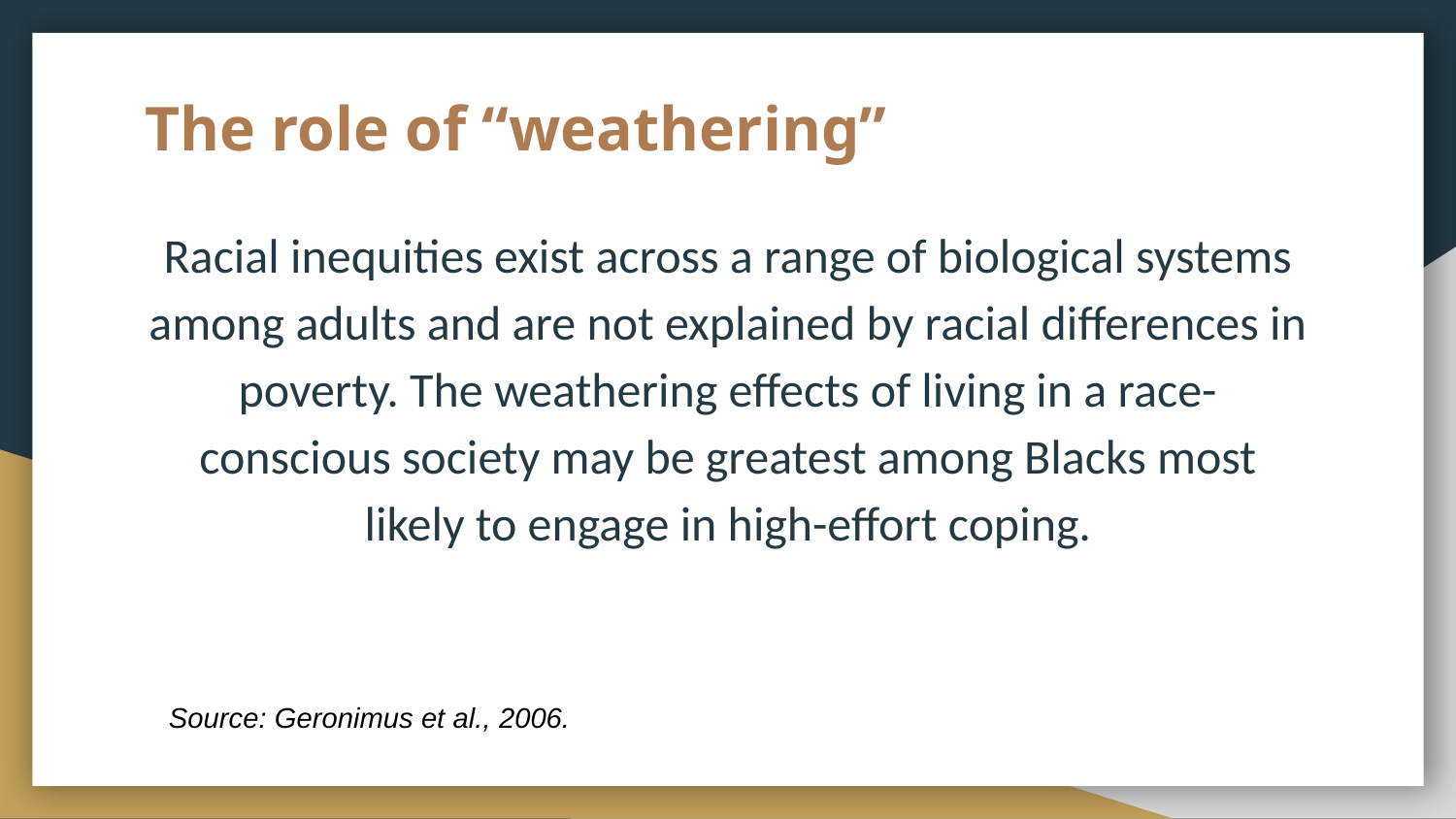

# The role of “weathering”
Racial inequities exist across a range of biological systems among adults and are not explained by racial differences in poverty. The weathering effects of living in a race-conscious society may be greatest among Blacks most likely to engage in high-effort coping.
Source: Geronimus et al., 2006.

## Slide 42
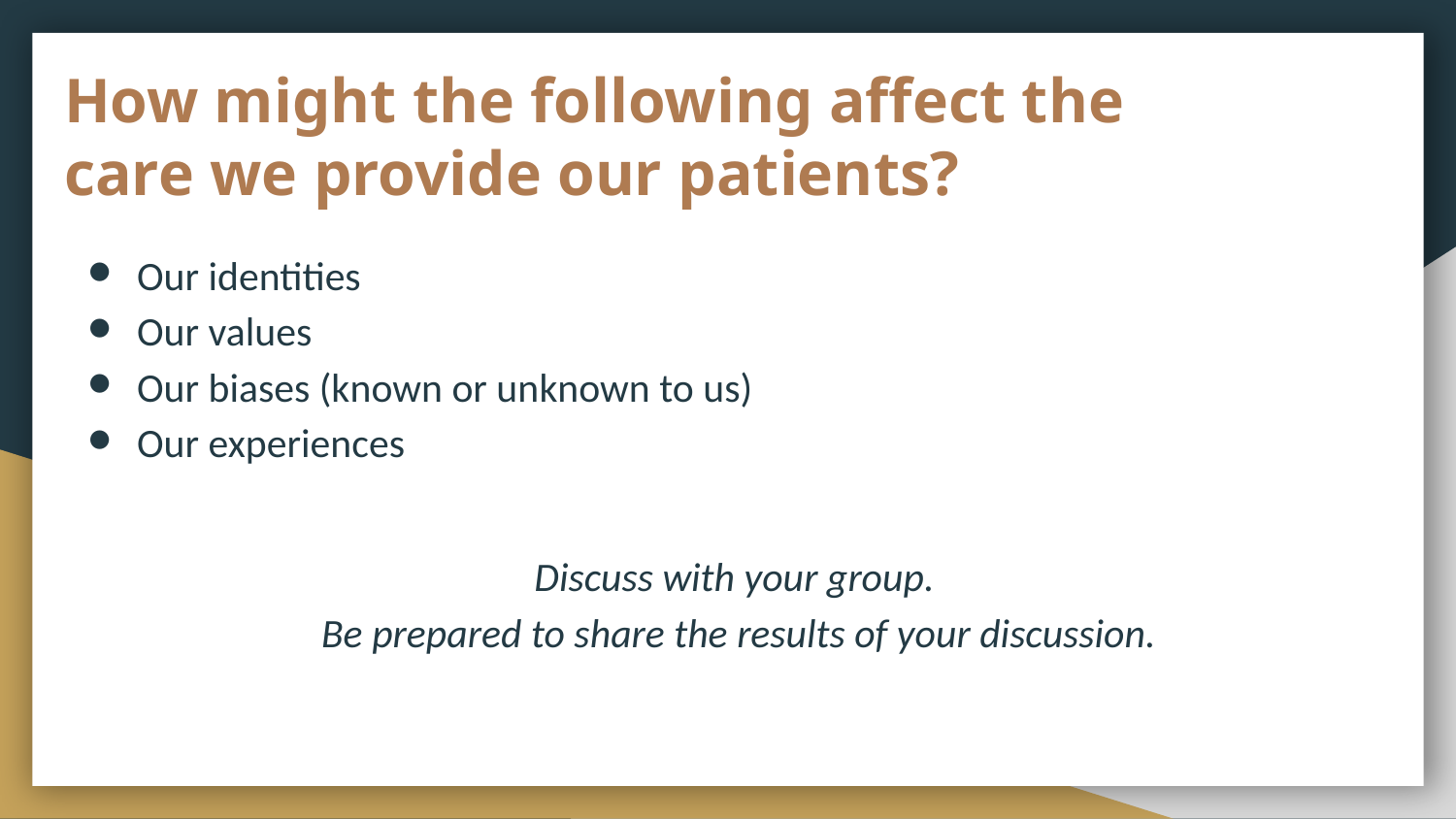

# How might the following affect the care we provide our patients?
Our identities
Our values
Our biases (known or unknown to us)
Our experiences
Discuss with your group.
Be prepared to share the results of your discussion.

## Slide 43
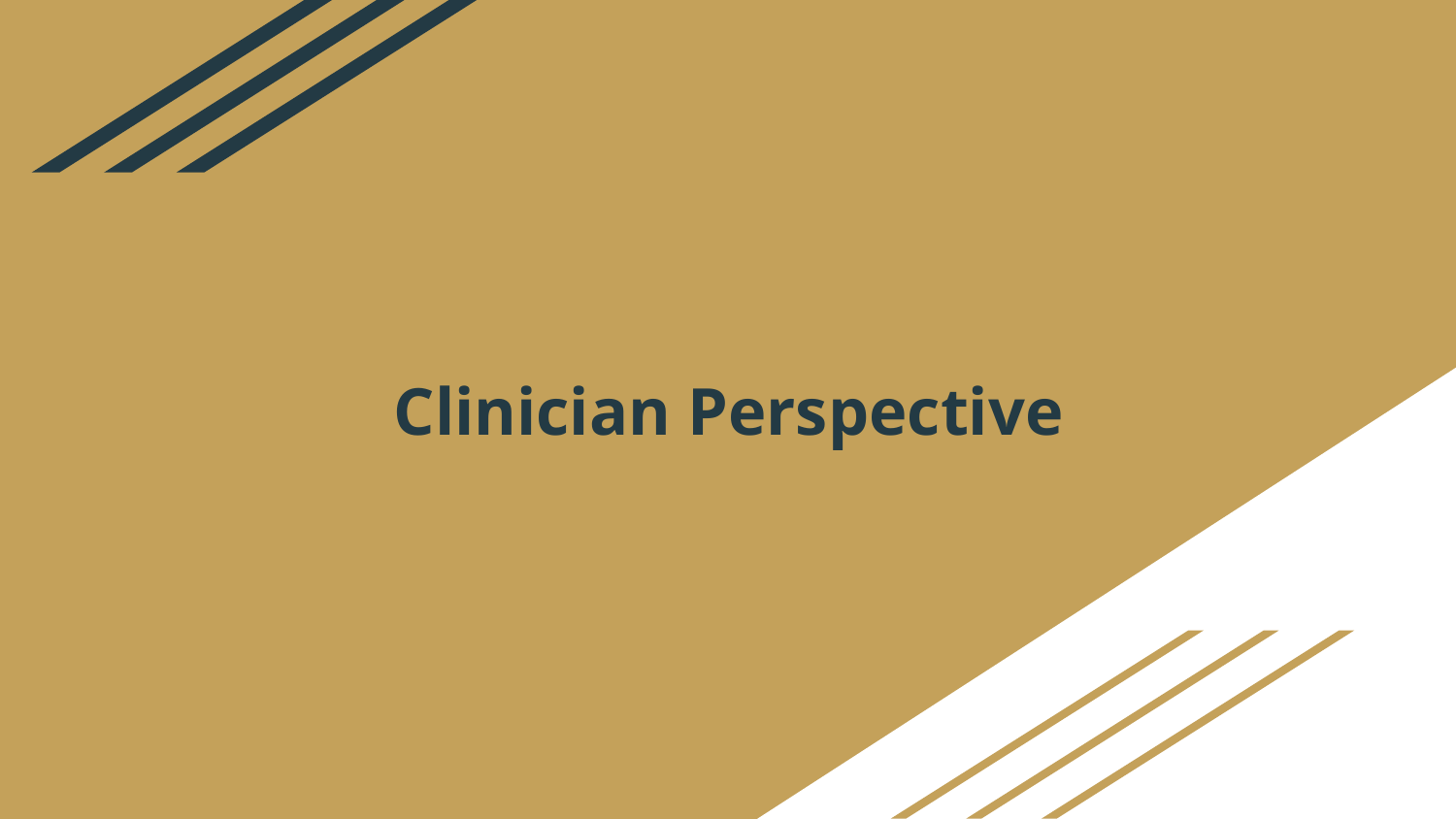

# Clinician Perspective

## Slide 44
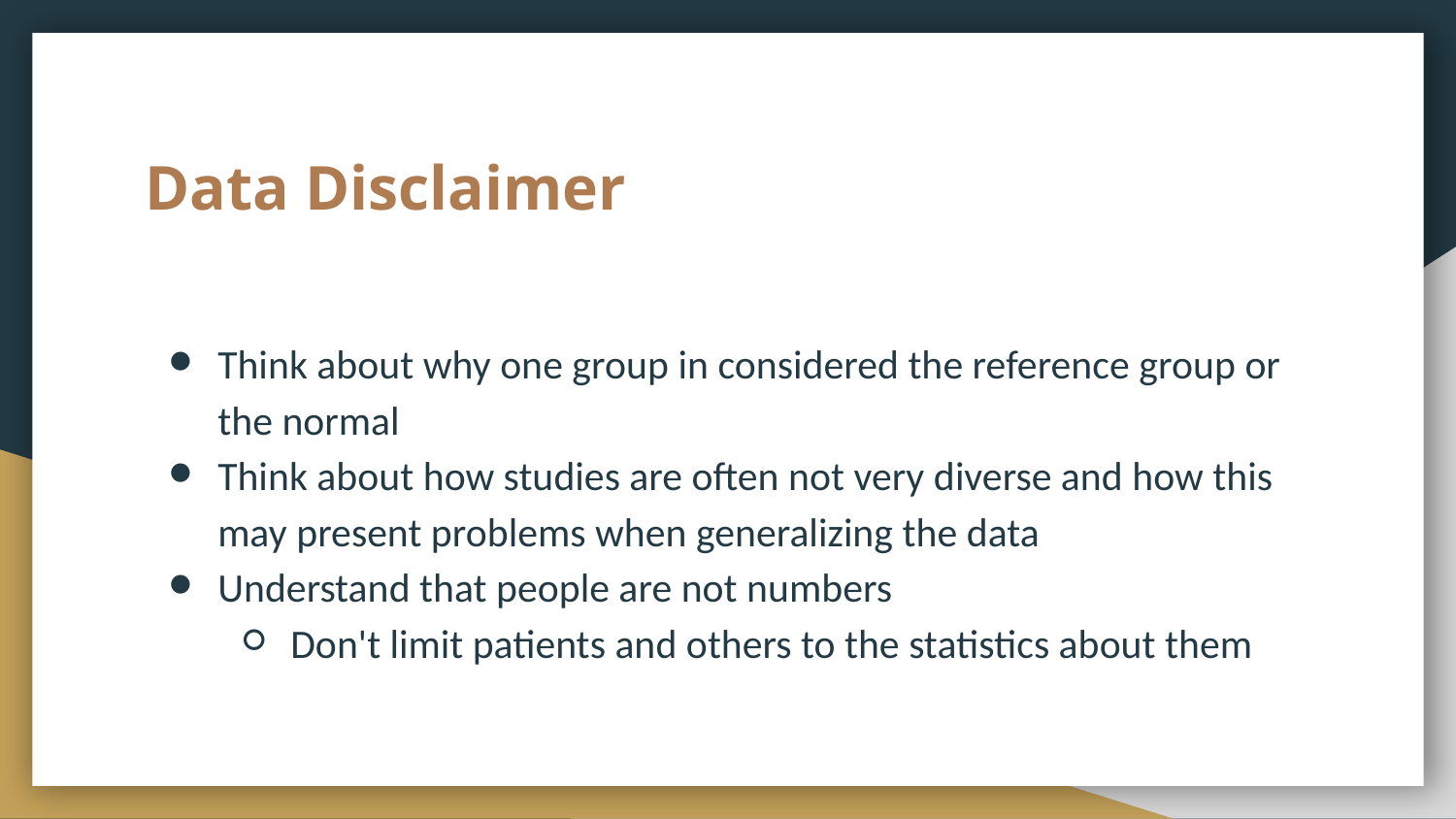

# Data Disclaimer
Think about why one group in considered the reference group or the normal
Think about how studies are often not very diverse and how this may present problems when generalizing the data
Understand that people are not numbers
Don't limit patients and others to the statistics about them

## Slide 45
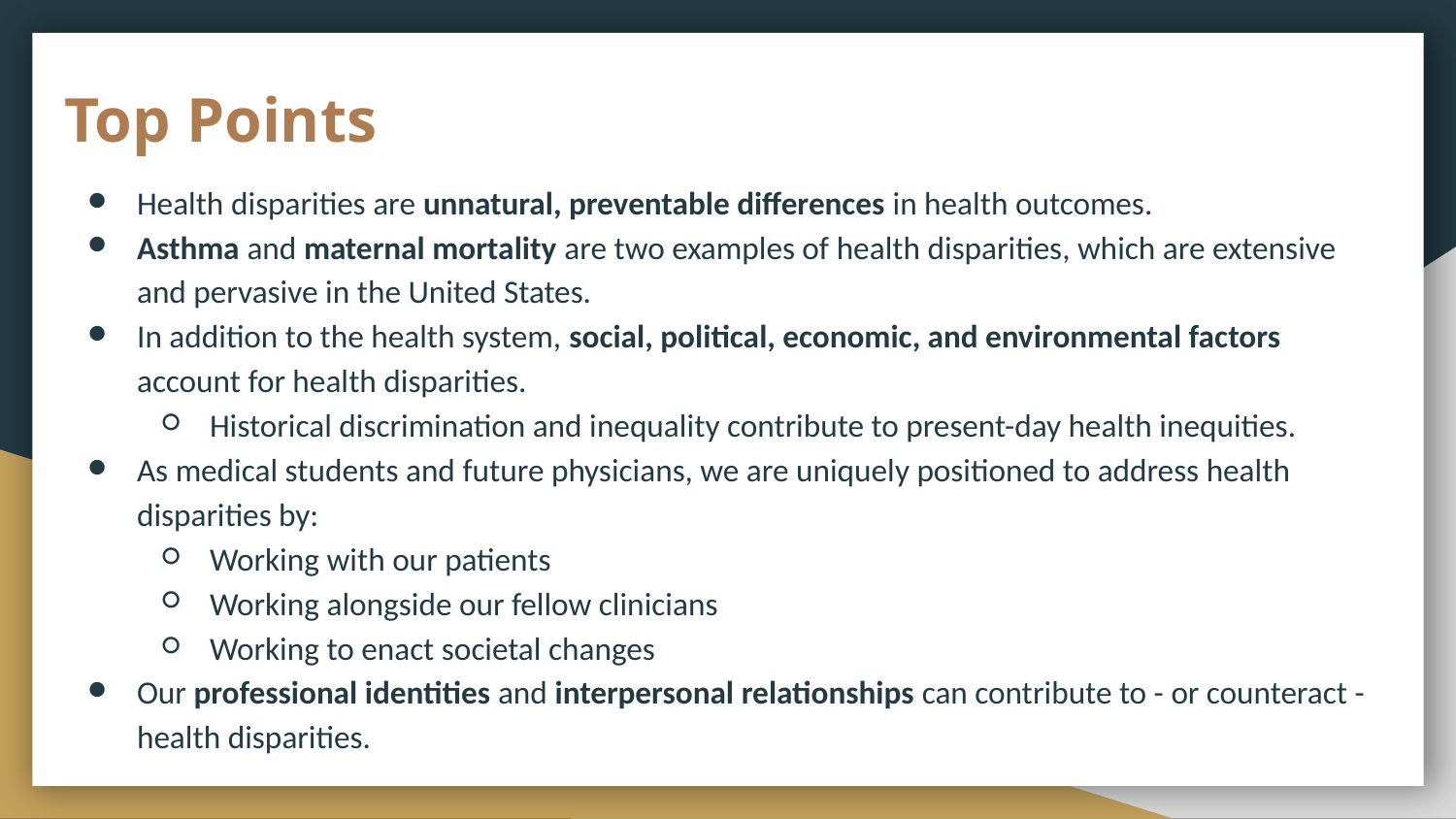

# Top Points
Health disparities are unnatural, preventable differences in health outcomes.
Asthma and maternal mortality are two examples of health disparities, which are extensive and pervasive in the United States.
In addition to the health system, social, political, economic, and environmental factors account for health disparities.
Historical discrimination and inequality contribute to present-day health inequities.
As medical students and future physicians, we are uniquely positioned to address health disparities by:
Working with our patients
Working alongside our fellow clinicians
Working to enact societal changes
Our professional identities and interpersonal relationships can contribute to - or counteract - health disparities.

## Slide 46
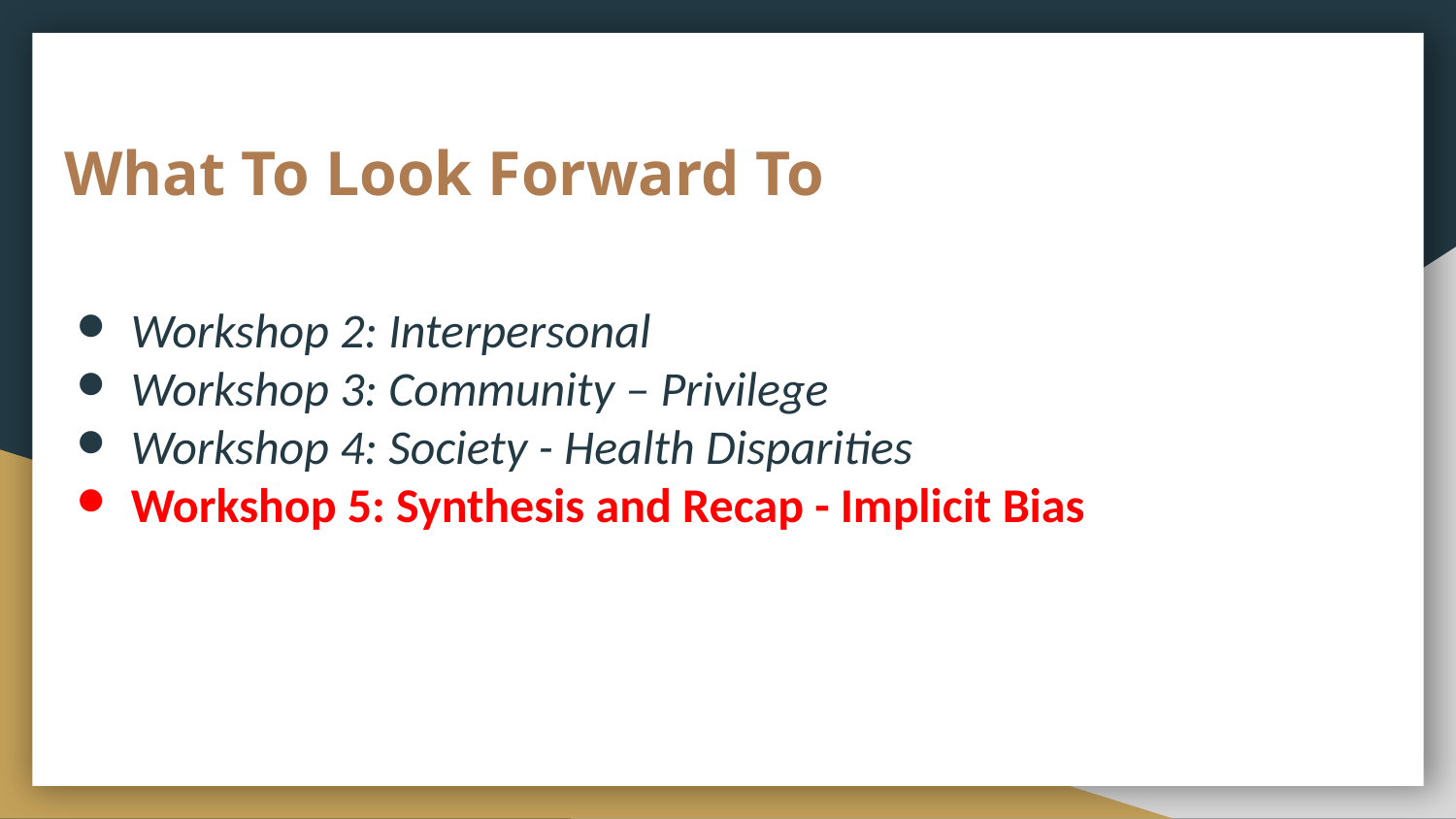

# What To Look Forward To
Workshop 2: Interpersonal
Workshop 3: Community – Privilege
Workshop 4: Society - Health Disparities
Workshop 5: Synthesis and Recap - Implicit Bias

## Slide 47
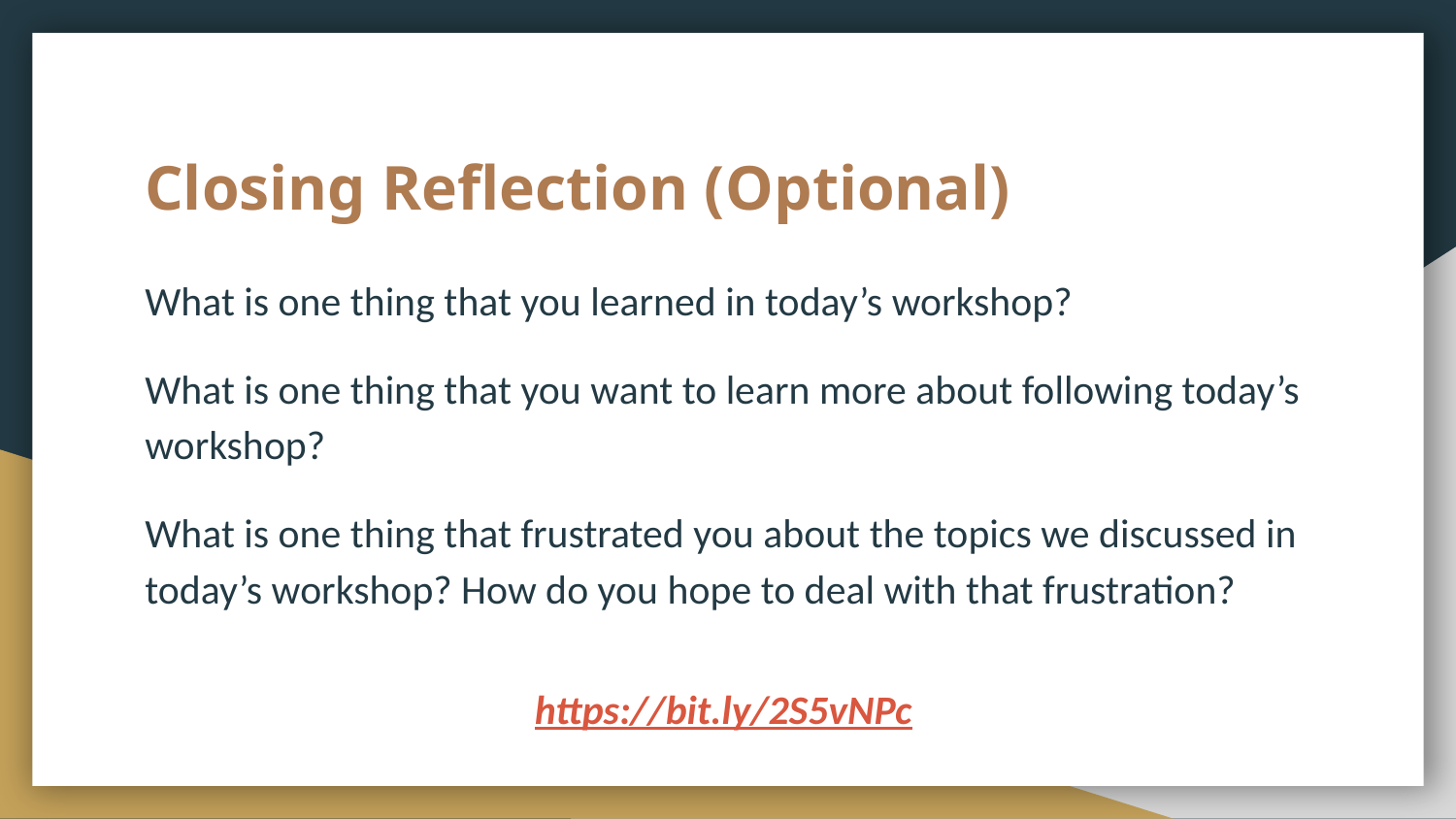

# Closing Reflection (Optional)
What is one thing that you learned in today’s workshop?
What is one thing that you want to learn more about following today’s workshop?
What is one thing that frustrated you about the topics we discussed in today’s workshop? How do you hope to deal with that frustration?
https://bit.ly/2S5vNPc
