# Supplementary material for: Critical Consciousness as a Framework for Health Equity–Focused Peer Learning
Source: MedEdPORTAL. 2021 Apr 28;17:11145. doi: 10.15766/mep_2374-8265.11145 (PMC8079426; doi:10.15766/mep_2374-8265.11145)
Supplement: Supplementary file 1 — Workshop 1 Presentation.pptxWorkshop 1 Student Handout.docxWorkshop 2 Presentation.pptxWorkshop 2 Student Handout.docxWorkshop 3 Presentation.pptxWorkshop 3 Student Handout.docxWorkshop 4 Presentation.pptxWorkshop 5 Presentation.pptxFacilitator Orientation.pptxWorkshop 1 Facilitator Guide.docxWorkshop 2 Facilitator Guide.docxWorkshop 3 Facilitator Guide.docxWorkshop 4 Facilitator Guide.docxWorkshop 5 Facilitator Guide.docxEvaluation Tools.docx [file mep_2374-8265.11145-s001.zip › H. Workshop 5 Presentation.pptx]

## Slide 1
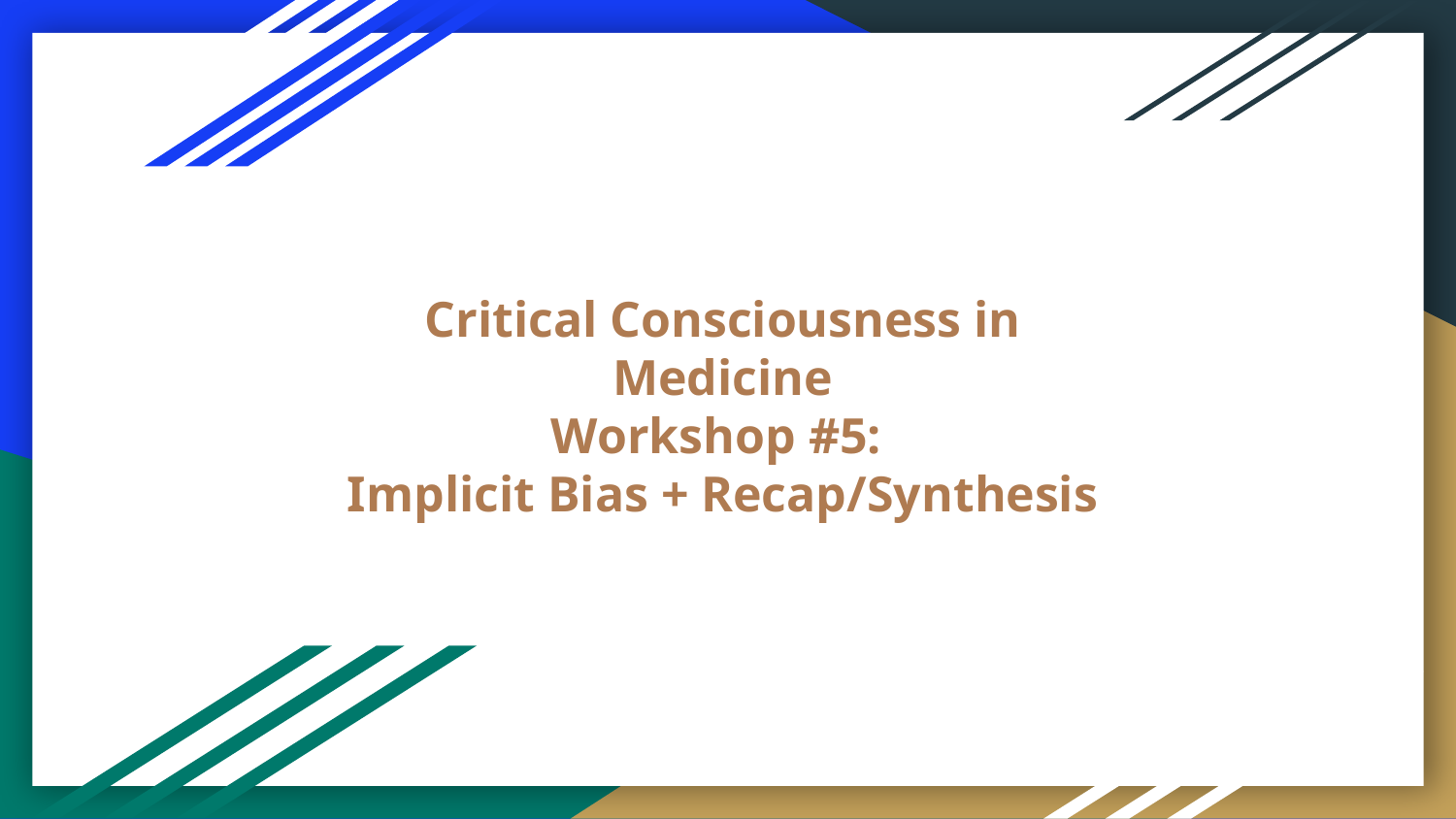

# Critical Consciousness in Medicine
Workshop #5:
Implicit Bias + Recap/Synthesis

## Slide 2
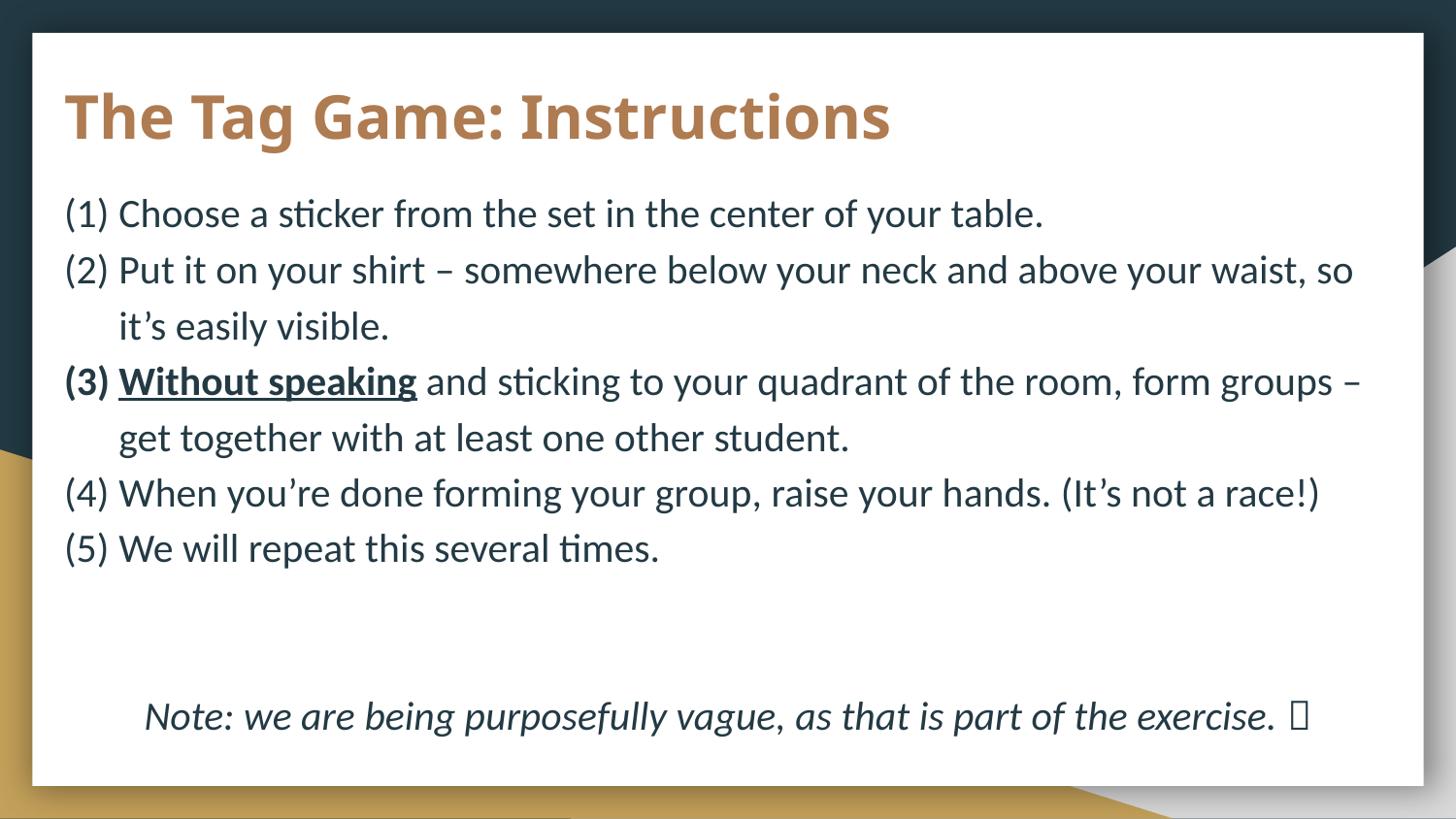

# The Tag Game: Instructions
Choose a sticker from the set in the center of your table.
Put it on your shirt – somewhere below your neck and above your waist, so it’s easily visible.
Without speaking and sticking to your quadrant of the room, form groups – get together with at least one other student.
When you’re done forming your group, raise your hands. (It’s not a race!)
We will repeat this several times.
Note: we are being purposefully vague, as that is part of the exercise. 

## Slide 3
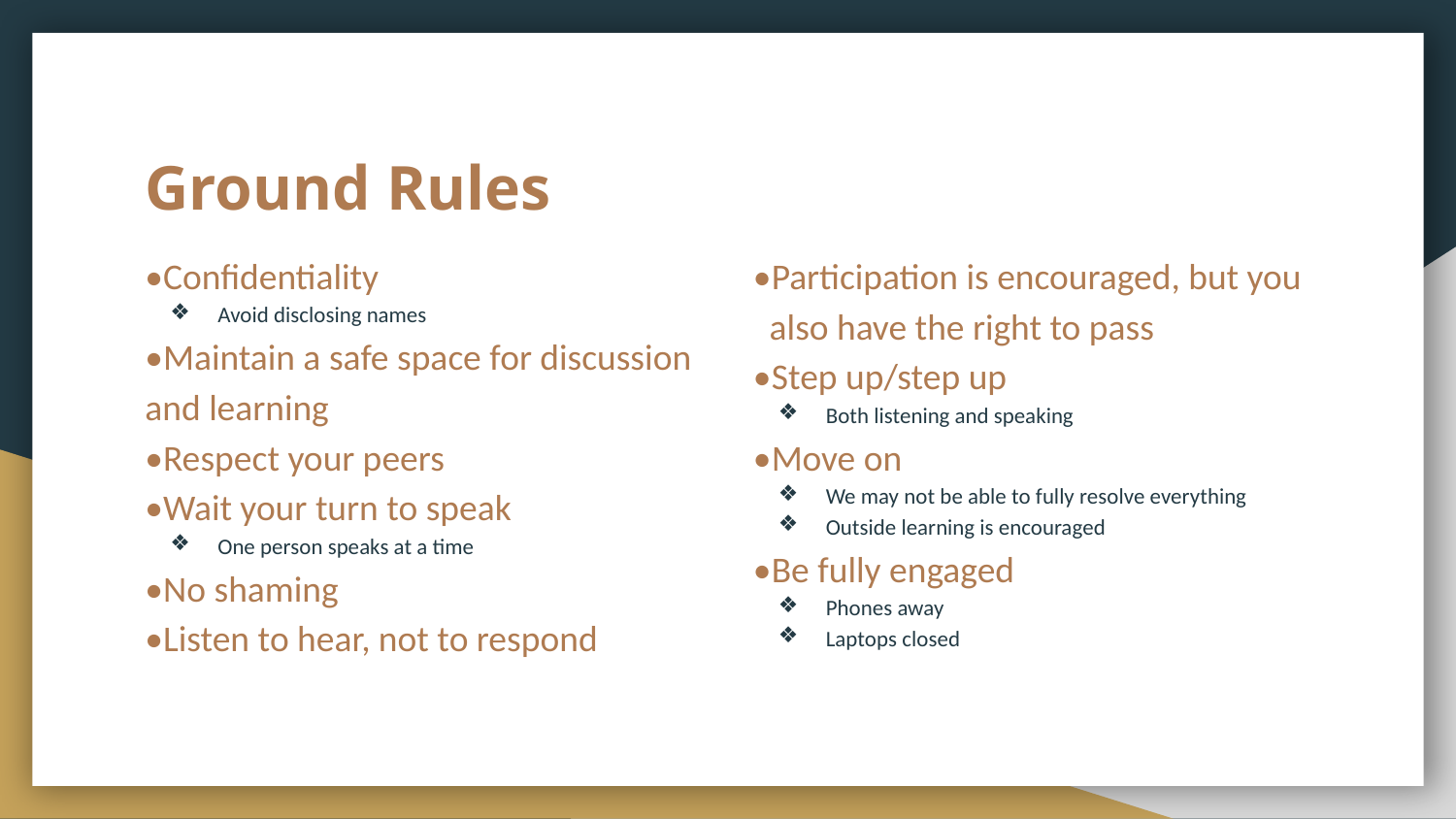

# Ground Rules
•Confidentiality
Avoid disclosing names
•Maintain a safe space for discussion and learning
•Respect your peers
•Wait your turn to speak
One person speaks at a time
•No shaming
•Listen to hear, not to respond
•Participation is encouraged, but you also have the right to pass
•Step up/step up
Both listening and speaking
•Move on
We may not be able to fully resolve everything
Outside learning is encouraged
•Be fully engaged
Phones away
Laptops closed

## Slide 4
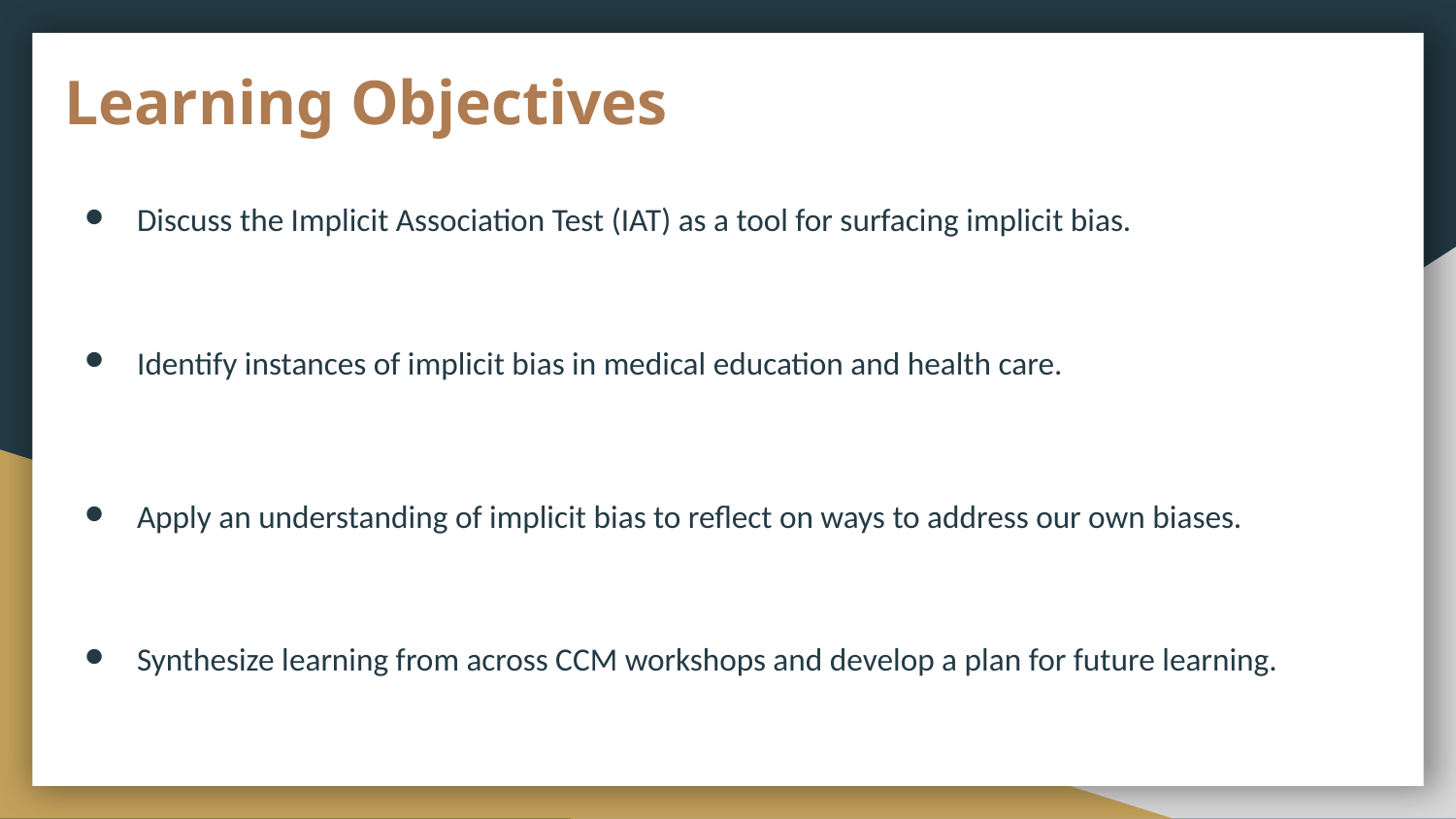

# Learning Objectives
Discuss the Implicit Association Test (IAT) as a tool for surfacing implicit bias.
Identify instances of implicit bias in medical education and health care.
Apply an understanding of implicit bias to reflect on ways to address our own biases.
Synthesize learning from across CCM workshops and develop a plan for future learning.

## Slide 5
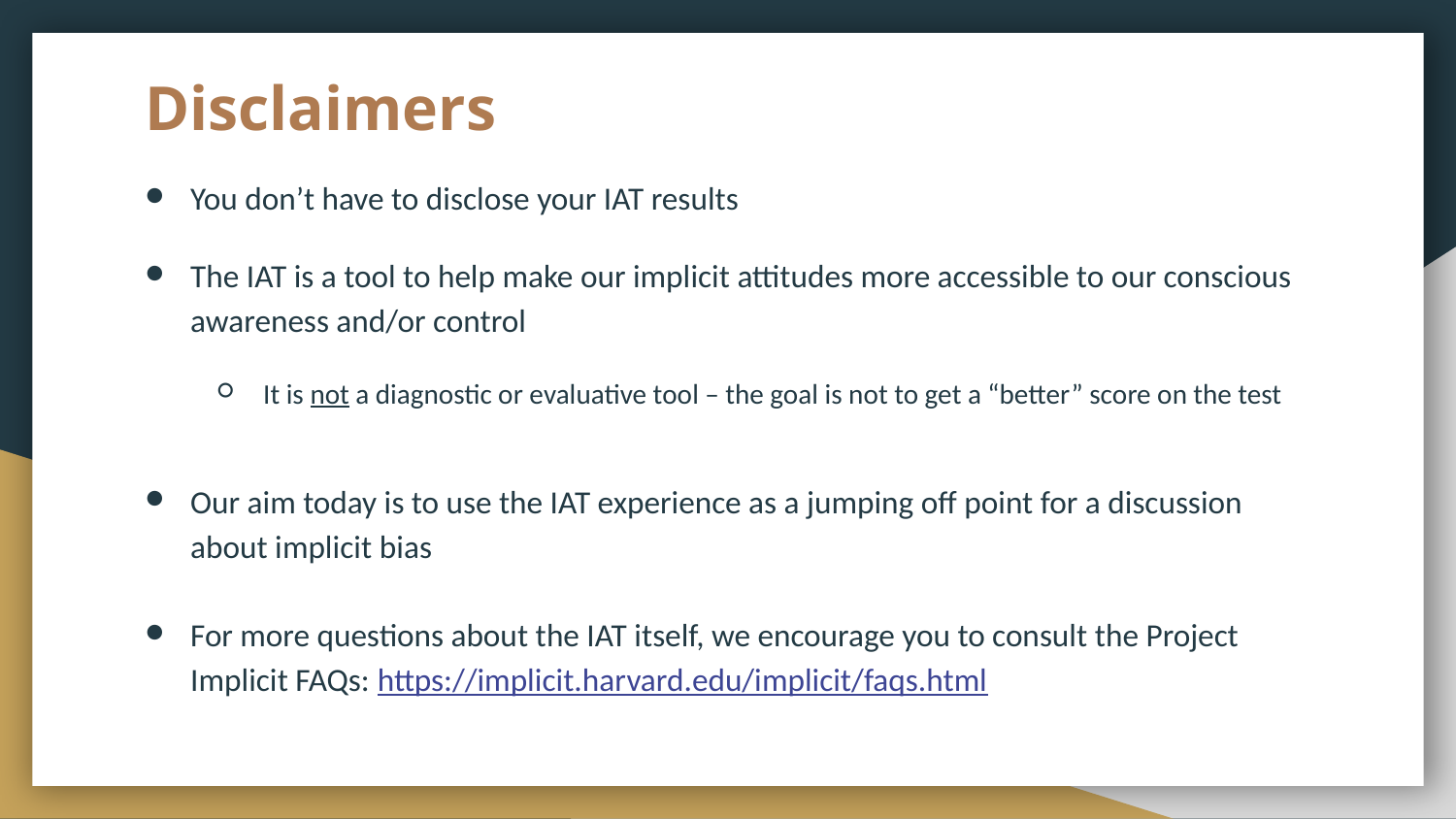

# Disclaimers
You don’t have to disclose your IAT results
The IAT is a tool to help make our implicit attitudes more accessible to our conscious awareness and/or control
It is not a diagnostic or evaluative tool – the goal is not to get a “better” score on the test
Our aim today is to use the IAT experience as a jumping off point for a discussion about implicit bias
For more questions about the IAT itself, we encourage you to consult the Project Implicit FAQs: https://implicit.harvard.edu/implicit/faqs.html

## Slide 6
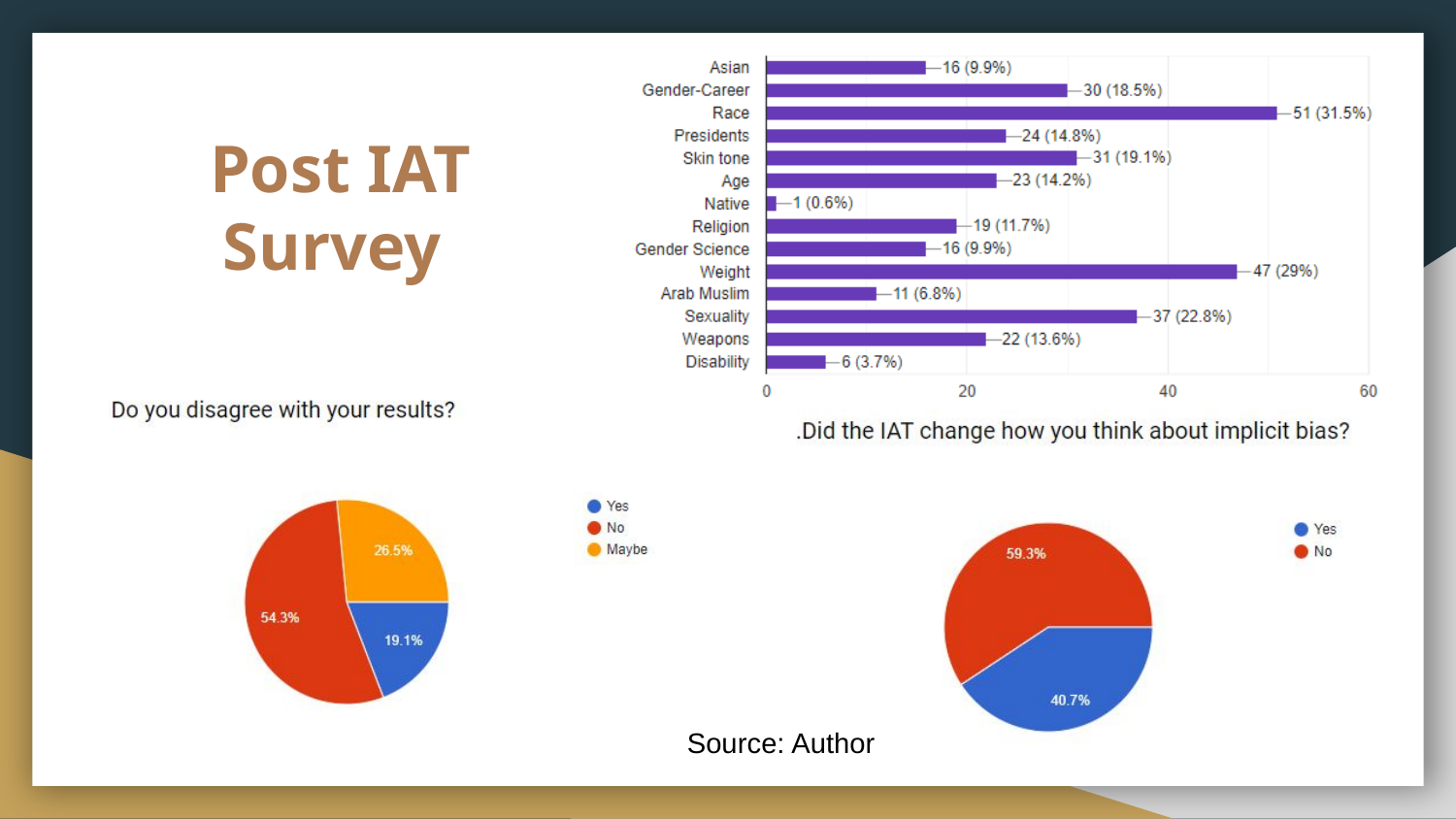

Post IAT Survey
Source: Author

## Slide 7
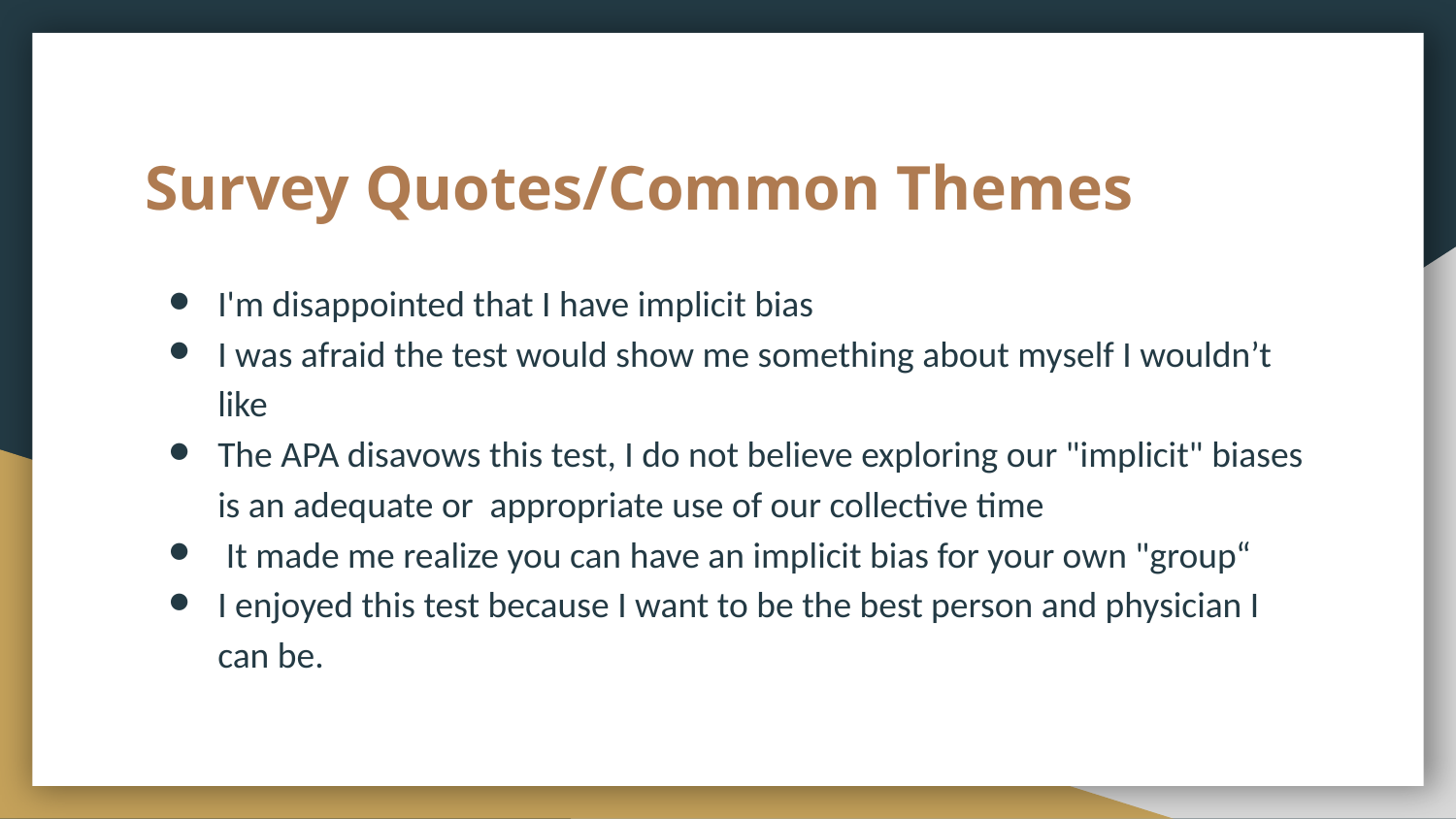

# Survey Quotes/Common Themes
I'm disappointed that I have implicit bias
I was afraid the test would show me something about myself I wouldn’t like
The APA disavows this test, I do not believe exploring our "implicit" biases is an adequate or appropriate use of our collective time
 It made me realize you can have an implicit bias for your own "group“
I enjoyed this test because I want to be the best person and physician I can be.

## Slide 8
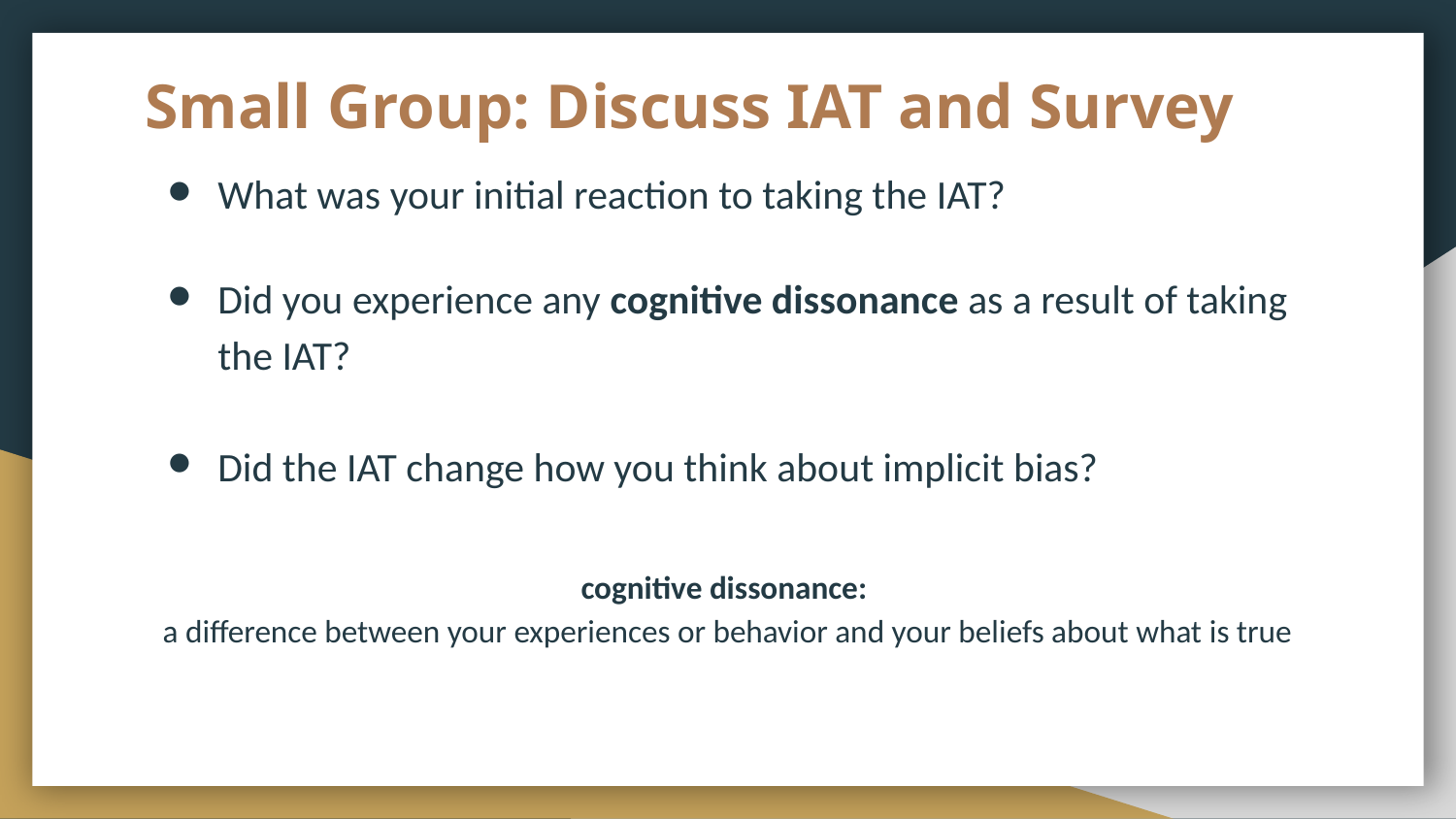

# Small Group: Discuss IAT and Survey
What was your initial reaction to taking the IAT?
Did you experience any cognitive dissonance as a result of taking the IAT?
Did the IAT change how you think about implicit bias?
cognitive dissonance:
a difference between your experiences or behavior and your beliefs about what is true

## Slide 9
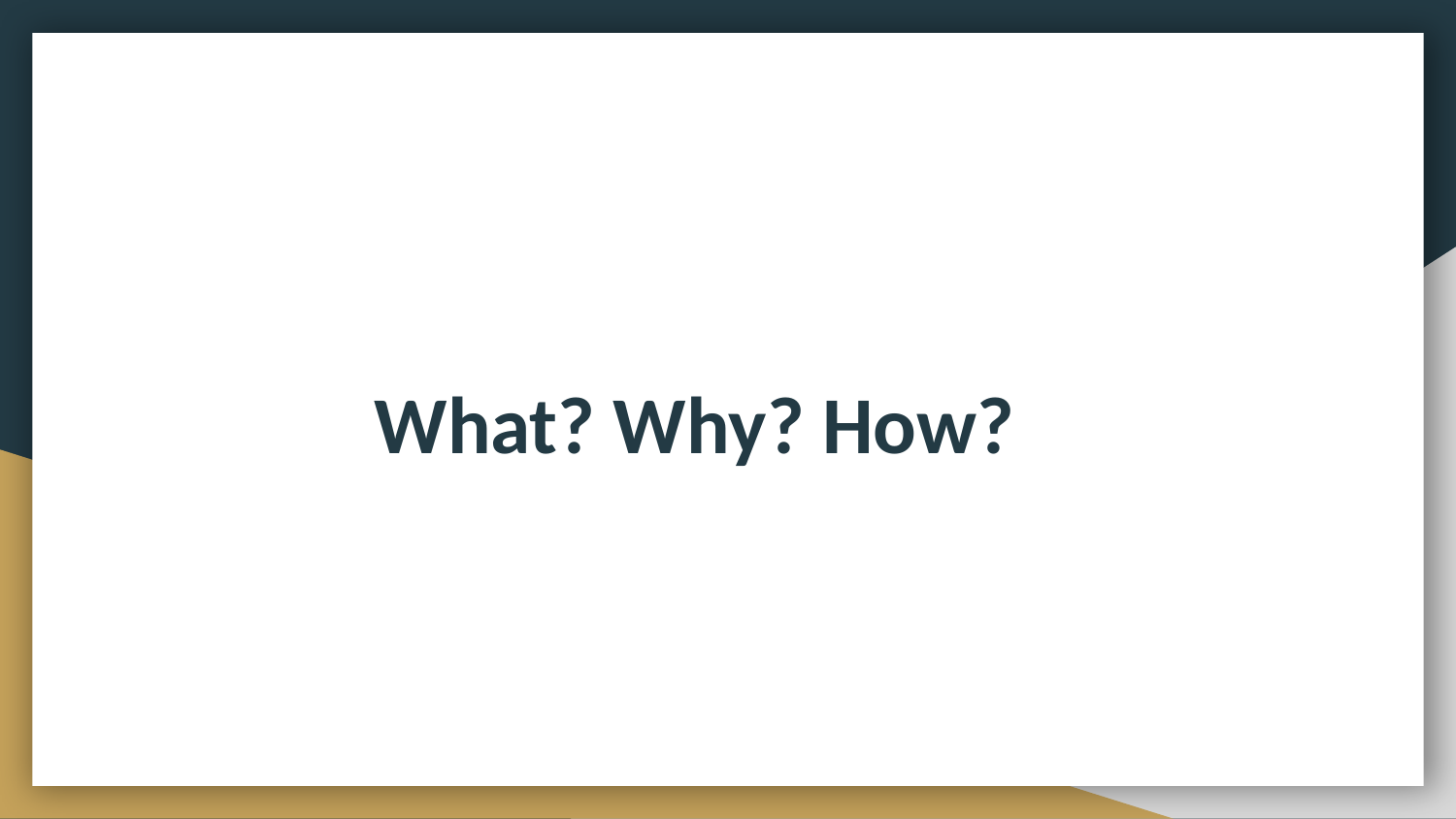

What? Why? How?

## Slide 10
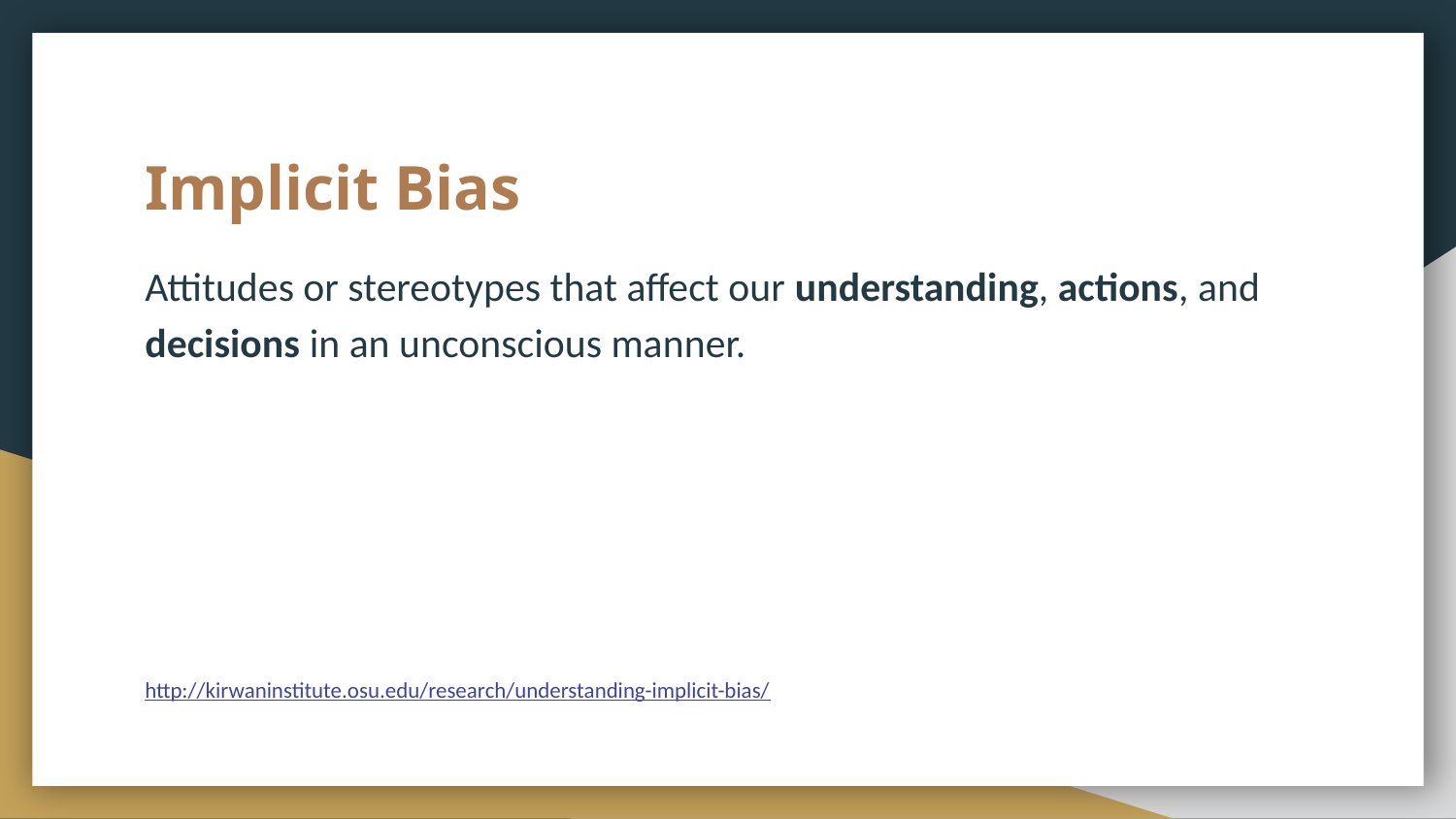

# Implicit Bias
Attitudes or stereotypes that affect our understanding, actions, and decisions in an unconscious manner.
http://kirwaninstitute.osu.edu/research/understanding-implicit-bias/

## Slide 11
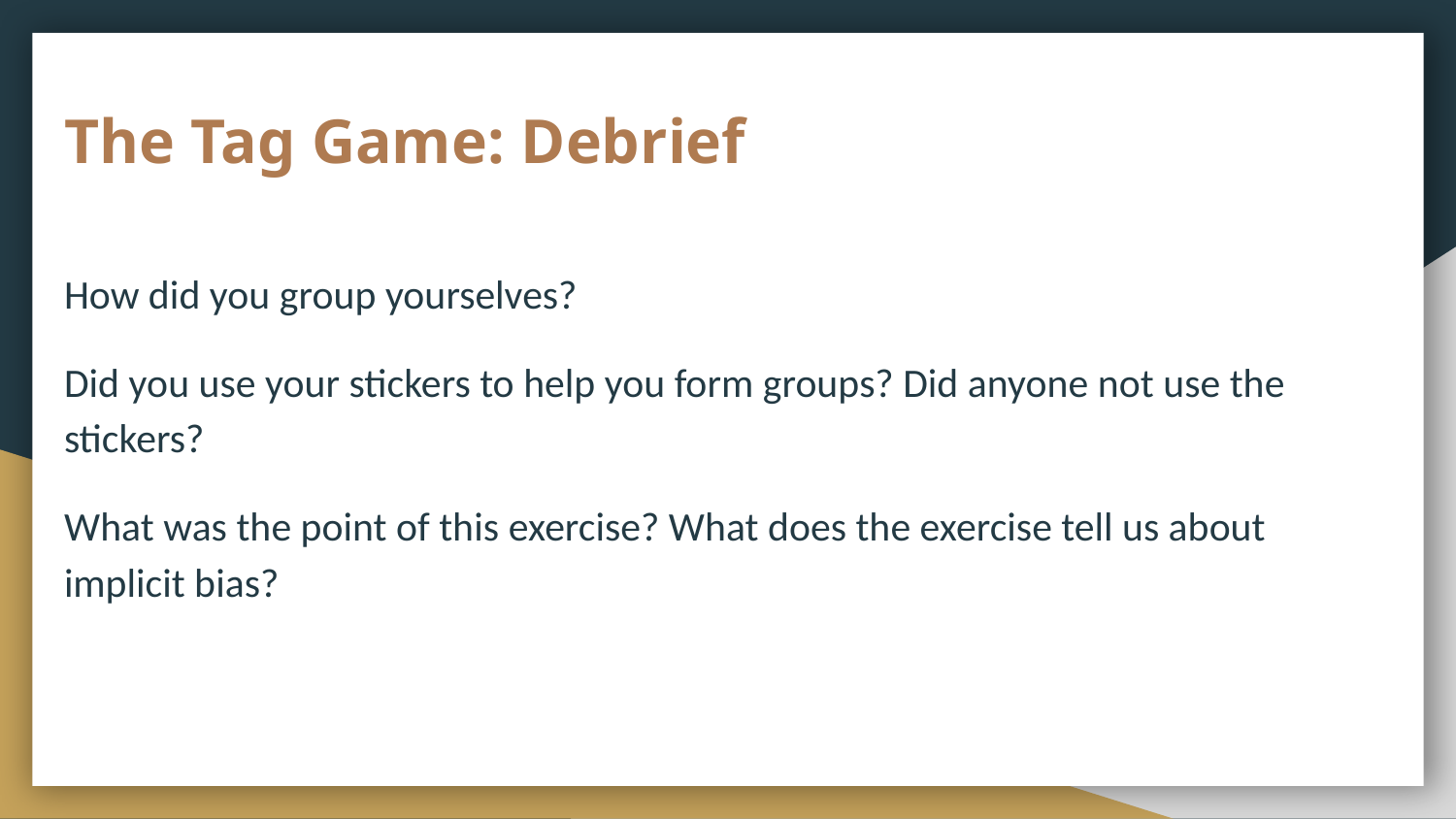

# The Tag Game: Debrief
How did you group yourselves?
Did you use your stickers to help you form groups? Did anyone not use the stickers?
What was the point of this exercise? What does the exercise tell us about implicit bias?

## Slide 12
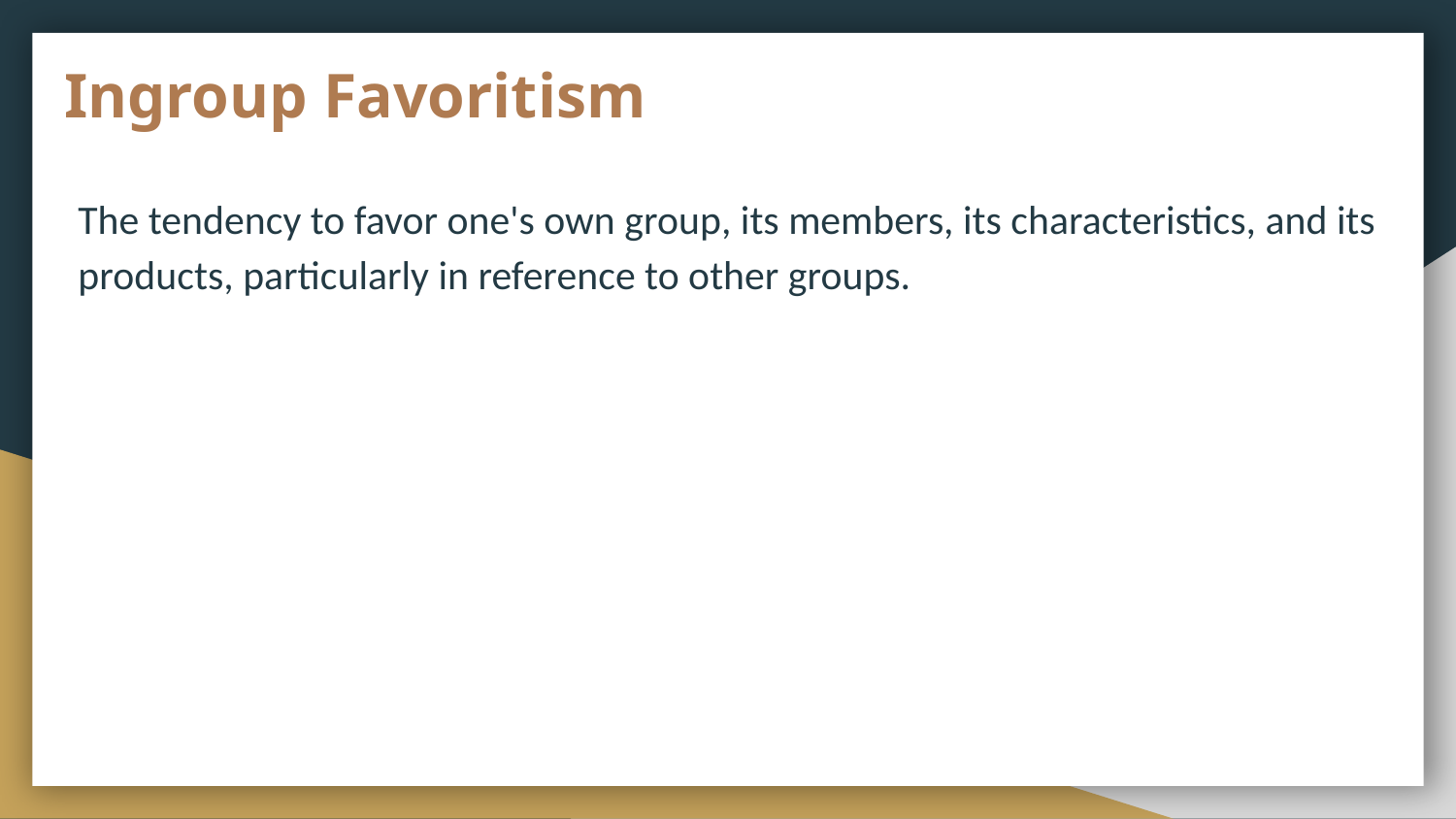

# Ingroup Favoritism
The tendency to favor one's own group, its members, its characteristics, and its products, particularly in reference to other groups.

## Slide 13
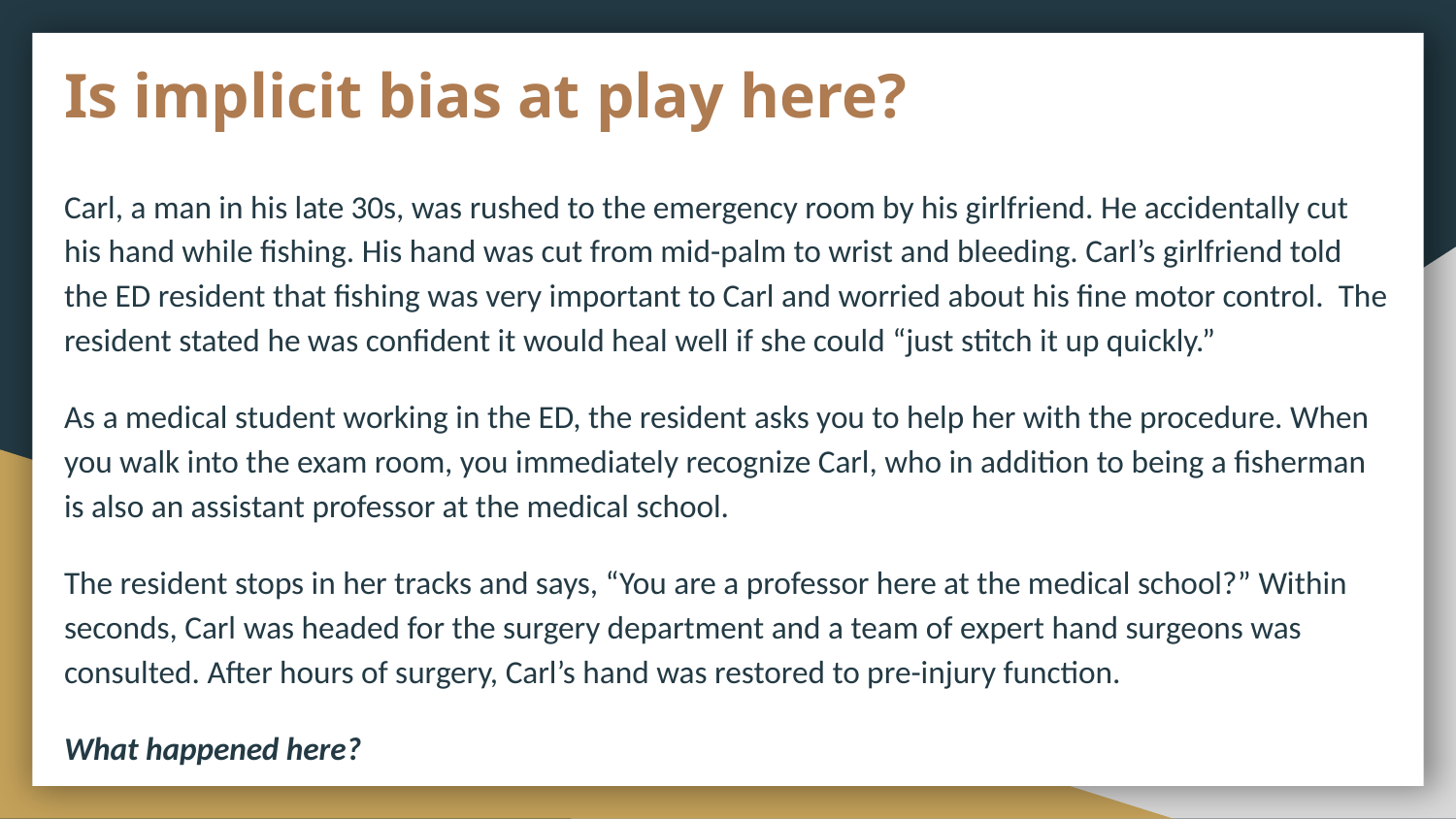

# Is implicit bias at play here?
Carl, a man in his late 30s, was rushed to the emergency room by his girlfriend. He accidentally cut his hand while fishing. His hand was cut from mid-palm to wrist and bleeding. Carl’s girlfriend told the ED resident that fishing was very important to Carl and worried about his fine motor control. The resident stated he was confident it would heal well if she could “just stitch it up quickly.”
As a medical student working in the ED, the resident asks you to help her with the procedure. When you walk into the exam room, you immediately recognize Carl, who in addition to being a fisherman is also an assistant professor at the medical school.
The resident stops in her tracks and says, “You are a professor here at the medical school?” Within seconds, Carl was headed for the surgery department and a team of expert hand surgeons was consulted. After hours of surgery, Carl’s hand was restored to pre-injury function.
What happened here?

## Slide 14
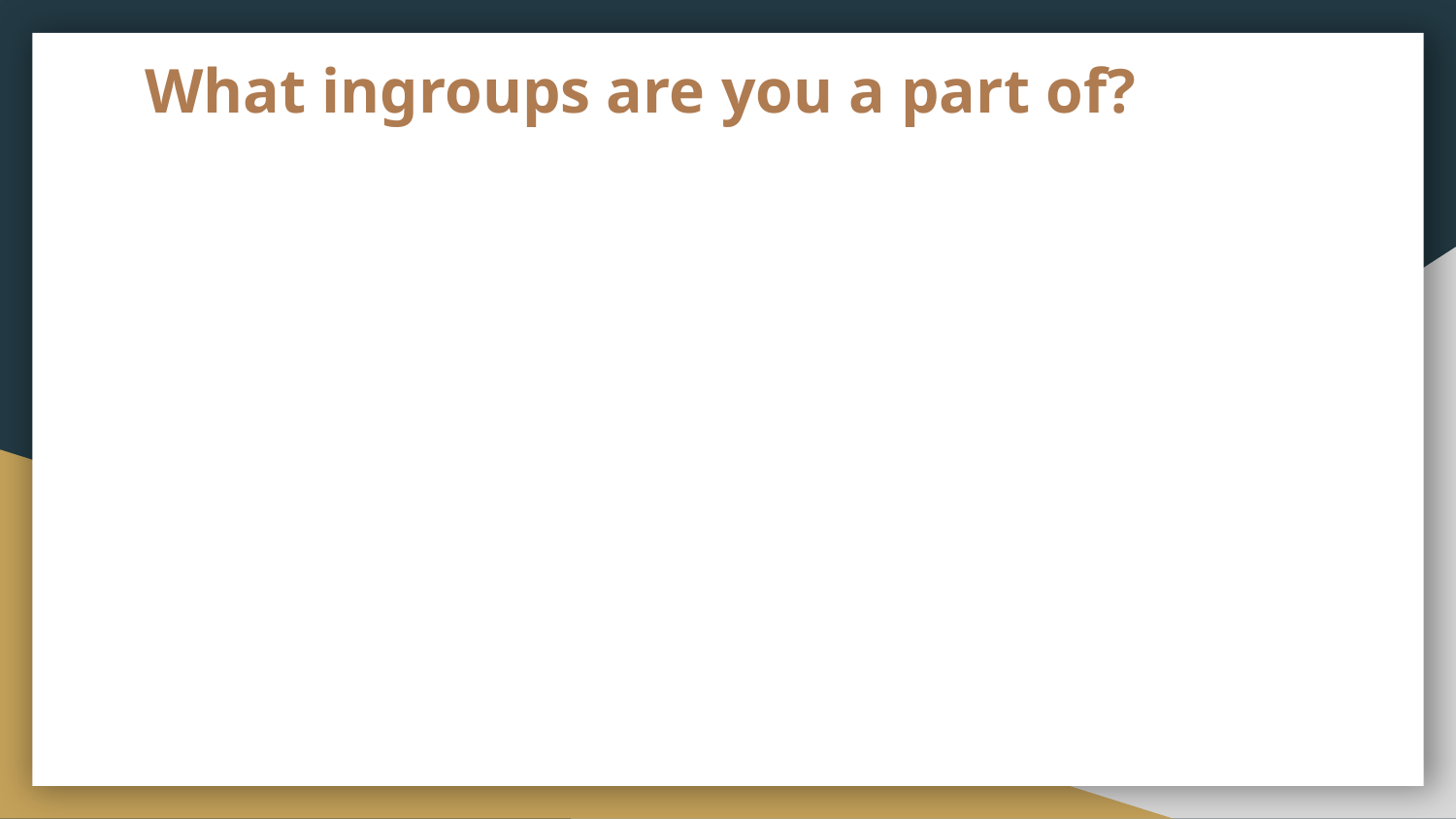

# What ingroups are you a part of?

## Slide 15
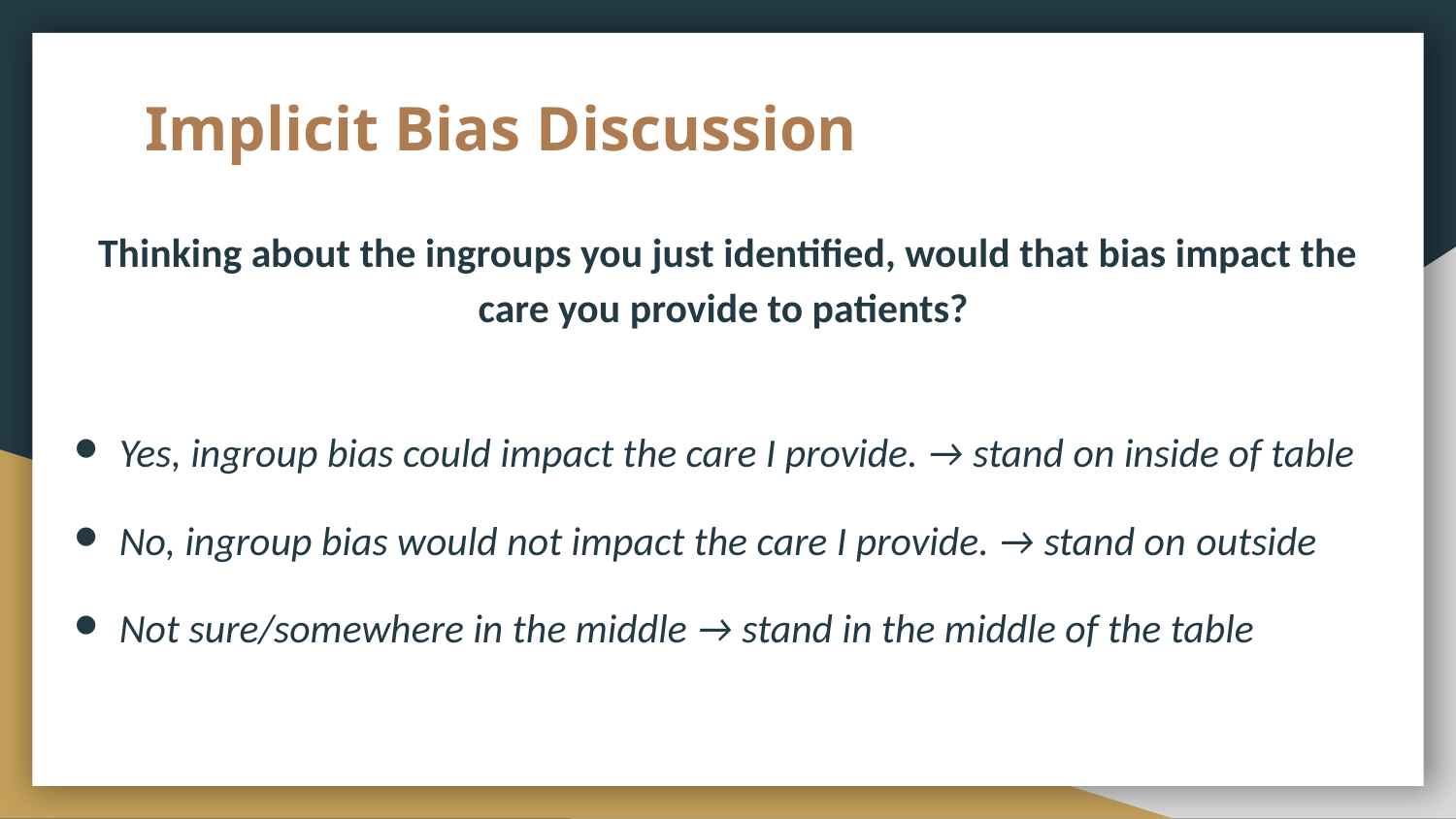

# Implicit Bias Discussion
Thinking about the ingroups you just identified, would that bias impact the care you provide to patients?
Yes, ingroup bias could impact the care I provide. → stand on inside of table
No, ingroup bias would not impact the care I provide. → stand on outside
Not sure/somewhere in the middle → stand in the middle of the table

## Slide 16
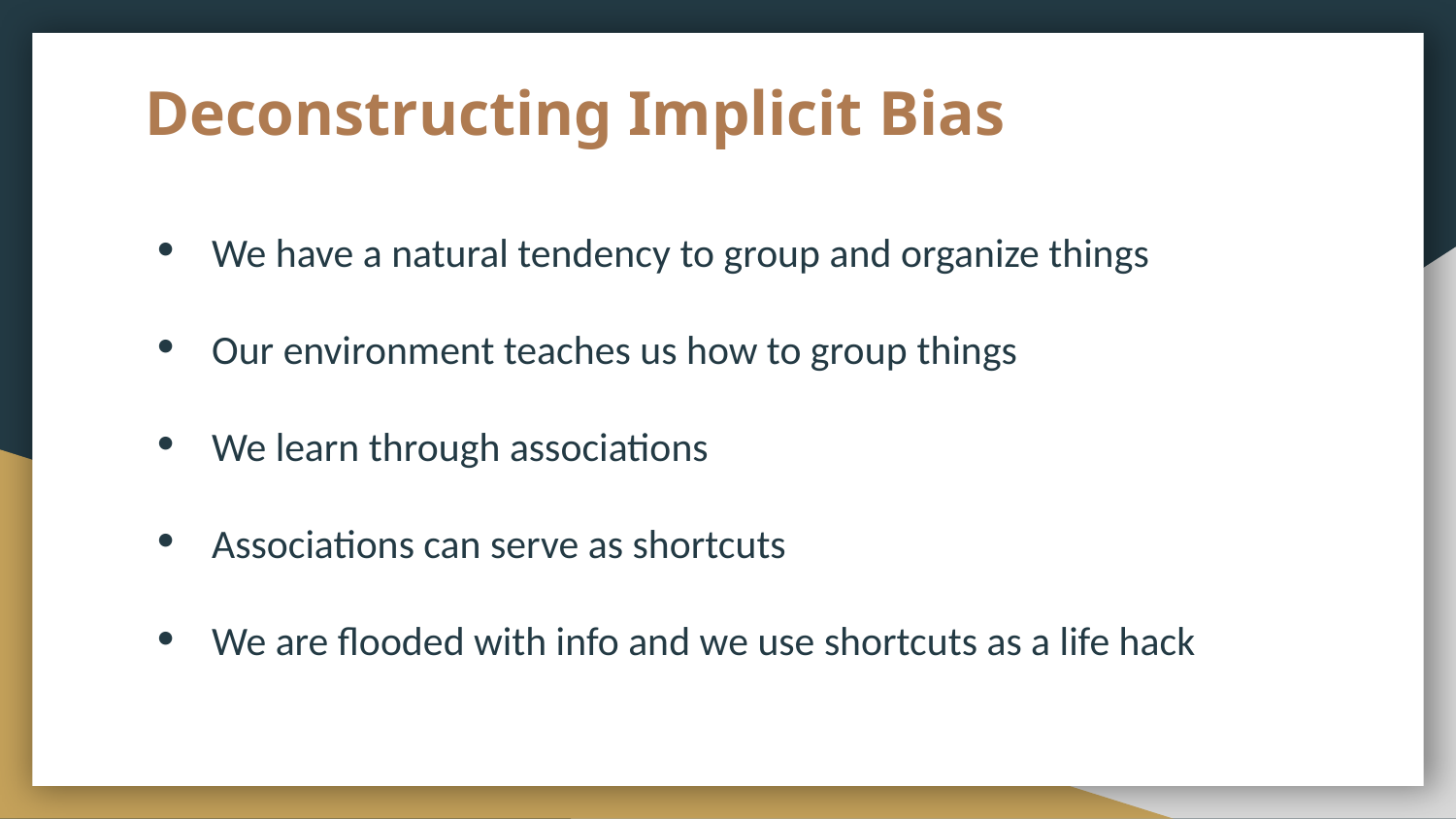

# Deconstructing Implicit Bias
We have a natural tendency to group and organize things
Our environment teaches us how to group things
We learn through associations
Associations can serve as shortcuts
We are flooded with info and we use shortcuts as a life hack

## Slide 17
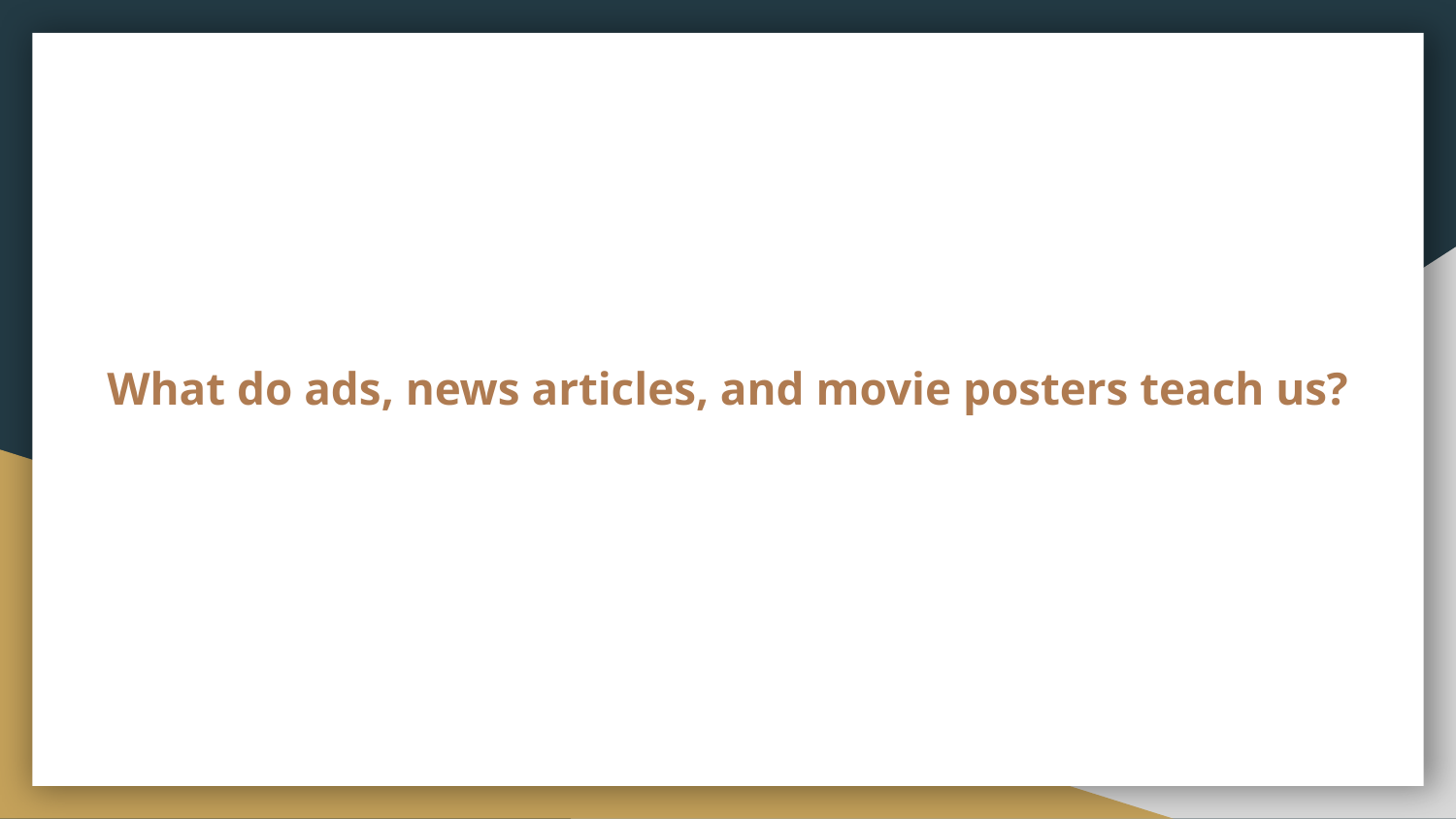

# What do ads, news articles, and movie posters teach us?

## Slide 18
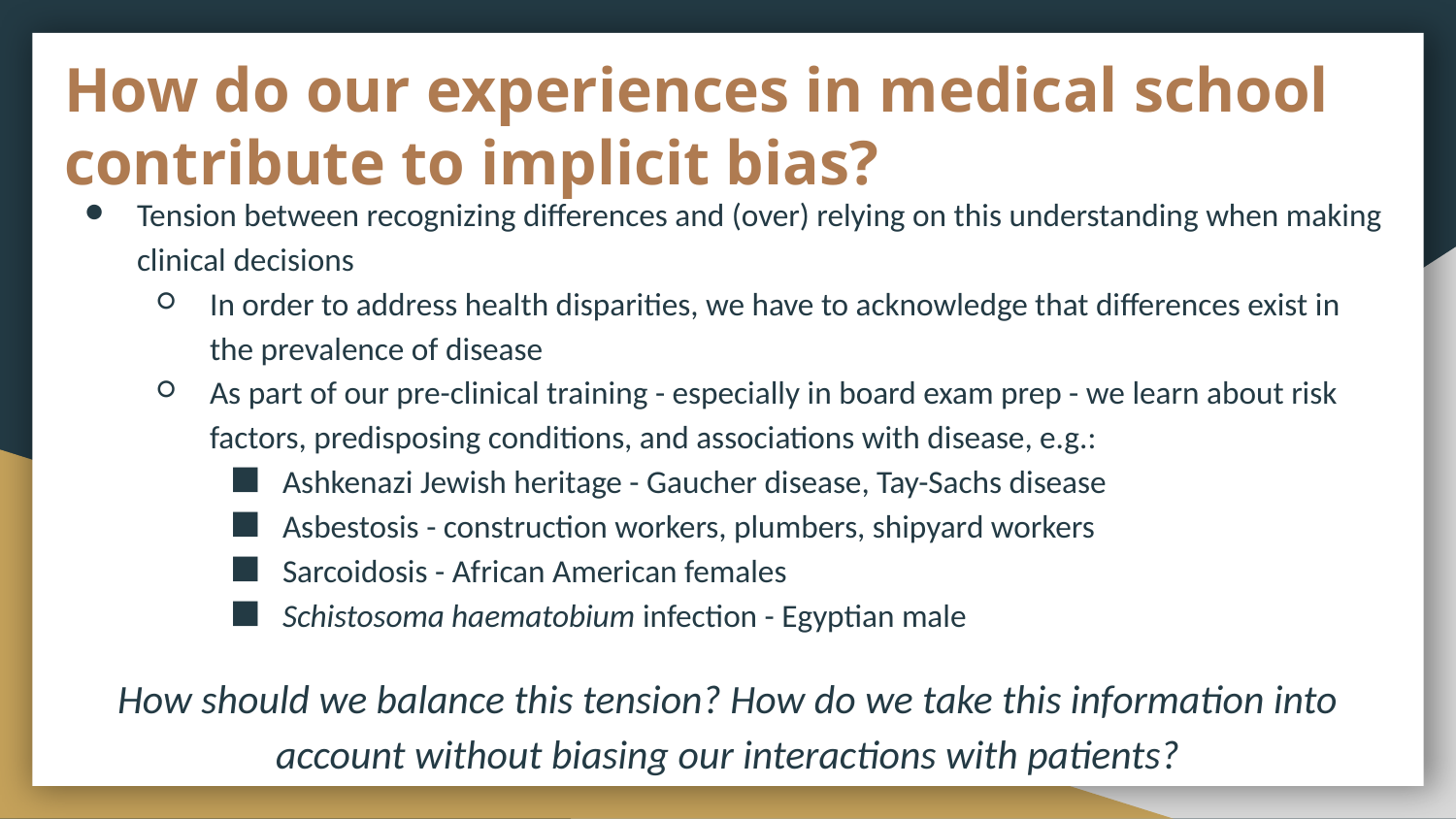

# How do our experiences in medical school contribute to implicit bias?
Tension between recognizing differences and (over) relying on this understanding when making clinical decisions
In order to address health disparities, we have to acknowledge that differences exist in the prevalence of disease
As part of our pre-clinical training - especially in board exam prep - we learn about risk factors, predisposing conditions, and associations with disease, e.g.:
Ashkenazi Jewish heritage - Gaucher disease, Tay-Sachs disease
Asbestosis - construction workers, plumbers, shipyard workers
Sarcoidosis - African American females
Schistosoma haematobium infection - Egyptian male
How should we balance this tension? How do we take this information into
account without biasing our interactions with patients?

## Slide 19
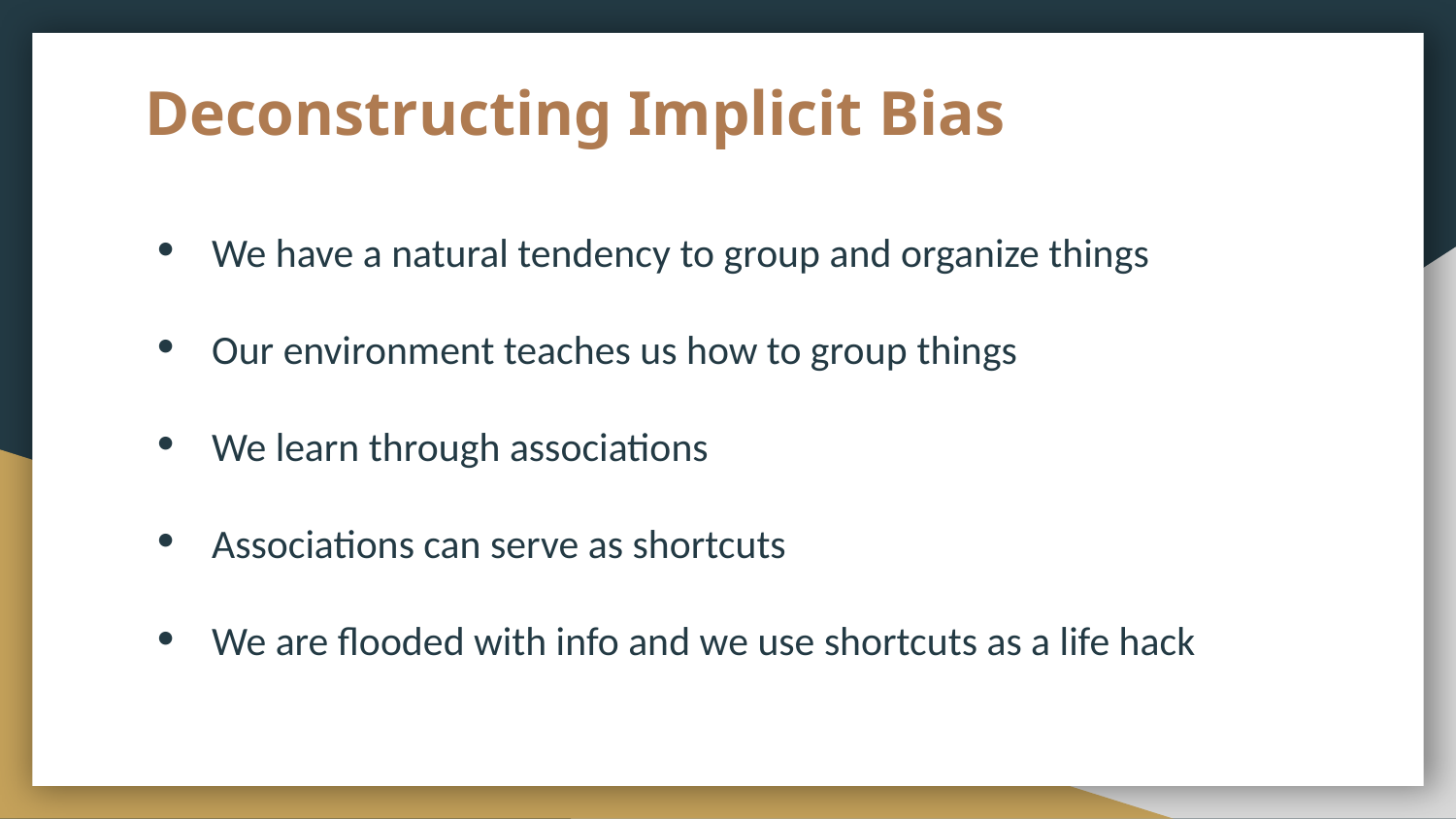

# Deconstructing Implicit Bias
We have a natural tendency to group and organize things
Our environment teaches us how to group things
We learn through associations
Associations can serve as shortcuts
We are flooded with info and we use shortcuts as a life hack

## Slide 20
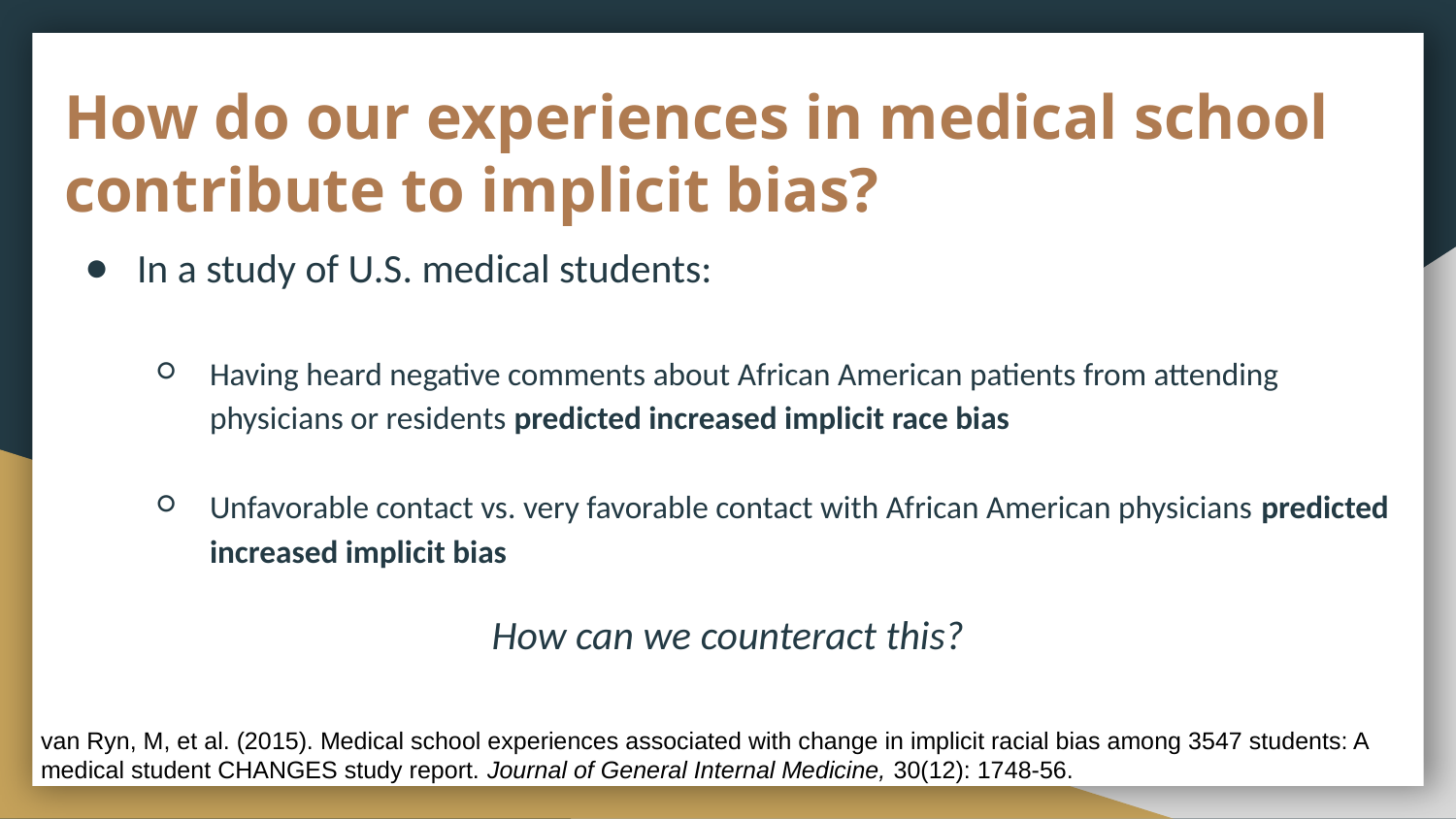

# How do our experiences in medical school contribute to implicit bias?
In a study of U.S. medical students:
Having heard negative comments about African American patients from attending physicians or residents predicted increased implicit race bias
Unfavorable contact vs. very favorable contact with African American physicians predicted increased implicit bias
How can we counteract this?
van Ryn, M, et al. (2015). Medical school experiences associated with change in implicit racial bias among 3547 students: A medical student CHANGES study report. Journal of General Internal Medicine, 30(12): 1748-56.

## Slide 21
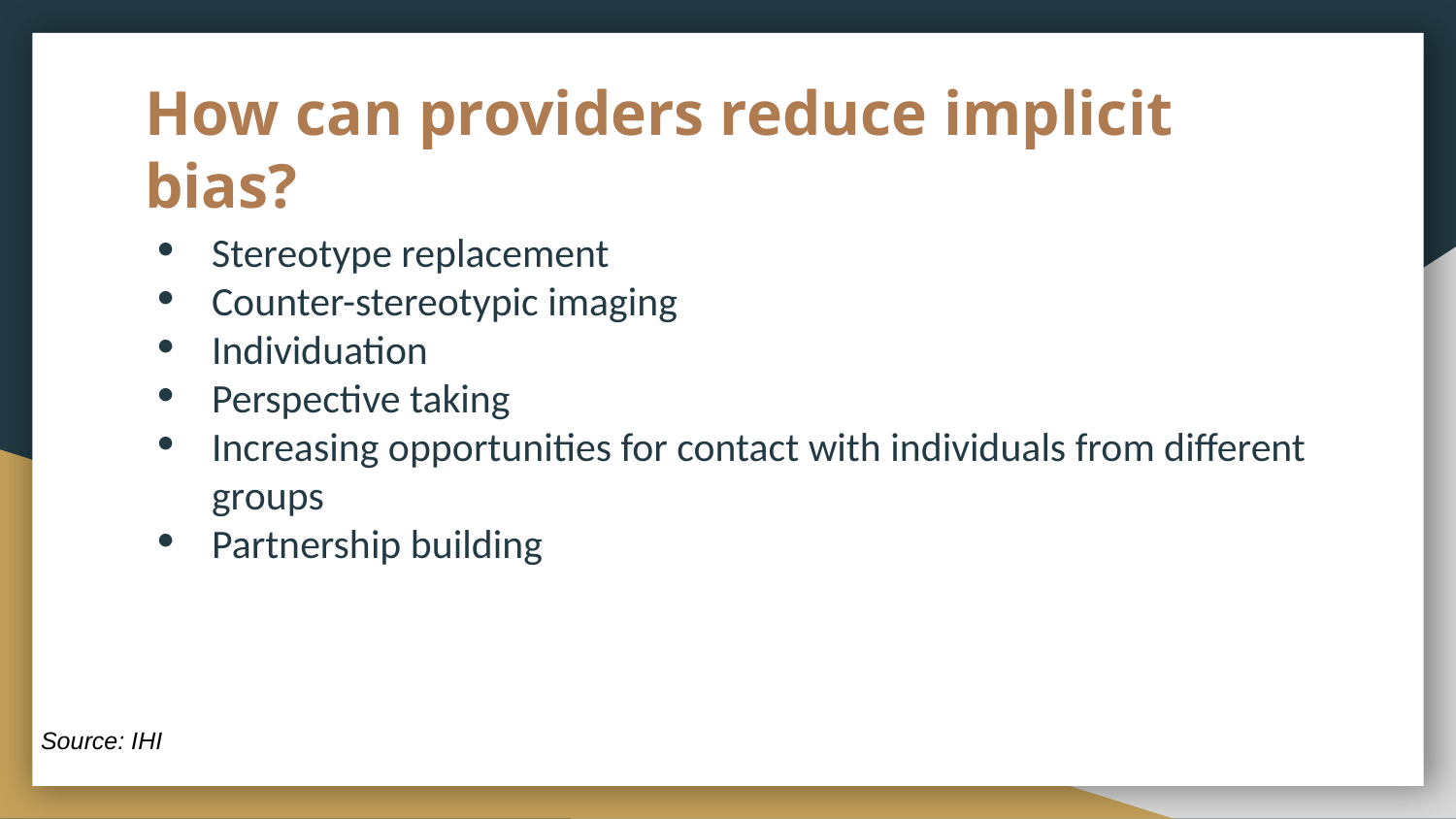

# How can providers reduce implicit bias?
Stereotype replacement
Counter-stereotypic imaging
Individuation
Perspective taking
Increasing opportunities for contact with individuals from different groups
Partnership building
Source: IHI

## Slide 22
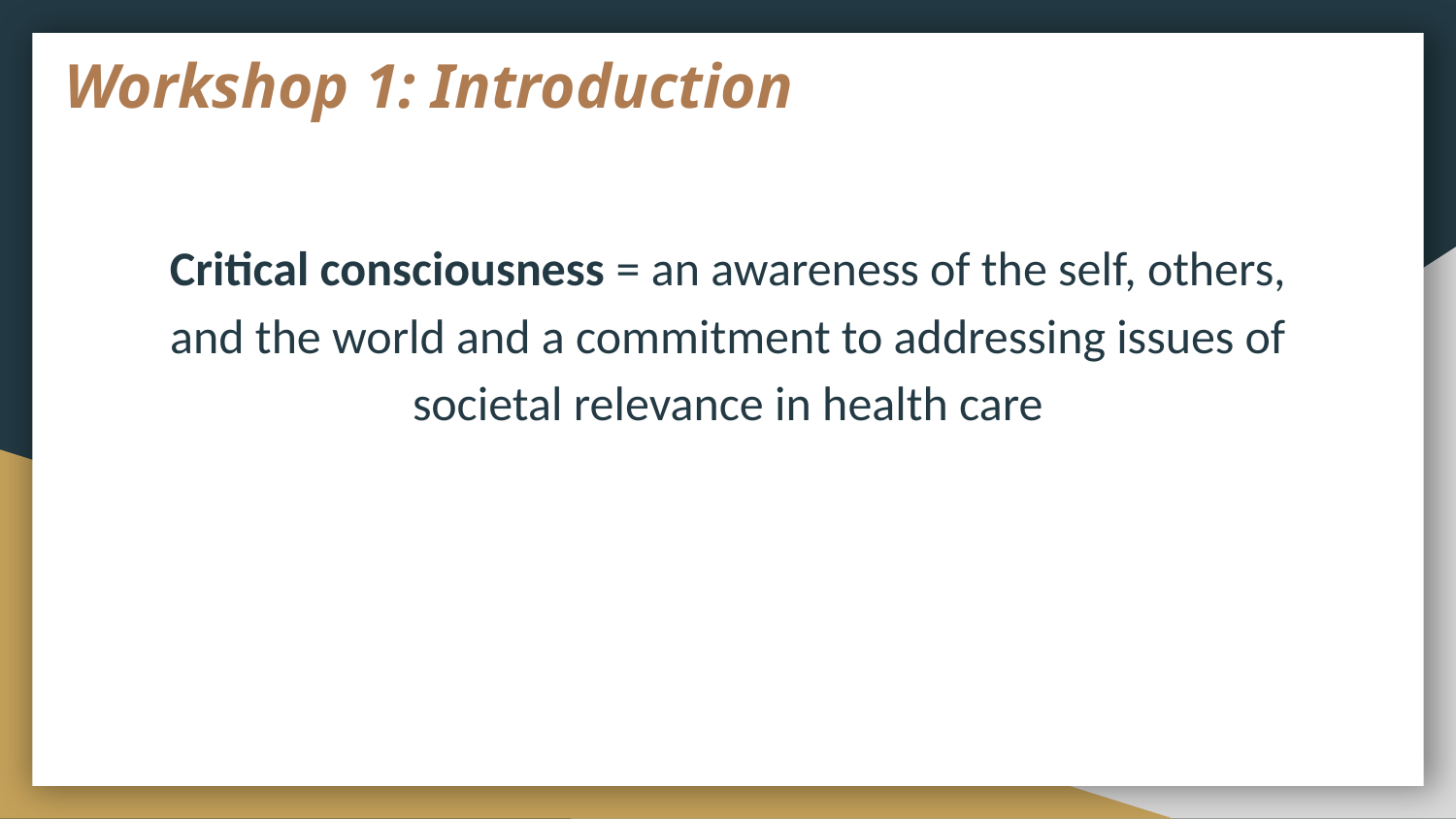

# Workshop 1: Introduction
Critical consciousness = an awareness of the self, others, and the world and a commitment to addressing issues of societal relevance in health care

## Slide 23
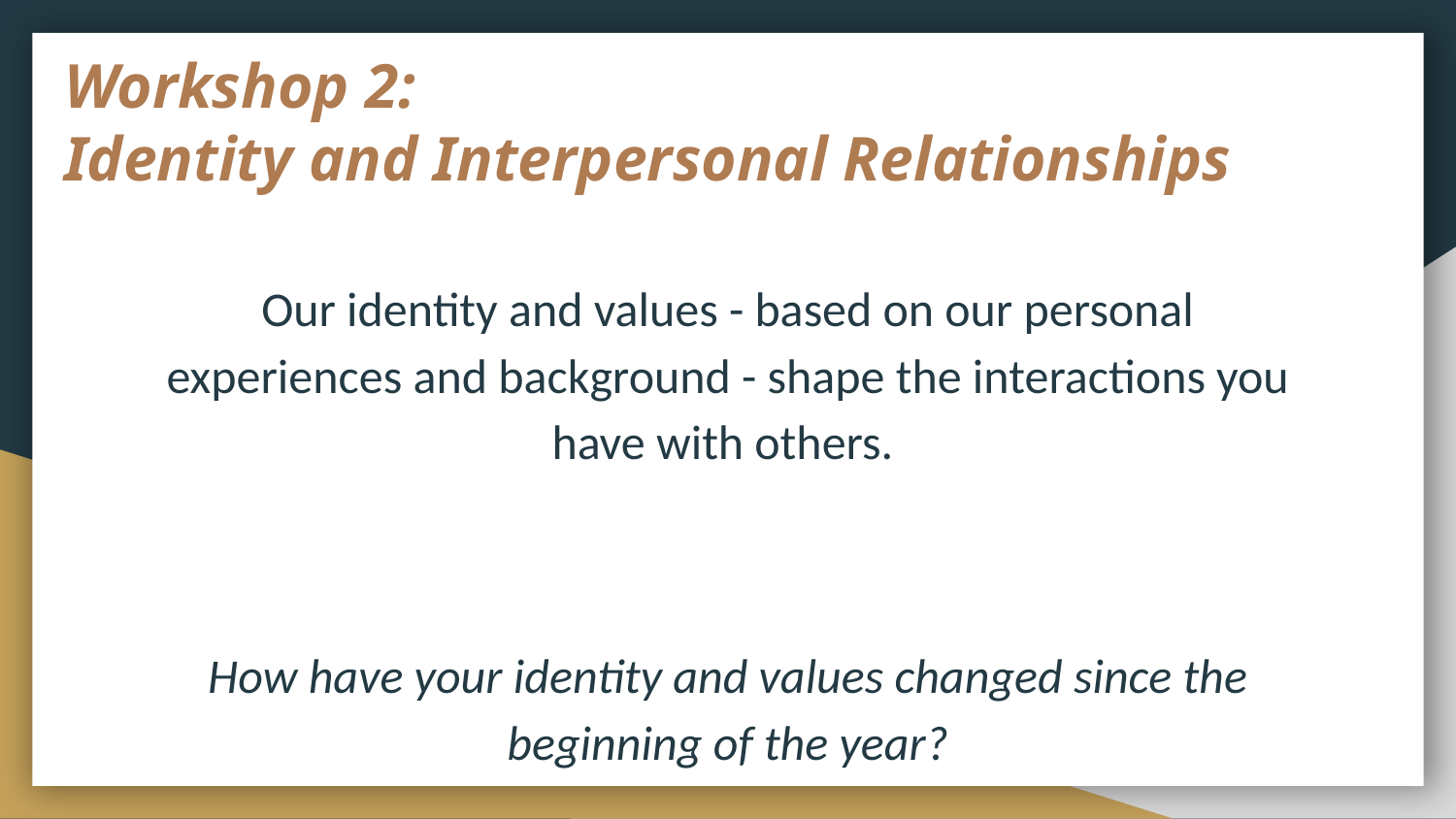

# Workshop 2: Identity and Interpersonal Relationships
Our identity and values - based on our personal experiences and background - shape the interactions you have with others.
How have your identity and values changed since the beginning of the year?

## Slide 24
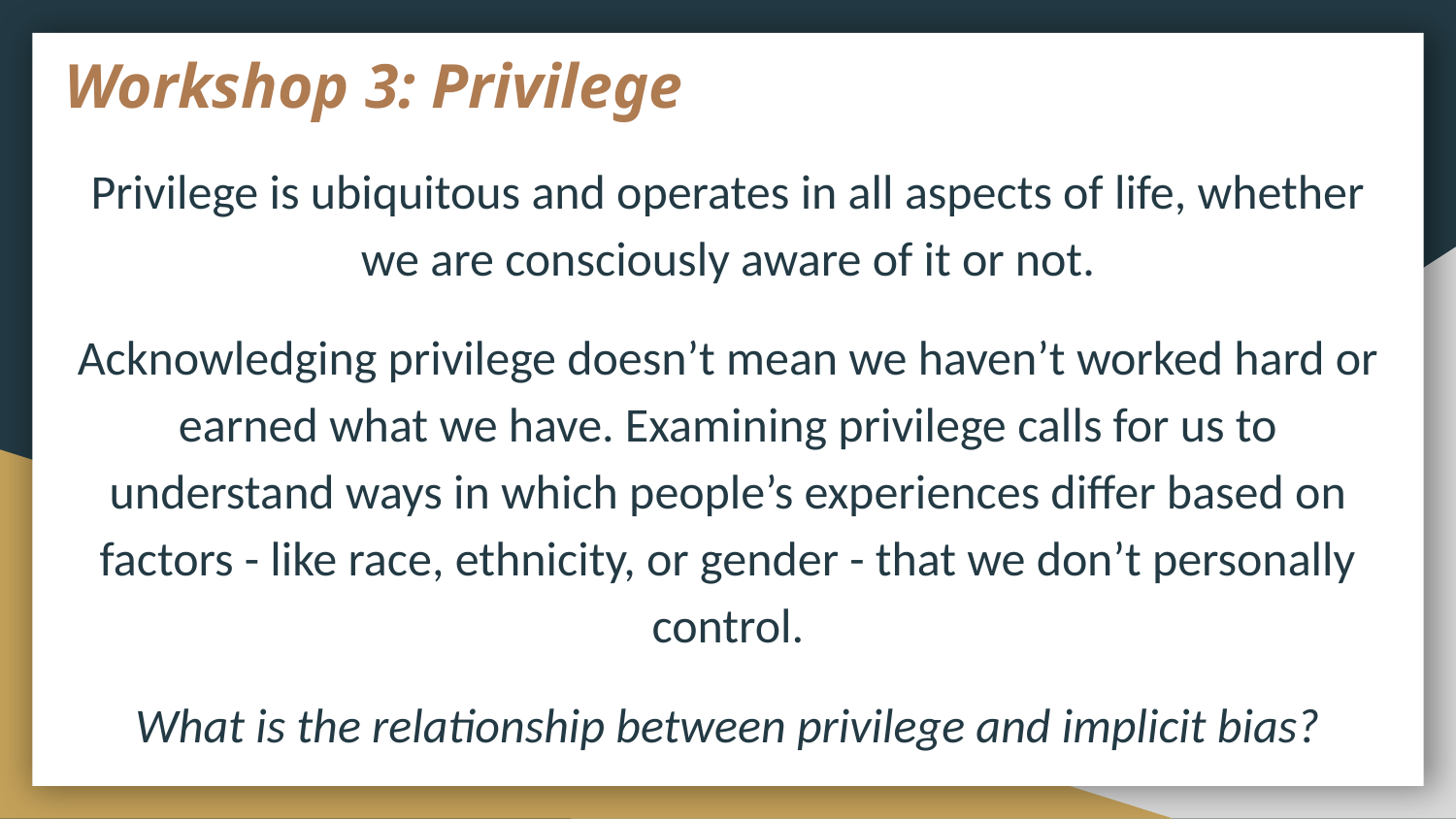

# Workshop 3: Privilege
Privilege is ubiquitous and operates in all aspects of life, whether we are consciously aware of it or not.
Acknowledging privilege doesn’t mean we haven’t worked hard or earned what we have. Examining privilege calls for us to understand ways in which people’s experiences differ based on factors - like race, ethnicity, or gender - that we don’t personally control.
What is the relationship between privilege and implicit bias?

## Slide 25
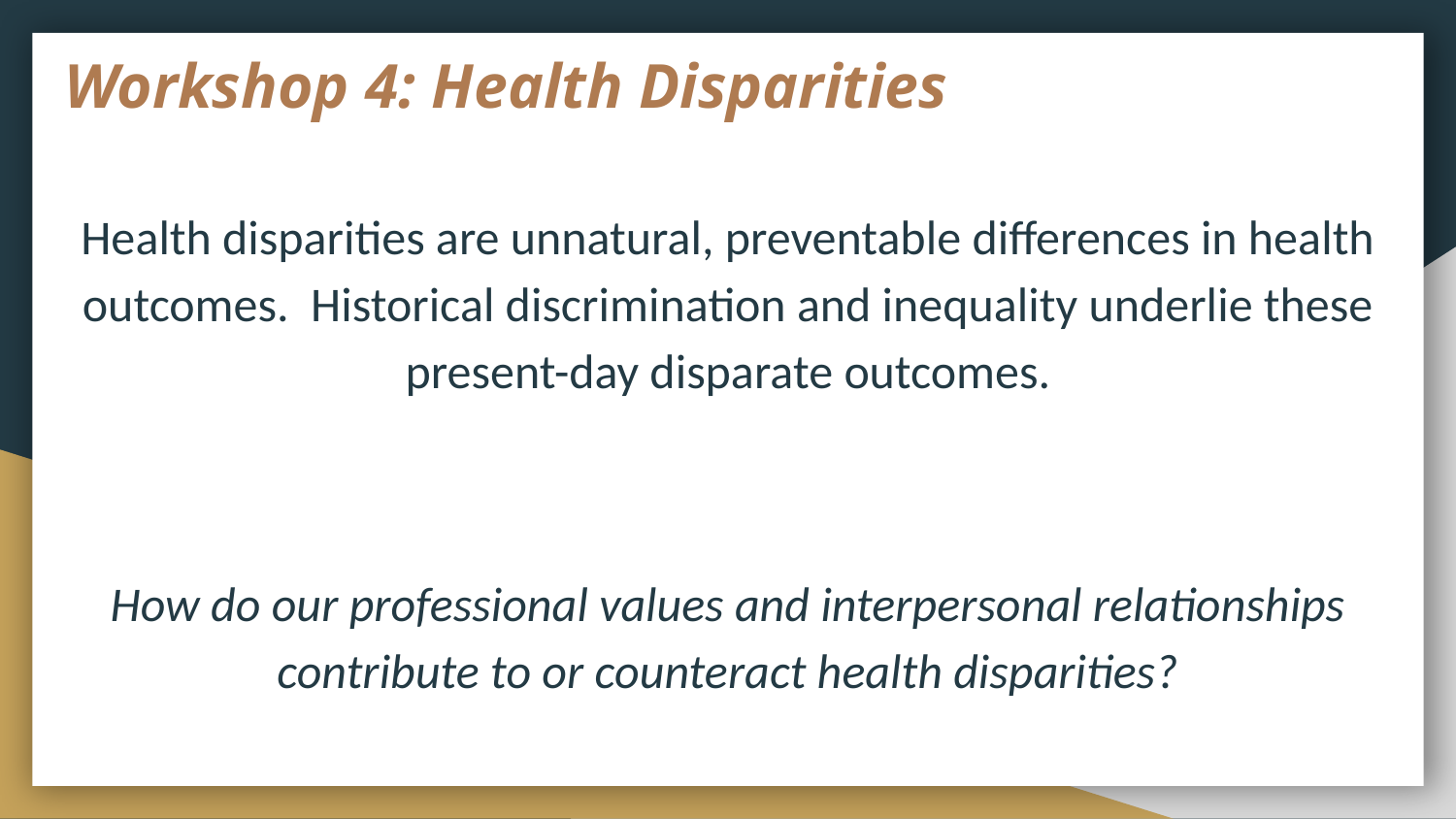

# Workshop 4: Health Disparities
Health disparities are unnatural, preventable differences in health outcomes. Historical discrimination and inequality underlie these present-day disparate outcomes.
How do our professional values and interpersonal relationships contribute to or counteract health disparities?

## Slide 26
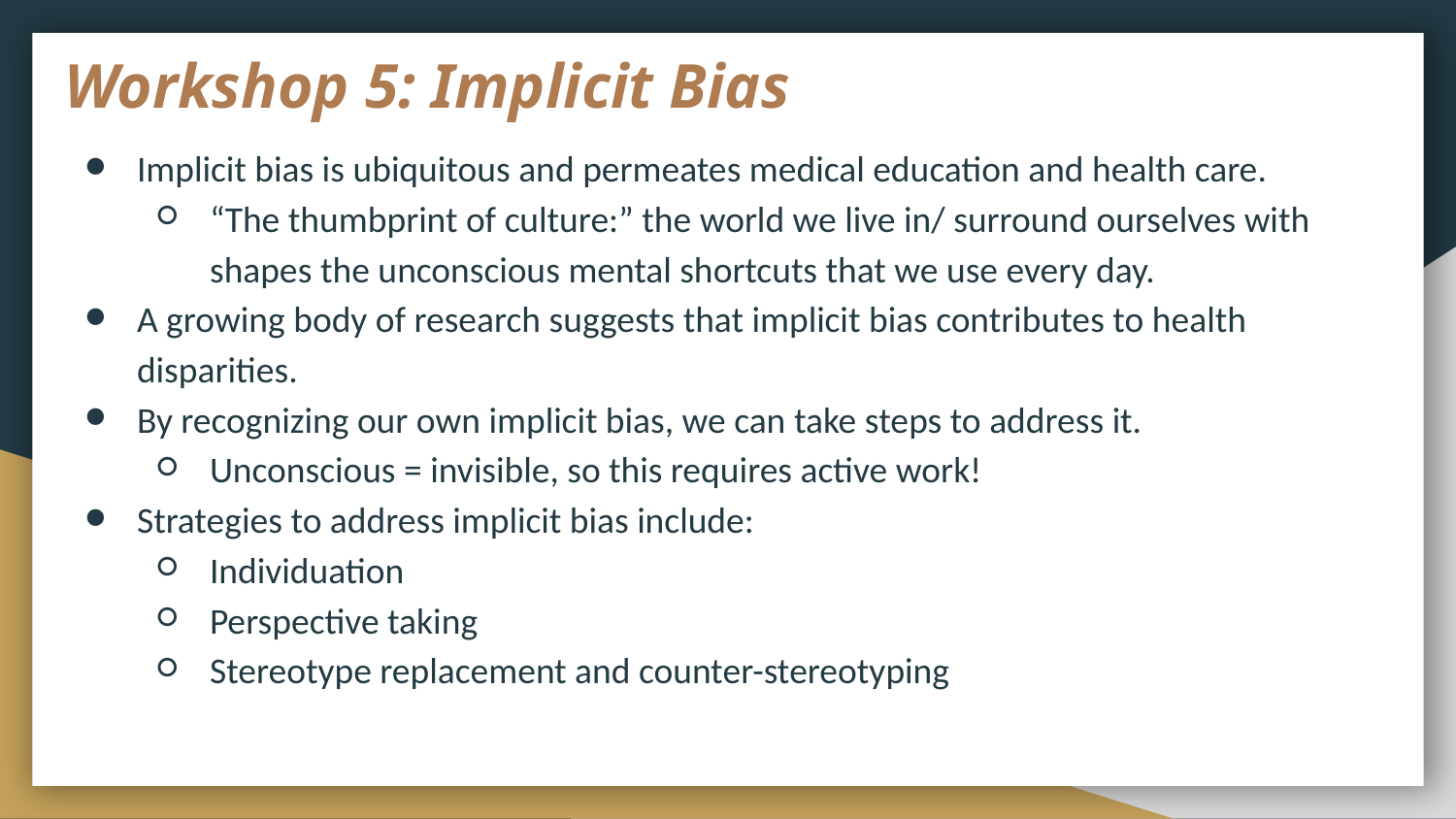

# Workshop 5: Implicit Bias
Implicit bias is ubiquitous and permeates medical education and health care.
“The thumbprint of culture:” the world we live in/ surround ourselves with shapes the unconscious mental shortcuts that we use every day.
A growing body of research suggests that implicit bias contributes to health disparities.
By recognizing our own implicit bias, we can take steps to address it.
Unconscious = invisible, so this requires active work!
Strategies to address implicit bias include:
Individuation
Perspective taking
Stereotype replacement and counter-stereotyping

## Slide 27
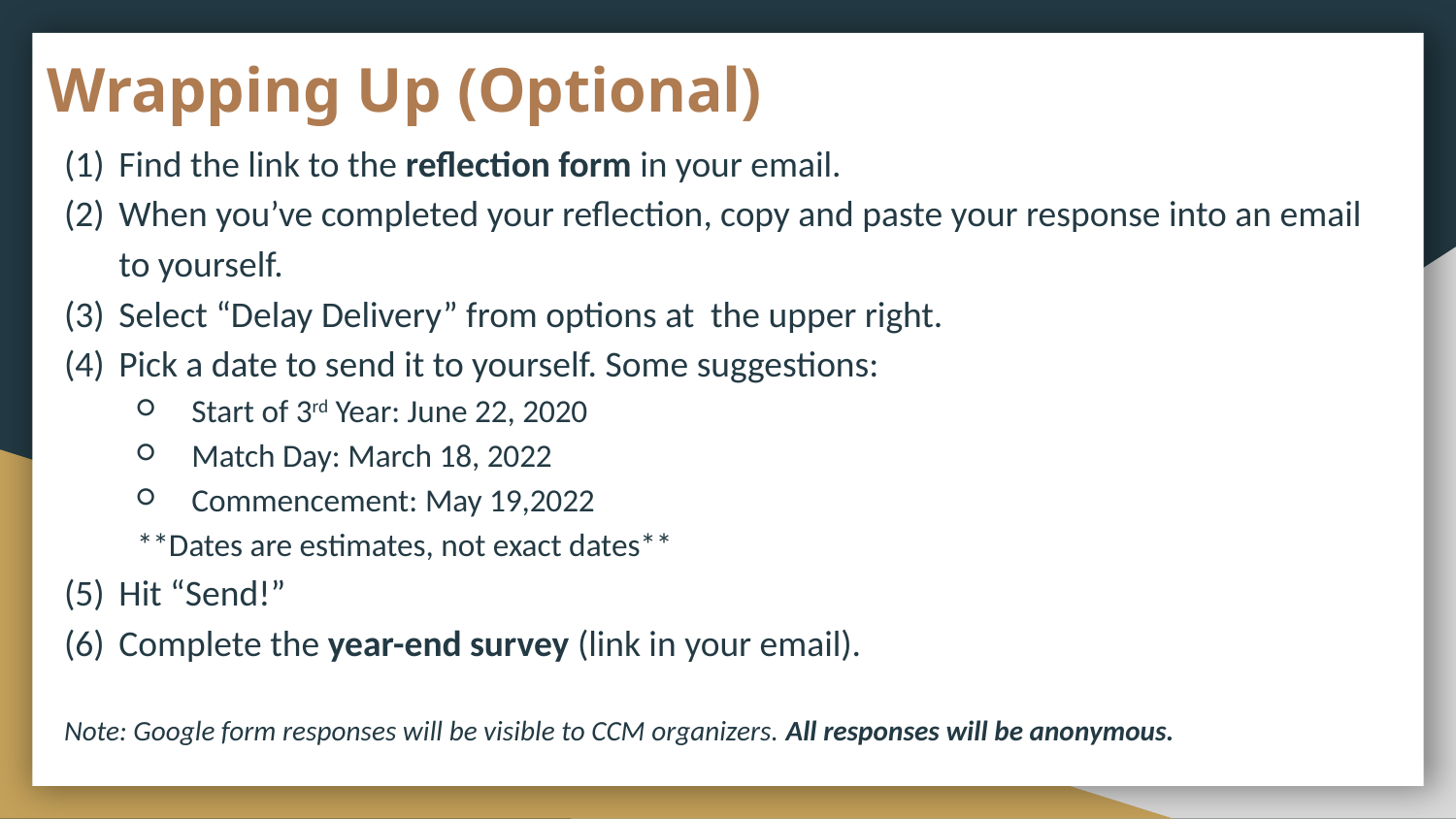

# Wrapping Up (Optional)
Find the link to the reflection form in your email.
When you’ve completed your reflection, copy and paste your response into an email to yourself.
Select “Delay Delivery” from options at the upper right.
Pick a date to send it to yourself. Some suggestions:
Start of 3rd Year: June 22, 2020
Match Day: March 18, 2022
Commencement: May 19,2022
**Dates are estimates, not exact dates**
Hit “Send!”
Complete the year-end survey (link in your email).
Note: Google form responses will be visible to CCM organizers. All responses will be anonymous.
