# Supplementary material for: Critical Consciousness as a Framework for Health Equity–Focused Peer Learning
Source: MedEdPORTAL. 2021 Apr 28;17:11145. doi: 10.15766/mep_2374-8265.11145 (PMC8079426; doi:10.15766/mep_2374-8265.11145)
Supplement: Supplementary file 1 — Workshop 1 Presentation.pptxWorkshop 1 Student Handout.docxWorkshop 2 Presentation.pptxWorkshop 2 Student Handout.docxWorkshop 3 Presentation.pptxWorkshop 3 Student Handout.docxWorkshop 4 Presentation.pptxWorkshop 5 Presentation.pptxFacilitator Orientation.pptxWorkshop 1 Facilitator Guide.docxWorkshop 2 Facilitator Guide.docxWorkshop 3 Facilitator Guide.docxWorkshop 4 Facilitator Guide.docxWorkshop 5 Facilitator Guide.docxEvaluation Tools.docx [file mep_2374-8265.11145-s001.zip › I. Facilitator Orientation.pptx]

## Slide 1
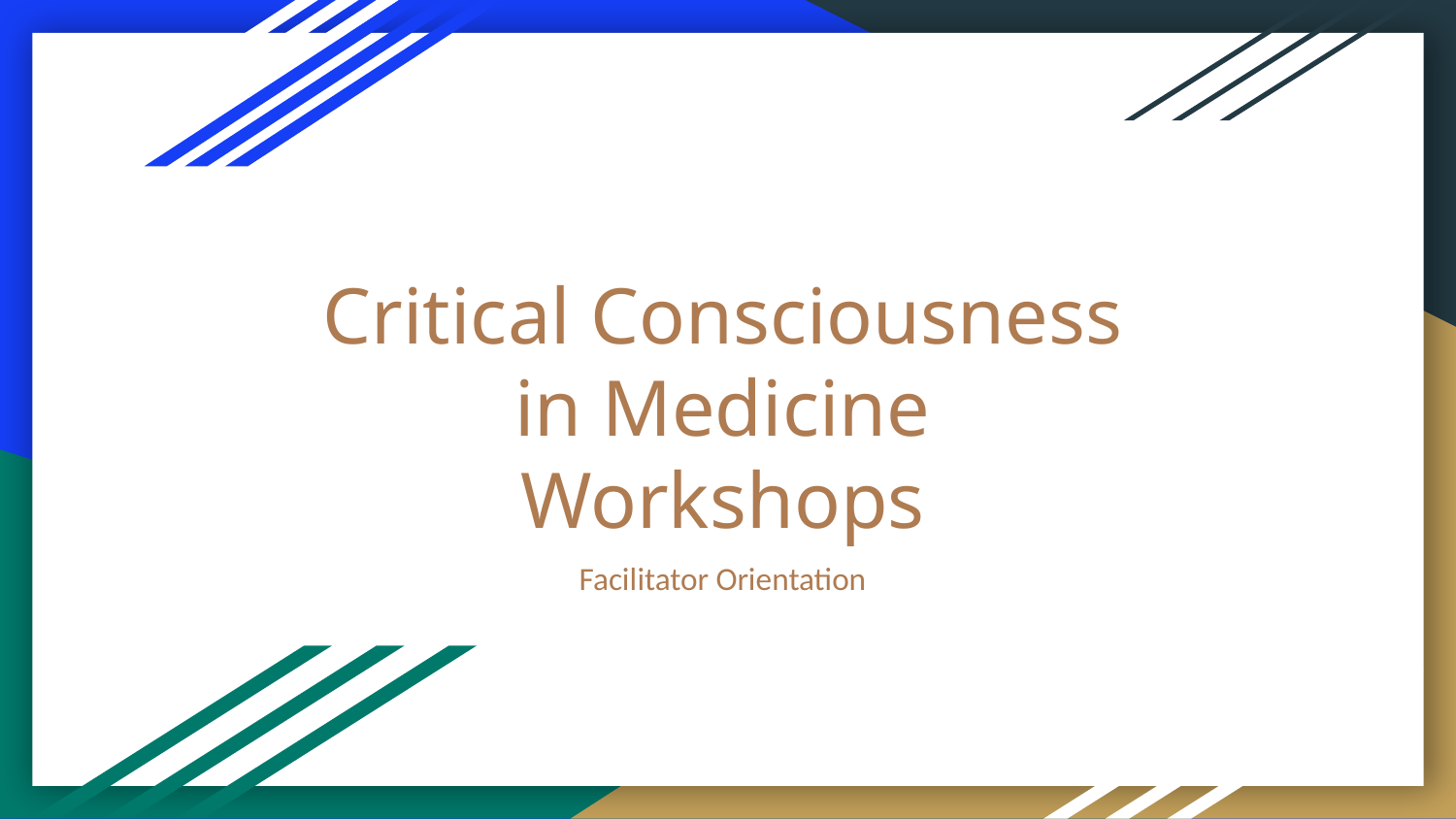

# Critical Consciousness in Medicine Workshops
Facilitator Orientation

## Slide 2
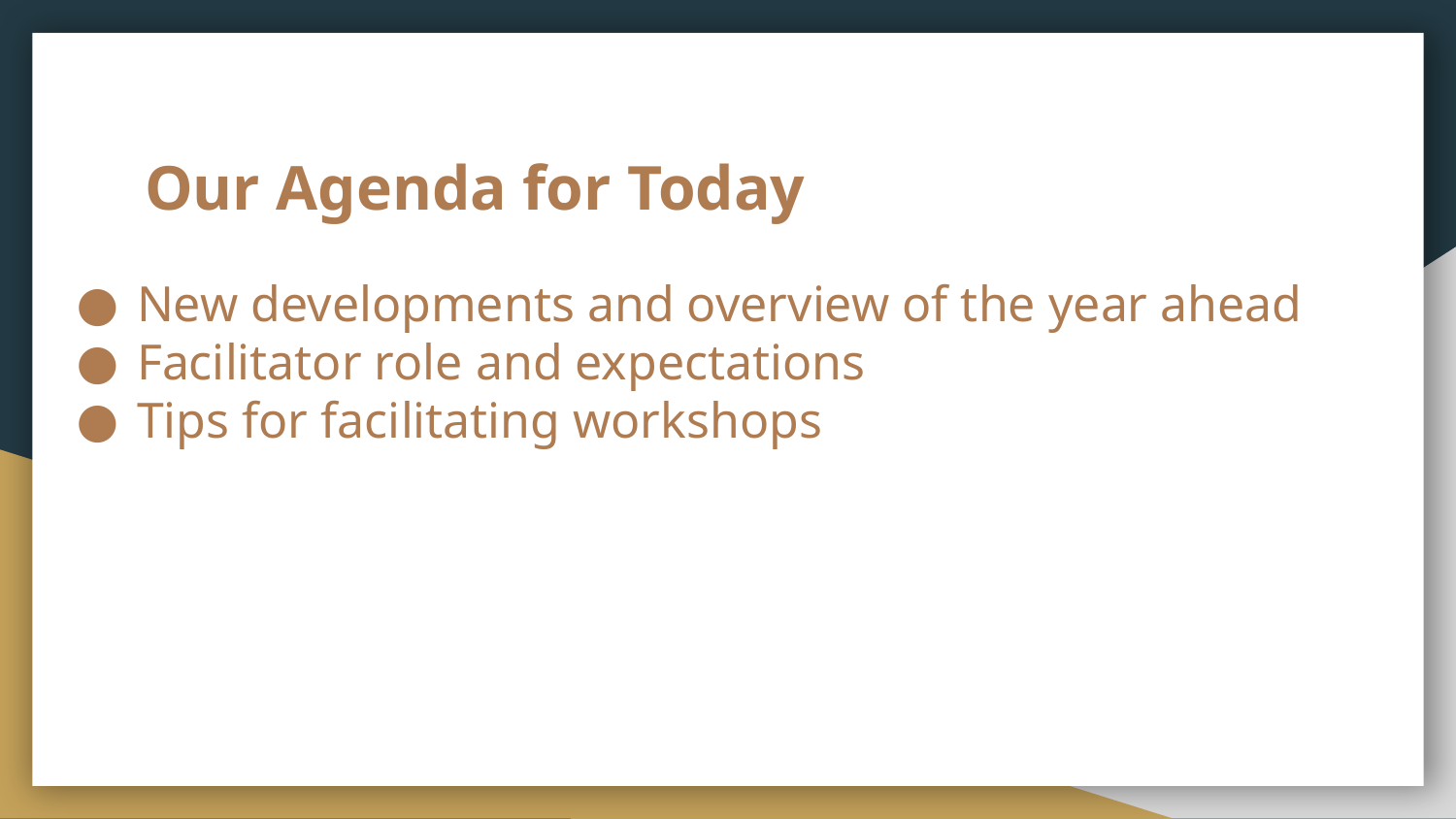

# Our Agenda for Today
New developments and overview of the year ahead
Facilitator role and expectations
Tips for facilitating workshops

## Slide 3
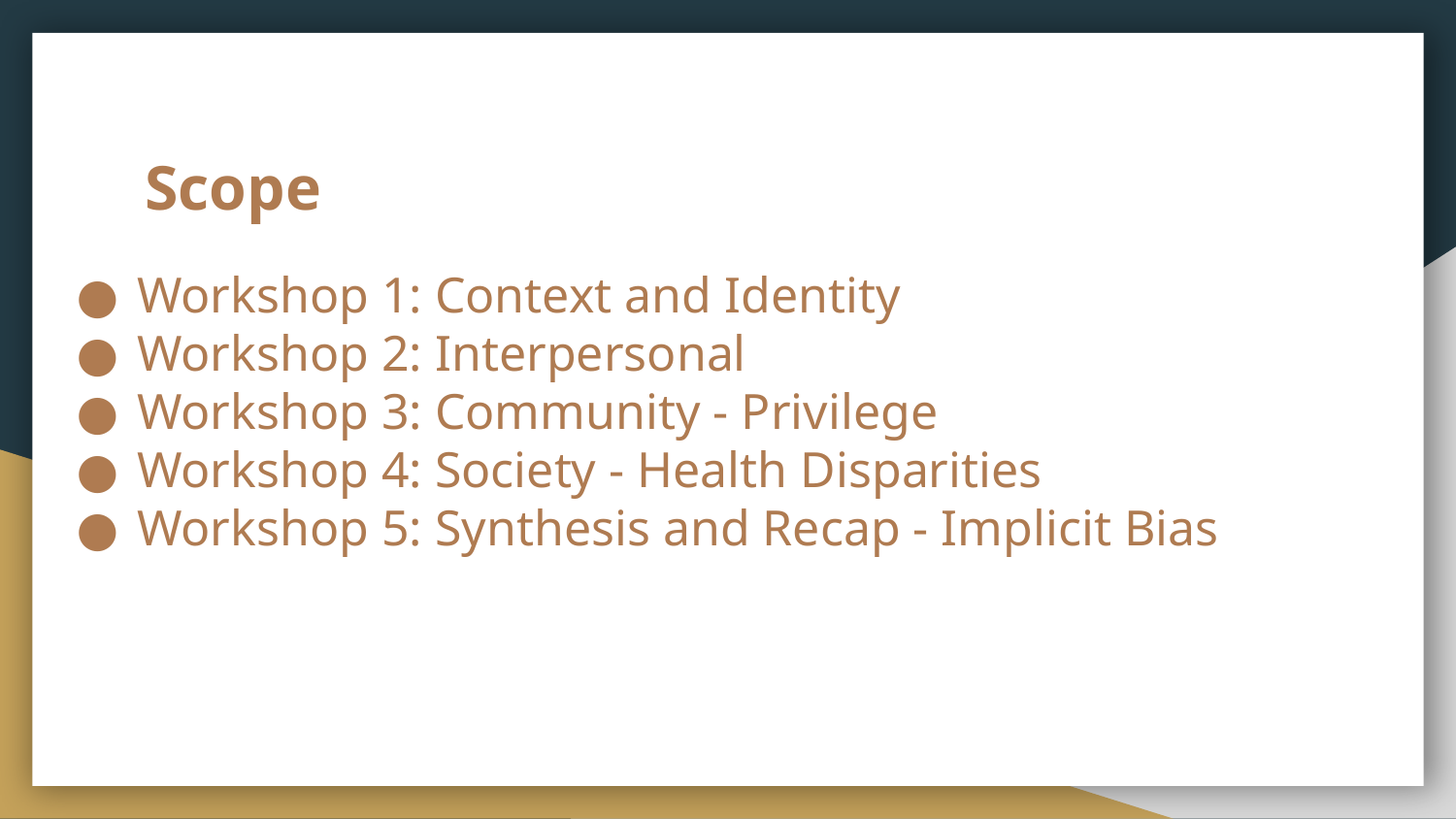

# Scope
Workshop 1: Context and Identity
Workshop 2: Interpersonal
Workshop 3: Community - Privilege
Workshop 4: Society - Health Disparities
Workshop 5: Synthesis and Recap - Implicit Bias

## Slide 4
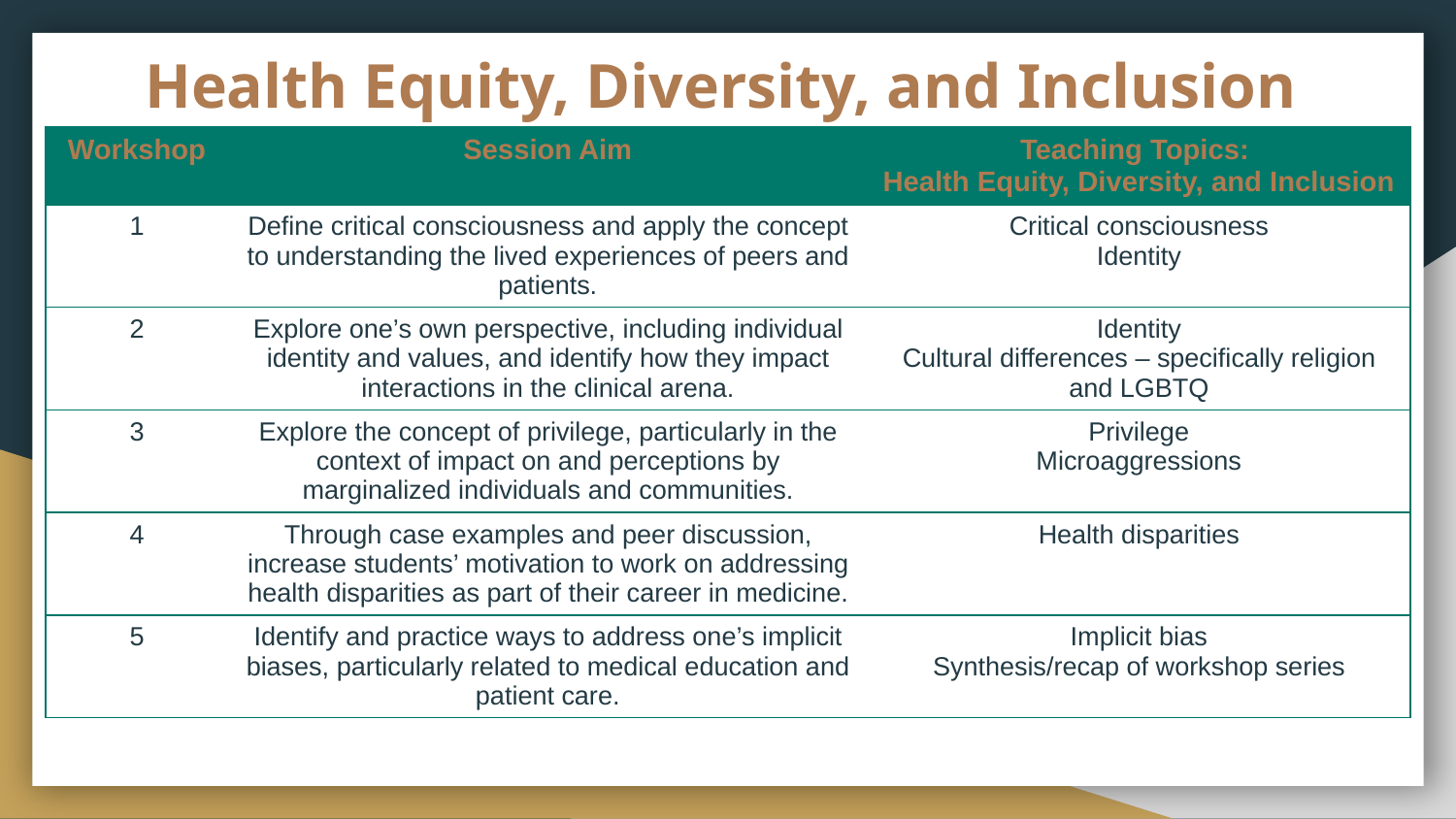

# Health Equity, Diversity, and Inclusion
| Workshop | Session Aim | Teaching Topics: Health Equity, Diversity, and Inclusion |
| --- | --- | --- |
| 1 | Define critical consciousness and apply the concept to understanding the lived experiences of peers and patients. | Critical consciousness Identity |
| 2 | Explore one’s own perspective, including individual identity and values, and identify how they impact interactions in the clinical arena. | Identity Cultural differences – specifically religion and LGBTQ |
| 3 | Explore the concept of privilege, particularly in the context of impact on and perceptions by marginalized individuals and communities. | Privilege Microaggressions |
| 4 | Through case examples and peer discussion, increase students’ motivation to work on addressing health disparities as part of their career in medicine. | Health disparities |
| 5 | Identify and practice ways to address one’s implicit biases, particularly related to medical education and patient care. | Implicit bias Synthesis/recap of workshop series |

## Slide 5
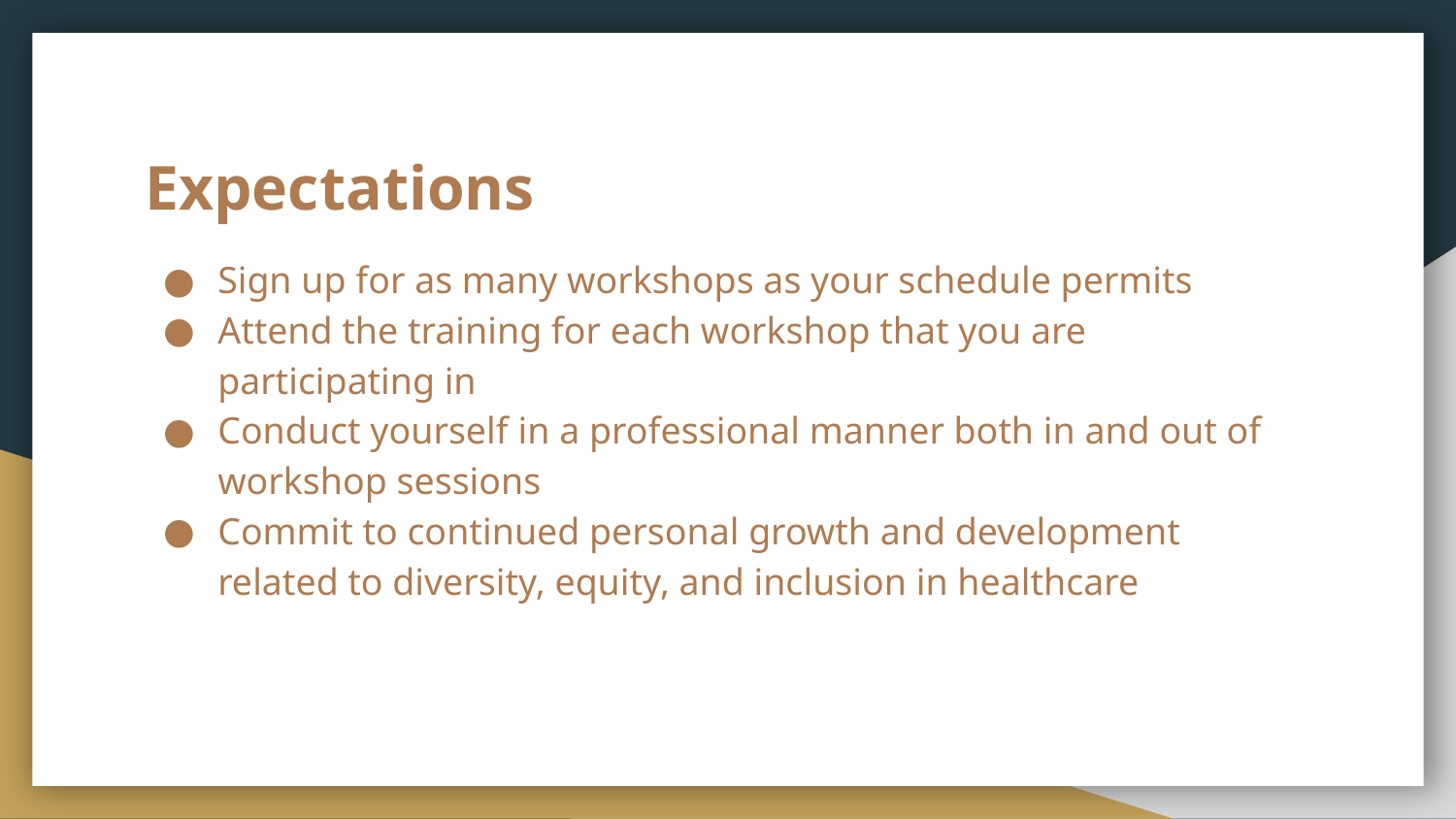

# Expectations
Sign up for as many workshops as your schedule permits
Attend the training for each workshop that you are participating in
Conduct yourself in a professional manner both in and out of workshop sessions
Commit to continued personal growth and development related to diversity, equity, and inclusion in healthcare

## Slide 6
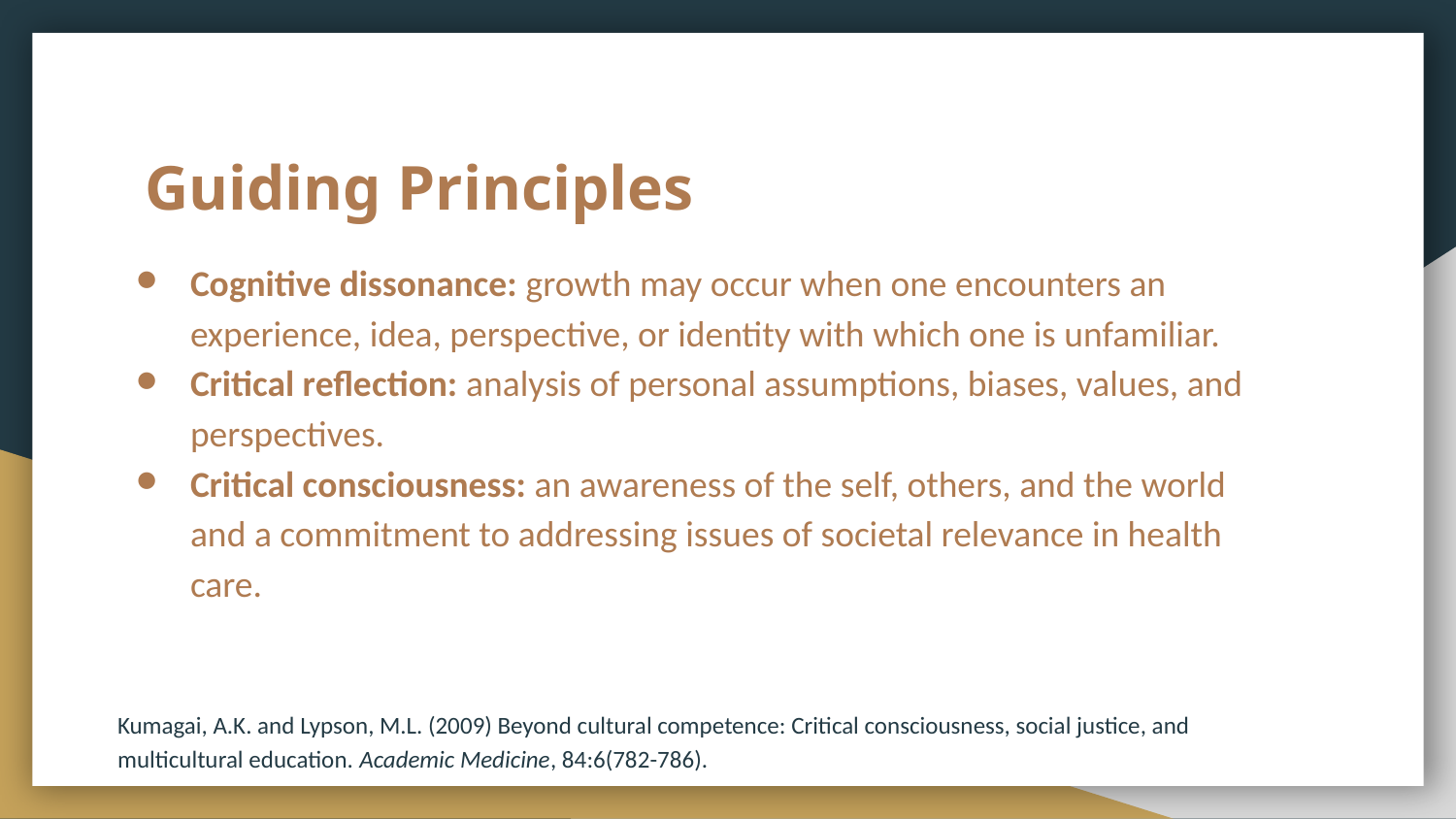

# Guiding Principles
Cognitive dissonance: growth may occur when one encounters an experience, idea, perspective, or identity with which one is unfamiliar.
Critical reflection: analysis of personal assumptions, biases, values, and perspectives.
Critical consciousness: an awareness of the self, others, and the world and a commitment to addressing issues of societal relevance in health care.
Kumagai, A.K. and Lypson, M.L. (2009) Beyond cultural competence: Critical consciousness, social justice, and multicultural education. Academic Medicine, 84:6(782-786).

## Slide 7
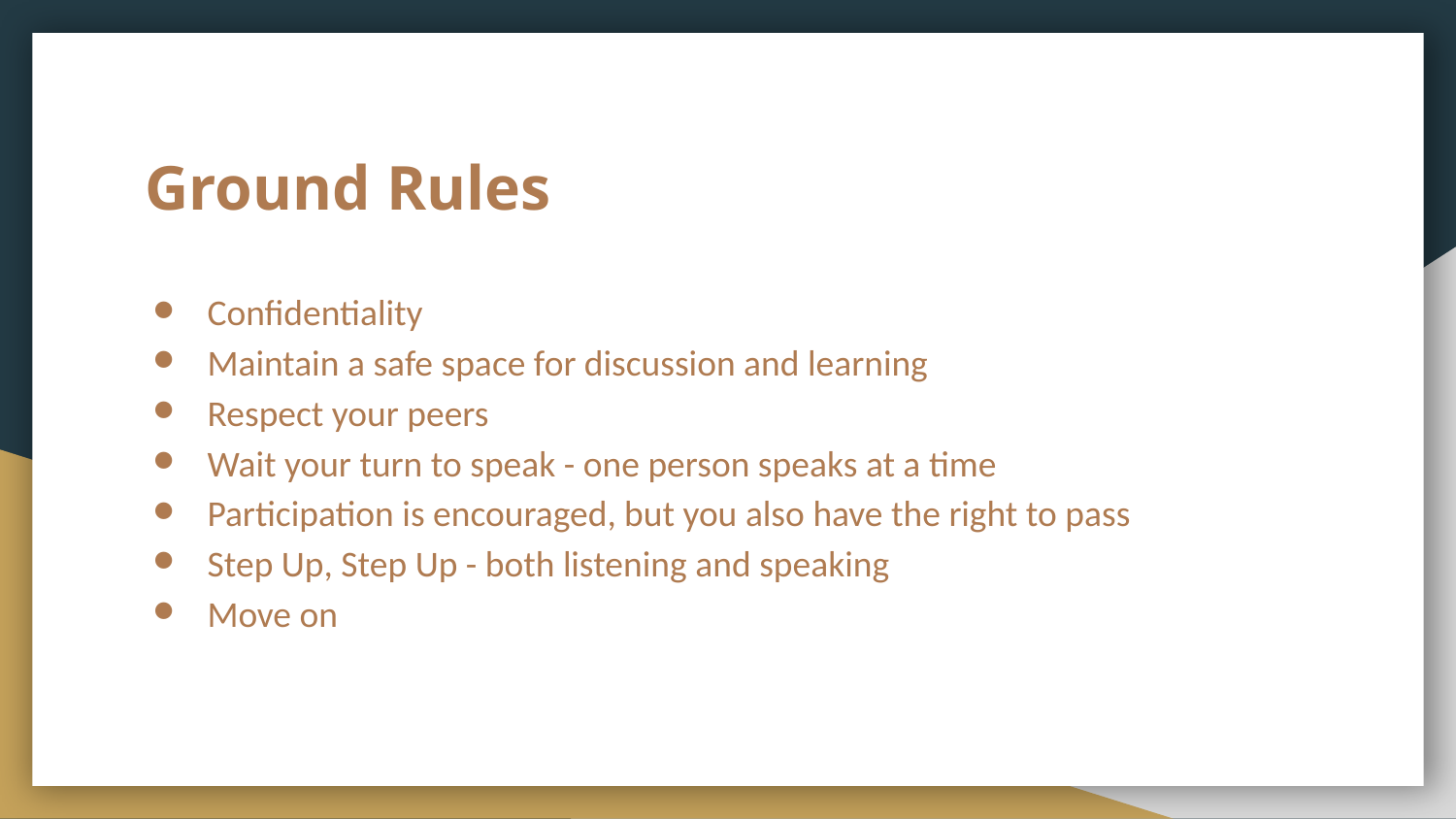

# Ground Rules
Confidentiality
Maintain a safe space for discussion and learning
Respect your peers
Wait your turn to speak - one person speaks at a time
Participation is encouraged, but you also have the right to pass
Step Up, Step Up - both listening and speaking
Move on

## Slide 8
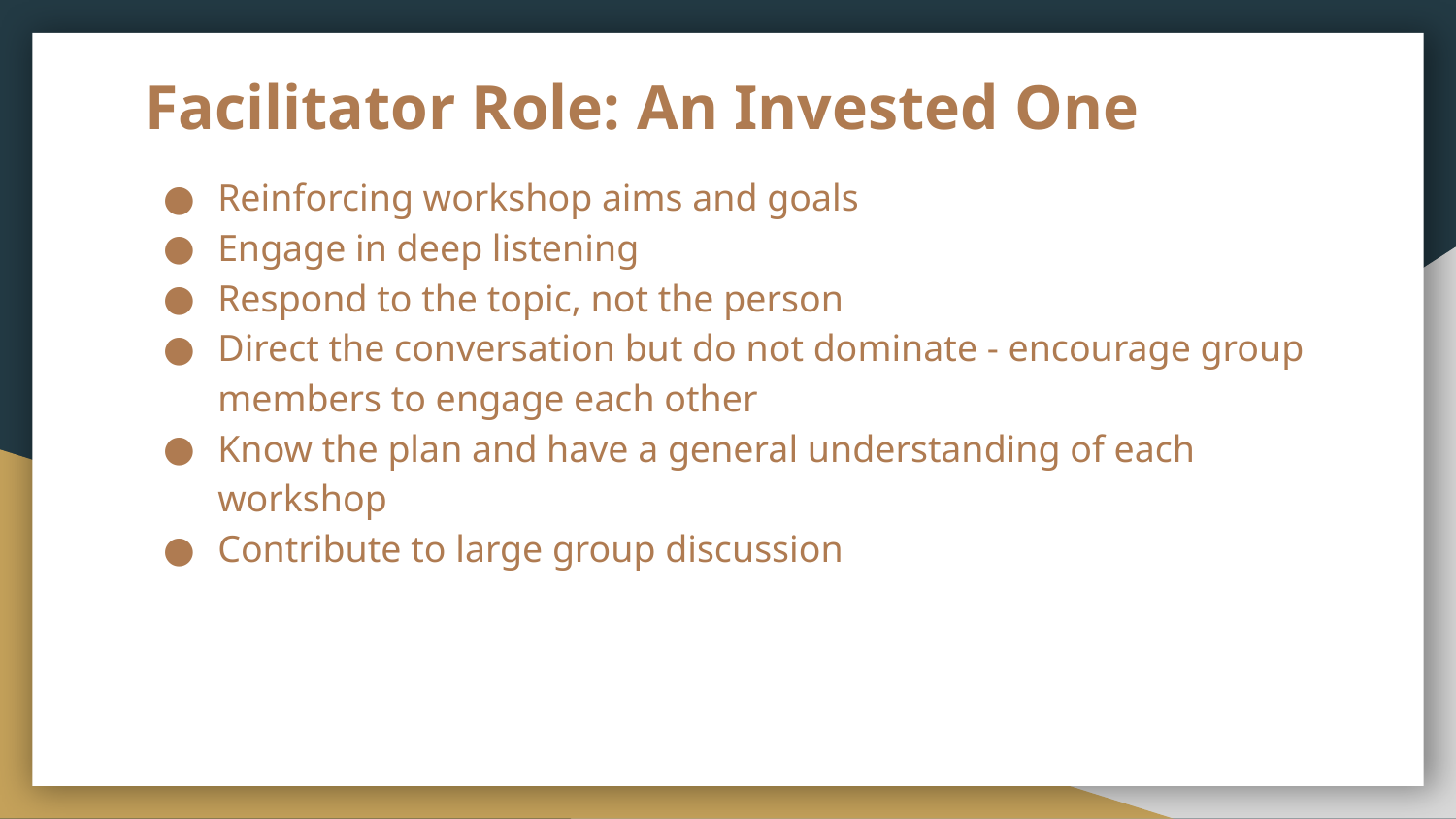

# Facilitator Role: An Invested One
Reinforcing workshop aims and goals
Engage in deep listening
Respond to the topic, not the person
Direct the conversation but do not dominate - encourage group members to engage each other
Know the plan and have a general understanding of each workshop
Contribute to large group discussion
Source: Interaction Institute for Social Change

## Slide 9
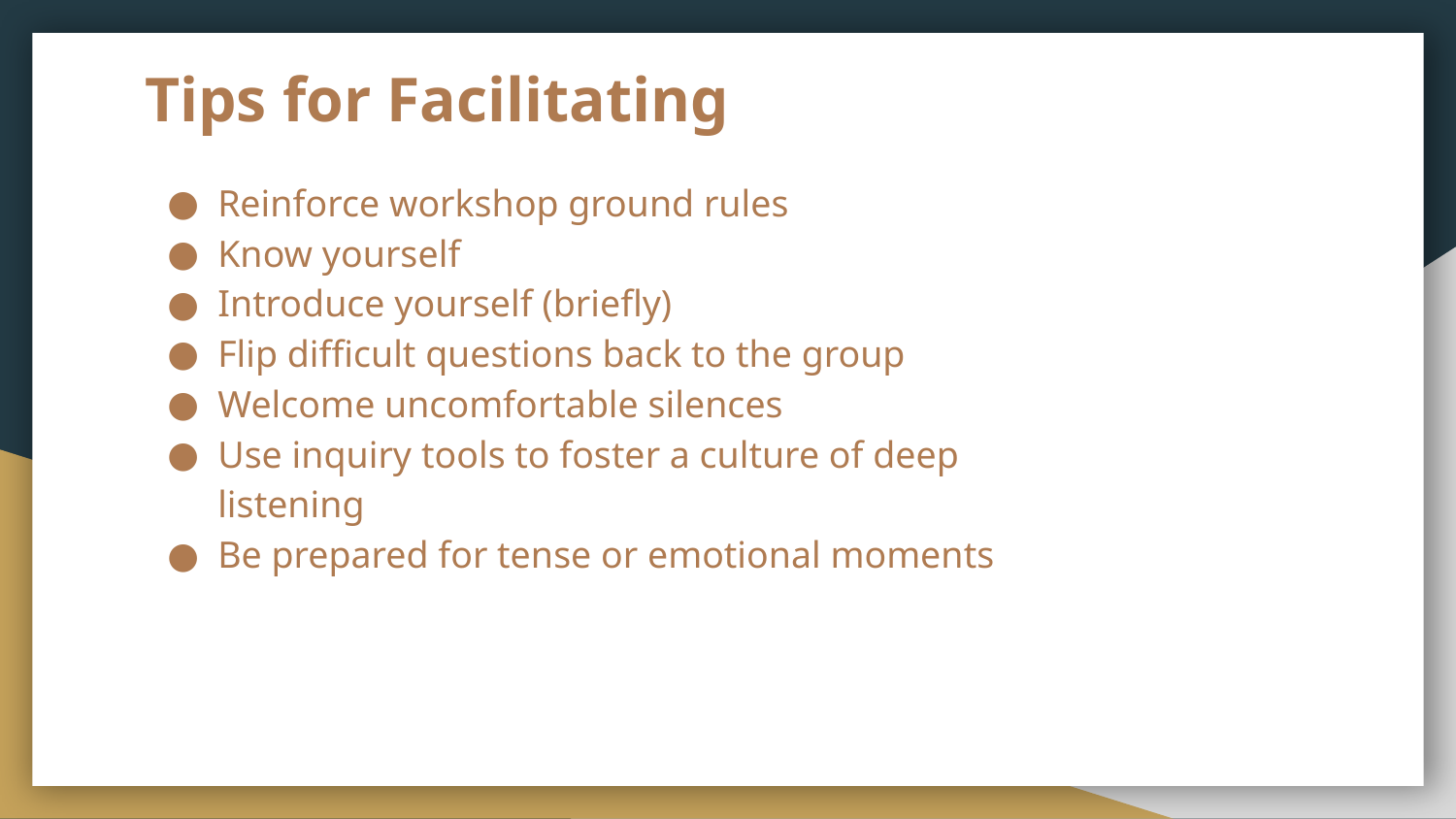

# Tips for Facilitating
Reinforce workshop ground rules
Know yourself
Introduce yourself (briefly)
Flip difficult questions back to the group
Welcome uncomfortable silences
Use inquiry tools to foster a culture of deep
listening
Be prepared for tense or emotional moments
Source: Interaction Institute for Social CHange

## Slide 10
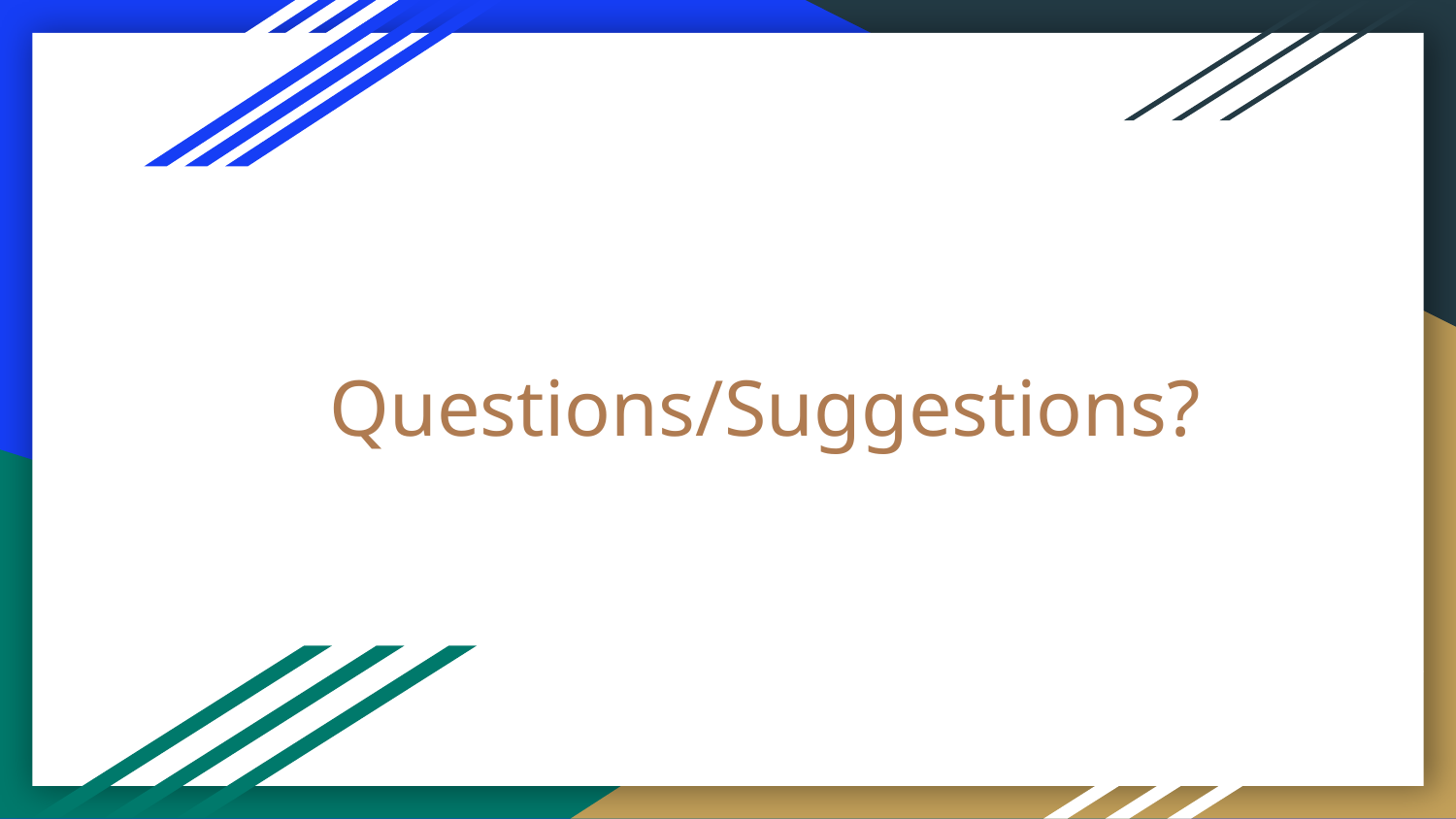

# Questions/Suggestions?
