# Supplementary material for: Critical Consciousness as a Framework for Health Equity–Focused Peer Learning
Source: MedEdPORTAL. 2021 Apr 28;17:11145. doi: 10.15766/mep_2374-8265.11145 (PMC8079426; doi:10.15766/mep_2374-8265.11145)
Supplement: Supplementary file 1 — Workshop 1 Presentation.pptxWorkshop 1 Student Handout.docxWorkshop 2 Presentation.pptxWorkshop 2 Student Handout.docxWorkshop 3 Presentation.pptxWorkshop 3 Student Handout.docxWorkshop 4 Presentation.pptxWorkshop 5 Presentation.pptxFacilitator Orientation.pptxWorkshop 1 Facilitator Guide.docxWorkshop 2 Facilitator Guide.docxWorkshop 3 Facilitator Guide.docxWorkshop 4 Facilitator Guide.docxWorkshop 5 Facilitator Guide.docxEvaluation Tools.docx [file mep_2374-8265.11145-s001.zip › J. Workshop 1 Facilitator Guide.docx]

Facilitator Guide
Critical Consciousness in Medicine Workshop #1: Introduction and Context

Summary Table: How CCM Workshop #1 Teaches Pre-Clinical Medical Students About Diversity, Inclusion, and Health Equity

| *Overall Goal* | *Learning Objectives* | *Associated Activities* | *Anticipated Learning Outcomes* |
| --- | --- | --- | --- |
| Provide an overview of the workshop series scope, introduce guiding principles, and provide an opportunity to practice critical consciousness through critical self-reflection. | Describe the scope and sequence of this workshop series, how it fits into the medical school curriculum and its importance alongside clinical training. | Workshop presentation | Students gain an understanding of the structure and expectations of the workshop series and an appreciation for the importance of teaching about health equity, diversity, and inclusion in the medical school curriculum. |
|  | Identify the guiding principles of this workshop series. | Workshop presentation | Students are able to define key terms such as cognitive dissonance, critical reflection, and critical consciousness. |
|  | Begin to critically self-reflect on one’s individual identity and its role in shaping perspective. | Identity activity | Students highlight aspects of their personal identity and gain an appreciation for the diversity of their peers through partner conversation and whole room discussion. |

| **WHEN** | **WHAT** | **WHO** |
| --- | --- | --- |
| 25 min. | *[Slides 1-7]: Welcome, opening remarks, “making the case” for this workshop series*   - [2] Introduce the workshop organizers - [3] Transition slide - [4] Overview of today’s learning objectives - [5] This quote addresses the rationale for including workshops like this in medical education   - Medical schools across the country and throughout the world are finding ways to teach about health equity, diversity and inclusion   - This reflects the fact that medicine is not removed from the greater world – the issues that we face in society are also reflected in the medical community   - As medical professionals, we need to address these issues head-on – for the well-being of our patients, our co-workers, and our communitieis - [6] Moving beyond cultural competence   - This workshop series is part of a broader movement to enhance medical student training related to health equity, diversity, and inclusion. Many of you may be familiar with the term “cultural competence” – we are seeking to move beyond that notion to a deeper level of introspection, critical reflection, and understanding   - We hope this workshop series will help you contextualize the rest of your medical training and enable you to recognize societal problems that manifest themselves in medicine.   - Social justice has become a polarizing term, but we hope to speak to concerns of social justice in this workshop series, specifically acknowledging the dignity and autonomy of all members of society and the importance of delivery high-quality medical care to everyone (Kumagai and Lypson definition) - [7] Why should we care?   - This slide seeks to “make the case” for this workshop series by addressing common areas of concern for students. Key points:   - This workshop series will involve difficult conversations. We encourage to embrace those an opportunity for “stretch” and learning   - Understanding social and cultural issues – health equity, diversity and inclusion – is essential to good medical care   - We all share a responsibility for addressing discrimination/inequity and for ensuring an inclusive environment for our peers and patients   - We will be addressing big, complex issues in these workshops. Our focus is on collective learning, not solutions (though we may encounters those, as well) | Workshop Organizers |
| 10 min. | *[Slides 8-15]: Expectations for Learners*   - [8] We will now discuss what you can expect from these workshops - [9] Overall goal   - Inspired by leaders at the University of Michigan, our workshop series is grounded in critical consciousness   - We hope to introduce you to concepts of discrimination and inequity and initiate conversation and reflection on these issues. In so doing, we hope to (1) instill professionalism, humanism, and cultural openness and humility in medical students and (2) inspire a worldview that is more complex, inclusive, and oriented towards moral action (from Kumagai and Lypson) - [10] The Workshop Team   - The team involved in designing and delivering these workshops includes medical students (both as organizers and facilitators) and faculty.   - This series is student-led, but faculty are here to help make sure these workshops are integrated within the larger curriculum and contribute to these conversations - [11] Outline the format of the workshop series. Key points:   - Mix of small group and whole room activities   - Student facilitators help to guide the conversation, but there are rarely “right” answers - [12] This slide identifies connections between CCM and other initiatives related to health equity, diversity, and inclusion in medical education, both nationally and at our institution. You may wish to edit this slide to highlight related initiatives that are relevant for your audience. - [13] Ground rules   - We introduced ground rules for the workshop series and invited students to contribute their own. You may wish to add or edit these for your audience.   - Confidentiality: if sharing a story, avoid disclosing names. Keep what is said in this room here.   - Maintain a safe space for discussion and learning: be mindful of yourself and the impact you may be having on others   - Respect your peers   - Wait your turn to speak: “one mic rule” – one person speaks at a time   - Participation is encouraged but you also have the right o to pass.   - Step up/step up – a variation of “step up/step back,” which encourages participants to be mindful of how much they are speaking. Here, we invite students to “step up” both listening and speaking, being aware of and modulating their participation.   - Move on – as participants in this workshop series, we have to embrace ambiguity and discomfort. Inevitably, we will not be able to address everything in these workshops, so we encourage outside/independent learning on the issues raised.   - Be fully engaged – keep your phones away and laptops closed unless specifically directed otherwise - [14] What will be asked of you – expectations for learners. Key points:   - Expect to participate fully, be challenged, respected, and to engage critically with complex issues - [15] Pause to invite questions/responses from students at this point | Workshop Organizers |
| 5 min | *[Slides 16]: Guiding Principles*   - [16] Definitions of key terms that will be commonly used in these workshop series (as defined by Kumagai and Lypson) - These concepts serve as guiding principles - Encourage students to seek out opportunities for critical self-reflection and - Critical consciousness, as reflected in the title of this workshop series, is foundational. We are seeking to teach students in a way that promotes an approach to understanding, as opposed to specific terms/competencies | Workshop Organizers |
| 20 min. | *[Slide 17-20]: Identity*   - [17] We are now transitioning to a more active part of the workshop, where we will talk about identity – we are looking for your participation!. Can anyone provide a definition or an example of what you think identity is? - [18] Definitions/dimensions of identity   - Note that understanding our own identity is central to develop critical consciousness – we do that in part through reflecting on who we are and what we are bringing to the conversation - [19] Identity activity   - Worksheet (5 min.) – students complete individually (see separate appendix for worksheet)     - Write five labels/descriptions that you use to describe yourself     - Write five labels/descriptions that you think others use to describe you (these words can overlap)   - Partner conversation (5 min.) – turn and talk with another person at your table (talk in pairs)     - Notice the words inside and outside of your hand outline. Which ones are the same? Which ones are different?     - Explain why some words might be the same, while others might be different - [20] Identity activity   - We invited “report outs” from any students who wanted to share insights   - We then discussed the discussion questions listed on the slide   - Importantly, we discussed how this activity gave students an opportunity to start practicing critical consciousness. Specifically, we asked students to identify ways the might have experienced cognitive dissonance or how they may have critically self-reflected through this activity | Workshop Organizers |
| 10 min. | *[Slide 21-23] Wrap Up*   - [21] Review takeaways from this session - [22] Preview next session. We briefly discussed here how these workshops are designed to build on one another, starting at the individual level and working up to examining societal implications for health equity, diversity, and inclusion. - [23] Final slide inviting questions or comments from students | Workshop Organizers |
