# Supplementary material for: Critical Consciousness as a Framework for Health Equity–Focused Peer Learning
Source: MedEdPORTAL. 2021 Apr 28;17:11145. doi: 10.15766/mep_2374-8265.11145 (PMC8079426; doi:10.15766/mep_2374-8265.11145)
Supplement: Supplementary file 1 — Workshop 1 Presentation.pptxWorkshop 1 Student Handout.docxWorkshop 2 Presentation.pptxWorkshop 2 Student Handout.docxWorkshop 3 Presentation.pptxWorkshop 3 Student Handout.docxWorkshop 4 Presentation.pptxWorkshop 5 Presentation.pptxFacilitator Orientation.pptxWorkshop 1 Facilitator Guide.docxWorkshop 2 Facilitator Guide.docxWorkshop 3 Facilitator Guide.docxWorkshop 4 Facilitator Guide.docxWorkshop 5 Facilitator Guide.docxEvaluation Tools.docx [file mep_2374-8265.11145-s001.zip › K. Workshop 2 Facilitator Guide.docx]

Facilitator Guide
Critical Consciousness in Medicine Workshop #2: Identity and Interpersonal Relationships

**Bolded words are specific tasks for facilitators**

Summary Table: How CCM Workshop #2 Teaches Pre-Clinical Medical Students About Diversity, Inclusion, and Health Equity

| *Overall Goal* | *Learning Objectives* | *Associated Activities* | *Anticipated Learning Outcomes* |
| --- | --- | --- | --- |
| Encourage students to reflect on their own identity and values, to understand and embrace the differences of others, and promote a climate of inclusion in medical education and health care. | Recognize the ways in which individual identity shapes person to person (interpersonal) interactions in medical education and healthcare. | Peer conversation  Case discussions | Through conversation with one another, students gain an appreciation for the diversity of their peer group.  Students apply their understanding of the concepts of identity and values to cases examples highlighting differences between doctors and patients. |
|  | Identify your own value system and reflect on your values and how they may evolve over the course of your medical education and career. | Values activity | Students gain awareness of their values, differences with the identity of their peers, and appreciate how medical school may (or may not) change their values. |
|  | Identify how a physician’s values can shape his or her decision making. | Case discussions | Students apply their understanding of the concepts of identity and values to cases examples highlighting differences between doctors and patients. |

| **WHEN** | **WHAT** | **WHO** |
| --- | --- | --- |
| 5 min. | *[Slides 1-4]: Welcome and opening remarks*   - [2] Review and re-emphasize ground rules and invite suggestions for additional ground rules - [3] Re-introduce workshop team, including facilitator and faculty roles - [4] Overview of today’s learning objectives | Workshop Organizers |
| 5 min. | *[Slides 5-9]: Intro to First Activity*   - [5] Transition slide: “To be a physician requires a transformation of the individual—one does not simply learn to be a physician, one becomes a physician.” - [6] Identity: Recap from last week   - Recall from last week’s activity that identity is shaped by both how we see ourselves and how others see us (or how we think they see us)   - These “multiple identities” may align or diverge   - We want to encourage you to critically reflect on your own identity and how that may impact your interactions with others - today we’ll be doing more of that - [7] What are values?   - Ask group what comes to mind when they think of values, then introduce definition     - Values are a person's principles or standards of behavior; one's judgment of what is important in life. - [8] Take a moment to reflect: how do our personal experiences shape our value systems?   - We’re going to examine this more closely during today’s workshop. - [9] Now we’re going to do an activity that will help you identify your own values | Workshop Organizers |
| 30 min. | *[Slides 10-14]: Values Activity*   - [10] Instructions (1 min.)   - **Distribute** the values worksheet (Appendix F) to students (one worksheet for every two students)   - **Explain** that they will complete the worksheet in pairs: partners take turns answering/discussing each question and identifying values based on their responses. **Each student should finish the activity with a list of 3-4 values that he/she identifies with.**     - Facilitators have a **“Values Word Bank”** to help students generate ideas for values based on their personal experiences.     - After pair discussion, small groups will share and discuss their responses to the activity. We will then share out and reflect as a whole room. - [11] Step 1: Pair Activity (10 min.)   - Students work in pairs to respond to prompts:     - What was a time that brought you great joy? Who were you with? What were you doing?     - What was a time where you felt called to action? What motivated you to act? What did you do?     - What are your passion areas? What do you love about them? What do they add to your life?     - What was a time you had to overcome an obstacle? How did you overcome the challenge you faced?   - From your responses, what stands out to you as being important? Use your answers to identify and articulate 3-4 personal values. Students may look at the facilitator’s **“Value Word Bank”** if they need help with this part of the idea - [11] Step 2: Small Group Discussion (9 min.)   - **Ask** students to share their responses. What values did they identify based on these experiences?     - Try to hear at least one response to each prompt.   - **Invite** critical reflection: did anything surprise you about your responses, or something you heard someone else say? - [12] Step 3: Whole Room Reflection (10 min.) – led by Workshop Organizers   - Invite sharing out from the room: would anyone like to share something that you talked about with your partner or in your small group?   - Invite critical reflection: did anything surprise you about your responses, or something you heard someone else say?   - Wrap up: remember, reflecting critically requires you to have an awareness of your own personal assumptions, biases, values, and perspectives. Keep your own values in mind as we move into case discussions.     - [13] Oath of Hippocrates: think about the values of the medical profession that just last week you pledged to uphold. How do they align with the personal values you just identified?     - [14] How can our values conflict with the values of our...classmates? Teachers? Future patients? Medical field?       - We’ll consider this more closely in the cases that we will be discussing next. | Student Facilitators |
| 25 min. | *[Slide 15-22]: Model Case Discussion: “Cross-Cultural Differences in Communication about a Dying Child”*  ***[Additional notes on this activity follow the workshop plan]***   - [16] Introduce case discussion format (1 min.)   - We will model our first case discussion through a whole room discussion   - The general format for our case discussions will be:     - Read the case (either aloud or individually)       - Invite any initial feedback/reactions     - Present and discuss different perspectives on the case     - Wrap up       - Invite final reflection on the case, as well as the activity itself       - Highlight key takeaways from the case - [17-18] Read the case (7 min.)   - We will ask someone to volunteer to read the case aloud   - Any initial feedback/reactions? (limit this to 1-2 min.) - Present and discuss different perspectives on the case (12 min. total)   - [19] Perspective A: The Physicians (6 min.)     - What is their perspective?     - Why might they feel this way?     - What values do you think they hold?     - What assumptions might they be making about the father’s values?     - How might their own identities as mothers, fathers, etc shape their decisions?   - [20] Perspective B: The Father (6 min.)     - What is his perspective?     - Why might he feel this way?     - What values does he hold?     - What assumptions might he be making about the physicians or his family?   - *If needed, read excerpts from the case perspectives to add to the discussion.* - Wrap Up (5 min.)   - [21] Invite final reflections from students (limit to 1-2 min.)   - [22] What can we take away from this case?     - “Communication with understanding is key to situations where values clash.”       - Explain condition to father - it may not be possible to hold this news from family much longer       - Consider involving a religious/spiritual leader       - Father eventually asked doctors to share prognosis with family several days later, when he believed they were in a better position to hear the news     - As students, we should not expect to be experts on every culture we may encounter. But approaching cross-cultural interactions - situations where identity and values may differ - with a spirit of humility and inquiry can help to navigate these situations.     - Cultural humility calls us as physicians to recognize our own values (and those of our profession) and consider how we may need to compromise them in order to best meet the needs of our patients.       - This doesn't imply a moral relativism where "everyone is right" but does require physicians to work to understand where patients are coming from and how we might meet them in some way. | Workshop Organizers |
| 45 min. | *[Slide 23-26] Case Discussion: “Physician Values and Clinical Decision Making”*  ***[Additional notes on this activity follow the workshop plan]***   - [23] Introduce case discussion format (1 min.)   - We will now transition into a case study that you will discuss in your small groups   - We will follow the same format, but this case will touch on some different issues     - Read the case     - Discuss different perspectives     - Wrap up - [24] Read the case (12 min.)   - Students read case individually   - **Ask if students have any initial feedback/reactions to the case (limit this to 2-3 min.)** - Present and discuss different perspectives on the case (20 min. total)   - Perspective A: The Physicians (10 min.)     - **Use the questions below to stimulate the conversation in your group, if needed.**       - What is his perspective?       - Why might he feel this way?       - What values do you think he holds?       - What assumptions might they be making about the patient?       - How might his own identity shape his interaction with Karl?       - Is he forcing his values onto Karl?   - Perspective B: Karl (10 min.)     - **Use the questions below to stimulate the conversation in your group, if needed.**       - What is his perspective?       - Why might he feel this way?       - What values do you think he holds?       - How might this interaction with the doctor affect his health?   - **Read excerpts from the case perspectives to add to the discussion, if needed.** - Wrap Up: Whole Room (12 min. total) – led by Workshop Organizers   - [25] Invite final reflections from students (8 min.)     - How did Dr. Breck’s and Karl’s values clash?     - What are the consequences of not understanding your patient?     - Invite critical reflection       - Did anything surprise you about this case, or about something you heard in your group’s discussion?       - Did this activity spur you to reflect on your own values and assumptions? If so, how?     - Lastly: how did you think this activity went? Do you have any suggestions for how we could improve something like this in the future?   - [26] What can we take away from this case? (4 min.)     - “No physician can claim to practice value-free medicine. Undoubtedly, physicians are raised with values, religious or otherwise, that shape their decisions to become professional caretakers…Nevertheless, while being aware of our own values, we must also respect those of our patients, even those with which we might disagree. Otherwise, there is a risk that our personal values may interfere with medical judgment.” *(excerpt from commentary 1)*       - Physicians should never impose their own values on a patient.     - Nurturing the doctor-patient relationship calls for attending to physical, psychological, and social (including spiritual) dimensions of care.       - “It should be obvious (though it often is not) that secular biases are as value-laden as the religious biases we attribute to Dr. Breck. The imperative to review and acknowledge the effects of our beliefs and values on patients binds all physicians—Dr. Breck and those whose biases differ from his.” *(excerpt from commentary 2)* | Student Facilitators |
| 10 min. | *[Slide 27-28] Wrap Up*   - [27] Review takeaways from this session - [28] Preview next session | Workshop Organizers |

**Practice/Model Case: *Cross-Cultural Differences in Communication About A Dying Child***

Cochran D, Saleem S, Khowaja-Punjwani S, Lantos JD. Cross cultural differences about communication in a dying child. Pediatrics. 2017;140(5):e20170690.

*Excerpts from this case are provided in Appendix F.*

Perspective A: The physicians

- What is their perspective?
- Why might they feel this way?
- What values do you think they hold?
- What assumptions might they be making about the father’s values?
- How might their own identities as mothers, fathers, etc shape their decisions?

Perspective B: The father

- What is his perspective?
- Why might he feel this way?
- What values does he hold?

What assumptions might he be making about the physicians or his family?

***Potential Questions (And How To Address Them):***

This case addresses not only a challenging and tragic diagnosis but also sensitive cultural issues and differences, including gender issues. As such, it may be a sensitive topic for students to discuss. We should expect people to be challenged working through this case, as well as be prepared to field any potentially challenging or problematic questions or statements. Such questions and statements may include (but not be limited to) the following:

- Blanket statement about Pakistani culture, especially in regards to gender roles.
- Derogatory statement about the father in this case and/or his culture and religion.
- Derogatory statement about the mother and/or women in her culture.
- Question about why the child is not taken away by child protection services/assumption that the father is being abusive.

In the event of any of these or other questions and statements being brought up, it is important to take a second to listen to what is being said before addressing the comment. If you need to take a moment to sort your thoughts and/or think of a good response that is more than acceptable. Be sure to engage the whole group in addressing any controversial statements and questions. One effective approach is to flip the question back to the student/group by asking them first what they think about it, following up with further questions to force them to think deeper about the issue. If you do not feel as though you are able to properly address a certain statement or question that is fine - the group leaders, faculty, and other facilitators are present to assist if need be.

**Small Group Case Discussion: *Physician Values and Clinical Decision Making***

Drescher J, Fergusson A. Physician values and clinical decision making. Virtual Mentor: Ethics Journal of the American Medical Association. 2006;8(5):303-308

*Excerpts from this case are provided in Appendix F.*

Perspective A: The physician

- What is his perspective?
- Why might he feel this way?
- What values do you think he holds?
- What assumptions might he be making about the patient?
- How might his own identity shape his interaction with Karl?
- Is he forcing his values onto Karl?

Perspective B: The patient (Karl)

- What is his perspective?
- Why might he feel this way?
- What values do you think he holds?
- How might this interaction with the doctor affect his health?

***Potential Questions (And How To Address Them):***

This case addresses sexual histories and STI diagnoses, which are sensitive topics to begin with, as well as discrimination against LGBTQ persons and the very relevant and controversial topics of religion in medicine and conversion therapy. As with the practice case, we should expect people to be challenged working through this case. And, we should also be prepared for any potentially problematic questions and statements such as (but, again, not limited to):

- Blanket statement about MSM sexual activity.
- Question/statement about MSM/general LGBTQ sexual practices and norms that may not be culturally sensitive or may come across as judgmental.
- Derogatory statements about the LGBTQ community.
- Derogatory statements about religion, in particular Christianity.

As noted before, in the event of any of these or other questions and statements being brought up, it is important to take a second to listen to what is being said before addressing the comment. If you need to take a moment to sort your thoughts and/or think of a good response that is more than acceptable. Be sure to engage the whole group in addressing any controversial statements and questions. One effective approach is to flip the question back to the student/group by asking them first what they think about it, following up with further questions to force them to think deeper about the issue. If you do not feel as though you are able to properly address a certain statement or question that is fine - the group leaders, faculty, and other facilitators are present to assist if need be.
