# Supplementary material for: Critical Consciousness as a Framework for Health Equity–Focused Peer Learning
Source: MedEdPORTAL. 2021 Apr 28;17:11145. doi: 10.15766/mep_2374-8265.11145 (PMC8079426; doi:10.15766/mep_2374-8265.11145)
Supplement: Supplementary file 1 — Workshop 1 Presentation.pptxWorkshop 1 Student Handout.docxWorkshop 2 Presentation.pptxWorkshop 2 Student Handout.docxWorkshop 3 Presentation.pptxWorkshop 3 Student Handout.docxWorkshop 4 Presentation.pptxWorkshop 5 Presentation.pptxFacilitator Orientation.pptxWorkshop 1 Facilitator Guide.docxWorkshop 2 Facilitator Guide.docxWorkshop 3 Facilitator Guide.docxWorkshop 4 Facilitator Guide.docxWorkshop 5 Facilitator Guide.docxEvaluation Tools.docx [file mep_2374-8265.11145-s001.zip › L. Workshop 3 Facilitator Guide.docx]

Facilitator Guide

Critical Consciousness in Medicine Workshop #3: Privilege

**Bolded words are specific tasks for facilitators**

Summary Table: How CCM Workshop #3 Teaches Pre-Clinical Medical Students About Diversity, Inclusion, and Health Equity

| *Overall Goal* | *Learning Objectives* | *Associated Activities* | *Anticipated Learning Outcomes* |
| --- | --- | --- | --- |
| Provide students with an opportunity to engage in dialogue with one another around the concepts of privilege and microaggressions, and how to restore a damaged relationship with a patient or colleague after an honest mistake. | Identify 1-3 types of privilege that we ourselves actually have. | Privilege exercise, including pre-workshop survey  Peer conversation | Through self-reflection, students identify points of privilege that they may have (or lack).  Through conversation with one another, students gain an appreciation for the diversity of their peer group. |
|  | Identify the ways that privilege interferes with colleague-colleague and doctor-patient communication and understanding. | Case discussion: “White Privilege in a White Coat”  Peer conversation: Microaggessions | Students gain understanding of how privilege may affect the care we provide to patients.  Students gain understanding of how microaggressions may impact others, in particular the perspective of marginalized individuals. |
|  | Identify a strategy to explore someone else’s perspective on privilege. | Case discussion: “My Name is Not Interpreter” | Students learn to foster a more inclusive environment by working to understand others’ perspectives and offering a clear apology when a relationship has been damaged. |

| **WHEN** | **WHAT** | **WHO** |
| --- | --- | --- |
| 10 min. | *[Slides 1-5]*   - [2] 2 min: Review ground rules - highlight a few that are important for this workshop   - Listen to hear, not to respond   - Move on   - Be fully engaged - [3] 2 min: Disclaimers - both for today and for this workshop series in general   - I want to encourage all of us to lean into uncomfortable conversations. Those moments of “stretch” can be times of immense learning.   - Some of us may feel like we “don’t need” these workshops, perhaps because of knowledge or experience that we bring into this room. I want to urge us to reflect critically on where those feelings might be coming from, and challenge each of us to “Step Up, Step Up,” both our listening and speaking - everyone in this room has a role to play here and can contribute to our collective learning.   - With these workshops we are trying to steer clear of advancing any political agenda. Though our conversations might end up overlapping with political territory, our hope is to focus on issues that impact how we work with one another as students and future health care providers, and ultimately how those issues will impact the patients we’ll be caring for one day. That’s one of the reasons why you’ll see us citing publications in our slides and why we’ll share those with you after each session so you can continue learning on your own.   - Remember, our focus here is on learning. While we might do activities in small groups, our focus isn’t necessarily team-based learning, per se. Rather, our hope is that we can learn from conversations with others, and that those conversations will spark introspection and self-reflection that will continue when we walk out the door. - [4] 2 min: Recap of what we learned last time   - Reiterate critical consciousness as a foundational principle of this workshop series   - Today is about pivoting from the personal (identities, values) to societal influences on how we relate to others - [5] 3 min: Today’s learning objectives   - Identify 1-3 types of privilege that we ourselves actually have.   - Identify the ways that privilege interferes with colleague-colleague and doctor-patient and communication and understanding.   - Identify a strategy to explore someone else’s perspective on privilege. | Workshop Organizers |
| 10 min. | *[Slides 6-8]*   - Defining privilege: Ijeoma Oluo - How we will discuss privilege - Destigmatizing the privilege discussion | Workshop Organizers |
| 5 min. | *[Slides 9-10; Optional/may need to be adapted for use with another audience/institution]*   - Pre-workshop survey results   - [9] Analogy of football field   - Top points:     - We all have privilege     - How different would the chart look in a room not full of medical students     - [10] Show certain survey results | Workshop Organizers |
| 15 min. | *[Slide 11]*   - Privilege discussion - preview whole room recap by saying “Pick someone in your group who is willing to report out on your group’s conversation. We will be calling in groups to share what they talked about.” - **[11] Small group discussion (10 min.)**   - Reflect on pre-workshop survey and results. Some discussion prompts:     - *How did you feel when you were taking the survey?*     - *What do you think about the results of the survey?*     - *What other reactions did you have to this exercise?*   - Identify types of privilege that you have. Some discussion prompts:     - *Would anyone be willing to share a privilege that you have - something you answered “yes” to in the survey?*     - *How does that privilege impact your day-to-day life?*     - *Which privilege impacts your day the most?*   - What is the cost of privilege to those that possess it?     - *Perspective - being privileged in certain areas shields you from the worries that people with less privilege have. It’s easy to say certain things do not exist or aren’t happening when you have never had to experience them.* - Whole room recap - call on groups in different part of the room to share some of the things they talked about   - Elicit different opinions by saying, “Did anyone talk about something different in their group?” | Workshop Organizers /  Student Facilitators (for small group discussion) |
| 15 min. | *[Slides 12-14]*   - [13] Case discussion: “White Privilege in a White Coat”   - 3 min to read case (1st page of article)   - **Small group discussion (10 min.)**     - Why do you think he felt that way?     - Do you agree/disagree?     - Do the comments made by the residents reflect or indicate how they may treat their patients?     - Discuss his bullet points reflecting on his privilege - [14] 2 min: privilege take homes | Student Facilitators (for small group discussion) /  Workshop Organizers |
| 5 min. | *[Slides 15-16]*   - [15] Transition to microaggressions   - [16] Definition | Workshop Organizers |
| 20 min. | *[Slides 17-18]*   - **[17] Small group discussion (12 min.)**   - *How do we know if something is considered a microaggression- as the culprit and the recipient?*   - *How are microaggressions context specific?*   - *How do you think privilege and microaggressions are connected?*   - *What is the impact of microaggressions on peer-to-peer relationships?* - [18] Whole room recap (8 min.)   - Again we can prime this by announcing that we’ll be calling on groups to share some of the highlights of their group’s conversation. | Student Facilitators (for small group discussion) / Workshop Organizers |
| 20 min. | *[Slides 19-20]*   - [19] 2 min: Introduction: now we’re going to read and discuss a case, written by Roberto Montenegro, a child psychiatrist who talks about his experience with micoaggressions. As you’re reading this case, try to put yourself in his shoes, to explore his perspective on privilege. After you’ve discussed the case in your groups, I’ll we’ll ask for a few groups to talk about how they would approach restoring a damaged relationship after an honest mistake. - **[20] Small Group Discussion (10 mins)**:   - ***Frame this discussion as: by the end of this conversation, we want to try to figure out how we might restore a damaged relationship after an honest mistake?***   - *Before we do that, let’s step back and imagine ourselves as the author of this article - as him, in his shoes.*     - *What’s it like to be him?*     - *How does it feel to go to work each day?*     - *Facilitator note: the key here is for students to imagine themselves as the author himself, not a “what would you feel like if this happened to you?” scenario.*   - *Now imagine yourself as the attending physician.*      - *How would you restore the relationship with your trainee?*   - *Could you imagine situations like this happening with colleagues - other students? Or patients? What might restoring those relationships look like?*     - ***Facilitator note: we want to work toward students (1) having an awareness of when they may have said something that could make a classmate/colleague/patient uncomfortable and (2) being able to acknowledge that and apologize for it. We’ll be talking more about this as we wrap up, but it’s great if your group moves in that direction as the conversation unfolds.***     - ***Facilitator note: Before the small group discussion ends, make sure your group has someone who is willing to answer the question “How do we restore a damaged relationship after an honest mistake?”*** - 8 min: Whole Room Recap   - Will call on 2-3 groups to offer their suggestions of how to restore a damaged relationship after an honest mistake     - Note that we are calling this an “honest mistake” because we want to assume the best of intentions - and it often is the case that microaggressions did not originate from a place of malintent. But, importantly, they have a damaging impact nonetheless. | Workshop Organizers / Student Facilitators (for small group discussion) |
| 10 min. | *[Slides 21-22]*   - [21] Ask for student volunteer to read Montenegro perspective on restoring relationships   - Here organizers may wish to offer a personal perspective on the topics discussed this far, especially as they relate to restoring relationships after a harm - [22] Highlights from Gonzalez research   - Perceived bias or discrimination is shaped by the patient’s lived experience - we often don’t know the particulars of this experience   - How the physician responds to a moment of perceived bias is key - acknowledging the patient’s perception improves trust and engagement, while ignoring it damages trust and can lead to avoidance of medical care   - Interestingly, most patients just wanted the incident to be acknowledged and were satisfied with an apology from the physician   - Of course, this requires us to be aware of when something has gone wrong in our interactions with colleagues and patients - this can be hard to do! - Questions/conversation? | Workshop Organizers |
| 10 min. | *[Slides 23-24]*   - [23] Take homes and final thoughts - [24] What to look forward to | Workshop Organizers |
