# Supplementary material for: Critical Consciousness as a Framework for Health Equity–Focused Peer Learning
Source: MedEdPORTAL. 2021 Apr 28;17:11145. doi: 10.15766/mep_2374-8265.11145 (PMC8079426; doi:10.15766/mep_2374-8265.11145)
Supplement: Supplementary file 1 — Workshop 1 Presentation.pptxWorkshop 1 Student Handout.docxWorkshop 2 Presentation.pptxWorkshop 2 Student Handout.docxWorkshop 3 Presentation.pptxWorkshop 3 Student Handout.docxWorkshop 4 Presentation.pptxWorkshop 5 Presentation.pptxFacilitator Orientation.pptxWorkshop 1 Facilitator Guide.docxWorkshop 2 Facilitator Guide.docxWorkshop 3 Facilitator Guide.docxWorkshop 4 Facilitator Guide.docxWorkshop 5 Facilitator Guide.docxEvaluation Tools.docx [file mep_2374-8265.11145-s001.zip › M. Workshop 4 Facilitator Guide.docx]

Facilitator Guide

Critical Consciousness in Medicine Workshop #4: Health Disparities

**NB: Due to a scheduling conflict, student facilitators were unavailable for this session. Instead, groups were “self-facilitated” by students at each table, with assistance from workshop organizers circulating the room. Therefore, there are no bolded facilitator instructions in this guide.**

Summary Table: How CCM Workshop #4 Teaches Pre-Clinical Medical Students About Diversity, Inclusion, and Health Equity

| *Overall Goal* | *Learning Objectives* | *Associated Activities* | *Anticipated Learning Outcomes* |
| --- | --- | --- | --- |
| Discuss the historical and structural factors that contribute to health disparities and inspire students to work on addressing health disparities as part of their career in medicine. | Identify two examples of health disparities in U.S. society. | Health disparities overview presentation  Peer conversation/ Large group discussions | Students are able to define key terms including health disparities, social determinants of health, and health equity.  Students identify asthma and maternal mortality as examples of health disparities in the U.S. |
|  | Analyze the historical context and structural factors that contribute to present-day health disparities. | Peer conversation/ Data discussion | Students become familiar with data related to health disparities (specifically asthma and maternal mortality).  Students identify factors within the health system and outside of the health system that contribute to health disparities. |
|  | Recognize the opportunity that medical students and physicians have to work on addressing health disparities. | Self-reflection | Students generate “action items” for physicians interested in working on health disparities and cultivate their own sense of social responsibility as future physicians. |

*Note: We used Turning Point technology to facilitate student engagement throughout this session; this “clicker” technology is accessed via students’ phone or computer. This session was also designed to involve the use of online resources and reflection forms. As a result, students were encouraged to bring their laptop to the workshop.*

| **WHEN** | **WHAT** | **WHO** |
| --- | --- | --- |
| 5 min. | Welcome/Recap [Slides 1-4]   - [2] Ground rules recap (2 min.)   - Emphasize importance of upholding these in the absence of facilitators. Today’s conversation will look differently from previous ones, but these rules still apply.   - Full engagement: we want to hear from all parts of the room and different perspectives. - [3] Learning objectives (1 min.) - [4] Our approach today (2 min.)   - Logistics     - Everyone should have a computer, and every group should have one person (and only one person) signed into TurningPoint. Do that now if you haven’t already.     - We’ll be asking you to use your computers at various points in today’s session but if you aren’t using it for something related to our conversation, please close your computer so we can ensure full participation - this will also help us know when we’re ready to move on to   - Overall aims/approach for the day     - In our first two workshops, we talked about our own identities and how privilege shapes the ways that we relate to others, especially people from different backgrounds, and how those factors impact the care we provide as clinicians.     - Today, we’re going to expand our scope to examine how broader social factors have an impact on health outcomes. We’re going to do that through two case discussions that we hope will connect to some of the issues we talked about in the first half of the year and illustrate how health disparities manifest themselves. So let’s begin! | Workshop Organizer |
| 10 min. | Health Disparities Overview [Slides 5-11] (10 min.)   - [5] Virchow was a 19th century physician who made major contributions to the fields of cell biology and pathology. He was also responsible for some of the major public health advancements during this time. As Virchow shows us, there is a long history of physicians considering the ways that social factors impact their patients’ health - that is where we’ll turn our attention today. - [6] Health disparities definition   - Note that these differences are preventable and are experienced by socially disadvantaged populations.     - Note that health disparities typically do not refer to differences that are due to a biological cause - e.g. higher rates of breast cancer among women than men (though we could examine disparities in breast cancer outcomes in women).   - These differences don’t exist due to happenstance or coincidence - they are historically produced! Today, we hope we can examine some of the historical context behind health disparities and hope that you can do the same in your work going forward. - [7] Examples of health disparities   - Note that these examples are cross-cutting - across types of health condition (e.g. cancer, chronic disease, mental health, accidents/injuries) and across the populations affected (i.e. health disparities are not confined to a single race - again, they disproportionately impact socially disadvantaged populations) - Social determinants of health   - [8] Definition: we want to introduce you to this important concept, which is a driver of many health disparities   - [9] Examples of SDOH     - Some of these (access to health care, health literacy) are more proximal to the health care system, but all of these factors impact people’s health outcomes   - [10] SDOH graphic     - Shows how health society, environment, and community are the foundation for health people     - Much more than health care goes into determining whether or not people are healthy   - [11] Health equity = everyone has the highest level of health     - This the goal of efforts to address health disparities | Workshop Organizer |
| 45 min. | Case Discussion: Asthma [Slides 12-30]   - [13] Outline (1 min.)   - Here’s our plan for this conversation. (TP) means there is some TurningPoint participation involved, either as a clicker question or word cloud, so make sure your group has someone logged in. We will be looking for 32 responses (one from each group) - [14] What is your diagnosis? (2 min.)   - History of respiratory problems relieved by steroids is highly suggestive of asthma. If this wasn’t clear to you, that ok - we are using this diagnosis as a jumping off point for our conversation - Data: Asthma as a Health Disparity   - [15] Discuss these questions in your group and be ready to share out the results of your conversation (3 min.)   - [16] Report out + highlight data (5 min.)     - Emphasize that there are differences in terms of both incidence and outcomes - minority children are more likely to have asthma *and* are more likely to have asthma exacerbations - Health Systems Contributors   - [17] Discuss in your group and submit via TP (2 min.)   - [18] Review TP responses (1 min.)   - [19] Review health systems contributors (3 min.) - Social determinants - as we’ve talked about, many factors contribute to health disparities   - [20] Discuss in your group and submit via TP (2 min.)   - [21] Review TP responses (1 min.)   - [22] Review social determinants (3 min.) - [23-24] Historical context (5 min.)   - On the last slide, we saw that housing is a social determinant of health that contributes to asthma disparities. Let’s look at some of the historical processes that shape asthma as a health disparity   - Emphasize how local and federal housing policies set tone for housing markets, which in turn dictated allocation of and access to resources (e.g. where are landfills/industry located, vs. where businesses and services are located)   - The historical redlining map correlates closely with present-day health disparities - [25] Reflection (7 min.)   - Go to RWJF website and type in your address (or any address you want).   - Look at the data, then go to the reflection site. We are not going to discuss these reflections as a group and they will not be shared, so we encourage you to write candidly and thoughtfully.   - Reflection questions:     - Is life expectancy in your area better than, worse than, or about the same as life expectancy in New Orleans as a whole?     - What do you think accounts for life expectancy in your area? Is it different from the life expectancy in New Orleans as a whole? If so, why?     - What is your reaction to these data about life expectancy? Is this information important for health care providers? Why or why not? - What can we do about it? - *may eliminate TP response element based on time*   - [26] Discuss in your group and submit via TP (2 min.)   - [27] Review TP responses (1 min.)   - [28-29] Review opportunities for action (3 min.)     - It should be clear now that a complex web of factors. No single action is going to be the solution, but there are opportunities to address health disparities at a number of different levels.   - [30] Clinician perspective (5 min.) | Workshop Organizer |
| 45 min. | Case Discussion: Maternal Mortality [Slides 31-43]   - See accompanying slides in Appendix J for details on this portion of the workshop | Workshop Organizer |
| 15 min. | Reflection and Wrap Up [44-47]   - [44-45] Data disclaimer → top points   - Reiterate importance of considering historical processes when examining health disparities   - Opportunities for action exist at multiple levels   - The topics we discussed in our first two workshops are connected to health disparities - identity and interpersonal relationships can worsen disparities or improve the situation. - [46] What to look forward to - [47] Closing reflection (via Google form)   - What is one thing that you learned in today’s workshop?   - What is one thing that you want to learn more about following today’s workshop?   - What is one thing that frustrated you about the topics we discussed in today’s workshop? How do you hope to deal with that frustration? | Workshop Organizers |
