# Supplementary material for: Critical Consciousness as a Framework for Health Equity–Focused Peer Learning
Source: MedEdPORTAL. 2021 Apr 28;17:11145. doi: 10.15766/mep_2374-8265.11145 (PMC8079426; doi:10.15766/mep_2374-8265.11145)
Supplement: Supplementary file 1 — Workshop 1 Presentation.pptxWorkshop 1 Student Handout.docxWorkshop 2 Presentation.pptxWorkshop 2 Student Handout.docxWorkshop 3 Presentation.pptxWorkshop 3 Student Handout.docxWorkshop 4 Presentation.pptxWorkshop 5 Presentation.pptxFacilitator Orientation.pptxWorkshop 1 Facilitator Guide.docxWorkshop 2 Facilitator Guide.docxWorkshop 3 Facilitator Guide.docxWorkshop 4 Facilitator Guide.docxWorkshop 5 Facilitator Guide.docxEvaluation Tools.docx [file mep_2374-8265.11145-s001.zip › N. Workshop 5 Facilitator Guide.docx]

Facilitator Guide

Critical Consciousness in Medicine Workshop #5: Implicit Bias

*Special instructions for facilitators highlighted – for this session we invited our incoming CCM leadership to join workshop organizers in facilitating the session.*

Summary Table: How CCM Workshop #5 Teaches Pre-Clinical Medical Students About Diversity, Inclusion, and Health Equity

| *Overall Goal* | *Learning Objectives* | *Associated Activities* | *Anticipated Learning Outcomes* |
| --- | --- | --- | --- |
| Discuss implicit bias and ways to address bias.  Synthesize and recap learning from all five CCM workshops. | Discuss the Implicit Association Test (IAT) as a tool for surfacing implicit bias. | IAT and post-IAT survey (completed prior to workshop)  Peer discussion | Students reflect on the experience of having completed the IAT and discuss its utility in helping to surface implicit bias, as well as its limitations. |
|  | Identify instances of implicit bias in medical education and health care. | Tag Game – introductory activity  Peer discussion and “vote with your feet” activity | Students are able to define key terms such as implicit bias and ingroup favoritism and consider ways in which these phenomena may manifest themselves in clinical scenarios. |
|  | Apply an understanding of implicit bias to reflect on ways to address our own biases. | Workshop presentation  Peer discussion | By better understanding the concept of implicit bias, students are able to deconstruct their own biases and identify ways that health care providers can reduce implicit bias. |
|  | Synthesize learning from across CCM workshops and develop a plan for future learning. | Workshop presentation  Self-reflection | Students review key concepts from the workshop series, draw connections between workshop sessions, and identify individually salient points of growth and learning. |

*Notes:*

- *We used Turning Point technology to facilitate student engagement at several points in this session; this “clicker” technology is accessed via students’ phone or computer. This session was also designed to involve the use of online reflection forms. As a result, students were encouraged to bring their laptop to the workshop.*
- *Prior to this workshop, students received a didactic lecture on implicit bias from one of our faculty advisors and completed the Implicit Association Test (IAT), available at implicit.harvard.edu*

| **WHEN** | **WHAT** | **WHO** |
| --- | --- | --- |
| 20 min. | Welcome/Intro [Slides 1-5]   - [2] Tag Game (10 min.)   - Set timer when we start instructions   - Introduce game, explain that we are being purposefully vague as part of the exercise.   *Facilitators:*   - *Stickers will be laid out on the tables in advance, make sure everyone gets one.* - *Do some “crowd control” to help keep people in their quadrant of the room.* - *(Kindly) remind students to form their groups* without *talking.* - *Keep an eye on how groups are forming so you can let us know when we’re ready to move on. You may also make some observations that are helpful in the debrief.* - *If issues come up, let me or Jonte know.* - [3] Ground rules reminder (3 min.)   - Emphasize importance of full engagement - phones away, laptops closed   - Introduce *facilitators* and explain their role: they will be organizing next year’s workshops and will be helping us with today’s workshop by periodically checking in with your groups - [4] Learning objectives (2 min.)   - The first part of today’s workshop will give you a chance to debrief and share reactions to the IAT along with your classmates.   - We will then discuss some ways that implicit bias plays out in medical education and health care and spend some time reflecting on our own biases   - Lastly, since this is our last session of the year, we are going to try to recap what we’ve talked about over the course of these workshops and synthesize all that we’ve learned together. - [5] Disclaimers (5 min.)   - The creator of the IAT believes that results of the test should be like your medical records or who you voted for - you shouldn’t have to disclose your results unless you want to. We will treat things the same way today: we will spend some time allowing you to process the experience of taking the IAT, but you do not have to talk about the results of your test, unless you want to.   - Some notes about the IAT itself:     - Our purpose today is to use your experience taking the test as a jumping off point for a broader conversation about implicit bias     - The test is a tool to aid with self reflection, it is not a diagnostic. The test can’t tell you if you are or aren’t a bigot and that’s not what we’re here to do, either.     - Our hope is to help surface our implicit attitudes to make them more accessible to our awareness, not to try to get a “better” score on the test     - If you have more questions about the test itself how it was developed, how to interpret results, etc. - I encourage you to check out the Project Implicit FAQs, as this will not be the focus of our conversation today | Workshop Organizers |
| 15 min. | IAT Debrief [Slides 6-8]   - [6] Post IAT Survey Results (1 min.) - [7] Survey quotes/common themes (1 min.)   - Reiterate disclaimers: IAT is not a diagnostic tool, nor is to be used to label anyone. Our goal is to expose students to an important component of the growing discourse about implicit bias in medicine and to use the IAT as a tool to help inform our learning today about implicit bias - [8] Discuss IAT and Survey   - Small group (8 min.)   - Whole room (5 min.)   *Facilitators: The focus here is to keep the conversation centered on the* experience *of the IAT, not the test itself (e.g. fixating on the validity of the test).*   - *Encourage students to focus on* ***“What can I learn from the IAT?”*** *not* ***“What can I learn about the IAT?”*** - *If students start talking about the test itself that’s ok, just try to redirect the conversation by reminding students that we aren’t focusing our conversation on the test itself and posing one of the discussion questions to the group.* - *You may get asked questions about the IAT. You should familiarize yourself with the FAQs and can answer questions if you feel comfortable doing so. But if you don’t know the answer, say that and encourage students to learn more about the test* after *the workshop is over.* - *We really want to encourage students to explore any* ***cognitive dissonance*** *they experienced as a result of the IAT. Probe to see if anyone experienced something that surprised them or challenged them.* - *Remind the group that they should be prepared to share out in the whole room conversation, and encourage sharing from voices we haven’t heard from a great deal over the course of the year.* | Workshop Organizer |
| 5 min. | What? Why? How? [Slides 9-11]   - [9] Transition slide (1 min.)   - You have already had a lecture on what implicit bias is. Today we will recap the definition, explain why we have implicit bias as well as why its important that physicians are aware of implicit bias. And we will discuss how we can try to combat our implicit bias. - [10] Implicit bias defined (2 min.) | Workshop Organizer |
| 25 min. | Ingroup Favoritism and Implicit Bias [Slides 12-16]   - [11] Tag Game: Debrief (5 min.)   - Explain:     - This was an exercise to get us thinking about social categorization and ingroup bias.     - There is an automaticity to “us” vs “them” categorizations - we are primed to have a greater affinity for people who are like us, even if it’s in an arbitrary category like stickers.     - Also note that very few groups formed intentionally diverse groups in which many shapes, colors, and sizes were represented. Promoting diversity often requires intentional effort, in part because our natural tendency runs counter to this.     - Some participants might express frustration with the limitations of the exercise, and that’s ok! Where is that frustration coming from?       - Some students might feel that this was an arbitrary exercise - of course they would group themselves based on stickers when we handed them stickers. But how different are the stickers in this exercise from the traits that carry implicit associations in real life? E.g. sex (or outward appearance of gender), skin color, etc. - [12] Ingroup bias defined (2 min.)   - We are conditioned early: for example, babies start to show a preference for faces of their own race at three months of age   - We’ve seen some examples of ingroup favoritism, but how could this play out in health care? - [13] Case discussion: “Carl”   - Read the case, then turn and talk with a partner (or a group of three) at your table. Discuss: (4 min.)     - What happened here? Was implicit bias at play?   *Facilitators: Reinforce these discussion questions, help let us know when groups are wrapping up discussion.*   - - Summary (not whole room discussion) (1 min.)     - One way to interpret this case is as a case of subtle discrimination. What do I mean by that?     - We have both “Carl the fisherman” and “Carl the LSU professor”     - Carl-as-professor triggered an in-group bias     - Carl-the-fisherman suddenly became a fellow member of the LSU community and qualified for elite care     - The discrimination here is the difference between okay and elite care.     - In-group favoritism can increase the relative advantages of those who are already advantaged     - Note that we are particularly prone to bias in situations of high stress - e.g. an emergency room! - [14] What ingroups are you a part of? (3 min.)   - Let’s pause here and do some self-reflection: what ingroups are you a part of?   - Turn and talk with a partner (or group of three) at your table, and then type in some of the ingroups you’ve each identified in the TurningPoint word cloud   *Facilitators: Some students may have a hard time identifying an ingroup. Ingroup = a group with a shared interest or identity. It may help if you can share an example or two.*   - [15] Would your ingroups bias the care you provide? (10 min.)   - Vote with your feet!     - If you feel that “Yes - ingroup bias could impact the care I provide” - stand at the edge of the table nearest the center of the room     - If you feel that “No - ingroup bias would not impact the care I provide” - stand at the edge of the table nearest the outside of the room.     - If you’re not sure or feel somewhat less strongly - stand in the middle of the table, shaded toward the side you believe   *Facilitators: We may need your help directing traffic/explaining the “vote with your feet” mechanism.*   - - Invite students to share why they are standing where they are. Try to hear from at least one “yes,” one “no,” and one in the middle.   - To wrap up this section: belonging to an ingroup doesn’t guarantee bias. But, as we’ve seen from the Tag Game exercise and the case with Carl, ingroup favoritism does predispose to have an affinity for those who are like us, and it can (but doesn’t always) lead to bias against those who are different. | Workshop Organizer |
| 25 min. | Deconstructing Implicit Bias: How our Environment and Experiences Contribute [16-23]   - [16] Deconstructing implicit bias (2 min.)   - We have a natural tendency to group things around us--ingroups are an example of this   - Our environments teach us us how exactly to group - [17] What do these ads, news articles, and movie posters teach us?   - Discuss with your partner (or group of three) (5 min.)   - Whole room sharing (2 min.)   *Notes for facilitators:*   - [18] Implicit bias in medical education   - In some ways, our training enforces/reinforces biases.   - Board questions in particular rely on students making associations to help make diagnoses   - There is a tension here: how do we take this legitimate information about risk for disease into account without biasing our interactions with patients?   - Discuss with your table (5 min.)   *Facilitators: Many of the examples we shared are from second year content; it might help students if you can identify examples of risk factors/predisposing conditions that you have learned about in the first year that could play into bias.*   - *You might encourage students to think about a patient they have seen (e.g. during preceptorship). Did those patients “conform” with the stereotype of their disease? Knowing what they know now, would the students approach the patient differently?*   - Whole room sharing (3 min.)     - Summary: we always have to take patients’ circumstances into account - including risk factors and predisposing conditions. But we can’t rely only on associations to guide our diagnosis and treatment - we have to understand their individual circumstances. This is an example of **individuation**, one of the techniques for combating implicit bias - we will learn more about this in a bit. - [19] Deconstructing implicit bias (2 min.)   - Reiterate points from slide [16]     - Through the Tag Game, we saw how we have a natural tendency to group things and affiliate with others who are like us     - Looking at media and advertising, we saw how our environment teaches us *how* to group things     - We learn through association, and in medicine especially, these associations can serve as shortcuts - we are explicitly taught some of these associations in medical school - [20] Beyond the preclinical years, studies suggest that students’ experiences with patients and physicians during clerkships can contribute to implicit bias (2 min.)   - Raising this point here because in CCM we have talked about medical school as a time of transformation. Think about how you will change - perhaps in some ways that a worth resisting   - So, how can we counteract implicit bias? - [21] How to Fight Our Own Implicit Bias (4 min.)   - This slide summarizes some of the key points from an Institute for Healthcare Improvement article on how providers can reduce implicit bias | Workshop Organizers |
| 30 min. | Reflection, Recap, and Wrapping Up [22-27]   - [22] Workshop 1 Recap (1 min.)   - Let’s take a step back to briefly recap everything we’ve learned over the course of this year   - The title of this workshop series is “Critical Consciousness in Medicine” because that’s one thing we hope to impart through these workshops: an awareness of self, others, and the world, and a commitment to addressing issues of societal relevance in health care     - Obviously this isn’t something that we can accomplish through one series of workshops, but we hope to have contributed to the process of your developing critical consciousness as a medical student and future physician - [23] Workshop 2 Recap (1 min.)   - In this workshop, we looked at how identity and values shape the interactions we have with others   - We discussed two cases where identity and values impacted care   - Something for you to ponder: how have your identity and values changed since the beginning of the year? - [24] Workshop 3 Recap (1 min.)   - In this workshop, we expanded our focus to talk about privilege.   - Remember, privilege is an advantage or set of advantages that you have that others do not. This doesn’t mean that we haven’t worked hard or earned what we have. It means that we have to understand how people’s experiences differ based on factors that we don’t personally control.   - In this session, we also learned about microaggressions - the indirect and unintentional ways that privilege can manifest itself in our day-to-day interactions with one another   - And we talked about how we can make it right after an honest mistake     - Remember - after an incident of perceived bias, most patients just want an acknowledgment and a meaningful apology. This goes a long way toward maintaining the trust and engagement that are key to a strong doctor-patient relationship   - Something for you to ponder after today’s workshop: what is the relationship between privilege and implicit bias? - [25] Workshop 4 Recap (1 min.)   - In this workshop, we broadened the discussion to encompass community and societal issues: specifically, health disparities   - Health disparities are unnatural, preventable differences in health outcomes that often have underlying historical discrimination and inequality underneath them   - We looked at two examples of health disparities - asthma and maternal mortality - and how historical factors and the social determinants of health contribute to unjust outcomes   - Thinking back to the first two workshops, something for you to ponder: how do our values and relationships contribute to or counteract health disparities? - [26] Workshop 5 Recap   - Today we talked about implicit bias, a topic that bridges many of the domains we covered in this workshop series, from societal to individual   - There is a growing body of research examining implicit bias in health care, including how bias may contribute to health disparities   - There is also a growing field looking at how we can counteract implicit bias - we learned about some of those strategies today - [27] Wrapping up   - To close out, we have a reflection form and a year-end survey. You can find links to both of these in an email that has been sent to you   - Once you’ve filled out the reflection form, we encourage you to send this as an email to yourself so that you can check in with yourself in the future.     - Instructions for how to do that are here and we have listed some dates that you might consider sending the email   - In the interest of transparency, it’s important to say that we will be able to see responses in the Google forms but all responses will be anonymous, so we encourage you to be open and honest with your reflections. | Workshop Organizers |
