# Supplementary material for: Critical Consciousness as a Framework for Health Equity–Focused Peer Learning
Source: MedEdPORTAL. 2021 Apr 28;17:11145. doi: 10.15766/mep_2374-8265.11145 (PMC8079426; doi:10.15766/mep_2374-8265.11145)
Supplement: Supplementary file 1 — Workshop 1 Presentation.pptxWorkshop 1 Student Handout.docxWorkshop 2 Presentation.pptxWorkshop 2 Student Handout.docxWorkshop 3 Presentation.pptxWorkshop 3 Student Handout.docxWorkshop 4 Presentation.pptxWorkshop 5 Presentation.pptxFacilitator Orientation.pptxWorkshop 1 Facilitator Guide.docxWorkshop 2 Facilitator Guide.docxWorkshop 3 Facilitator Guide.docxWorkshop 4 Facilitator Guide.docxWorkshop 5 Facilitator Guide.docxEvaluation Tools.docx [file mep_2374-8265.11145-s001.zip › O. Evaluation Tools.docx]

**Evaluation Tools
Critical Consciousness in Medicine Workshop Series**

This document outlines the various evaluation tools – including surveys and online reflection forms – that we used to assess student learning and evaluate the project as a whole.

Post-Workshop Feedback
*We used this survey to invite student feedback after the completion of each workshop. The survey was emailed to students following workshops and was optional.*

1. Please rate the performance of the student workshop organizers on a scale of 1-5 (1=Poor, 5=Excellent). Please include any additional comments you would like to share.
2. Please rate the performance of your small group facilitator on a scale of 1-5 (1=Poor, 5=Excellent). Please include any additional comments you would like to share.
3. Please rate the [learning activity] on a scale of 1-5 (1=Poor, 5=Excellent). Please include any additional comments you would like to share.
   *Note: we asked for separate ratings for each different learning activity (e.g. self-reflection, peer conversation, case discussion, etc.) in every workshop.*
4. Please rate the overall content of the workshop on a scale of 1-5 (1=Poor, 5=Excellent. Please include any additional comments you would like to share.

Privilege Self-Assessment (Pre-Survey for Workshop #3)
*This survey was administered to all students prior to CCM Workshop #3, which focuses on privilege. The survey is meant to encourage students to reflect on individual points of privilege that they may have. We shared data from this survey with students to encourage further reflection and conversation with peers during the workshop.*

*Source:* Survey prompts adapted from Holm AL, Gorosh MR, Brady M, White-Perkins, D. Recognizing privilege and bias: An interactive exercise to expand health care providers’ personal awareness. *Academic Medicine*. 2017;92(3)360-364.

1. I can move about in public without fear of being harassed or physically attacked because of my sexual/gender identity.
   - Yes
   - No
2. I can assume that I will easily have physical access to any building.
   - Yes
   - No
3. I am never asked to speak for all the people of my racial/gender/other identifying group.
   - Yes
   - No
4. I never worry about being recognized as the sex/gender with which I identify.
   - Yes
   - No
5. I am less likely to be sexually harassed at work than persons of other gender identities.
   - Yes
   - No
6. I do not have to think about the message my wardrobe sends about my sexual availability.
   - Yes
   - No
7. In general, I am not under much pressure to be thin or to worry about how people will respond to me if I’m overweight.
   - Yes
   - No
8. I can do well in a challenging situation without being called a credit to my race/gender/other identifying group.
   - Yes
   - No
9. If I have children and a successful career, few people will ask me how I balance work and home.
   - Yes
   - No
10. I feel safe walking by myself at night.
    - Yes
    - No
11. My parents went to college.
    - Yes
    - No
12. My parents went to college.
    - Yes
    - No
13. When I speak in a room full of my peers people listen.
    - Yes
    - No
14. I feel safe when I encounter law enforcement.
    - Yes
    - No
15. I can be sure that mainstream media will celebrate the holidays of my religion.
    - Yes
    - No
16. I do not need to worry about negative consequences of disclosing my religious identity to others.
    - Yes
    - No
17. I can be sure that my social class will be an advantage when I seek medical or legal help.
    - Yes
    - No
18. I don’t have to rely on public transportation; I can afford my own vehicle
    - Yes
    - No
19. I have a savings account with at least a month’s expenses in case of emergency.
    - Yes
    - No
20. I am in medical school
    - Yes
    - No
21. If I were a victim of a crime, I wouldn’t think twice about seeking police assistance due to my citizenship status.
    - Yes
    - No
22. If I wanted to, I could travel freely to almost any country and be admitted back into the U.S.
    - Yes
    - No

Health Disparities Reflection (Optional Activity for Workshop #4)
*These questions are part of the optional reflection activity outlined on Slide 25 of Workshop #4. We created this as an opportunity for student self-reflection in the midst of the workshop and as a means to qualitatively assess student learning related to health disparities.*

After visiting the Robert Wood Johnson Foundation's life expectancy gap tool (https://goo.gl/6KyGTa), submit a reflection on your results. Reflections are anonymous and will not be shared.

1. Is life expectancy in your area better than, worse than, or about the same as life expectancy in New Orleans as a whole?
   - Better
   - Worse
   - About the same
2. What do you think accounts for life expectancy in your area? Is it different from the life expectancy in New Orleans as a whole? If so, why? *(free text response)*
3. What is your reaction to these data about life expectancy? Is this information important for health care providers? Why or why not? *(free text response)*

Closing Reflection (Optional for Workshop #4)
*These questions are part of the optional reflection activity outlined on Slide 47 of Workshop #4. We administered these questions to all students immediately following the workshop to create an opportunity for student self-reflection after the workshop and to qualitatively assess student learning related to health disparities.*

1. What is one thing that you learned in today's workshop? *(free text response)*
2. What is one thing that you want to learn more about following today's workshop? *(free text response)*
3. What is one thing that frustrated you about the topics we discussed today? How do you hope to deal with that frustration? *(free text response)*

Post-IAT Survey (Pre-Workshop #5 Activity)
*Prior to Workshop #5, students were asked to complete the Implicit Association Test (IAT). We administered these questions to students immediately after completing the IAT to allow students to debrief and reflect on the experience and to anticipate student reactions/concerns to the IAT that might come up during the workshop. We presented some of the data from this survey in our workshop.*

1. Which IATs did you take?
   - Asian
   - Gender-Career
   - Race
   - Presidents
   - Skin Tone
   - Age
   - Native
   - Religion
   - Gender Science
   - Weight
   - Arab/Muslim
   - Sexuality
   - Weapons
   - Disability
2. What did you expect your results to be? Were you surprised? *(free text response)*
3. Did the IAT change how you think about implicit bias?
   - Yes
   - No
4. Explain your answer to Question 3. *(free text response)*
5. Do you disagree with your results?
   - Yes
   - No
   - Maybe
6. Explain your answer to Question 5. *(free text response)*
7. Any additional thoughts or feelings? *(free text response)*

Year-End Reflection (Optional Activity for Workshop #5)
*These questions are part of the optional reflection outlined on Slide 27 of Workshop 5. These activity was developed to provide students with an opportunity for self-reflection at the conclusion of the workshop series and to qualitatively assess student learning.*

1. Melanie Funchess talks about the need to do our own "personal work." What is your personal work? In order to build a world with equity, what aspects of yourself do you want to change? What topics that we have discussed this year do you want to learn more about? What new skills do you want to develop?
2. In the future, how will you know if you've done that work? What will you look like as a critically conscious medical provider?

Year-End Survey
*We administered this survey to all students at the conclusion of Workshop 5 to quantitatively assess student learning and growth over the course of the year. We also included a free text response to allow for qualitative assessment. You may consider structuring this as a true pre/post survey, with students rating themselves once before the first workshop and again after the final workshop.*

This survey asks you to rate yourself on a scale of 1-10, comparing the beginning of the year (August) to now.

1. Rate your ability to define critical consciousness (1=worst, 10=best).
   - At the start of the school year (1-10)
   - Now (1-10)
2. Rate your ability to identify your own value system (1=worst, 10=best).
   - At the start of the school year (1-10)
   - Now (1-10)
3. Rate your ability to identify how a physician's values can shape his or her decision making (1=worst, 10=best).
   - At the start of the school year (1-10)
   - Now (1-10)
4. Rate your ability to define privilege (1=worst, 10=best).
   - At the start of the school year (1-10)
   - Now (1-10)
5. Rate your ability to explore someone else's perspective on privilege (1=worst, 10=best).
   - At the start of the school year (1-10)
   - Now (1-10)
6. Rate your ability to define health disparities (1=worst, 10=best).
   - At the start of the school year (1-10)
   - Now (1-10)
7. Rate your motivation to work on addressing health disparities as a part of your career in medicine (1=worst, 10=best).
   - At the start of the school year (1-10)
   - Now (1-10)
8. Rate your ability to define implicit bias (1=worst, 10=best).
   - At the start of the school year (1-10)
   - Now (1-10)
9. Rate your ability to identify and address your own biases (1=worst, 10=best).
   - At the start of the school year (1-10)
   - Now (1-10)
10. How have CCM workshops contributed to your growth over the course of this school year? *(free text response)*
