# Supplementary material for: Critical Consciousness as a Framework for Health Equity–Focused Peer Learning
Source: MedEdPORTAL. 2021 Apr 28;17:11145. doi: 10.15766/mep_2374-8265.11145 (PMC8079426; doi:10.15766/mep_2374-8265.11145)
Supplement: Supplementary file 1 — Workshop 1 Presentation.pptxWorkshop 1 Student Handout.docxWorkshop 2 Presentation.pptxWorkshop 2 Student Handout.docxWorkshop 3 Presentation.pptxWorkshop 3 Student Handout.docxWorkshop 4 Presentation.pptxWorkshop 5 Presentation.pptxFacilitator Orientation.pptxWorkshop 1 Facilitator Guide.docxWorkshop 2 Facilitator Guide.docxWorkshop 3 Facilitator Guide.docxWorkshop 4 Facilitator Guide.docxWorkshop 5 Facilitator Guide.docxEvaluation Tools.docx [file mep_2374-8265.11145-s001.zip › Educational_Summary_Report_11145.pdf]

MedEdPORTAL® | The AAMC Journal of Teaching and Learning Resources

Original Publication

Open Access

Critical Consciousness as a Framework for Health Equity–Focused Peer Learning

Jonte Ellison, Chris Gunther, MPH*, Mary Beth Campbell, MSPH, Robin English, MD, Cathy Lazarus, MD

*Corresponding author: chrisjgunther@gmail.com

Abstract

Introduction: Recognizing the need to teach concepts of health equity, diversity, and inclusion as a part of medical students' preclinical training, we developed a series of workshops in the first year of medical school that introduced students to issues of discrimination and inequity and their effects on health outcomes. This student-led, faculty-supported project, known as Critical Consciousness in Medicine (CCM), adopted critical consciousness as a guiding principle for student learning. **Methods:** Over the course of the 2018-2019 academic year, student leaders developed and delivered five 2-hour workshops to 197 first-year students, with the assistance of student facilitators and input and guidance from faculty advisors. Workshops involved a mix of whole-class presentations and small-group discussions. Session topics included identity and interpersonal relationships, privilege, health disparities, and implicit bias. **Results:** Paired *t*-test analysis showed statistically significant growth in student self-ratings related to CCM learning objectives as measured in the end-of-year pre-/postsurvey. Student comments in year-end reflections further suggested learning, self-assessment, growth, and appreciation for the workshops' place in the preclinical curriculum. **Discussion:** This project modeled a student-faculty partnership for approaching diversity, inclusion, and health equity in medical education and highlighted the role of students as leaders in educating their peers. The CCM workshop series demonstrated high acceptability as a component of preclinical medical education and may increase student engagement around social issues in health care. CCM also illustrated the promise of using critical consciousness as an approach to educating medical students about equity, diversity, and inclusion.

Keywords
Critical Consciousness, Diversity & Inclusion, Student Leadership, Health Disparities, Implicit Bias, Diversity, Inclusion, Health Equity

Educational Objectives

By the end of this five-workshop series, learners will be able to:

1. Define critical consciousness and apply the concept to understanding the lived experiences of peers and patients.
2. Explore their own perspective, including individual identity and values, and identify how it impacts interactions in the clinical arena.
3. Explore the concept of privilege, particularly in the context of impact on and perceptions by marginalized individuals and communities.
4. Increase, through case examples and peer discussion, students' motivation to work on addressing health disparities as part of their career in medicine.
5. Identify and practice ways to address their own implicit biases, particularly those related to medical education and patient care.

Citation:
Ellison J, Gunther C, Campbell MB, English R, Lazarus C. Critical consciousness as a framework for health equity–focused peer learning. *MedEdPORTAL*. 2021;17:11145.
https://doi.org/10.15766/mep_2374-8265.11145

Introduction

Nationally, issues of diversity, inclusion, and equity are viewed with increasing importance in health care and medical education. For example, the Association of American Medical Colleges addresses health equity, diversity, and inclusion in its mission and has a series of initiatives aimed at assuring that academic medical centers and teaching hospitals are involved in developing solutions to health inequities.[1] In addition, the Liaison Committee on Medical Education's accreditation standards for curricular content require medical curricula to address societal problems, cultural competence and health disparities, and communication skills.[2]

Copyright © 2021 Ellison et al. This is an open-access publication distributed under the terms of the Creative Commons Attribution license.

This publication focuses on how student leaders at the LSU Health New Orleans School of Medicine (LSUHNOSOM), with support from faculty partners, have adopted critical consciousness as a framework to orient student learning about health equity, diversity, and inclusion during the preclinical years. This project, known as Critical Consciousness in Medicine (CCM), is a series of five mandatory workshops in the first year of medical school that introduces students to issues of discrimination and inequity and their effects on health outcomes.

While medical schools across the country are developing curricula to promote student learning about health disparities and health care in multicultural contexts, several institutions have notably invoked critical consciousness as an organizing principle for efforts to guide student learning around sociomedical topics. Kumagai and Lypson outlined the role of critical consciousness in shaping aspects of multicultural education at the University of Michigan Medical School.[3] They described critical consciousness as "an orientation… which places medicine in a social, cultural, and historical context and which is coupled with an active recognition of societal problems and a search for appropriate solutions."[3] They argued that "reading the world"—developing awareness of the differences in power and privilege and of the inequities embedded in social relationships—complements the style of critical thinking more traditionally fostered in medical schools.[3] The University of Michigan approach, as outlined by Kumagai and Lypson, includes student dialogues aimed at developing students' reflective awareness of their own values, worldview, and experiences; faculty development efforts to prepare instructors to facilitate exchanges and critical analysis of personal assumptions, biases, values, and perspectives; and efforts to keep the curriculum current and evaluate its effectiveness through qualitative studies and longitudinal surveys.

A more recent critical consciousness–focused curriculum at the University of Pennsylvania Perelman School of Medicine was developed by a faculty course director in conjunction with a student working group, reflecting a move away from a hierarchical, information-delivery model toward one that provokes students to engage with complex questions.[4] The product of this faculty-student collaboration was a required introductory cultural competence course for first-year students that defines critical consciousness as "a physician's dynamic orientation to navigating the world in three relational domains: *internal* (relationship to self), *interpersonal* (relationships with patients and colleagues/peers), and *structural* (relationship to society, government, culture, health systems, etc.)."[4]

Our project draws inspiration from the work of leaders at Michigan and Penn and aims to contribute to the emerging discourse around critical consciousness as a principal of pedagogy for medical learners. Indeed, the overall goals of our project, drawn directly from Kumagai and Lypson,[3] are to (1) instill professionalism, humanism, and cultural openness and humility in medical students and (2) inspire a worldview that is more complex, inclusive, and oriented towards moral action. Like the curriculum described by Dao and colleagues,[4] our workshops involve collaboration between students and faculty to develop, refine, and sustain the curriculum; a theoretical framework that bridges structural, internal, and interpersonal domains in approaching critical consciousness in medicine; the use of near-peer student facilitators; and instructional content that seeks to prepare students to critically analyze the norms and practices of being a physician.

Our project adds to the emerging body of literature supporting the role of critical consciousness in medical training. Critical consciousness is a topic with limited discussion to date in *MedEdPORTAL,* with existing publications focusing on the application of critical consciousness to onetime educational activities related to faculty development on multicultural education,[5] community empowerment,[6] and responding to racism, discrimination, and microaggressions.[7] The educational content described here overlaps with that of other publications in *MedEdPORTAL,* including discussions of identity,[8-10] privilege,[8] microaggressions,[7,11,12] racism,[13,14] health disparities,[15,16] the social determinants of health,[15,17,18] and implicit bias.[19,20] However, CCM is unique in that it adopts critical consciousness as the unifying concept and approach for a series of student-led workshops spanning these topics for all first-year medical students at our institution. In so doing, we hope to contribute to the growing movement away from medical education in health equity, diversity, and inclusion that is focused on teaching and learning terminology (e.g., the flawed notion of cultural competence) to one that is centered in phenomenology—supporting student inquiry about the lived experience of peers and patients.[21]

Methods

We developed five 2-hour sessions presented to 197 first-year students across 1 academic year.

This program could be implemented for any year in medical school, allopathic or osteopathic, as the curriculum did not require any prerequisite knowledge. We targeted first-year students and held our first session within the first week of

Copyright © 2021 Ellison et al. This is an open-access publication distributed under the terms of the Creative Commons Attribution license.

class. This was done in accordance with our objective to instill a framework with which to view patients and patient interactions before or alongside learning other clinical concepts. Furthermore, choosing first-year students allowed for building a foundation for adding curricular elements throughout medical students' educational careers.

Our program was the second iteration after an initial pilot program (entitled Diversity Forums) had been implemented a year prior with first-year students. The students who were the target audience of that program were interviewed and chosen to lead this iteration of the program by the prior student leaders. Our team included three second-year student leaders and 30 second-year students who volunteered to be facilitators, as well as faculty advisors who assisted with designing each session. Leaders redesigned the curriculum, gathered content, created session materials, and led and moderated each session. Facilitators guided small-group discussions among first-year students.

We created a curriculum of five peer-led workshops, which were delivered across the school year from August 2018 to April 2019. Logistical requirements for each session included space for meetings to hold the students and faculty for training, as well as space for workshops that needed to hold 200 students situated in small groups (10-14) for 2 hours. Each workshop also required use of projectors, audio equipment, computer and internet access, and paper handouts. Additional costs were minimal as these were materials immediately available through the school.

Workshop Series Overview

We planned workshop sessions to coincide with discussion of other topics related to health equity, diversity, and inclusion elsewhere in the LSUHNOSOM preclinical curriculum. We designed the series with content that would build upon each previous session and span levels of the social ecological model, ranging from individual to societal. The use of the social ecological model as a scaffold for the overall workshop was deliberate, as we sought to help students understand the interplay between (inter)personal and structural factors in producing discrimination in health care.

Each workshop had a set of objectives and included a mix of case studies, small- and large-group discussions, videos, and interactive activities designed to inspire further thinking and knowledge procurement regarding the content rather than didactic lecturing. See the Appendices for more detail on each session, including specific learning objectives (Appendices A and B for the workshop 1 presentation and student handout, Appendices C and D for the workshop 2 presentation and student handout, Appendices E and F for the workshop 3 presentation and student handout, Appendix G for the workshop 4 presentation, and Appendix H for the workshop 5 presentation).

Student Facilitators and Student Engagement

At the onset of the year, we recruited students from the second-year class to serve as facilitators. These students were recruited based on their involvement in other campus initiatives promoting health equity and/or campus affinity groups. We utilized the student facilitators in the second and third workshops. These students were trained by student leaders and faculty in an introductory session before the start of the year, and additional briefings were given before each workshop (Appendix I includes facilitator orientation materials). In the trainings before each session, student facilitators role-played as first-year students and discussed how best to moderate discussions. Facilitators were given all case studies and additional readings for review before each workshop. They were also given detailed facilitator guides with minute-by-minute instructions and guidelines for the upcoming workshop (Appendices J-N include the facilitator guides for the five workshops). Student facilitators were not used for the first (introductory) workshop and were not available for the fourth workshop due to a scheduling conflict with the second-year curriculum. For the fifth workshop, we recruited new student leaders from the first-year class; in addition to assuming leadership of the project for the next academic year, these students assisted with facilitation of the final workshop under our leadership (see Recruiting New Student Leaders, below). We used an audience response system technology to facilitate student engagement in the fourth and fifth workshops.

Creating Each Workshop

Our process for creating each workshop involved researching content, case studies, and student activities. We searched for content relevant to our school curriculum as well as local topics and demographics. We used the topics for each session (e.g., privilege, health disparities, etc.) as keywords in our search for case studies and activities among sources including local news media, PubMed for published case studies and articles, and tools such as YouTube for relevant videos.

We sought to ground the workshops in a guiding philosophy—critical consciousness—to inspire students to further research and explore these topics rather than presenting didactic lectures. Each workshop had components intended to do the following:

- Help students understand concepts using lecture-style presentation of definitions and analysis of chosen case studies.

Copyright © 2021 Ellison et al. This is an open-access publication distributed under the terms of the Creative Commons Attribution license.

- Identify a student's own emotions and feelings through activities that explored their own values.
- Initiate dialogue with peers like and unlike through small-group discussions (with groups ranging from six to 14 students, depending on the session), with a second-year student facilitator for workshops 2 and 3.

We designed the workshops to be self-contained (i.e., virtually all work required of student participants was completed within the session). Exceptions to this design included workshop 3, which involved a preworkshop survey related to student privilege, and workshop 5, which was paired with an existing didactic lecture given each year by faculty and required students to complete the Harvard Implicit Association Test prior to the workshop.[22] After creating the layout and choosing content for each session, we met with faculty advisors for feedback and approval, after which we held facilitator training sessions.

Recruiting New Student Leaders

Nearing the end of the academic year, we invited applications for leadership positions for the third iteration of the initiative (the second using this model). We evaluated applicants from the rising second-year class with a written essay application and in-person interviews. We selected four students to assume leadership of the project. As noted above, this group of incoming student leaders also served as facilitators for our fifth and final workshop. This group of students has implemented the workshops for the incoming first-year class and has selected a new group of students to continue the project for another academic year.

Evaluating Feedback

Students were given the opportunity to comment on each workshop via a survey sent out immediately after each workshop. We asked students to rate (on 5-point Likert scales where 1 = *Poor* and 5 = *Excellent*) each component of the session, including effectiveness of case discussions, interactive activities, and any media presented, as well the effectiveness of student leaders and specific group facilitators. Feedback was used to better structure each subsequent session.

At the final workshop, we distributed an end-of-year open-ended reflection exercise and a pre-/postsurvey to assess student learning and engagement and the effectiveness of the series. The instrument asked students to rate their knowledge, skills, and abilities on a 10-point scale (1 = *worst*, 10 = *best*) before and after completing the workshop series. Knowledge, skills, and abilities were directly related to learning objectives from CCM workshops. Students were also asked how CCM had contributed to their growth over the course of the school year. (For a full compilation of evaluation tools used in our workshops, see Appendix O.)

Statistical Analysis

The data for this study were collected via a pre/posttest method. As there was no control group, a paired *t*-test analysis was deemed the most appropriate one to compare students' answers before and after completion of the CCM workshops. All analysis was performed using SAS 9.4.

Results

Participating students were asked to complete the pre/post self-evaluation instrument ($n = 179$, 91% response rate). All students were first-year medical students nearing the end of the academic year; Table 1 includes demographics of student workshop participants. All respondents were presumably present for all five workshops, as the series was a mandatory requirement. Each evaluation question was designed in line with the objectives outlined for the series and each individual workshop.

Results of the tool showed the quantitative change between the average ratings. The ability with the lowest rating before attending the workshop, as well as with the largest quantitative change, was "Ability to define critical consciousness," with a change of 2.5. All prompts had a positive change >1 except "Ability to identify your own value system," which had a change of 0.9. Notably, this prompt had the highest rating already before starting CCM. Table 2 summarizes results of the end-of-year pre-/postsurvey; the Figure displays these results graphically.

The results of the paired *t*-test analysis showed that when comparing student survey answers from the start and end of the school year, there was a significant difference between the answers given on the pretest and those given on the posttest (for all comparisons, $p < .0001$). When also factoring in the average change in self-reported ranking for each question pretest and posttest, the results suggested that the CCM workshops positively influenced students' growth in knowledge, skills, and abilities related to the CCM learning objectives.

Table 1. Demographics of Workshop Participants ($N = 197$)

Demographic	Number	Percentage
Male	105	53.3
Female	92	46.7
Native American or Alaska Native	2	1.0
Asian	40	20.3
Black or African American	16	8.1
Hispanic or Latino	14	7.1
Native Hawaiian or other Pacific Islander	1	0.5
White	131	66.5
Other	3	1.5
Unknown	5	2.5

Copyright © 2021 Ellison et al. This is an open-access publication distributed under the terms of the Creative Commons Attribution license.

Table 2. CCM Pre/Post Self-Evaluation Results

Learning Measure[a]	Learning Objective (Associated Workshop Number)	*M* Self-Rating Before CCM	*M* Self-Rating After CCM	Change	*p*
1	Ability to define critical consciousness (1)	6.1	8.6	2.5	<.0001
2	Ability to identify your own value system (2)	7.9	8.8	0.9	<.0001
3	Ability to identify how a physician's values can shape their decision-making (2)	7.4	8.9	1.5	<.0001
4	Ability to define privilege (3)	7.6	8.8	1.2	<.0001
5	Ability to explore someone else's perspective on privilege (3)	7.2	8.4	1.2	<.0001
6	Ability to define health disparities (4)	7.6	9.1	1.5	<.0001
7	Motivation to work on addressing health disparities as a part of your career in medicine (4)	7.6	8.8	1.2	<.0001
8	Ability to define implicit bias (5)	7.1	8.9	1.8	<.0001
9	Ability to identify and address your own biases (5)	7.3	8.8	1.5	<.0001

Abbreviation: CCM, Critical Consciousness in Medicine workshop.
[a]See the Figure for a graphic display of the learning measure results.

Students also completed a questionnaire composed of open-ended questions ($n = 136$, 69% response rate), including how CCM had contributed to their growth over the course of the school year. A systematic thematic analysis was not performed; however, responses were reflective of notions of continuous learning, self-reflection, and analysis. The most common critiques highlighted the differences among students' individual knowledge at the beginning of the year. There were also dissenting opinions on the role of the covered topics in medical education, as well as how pointed or relaxed the sessions should feel. Themes and critiques from student reflections are presented with associated quotes in Table 3.

Discussion

CCM was developed to expand coverage of health equity, diversity, and inclusion in the LSUHNOSOM curriculum.

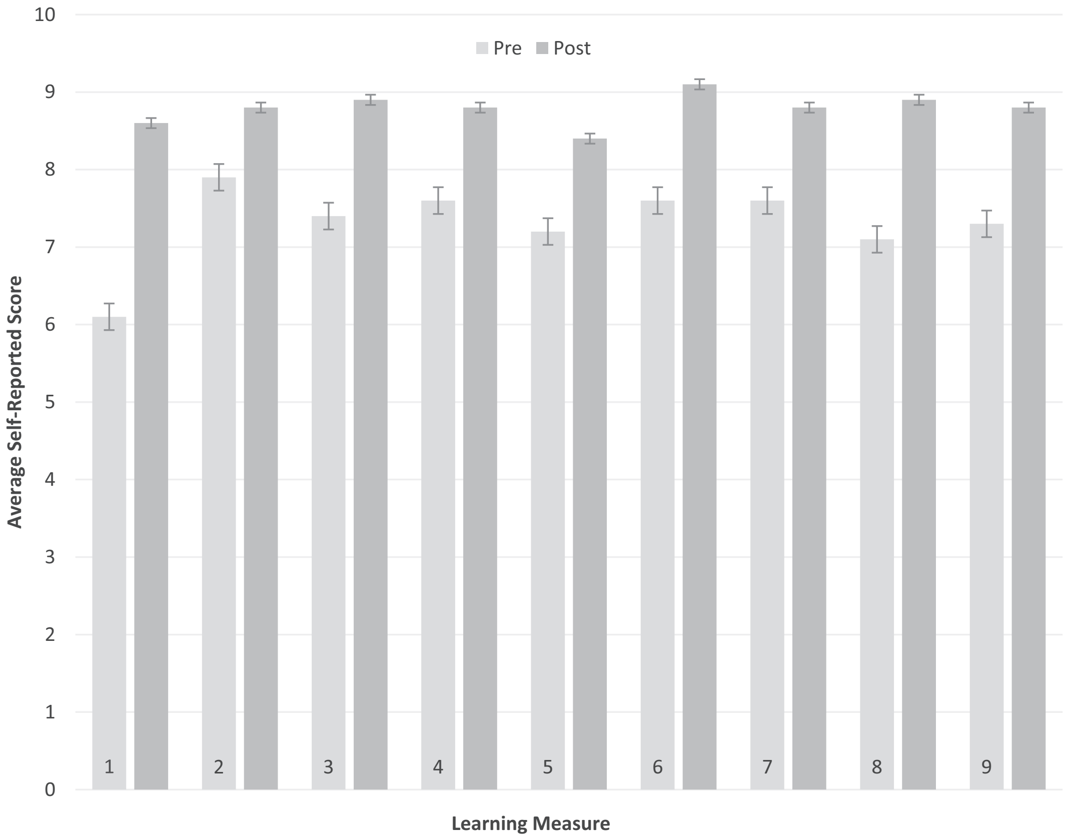

Figure. Critical Consciousness in Medicine workshop pre/post self-evaluation results. Error bars represent the 95% confidence interval for students' average pre/post self-assessments.

Copyright © 2021 Ellison et al. This is an open-access publication distributed under the terms of the Creative Commons Attribution license.

Table 3. Summary Themes and Critiques From Students' Year-End Reflections

Theme	Summary	Representative Quotes From Student Reflections
Learning	Many students mentioned having learned definitions and topics regarding social justice and medicine. Students also expressed new awareness of issues plaguing certain communities and the health care system.	"[CCM workshops] have made me aware of issues that I did not know plagued the healthcare system and forced me to see many issues from others' perspectives which I may not have done without a time such as this dedicated to seeing another person's point of view."
Self-assessment	Students mentioned that the workshops gave them the space and opportunity to reflect on issues they otherwise may have ignored.	"This course has allowed me the platform to evaluate my own thoughts and understand my role in healthcare as a critically conscious physician. I definitely thought more about my personal beliefs and reactions in relation to others throughout these workshops."<br>"The CCM workshops have been invaluable in assessing our own shortcomings as humans and allowing us the opportunity to discuss ways to remedy these human flaws. We are all human and by working together to better ourselves, we can better our communities' health."
Appreciation	Notably, students consistently expressed their appreciation both for the existence of such a program in our school's curriculum and for student leadership and guidance.	"These workshops have been a great learning opportunity. I feel like often, the social aspects of medicine are ignored in lieu of book work. Sometimes I feel that I have become more self-centered and less empathetic because it seems like these qualities are beaten out of us sometimes in our coursework. These workshops recenter me and remind me of the aspects of medicine that excite me about becoming a doctor."
Preexisting knowledge	While many students mentioned having learned much about these topics through the workshops, many also felt they already had knowledge of the concepts and therefore learned little.	"I feel that I am at the same level as I was at the beginning of the school year. I was already aware of these issues, so there was no substantial growth."<br>"To be honest I don't feel that they have contributed to any growth. I have not heard or learned anything I was not already aware of. Maybe the value is in repetition, however."
Nonconfrontational approach	Students commented that the sessions did not sufficiently challenge or provoke students whose views might be in opposition to the presented topics.	"I feel that I already knew a lot about implicit bias. I guess I am disappointed in some aspect of the workshop in that it seems like a 'feel-good' session given by problems that may appease racism and classism. It's all with good intentions but perhaps we can make the students feel more out of their comfort zone for next time."
Peer judgment	Some students felt that feeling judged by peers limited discussion.	"The biggest thing I can say to improve on is to prevent people from judging others that have different views."

Abbreviation: CCM, Critical Consciousness in Medicine.

Specifically, student leaders at our school perceived a need for more discussion about issues of discrimination and inequity during the preclinical years of medical education; we designed a series of peer-led workshops to facilitate student conversation around these topics. Seeking to move beyond cultural competence in these dialogues, we chose to organize our workshops around the notion of critical consciousness, with the aim of teaching an approach to reflection, critical analysis, and learning rooted in the lived experience of self and others that could persist throughout students' subsequent medical education and careers. Critical consciousness has several advantages as an approach to medical education related to equity, diversity, and inclusion, including the following:

- Situating learning as a continuous process of self-reflection and critique, as opposed to the strict acquisition of knowledge or skills implied by the cultural competence model.[3]
- Underscoring the importance of structural factors (alongside inter- and intrapersonal elements) in producing inequities.[4]
- In so doing, motivating students to take action to redress social injustices.[3]
- Establishing a theoretical framework that is malleable yet durable, allowing for an iterative process of curriculum development that adapts and improves with each subsequent delivery.[4]

The results of the CCM workshop series indicated a self-reported gain in ability and knowledge among students as well as a positive attitude and response to such initiatives in the standard medical education curriculum. Student questionnaires also indicated appreciation and acceptance of students developing and leading this type of initiative and activity.

CCM models a student-led, faculty-supported approach to teaching health equity, diversity, and inclusion; indeed, the element of student-faculty partnership is a core part of the project. Student leadership allows for peer-to peer-dialogue on topics that may not otherwise be possible; for example, due to the power structure of the student-faculty dynamic, faculty-led discussions can be limited by a social desirability bias

Copyright © 2021 Ellison et al. This is an open-access publication distributed under the terms of the Creative Commons Attribution license.

(i.e., a student's desire to please the faculty and provide the right answer, rather than an honest opinion). Student leadership addresses some of these issues and may promote increased student engagement. For example, as noted by DallaPiazza and colleagues in their publication describing a similar initiative at Rutgers New Jersey Medical School, student involvement in curriculum development and delivery improves learner engagement and helps make challenging learning experiences more approachable.[13] Our experience with CCM corroborates this finding and further demonstrates medical students' capacity to design and deliver a sequence of multiple health equity–focused peer-learning sessions over the course of an academic year.

Limitations and Considerations for Adaptation

This publication provides an adaptable program outline for teaching a diverse audience of preclinical medical students about topics in health equity, diversity, and inclusion. The project has been designed to instill confidence in students from marginalized groups for a more inclusive and diverse medical education. Requirements for adaptation at other institutions include assembling an organized student team of leaders and appointing faculty intimately involved in the planning of student curriculum and schedules, as well as student affairs, and passionate about social medicine. All aspects of the curriculum, as well as the content of specific workshops, are highly adaptable and can reflect needs—both cultural and logistical—at other institutions. However, our project had several limitations, including limited facilitation experience on the part of our student organizers and facilitators, limited instructional time, challenges related to the group size and mandatory nature of the workshops, and challenges with evaluation.

Our organizing team brought a diverse set of professional and personal experiences to our roles but no experience facilitating for adult learners on the scale of CCM workshops. Similarly, small-group facilitation was inconsistent at times as our small-group facilitators were also recruited from the student body. Facilitators had varying levels of knowledge of the topics covered in the CCM workshops and varying levels of facilitation skill—some were highly skilled facilitators, while others were novices. As a result, in both small-group and whole-room discussions, we often heard from a small segment of students who felt comfortable voicing their opinions; at the same time, other segments of the class were disengaged. Identifying resources to support students in building skill in facilitating discussions among adult learners (e.g., faculty advisors with facilitation experience, formal facilitation trainings for students, etc.) could be useful to train student leaders and facilitators in the future as well as at other institutions implementing this initiative. Standardizing the methods and tactics of discussion moderation among small groups could help to further balance the experience for students.

As mentioned above, our institution mandated the CCM workshops as a part of the first-year curriculum. Overall, this was a unique opportunity to engage students with regard to important issues related to health equity, diversity, and inclusion; however, effectively engaging a class of nearly 200 students was difficult. Organizing mandatory sessions for students to discuss provocative, sometimes personal and/or controversial topics presented unique challenges, as some students were disinclined to actively engage from the outset. Adapting this curriculum as an elective or to be given to an audience not mandated to attend may provide deeper discussions since students are willing participants. However, it could also lead to selection bias, as well as failing to educate many (arguably all) students who might benefit from the series.

Students inevitably entered sessions with a wide spectrum of knowledge and previous experiences related to the topics covered in CCM workshops. As noted in the student critiques in Table 3, some students had a stronger grasp of the issues presented in the workshops and therefore may have learned less from them. We did not conduct preassessments of students to determine baseline levels of knowledge or to identify topics that may have needed extra time in workshops. Going forward, structuring a didactic session before each workshop might be a way to level students' content knowledge coming into each session and let workshops be more focused on allowing students to learn from one another.

Organizing a workshop for a large group also posed logistical challenges, including supplies, room setup, and numbers of facilitators needed. Our institution has large class sizes in which students attend classes and team-based learning activities together as a large group. We have spaces equipped with microphones built into each table and multiple screens equally placed for visibility to all students. Institutions without such spaces can divide larger classes into smaller groups that can fit the space and equipment available. Institutions implementing this initiative can adapt the program to suit the needs of their class based on class size and demographics, noting that these may differ from those of the context where the workshops were originally developed. Due to other curricular demands during the first year, we were limited to five workshops (10 hours total of instructional time). As a result, individual sessions covered multiple topics that could each have merited a full workshop.

Copyright © 2021 Ellison et al. This is an open-access publication distributed under the terms of the Creative Commons Attribution license.

At the same time, the workshop series could also be expanded to cover additional topics. For comparison, the first semester course in the Penn model described above has 43 instructional hours.[4] Adapting institutions could consider expanding the workshop series or increasing meeting frequency to allow for deeper engagement with the workshop content.

At the study design level, the pre/posttest design, with its reliance on self-report, is subject to bias. The risk of bias, and therefore of threats to validity, was increased by the fact that there was no control group. Furthermore, the concept of critical consciousness is difficult to measure objectively due to its practice-based and context-specific nature; as a result, the measures presented here were at best proxies of students' critical consciousness. In the future, incorporating and analyzing qualitative interviews would help with understanding the impact the CCM workshops have on student learning and growth related to the CCM learning objectives.

Future Directions
At LSUHNOSOM, the first-year workshop series has continued under two successive generations of new student leaders following our implementation. These subsequent implementations have helped to standardize core CCM content while allowing for variation and student creativity, balancing between consistency and evolution. For example, one group of leaders maintained the overall structure and content of CCM workshops but rearranged their order to better introduce key concepts and facilitate student understanding.

Since our implementation, LSUHNOSOM has added mandatory CCM sessions in the second year focused specifically on issues affecting marginalized populations, such as individuals who are or have been incarcerated and individuals who have disabilities. There is also a second-year CCM elective for motivated students interested in deepening their understanding of the issues raised in the workshop series and cultivating their leadership skills to promote health equity. Our institution continues to develop plans to ensure that health equity, diversity, and inclusion are addressed throughout all 4 years of medical education, not just during the preclinical years. Eventually, we hope this will culminate in a clear, comprehensive 4-year course of training in health equity, diversity, and inclusion with the possibility for a certificate of distinction and/or scholarship support for students who dedicate themselves to this track of study. We encourage all institutions, including our own, to develop understanding, institutional capacity (e.g., consider hiring dedicated staff with expertise in these areas), and community partnerships centered in health equity, diversity, and inclusion to ensure that these issues are fully and effectively addressed throughout all 4 years of students' medical education.

Appendices

A. Workshop 1 Presentation.pptx
B. Workshop 1 Student Handout.docx
C. Workshop 2 Presentation.pptx
D. Workshop 2 Student Handout.docx
E. Workshop 3 Presentation.pptx
F. Workshop 3 Student Handout.docx
G. Workshop 4 Presentation.pptx
H. Workshop 5 Presentation.pptx
I. Facilitator Orientation.pptx
J. Workshop 1 Facilitator Guide.docx
K. Workshop 2 Facilitator Guide.docx
L. Workshop 3 Facilitator Guide.docx
M. Workshop 4 Facilitator Guide.docx
N. Workshop 5 Facilitator Guide.docx
O. Evaluation Tools.docx

All appendices are peer reviewed as integral parts of the Original Publication.

Jonte Ellison: Fourth-Year Medical Student, LSU Health New Orleans School of Medicine

Chris Gunther, MPH: Fourth-Year Medical Student, LSU Health New Orleans School of Medicine; ORCID: https://orcid.org/0000-0002-7955-0560

Mary Beth Campbell, MSPH: Graduate Student, Tulane University School of Public Health and Tropical Medicine

Robin English, MD: Assistant Dean for Undergraduate Medical Education, LSU Health New Orleans School of Medicine

Cathy Lazarus, MD: Associate Dean for Student Affairs, LSU Health New Orleans School of Medicine

Acknowledgments
We wish to acknowledge the LSU New Orleans School of Medicine students who initiated this project in 2017-2018—Hillary Gary, Anthony Pham, and De'Angellica Vaughn—as well as the students who have continued it: Joe Goddu, Kaila Holliday, Tierra Moultrie, and Tina Nguyen in 2019-2020, and Will Boles, Maddie Degrange, Mima Fondong, and Lamiya Tauhid in 2020-2021.

Disclosures
None to report.

Copyright © 2021 Ellison et al. This is an open-access publication distributed under the terms of the Creative Commons Attribution license.

Funding/Support
None to report.

Prior Presentations
Ellison J, Gunther C, Campbell MB, et al. Critical consciousness in medicine: student-led workshops increase engagement around social issues in health care. Poster presented at: Learn Serve Lead: The AAMC Annual Meeting; November 8, 2019; Phoenix, AZ.

Ethical Approval
Reported as not applicable.

References

1. Diversity & inclusion. Association of American Medical Colleges. Accessed March 9, 2021. https://www.aamc.org/what-we-do/mission-areas/diversity-inclusion
2. Liaison Committee on Medical Education. *Functions and Structure of a Medical School: Standards for Accreditation of Medical Education Programs Leading to the MD Degree.* Liaison Committee on Medical Education; 2020.
3. Kumagai AK, Lypson ML. Beyond cultural competence: critical consciousness, social justice, and multicultural education. *Acad Med*. 2009;84(6):782-787. https://doi.org/10.1097/ACM.0b013e3181a42398
4. Dao DK, Goss AL, Hoekzema AS, et al. Integrating theory, content, and method to foster critical consciousness in medical students: a comprehensive model for cultural competence training. *Acad Med*. 2017;92(3):335-344. https://doi.org/10.1097/ACM.0000000000001390
5. Lypson M, Ross P, Joiner T, Kumagai A. Using multimedia in faculty development on multicultural education: scenes from the movie "Crash." *MedEdPORTAL*. 2010;6:8008. https://doi.org/10.15766/mep_2374-8265.8008
6. Kuhle C, Varkey P. Enhancing the health of communities: a primer on empowerment. *MedEdPORTAL*. 2012;8:8400. https://doi.org/10.15766/mep_2374-8265.8400
7. Sotto-Santiago S, Mac J, Duncan F, Smith J. "I didn't know what to say": responding to racism, discrimination, and microaggressions with the OWTFD approach. *MedEdPORTAL*. 2020;16:10971. https://doi.org/10.15766/mep_2374-8265.10971
8. Chow CJ, Case GA, Matias CE. Tools for discussing identity and privilege among medical students, trainees, and faculty. *MedEdPORTAL*. 2019;15:10864. https://doi.org/10.15766/mep_2374-8265.10864
9. Bi S, Vela MB, Nathan AG, et al. Teaching intersectionality of sexual orientation, gender identity, and race/ethnicity in a health disparities course. *MedEdPORTAL*. 2020;16:10970. https://doi.org/10.15766/mep_2374-8265.10970
10. Elliott D. Cultural self-awareness workshop. *MedEdPORTAL*. 2009;5:1128. https://doi.org/10.15766/mep_2374-8265.1128
11. Acholonu RG, Cook TE, Roswell RO, Greene RE. Interrupting microaggressions in health care settings: a guide for teaching medical students. *MedEdPORTAL*. 2020;16:10969. https://doi.org/10.15766/mep_2374-8265.10969
12. Sandoval RS, Afolabi T, Said J, Dunleavy S, Chatterjee A, Ölveczky D. Building a tool kit for medical and dental students: addressing microaggressions and discrimination on the wards. *MedEdPORTAL*. 2020;16:10893. https://doi.org/10.15766/mep_2374-8265.10893
13. DallaPiazza M, Padilla-Register M, Dwarakanath M, Obamedo E, Hill J, Soto-Greene ML. Exploring racism and health: an intensive interactive session for medical students. *MedEdPORTAL*. 2018;14:10783. https://doi.org/10.15766/mep_2374-8265.10783
14. Brooks KC, Rougas S, George P. When race matters on the wards: talking about racial health disparities and racism in the clinical setting. *MedEdPORTAL*. 2016;12:10523. https://doi.org/10.15766/mep_2374-8265.10523
15. Neff J, Holmes SM, Knight KR, et al. Structural competency: curriculum for medical students, residents, and interprofessional teams on the structural factors that produce health disparities. *MedEdPORTAL*. 2020;16:10888. https://doi.org/10.15766/mep_2374-8265.10888
16. Kutscher E, Boutin-Foster C. Community perspectives in medicine: elective for first-year medical students. *MedEdPORTAL*. 2016;12:10501. https://doi.org/10.15766/mep_2374-8265.10501
17. Song AY, Poythress EL, Bocchini CE, Kass JS. Reorienting orientation: introducing the social determinants of health to first-year medical students. *MedEdPORTAL*. 2018;14:10752. https://doi.org/10.15766/mep_2374-8265.10752
18. Drake C, Keeport M, Chapman A, Chakraborti C. Social contexts in medicine: a patient-centered curriculum empowering medical students to provide contextualized care. *MedEdPORTAL*. 2017;13:10541. https://doi.org/10.15766/mep_2374-8265.10541
19. Perdomo J, Tolliver D, Hsu H, et al. Health Equity Rounds: an interdisciplinary case conference to address implicit bias and structural racism for faculty and trainees. *MedEdPORTAL*. 2019;15:10858. https://doi.org/10.15766/mep_2374-8265.10858
20. West TJ, Loomer K, Wyatt TR. How diverse is your universe? An activity for students to reflect on ethnoracial diversity during orientation. *MedEdPORTAL*. 2019;15:10840. https://doi.org/10.15766/mep_2374-8265.10840
21. Neubauer BE, Witkop CT, Varpio L. How phenomenology can help us learn from the experiences of others. *Perspect Med Educ*. 2019:8(2):90-97. https://doi.org/10.1007/s40037-019-0509-2
22. Project Implicit. Harvard University. Accessed May 14, 2020. https://implicit.harvard.edu/implicit

Received: May 28, 2020
Accepted: February 18, 2021
Published: April 28, 2021

Copyright © 2021 Ellison et al. This is an open-access publication distributed under the terms of the Creative Commons Attribution license.
